# Effect of Pegcetacoplan on Aqueous Humor Proteome in Geographic Atrophy: A Prospective Exploration

**DOI:** 10.1167/iovs.66.15.24

**Published:** 2025-12-05

**Authors:** Omer Trivizki, Charles C. Wykoff, Magda A. Smoor, David Rabinovitch, Avery Zhou, Jessica A. Cao, Yara T. E. Lechanteur, Lambert van den Heuvel, Alain J. van Gool, Jolein Gloerich, Joëlle E. Vergroesen, Caroline C. W. Klaver

**Affiliations:** 1 Tel Aviv Medical Center, Sackler Faculty of Medicine, Tel Aviv University, Israel; 2 Bascom Palmer Eye Institute, University of Miami, Miami, Florida, United States; 3 Retina Consultants of Texas, Houston, Texas, United States; 4 Department of Ophthalmology, Erasmus Medical Center, Rotterdam, the Netherlands; 5 Department of Epidemiology, Erasmus Medical Center, Rotterdam, the Netherlands; 6 Department of Ophthalmology, Radboud University Medical Center, Nijmegen, the Netherlands; 7 Department of Pediatric Nephrology, Radboud Institute for Molecular Life Sciences, Amalia Children's Hospital, Radboud University Medical Center, Nijmegen, the Netherlands; 8 Department of Pediatrics/Pediatric Nephrology, University Hospitals Leuven, Leuven, Belgium; 9 Department of Development and Regeneration, University Hospitals Leuven, Leuven, Belgium; 10 Translational Metabolic Laboratory, Department of Human Genetics, Radboud University Medical Center, Nijmegen, the Netherlands; 11 Institute of Molecular and Clinical Ophthalmology, Basel, Switzerland

**Keywords:** age-related macular degeneration, geographic atrophy, pegcetacoplan, proteomics, mass spectrometry

## Abstract

**Purpose:**

Pegcetacoplan, a complement component 3 (C3) inhibitor, has recently received U.S. Food and Drug Administration approval for treating geographic atrophy (GA), an advanced stage of age-related macular degeneration (AMD). However, the limited treatment response prompts further investigations into its molecular effects.

**Methods:**

We prospectively collected aqueous humor (AH) samples from 11 patients with GA before and at 2 months during pegcetacoplan treatment. Liquid chromatography with tandem mass spectrometry (LC-MS/MS) was used to analyze the proteome, and global normalization was applied to account for differences in protein concentration. To assess global proteomic shifts over time, principal component analysis (PCA) was performed. The Friedman test was used to assess differences in protein intensities across time points while adjusting for multiple testing using Benjamini–Hochberg false discovery rate (FDR) correction.

**Results:**

A total of 283 proteins were analyzed. PCA revealed temporal changes in global protein expression profiles, with a significant shift between baseline and month 2 (*P* = 0.01). The levels of complement components C3 (*P* = 0.12) and C5 (*P* = 0.27) remained stable after initiation of treatment, but the levels of C1qB, C1RL, C2, C6, C7, C8, C9, factor D, factor H, and factor I increased significantly (all *P* < 0.05). Most non-complement proteins showed no significant changes; however, beta-2–glycoprotein 1 (FDR = 0.09), kininogen 1 (FDR < 0.05), and prothrombin (FDR < 0.05) increased significantly, and kallistatin (FDR < 0.05) and plasma serine protease inhibitor (FDR < 0.05) decreased.

**Conclusions:**

This exploratory study suggests that pegcetacoplan modulates the AH proteome in GA. Although no changes in the target protein C3 were detected following treatment, significant changes in proteins tightly connected to complement, inflammation, and coagulation were observed. These findings underscore the need for further investigation into the biological and clinical relevance of the observed molecular shifts.

Age-related macular degeneration (AMD) is a leading cause of vision loss, particularly in the elderly. Geographic atrophy (GA), an advanced form of non-neovascular AMD, affects over 5 million people worldwide.^1^ The pathogenesis of AMD, especially its dry form characterized by GA, has been linked to the complement system, a key component of the innate immune response.^2^^,^^3^

The complement system, consisting of numerous serum and membrane-associated proteins, plays a critical role in host defense by recognizing, tagging, and eliminating foreign particles. It is activated through three pathways—classical, lectin, and alternative—which converge on the activation of complement component 3 (C3), splitting into C3a and C3b and further amplifying the complement cascade. The result of complement activation is inflammation, cell destruction, and apoptosis. The regulation of C3 convertase by factors such as decay-accelerating factor (DAF), factor H (FH), and factor I (FI) underscores the complexity of the system in modulating immune responses.^4^

The correlation between AMD and complement system dysregulation has been reported by many genetic epidemiologic studies linking variants in complement proteins to increased AMD risk. Common and rare variants in complement factor H (*CFH*), C3, C9, complement factor I (*CFI*), transmembrane protein 97 (*TMEM97*)/vitronectin (*VTN*) determine a large proportion of in particular the late stages of AMD.^5^^–^^7^ Many of these variants disrupt complement regulation, leading to persistent inflammation that contributes to AMD. Additionally, elevated complement activation markers have been detected in the systemic circulation,^2^^,^^8^ many complement components are found in drusen,^9^ and the membrane attack complex (MAC) is significantly increased in the choroid of AMD patients.^10^ Together, these findings underscore the critical contribution of the complement system to the pathogenesis of AMD, particularly in promoting inflammation and cellular lysis.^11^

In 2023, pegcetacoplan (Syfovre; Apellis Pharmaceuticals, Waltham, MA, USA), a compstatin-based complement inhibitor, became the first U.S. Food and Drug Administration–approved intravitreal treatment for GA. Pegcetacoplan selectively binds to C3 and its activation fragment C3b, preventing downstream activation of the cascade, including cleavage of C5 and formation of the MAC. Via intravitreal administration, the drug aims to target complement dysregulation in retina, retinal pigment epithelium, and choroid, ultimately slowing GA progression.^12^

The effectiveness of pegcetacoplan in treating GA secondary to AMD has been assessed in the FILLY phase 2 and OAKS and DERBY phase 3 clinical trials.^13^ Although these studies demonstrated significant reductions in the growth rate of GA lesions and more sustained retinal functionality measured by microperimetry, prespecified evidence for a difference in central visual acuity between the treatment and sham arm could not be provided. Moreover, inconsistent outcomes from other clinical trials question the overall utility of complement inhibitors.^12^^,^^14^^–^^17^ Although the development of complement inhibitors is regarded as a major breakthrough in treatment for GA, the impact of pegcetacoplan on the complement system at the local tissue level remains unclear.

Mass spectrometry technology has been instrumental in enabling comparative analysis of proteomics in small volumes of human samples. Previous studies analyzing vitreous humor (VH) have identified associations between VH protein profiles and retinal diseases, including AMD.^18^^–^^21^ However, obtaining VH samples is challenging, poses risks to patients, and is generally not feasible in routine clinical practice, hindering its broad application as a biomarker source in clinical research settings. In contrast, collecting aqueous humor (AH) samples is more readily possible and safely accomplished in clinical practice, prompting recent investigations into the potential of AH proteins to serve as a viable and convenient alternative for VH biomarker studies. Recent findings have demonstrated a strong correlation between specific complement levels in AH and VH.^22^ Rinsky et al.^23^ detected elevated complement proteins such as C3 in AH from AMD patients and compared their findings to previously published VH datasets.^19^ Although direct AH–VH correlation was not performed, their results support overlapping complement dysregulation. These authors also validated relative protein abundance determined by liquid chromatography–mass spectrometry (LC-MS) and ELISA.^23^ Hence, MS proteomic analyses of AH appear to offer a more feasible analysis of the intraocular complement landscape than VH. Nevertheless, it is important to emphasize that AH proteomics should be viewed as exploratory, providing indirect insights into local pharmacodynamic changes rather than serving as a direct proxy for retinal or vitreous complement activity. To date, no studies have examined AH protein profiles longitudinally before and after complement inhibition therapy, highlighting the novelty of our exploratory approach. The current proteome study employed a unique exploratory design to evaluate the effect of intravitreal pegcetacoplan on AH protein profiles among patients with GA secondary to AMD. By analyzing these levels before and after administration of the drug at two time points, we aimed to provide insight into the molecular consequences of complement inhibition therapy in the eye.

## Methods

### Study Design

This prospective, interventional, exploratory study aimed to evaluate the levels of complement proteins in the AH of patients with AMD and to compare these levels before and after pegcetacoplan administration for GA. The study protocol was approved by the local ethics committee, received institutional review board approval, and adhered to the tenets of the Declaration of Helsinki. All participants provided written informed consent.

### Study Population

Between March and September 2023, 11 consecutive AMD patients (including 12 eyes) were recruited from the Retina Consultants of Texas. All patients underwent a comprehensive ophthalmologic examination including best-corrected visual acuity, intraocular pressure measurement, and optical coherence tomography. In addition, fundus autofluorescence was performed. Clinical histories of non-ocular diseases and treatments were retrieved from electronic medical records. Inclusion criteria included a diagnosis of treatment-naïve GA secondary to AMD with no other clinically relevant ocular or systemic comorbidities. However, the presence of concomitant exudative AMD was permitted. Exclusion criteria included retinal atrophy due to causes other than AMD or the presence of other retinal diseases. After the baseline diagnosis, AMD patients received monthly intravitreal injections of pegcetacoplan (100 µL, 15 mg).

### Sample Collection

AH samples were collected from the anterior chamber of all patients at baseline and following the monthly administration of pegcetacoplan via intravitreal injection at Months 1 and 2. The procedure involved the aspiration of approximately 100 µL of AH using a fine-gauge needle, performed under sterile conditions and local anesthesia. Within 5 minutes after collection, the samples were placed on ice and immediately transferred to –80°C to preserve protein integrity for the subsequent analyses. Saliva samples were collected for genetic analyses using sterile tubes that contained a preservative solution for stabilization of the DNA. Samples were stored at 4°C and subsequently processed for DNA extraction. Although AH may not fully reflect the vitreous proteome, its clinical accessibility makes it a practical surrogate for longitudinal molecular monitoring in exploratory studies. Support for the notion that AH reflects vitreous changes comes from recent studies demonstrating marked reductions in vascular endothelial growth factor (VEGF) and angiopoietin-2 (Ang-2) levels following intravitreal faricimab injections.^24^^–^^26^

### Genetic Risk Analyses

Genotyping was performed for known AMD risk variants using the Illumina Global Screening Array, version 3, with custom content as described earlier.^27^ Genetic risk scores (GRSs) were calculated by summing the weighted effects of the established 52 common risk variants identified through genome-wide association analyses^6^ using the formula:

$$\mathrm{GRS}=\sum_{i=1}^{n}\left(\mathrm{Effect}\;\mathrm{Siz}e_{i}\times \mathrm{Genotyp}e_{i}\right)$$

In addition to the common variants, we screened for rare AMD risk variants in *C3*, *CFH*, and *CFI* genes using the same array.

### AH Sample Preparation

After thawing, 3 µL of each sample was digested as described previously, with minor modifications.^28^ Briefly, each sample was diluted 1:1 with 8-M urea to denature proteins, after which proteins were reduced with dithiothreitol, and reduced cysteines were alkylated through incubation with 2-choloacetamide (CAA) in the dark. Samples were subsequently 1:3 diluted with 50-mM ammonium bicarbonate, after which trypsin was added for overnight digestion of the proteins at 37°C. To quench the reaction, samples were diluted with trifluoroacetic acid (TFA) to an end concentration of 1% TFA, resulting in an end volume of 47 µL.

### Analysis of AH Proteome With LC-MS

All proteomic analyses were performed at the Translational Metabolic Laboratory (Radboud University Medical Center, Nijmegen, the Netherlands) under the supervision of Jolein Gloerich, PhD, and Alain van Gool, PhD. Peptides were separated on an Evosep One LC system (Evosep Biosystems, Odense, Denmark) coupled online to a hybrid trapped ion mobility spectrometry (TIMS) quadrupole time-of-flight (TOF) mass spectrometer (timsTOF Pro 2; Bruker, Bremen, Germany) via a CaptiveSpray nano-electrospray ion source using C18 Evotips (Evosep Biosystems). According to manufacturer's instructions, 2 µL of digested sample was loaded onto the C18 Evotips and separated onto a 15-cm C18 column (ReproSil-Pur C18, 150-µm inner diameter, 1.5-µm particle size; Evosep Biosystems) using the manufacturer's predefined method, 30 SPD, with a linear gradient of buffer A (HPLC-grade water with 0.1% formic acid) and buffer B (acetonitrile with 0.1% formic acid).^29^ Eluted peptides were analyzed on the timsTOF Pro 2 mass spectrometer operated in positive ion mode with enabled TIMS at 100% duty cycle (100 ms ramp time). Scan mode was set to the standard data independent acquisition parallel accumulation-serial fragmentation (DIA-PASEF) method for long gradients. Briefly, 32 isolation windows of 26 *m*/*z* width spanning from 400 to 1200 *m*/*z* were defined, with *m*/*z* of 1 overlap between the windows (on each side of a given window). After an MS1 scan, two isolation windows were fragmented per TIMS ramp. The collision energy was set to rise linearly over the covered mobility range (for 1/*K*_0_ values between 0.60 and 1.60 Vs/cm^2^, corresponding to 20 eV to 59 eV). All samples were measured in triplicate.

### Data Processing

Real-time data analysis was performed with the Parallel Search Engine in Real-Time (PaSER; Bruker), using an in-house–generated spectral library and LC-MS data recorded with the same instrument setup operated in data-dependent data acquisition mode (DDA) with parallel accumulation–serial fragmentation (PASEF) from LC-MS analysis of recombinant complement protein standards (complement factor B [CFB], complement factor D [CFD], CFH, CFI, complement factor P [CFP], C2, C3, C4, C5, C6, C7, C8, C9, and complement factor H–related protein 1 [CFHR-1], –2, –4, and –5); healthy subject plasma samples; VH and AH samples; and a database of the human proteome (UniProt, reviewed sequences). Real-time searches were performed using TIMS–data independent acquisition–neural network (TIMS DIA-NN) processing^30^ with precursor and fragment mass tolerances set to 20 and 15 ppm, respectively. Cysteine carbamidomethylation was set as a fixed modification and methionine oxidation as a variable modification. The false discovery rate (FDR) was set to 0.01, and the top three precursors were used for calculation of label-free quantitation (LFQ) protein intensities. Results were combined using match between runs (MBR) with an outlier frequency of 0.2 with global normalization in DIA-NN.

### Statistical Analysis

Consistency and repeatability of the data per sample were confirmed using correlation heatmaps, which demonstrated high reliability across sample analyses (data not shown). Differences in protein intensities were evaluated over time at three key time points: at baseline and at Month 1 and Month 2 after the start of the treatment regimen. The mean of the three replicates was calculated per protein; proteins with more than 25% missing values were excluded. To assess global proteomic shifts over time, principal component analysis (PCA) was performed on the expression values of 283 proteins using the pcaMethods package in R (R Foundation for Statistical Computing, Vienna, Austria), applying the probabilistic PCA (PPCA) method to accommodate missing values. Protein expression values were standardized prior to analysis. PCA scores (PC1 and PC2) were extracted and used to visualize patient-specific trajectories at baseline and after pegcetacoplan treatment (Months 1 and 2). In a subanalysis, PCA was also performed on proteins belonging to the complement pathway only. Wilcoxon signed-rank tests were conducted on PC1 and PC2 values to evaluate proteomic changes between visits. Changes in protein intensities over time at the individual level were visualized with spaghetti plots, and group-level changes were displayed with box plots. The Friedman test was used to assess differences in protein intensities across time points. Post hoc comparisons for non-parametric data were performed using the Wilcoxon signed-rank test. Multiple testing correction was performed using the Benjamini–Hochberg procedure. For each protein, the unadjusted *P* value is reported; when a protein remained significant after FDR correction (FDR < 0.05), the FDR-corrected *P* value is indicated. A circular heatmap was generated to illustrate relative changes in intensities of complement proteins after pegcetacoplan treatment. Proteins with significant longitudinal changes (Friedman test *P* < 0.05) were selected for pathway enrichment analysis using the Reactome database, utilizing the enrichPathway() function from the ReactomePA package. The top 10 enriched pathways (FDR < 0.05) were visualized using a network-based approach. Pathway–protein relationships were visualized using ggraph. All statistical analyses were performed using R.

## Results

### Patient Characteristics and Genetic Risk

Baseline characteristics are shown in the Table. Twelve eyes from 11 AMD patients were included; seven eyes had pure GA and five had GA concomitant with macular neovascularization (MNV) which had been treated with 2.0-mg aflibercept (Eylea; Regeneron Pharmaceuticals, Tarrytown, NY, USA). Ten patients were female; the mean age was 83 years. All AMD patients had a high genetic risk (GRS > 1.13); three patients could even be classified as having a very high genetic risk (GRS > 3).^5^ All but one were carriers of the common risk variant Y402H in *CFH*, and six were carriers of R102G in *C3*. None of the AMD patients carried rare, high-penetrant risk variants in *CFH*, *CFI*, or *C3*.

**Table.** Patient Characteristics and Genetic Risk Profiles

Patient No.	AMD Status	Prior Treatment	Gender	Age (Y)	Eye	Genetic Risk Score	*CFH* Y402H	*C3* R102G
1	GA	NA	F	76	OS	3.37	Het	Het
2	GA	NA	F	93	OS	3.10	Hom	Non-risk
3	GA	NA	F	81	OS	3.10	Het	Non-risk
	GA+MNV	Aflibercept	F	81	OD	3.10	Het	Non-risk
4	GA	NA	M	81	OS	2.91	Hom	Het
5	GA	NA	F	83	OS	1.71	Het	Het
6	GA	NA	F	78	OD	1.95	Hom	Non-risk
7	GA	NA	F	84	OS	1.55	Hom	Non-risk
8	GA+MNV	Aflibercept	F	80	OD	2.59	Het	Het
9	GA+MNV	Aflibercept	F	80	OS	1.68	Het	Non-risk
10	GA+MNV	Aflibercept	M	72	OS	1.42	Het	Hom
11	GA+MNV	Aflibercept	F	92	OS	1.45	Non-risk	Het

F, female; Het, heterozygous risk variant; Hom, homozygous risk variantl M, male; NA, not applicable; OD, oculus dexter; OS, oculus sinister.

### Global Changes in Proteomic Profile After Pegcetacoplan Treatment

**Figure 1.**
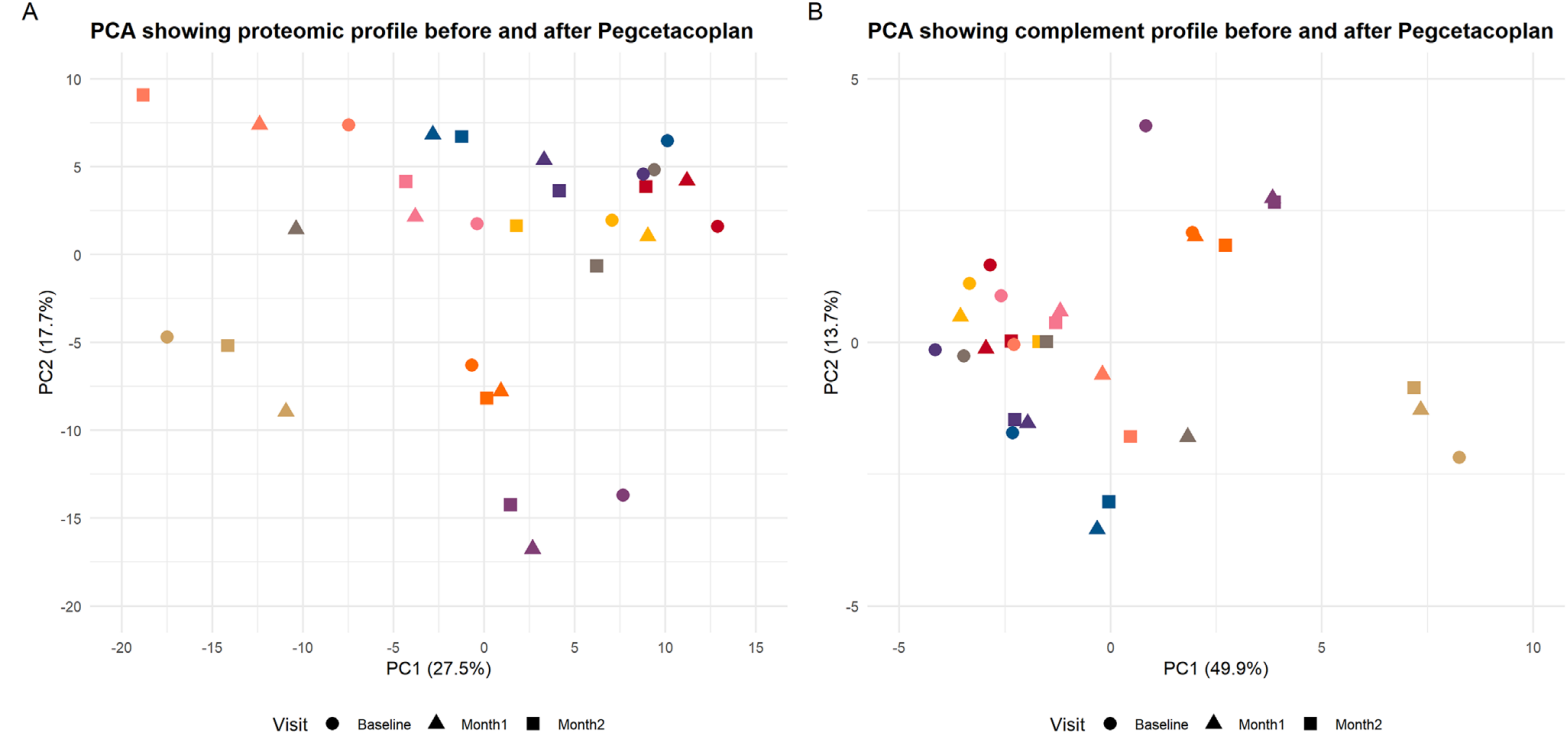
**PCA of proteomic profiles at baseline and after pegcetacoplan treatment (Month 1 and Month 2).** (**A**) PCA of the full proteomic dataset (283 proteins) shows patient-level shifts over time. Each point represents a sample, color-coded by patient and shaped by visit. PC1 and PC2 explained 27.5% and 17.7% of total variance, respectively. A significant shift along PC1 was observed from baseline to Month 2 (*P* = 0.01). (**B**) PCA of complement proteins shows a similar trend, with PC1 and PC2 explaining 49.9% and 13.7% of variance. A significant change in PC1 occurred between baseline and Month 2 (*P* = 0.01), but not at earlier time points.

PCA of the full proteomic dataset revealed temporal changes in global protein expression profiles (Fig. 1A). Visual inspection showed that baseline samples tended to cluster separately from post-treatment samples, particularly along PC1. There was a significant shift between baseline and Month 2 (*P* = 0.01), but not between baseline and Month 1 (*P* = 0.13) or between Month 1 and Month 2 (*P* = 0.38). No significant differences were observed in PC2 across time points, suggesting that the treatment-associated variation occurred primarily along PC1. In the complement protein subset, a similar temporal trend was observed, with modest separation of baseline and post-treatment samples along PC1 (Fig. 1B). There was a statistically significant shift between baseline and Month 2 (*P* = 0.01), but the change between baseline and Month 1 did not reach significance (*P* = 0.06). No significant difference was observed between Month 1 and Month 2 (*P* = 0.49). These findings suggest that complement proteins also respond to treatment, but the effect becomes apparent primarily at Month 2 and mirrors the pattern observed in the global proteome.

### Effect of Pegcetacoplan on AH Proteome Including Complement Proteins

Supplementary Figures S1 to S283 depict the spaghetti and box plots of the protein intensities of the 283 proteins before and after pegcetacoplan. Similar to baseline, the interindividual variation in response to treatment was substantial for many proteins. Consistent protein intensities were observed for C3 (Friedman *P* = 0.12) (Fig. 2) and C5 (Friedman *P* = 0.27) (Fig. 3) before and after treatment with pegcetacoplan. The proportional differences in intensities of all complement-associated proteins at baseline, Month 1, and Month 2 are provided in the circular heatmap shown in Figure 4. Most proteins showed statistically significant but only slightly increased intensities after treatment; however, C6 increased by approximately 200%. The C1s subcomponent and C4A were the only complement proteins for which the intensities decreased during treatment, albeit not significantly. Supplementary Figures S284 to S306 show box plots of the complement-associated protein intensities at baseline, Month 1, and Month 2, stratified on GRS (1–2, 2–3, >3), *CFH* Y402H variant (non-risk, heterozygous, homozygous), *C3* R102G variant (non-risk, heterozygous, homozygous), age, sex, and AMD status. No striking trends in treatment response were observed between genetic risk profiles, although values prior to treatment sometimes appeared higher (C3 in Supplementary Fig. S291) or lower (CFH in Supplementary Fig. S303) in carriers of risk variants.

**Figure 2.**
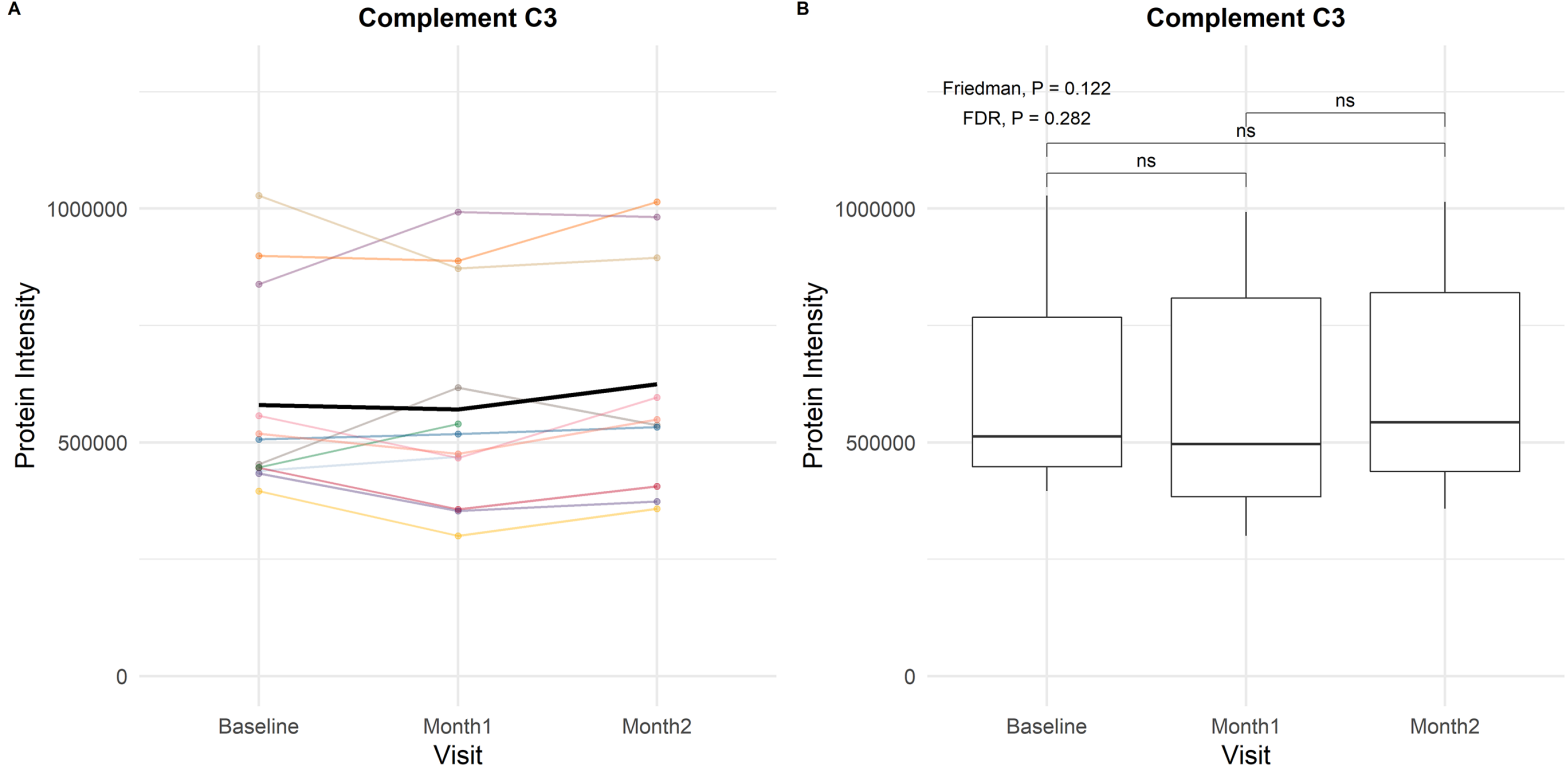
**C3 protein intensities before and after monthly pegcetacoplan treatment.** (**A**) Line plot illustrating individual patient trajectories of C3 protein intensity over time. The *bold black line* indicates the mean intensity over time. (**B**) Box plots depicting the distribution of C3 intensity at baseline, Month 1, and Month 2. Only AMD patients with measurements at all visits are included. The median, interquartile range, and outliers are displayed for each time point; ns, not significant.

**Figure 3.**
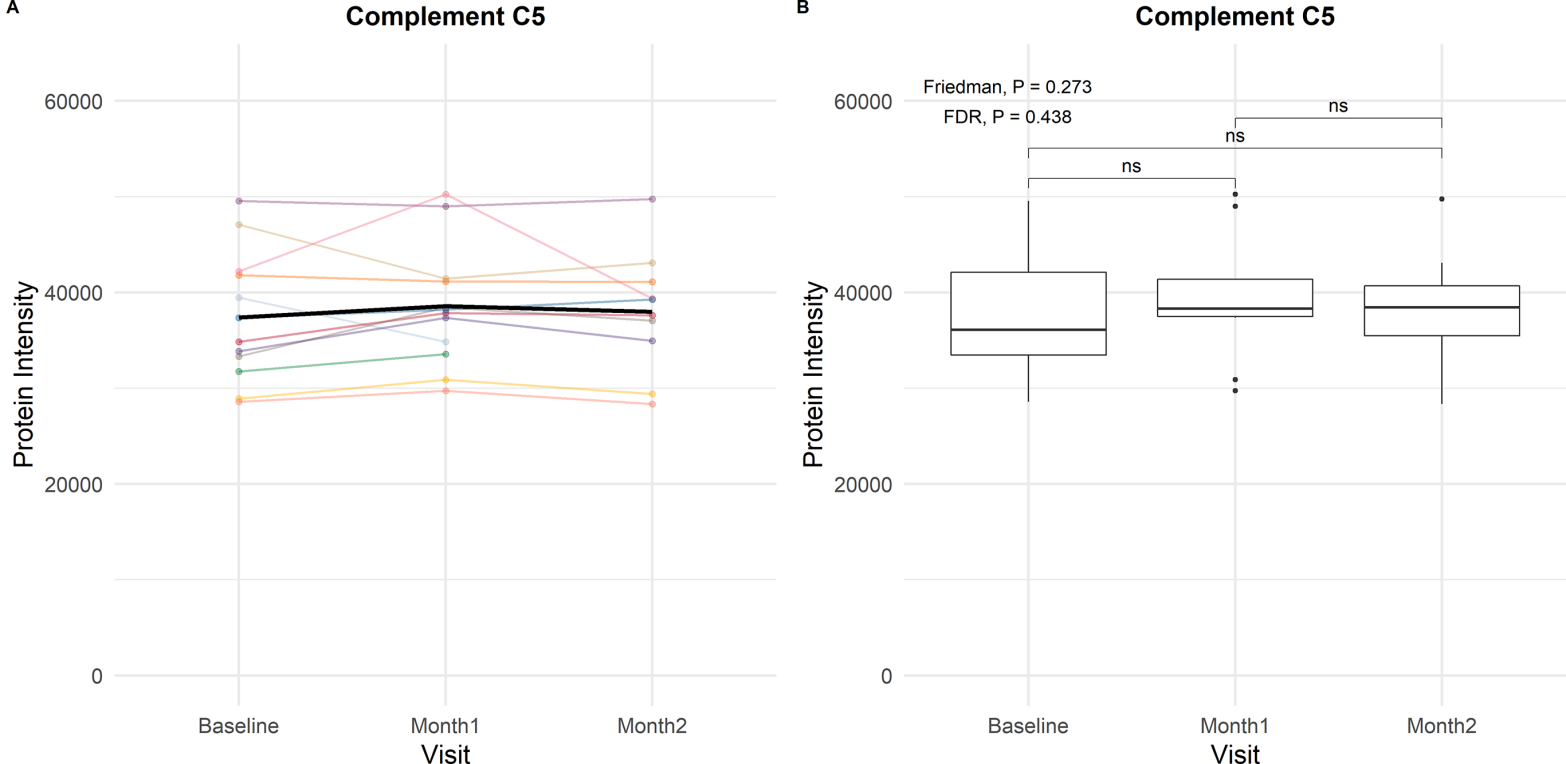
**C5 protein intensities before and after monthly pegcetacoplan treatment.** (**A**) Line plot illustrating individual patient trajectories of C5 protein intensity over time. The *bold black line* indicates the mean intensity over time. (**B**) Box plots depicting the distribution of C5 intensity at baseline, Month 1, and Month 2. Only AMD patients with measurements at all visits are included. The median, interquartile range, and outliers are displayed for each time point.

**Figure 4.**
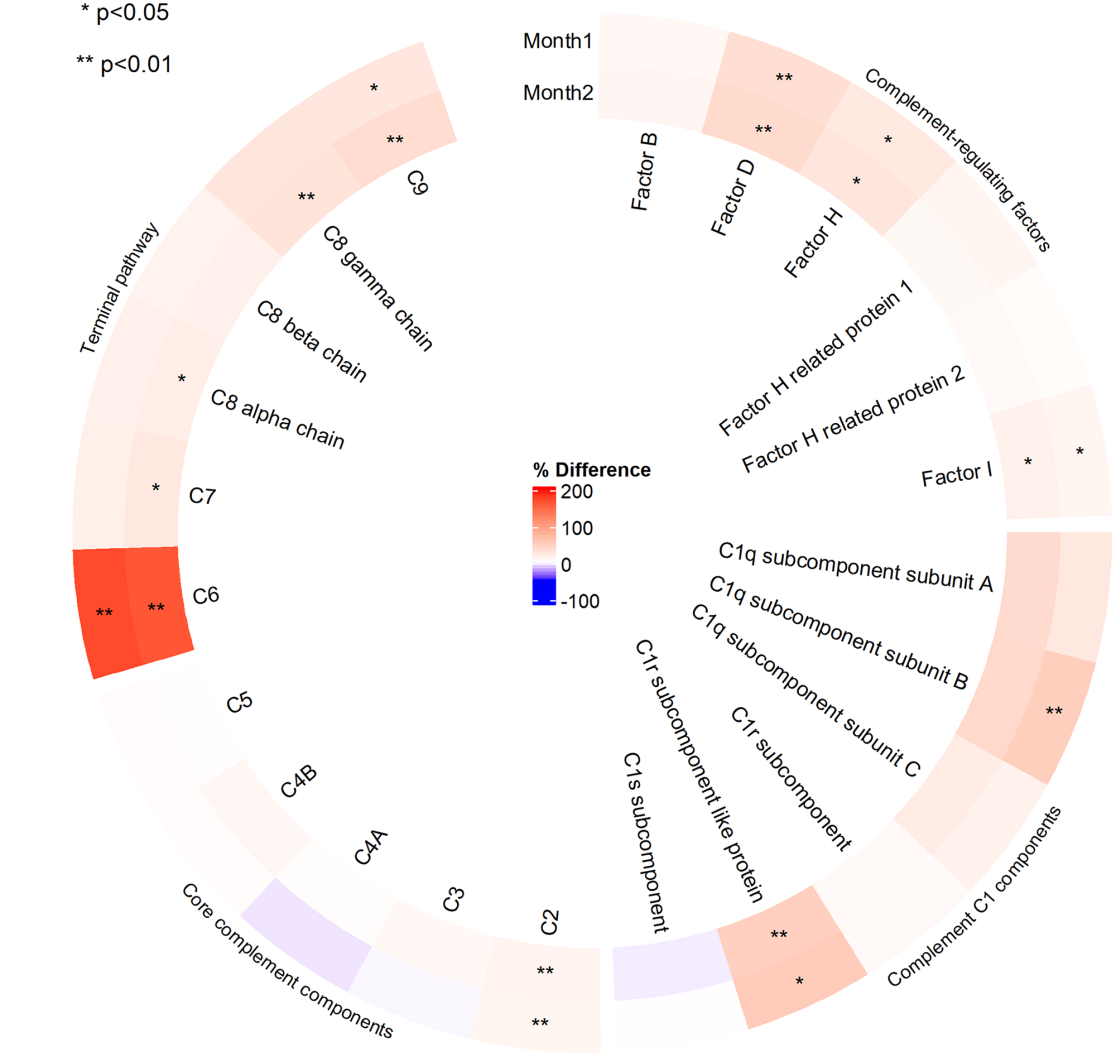
**Relative change in intensities of complement proteins after pegcetacoplan treatment.** The circular heatmap visualizes the changes in complement protein intensities in the aqueous humor 1 and 2 months after pegcetacoplan treatment. Intensity at baseline was used as reference for both comparisons. *P < 0.05, **P < 0.01.

### Effect of Pegcetacoplan on Other Proteins

**Figure 5.**
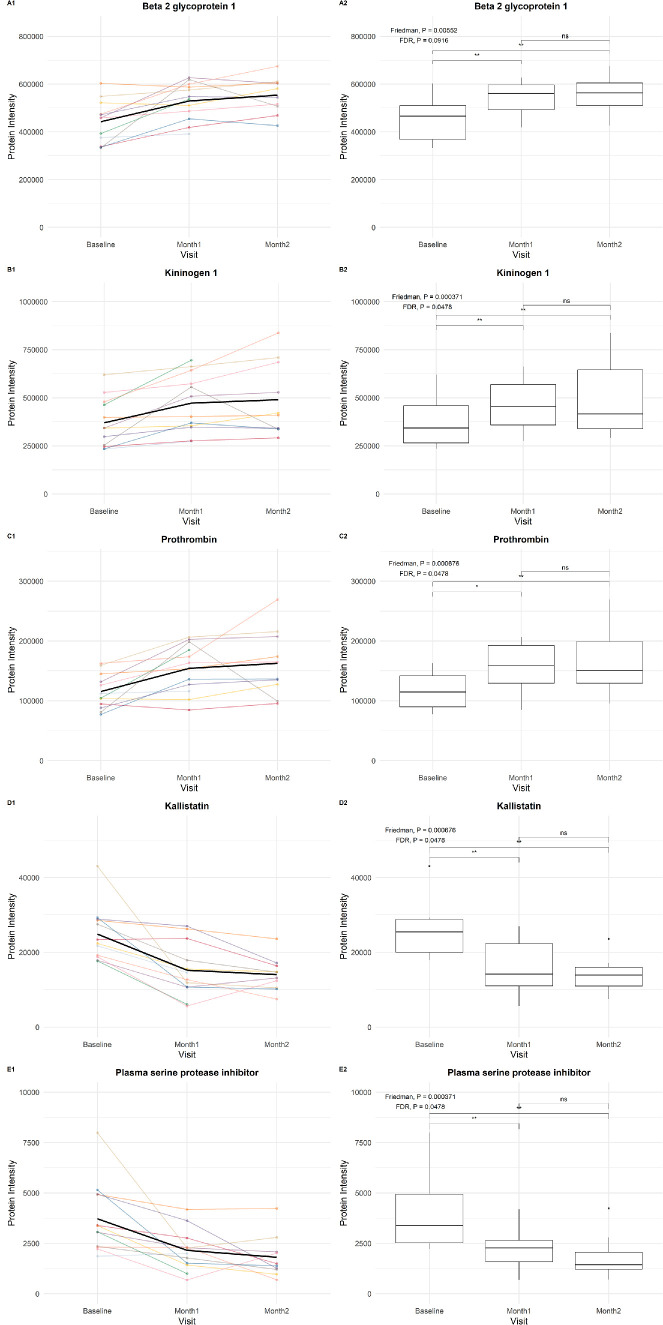
**Striking trends in non-complement proteins before and after monthly pegcetacoplan treatment.** (**A**) β2GPI, (**B**) KNG1, (**C**) prothrombin, (**D**) kallistatin, (**E**) plasma serine protease inhibitor. (**1**) Line plot illustrating individual patient trajectories of protein intensity over time. The *bold black line* indicates the mean protein intensity over time. (**2**) Box plots depicting the distribution of protein intensities at baseline, Month 1, and Month 2. Only AMD patients with measurements at all visits are included. The median, interquartile range, and outliers are displayed for each time point. *P < 0.05, **P < 0.01.

Most non-complement proteins showed no significant changes in intensities after treatment. We did observe significant changes for some proteins involved in the coagulation pathway. Specifically, intensities of beta-2–glycoprotein 1 (β2GPI; FDR < 0.10), kininogen 1 (KNG1; FDR < 0.05), and prothrombin (FDR < 0.05) were significantly higher after pegcetacoplan treatment, whereas the intensities of kallistatin (FDR < 0.05) and plasma serine protease inhibitor (FDR < 0.05) were significantly lower (Fig. 5). Box plots of these five protein intensities at baseline, Month 1, and Month 2, stratified on GRS (1–2, 2–3, >3), *CFH* Y402H variant (non-risk, heterozygous, homozygous), *C3* R102G variant (non-risk, heterozygous, homozygous), age, sex, and AMD status are displayed in Supplementary Figures S307 to S311. Similar to the complement proteins, no striking trends in subgroups were observed for these proteins. Notably, the trends in intensities of the coagulation-related proteins mentioned above were similar in both GA and GA+MNV patients.

### Effect of Pegcetacoplan Assessed on Pathway Level

**Figure 6.**
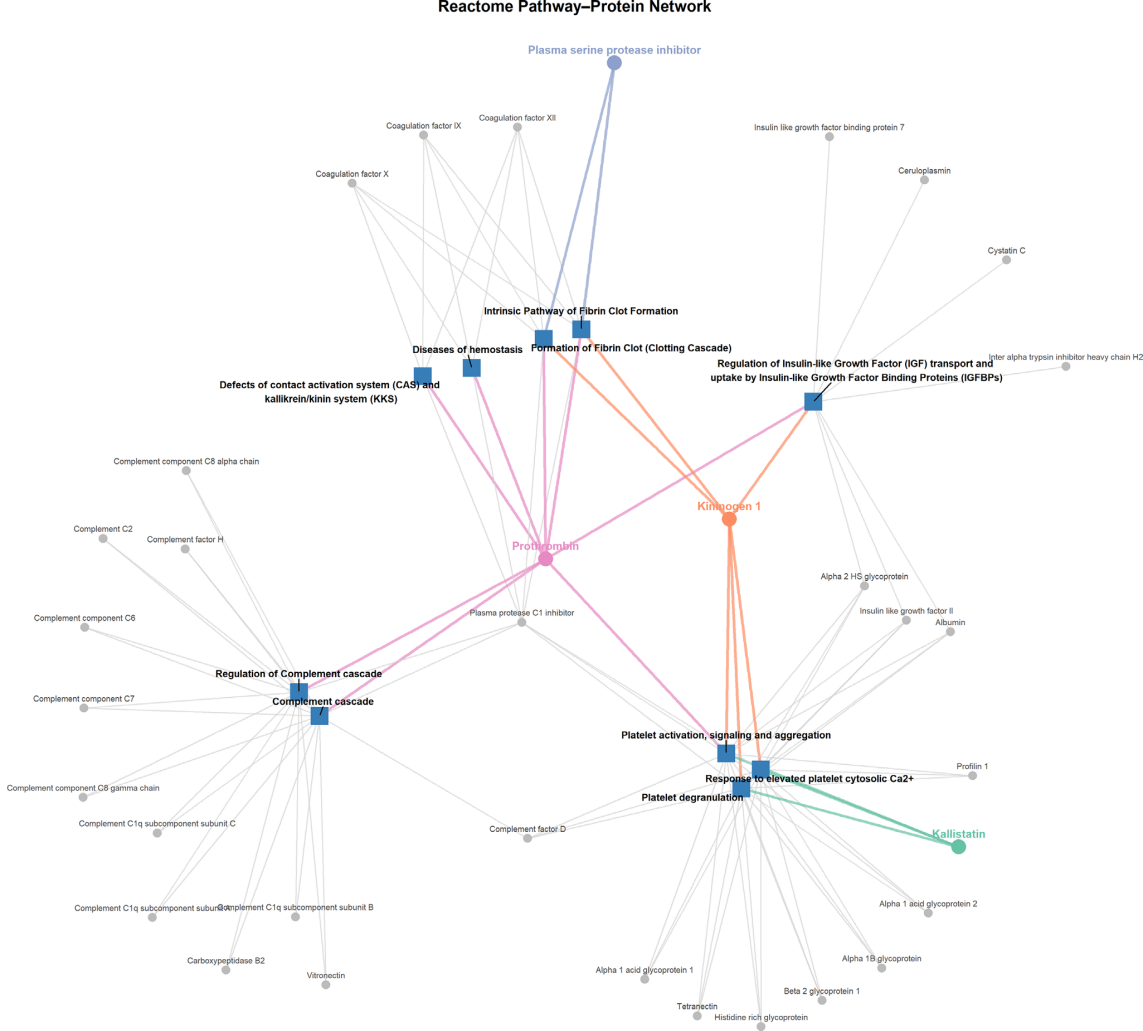
**Reactome pathway–protein interaction network.** Network visualization of the top 10 significantly enriched Reactome pathways (FDR < 0.05), based on proteins showing longitudinal changes after pegcetacoplan treatment (Friedman *P* < 0.05). Nodes represent pathways (*blue squares*) and proteins (*circles*). Significant proteins (Friedman *q* < 0.05) are shown in unique colors. The network illustrates shared membership across pathways and highlights proteins involved in multiple biological processes. All enriched pathways are displayed in Supplementary Table S1.

Reactome pathway enrichment revealed significant involvement of pathways related to complement activation, coagulation, platelet function, immune cell degranulation, and protein post-translational modifications (Supplementary Table S1). The top 10 pathways were visualized in a network showing their associated proteins (Fig. 6). The network highlights functional overlap across pathways and identifies central proteins linked to multiple processes.

## Discussion

Using mass spectrometry, this study explored whether pegcetacoplan treatment for GA affects the proteome in the AH of the eye. In our sample of 12 GA eyes, we did not observe changes in the levels of C3 and C5, the core proteins of the complement pathway. However, protein levels of C1qB, C1r subcomponent-like protein, C2, C6, C9, factor D, factor H, and factor I, increased at Month 1, with many of these elevations sustained through Month 2. We also found significant changes for proteins not directly involved in the complement system: significantly increased levels of β2GPI, KNG1, and prothrombin and significantly decreased levels of kallistatin and plasma serine protease inhibitor.

Before elaborating on our findings, it is important to acknowledge the strengths and limitations of this study. Key strengths include the innovative exploration of protein changes in the eye following treatment with complement inhibitor pegcetacoplan and the robust and reproducible protein analysis enabled by LC-MS. A limitation of the study is that protein analysis was performed on AH samples. VH sampling poses a risk in patients with retinal detachment or vitreous hemorrhage, but retinal detachment and vitreous hemorrhage are two example complications. These risks preclude routine VH sampling in clinical practice if there is not a strict medical indication. There is molecular exchange between the VH and AH through diffusion and convection currents, especially in soluble proteins, cytokines, and metabolites in older aged individuals. This means that retinal pathology can alter the biochemical composition not only of VH but also of AH.^31^^–^^33^ Although AH is not a perfect substitute for VH, as particularly large and insoluble molecules are likely to be underrepresented in AH, it is a practical source for studying complement factors (soluble) and complement activity in the eye of AMD patients.^34^^–^^36^ Moreover, Wilson et al.^37^ demonstrated moderate correlations for ∼60% of proteins between AH and VH, although correlations varied by protein. Importantly, their cohort did not include AMD patients, and key complement proteins such as C3 were not correlated. As a drug injected into the vitreous would only be found in very small fractions in the blood, and serum levels represent systemic metabolomics rather than intra-ocular kinetics, we did not consider the use of serum as an alternative accessible resource. Other limitations include the relatively short treatment period and the lack of power to relate protein levels to GA lesion size and progression. Aside from benefits, LC-MS has drawbacks, as well. This method can capture all ions within a mass range, but it cannot distinguish peptides of an intact protein from those of a cleaved protein when sequences are highly identical. Consequently, we were unable to differentiate protein levels between C3 and its fragments C3a and C3b, nor deC3d. The comparable levels of C3 observed before and during treatment could either indicate successful inhibition of cleavage, suggesting effective treatment or simply that cleaved forms C3a/C3b were still present post-treatment, which could indicate a lack of treatment efficacy.

The first prominent observation was the considerable interindividual variability in levels of complement and other proteins at baseline and during treatment. This variability may have obscured significant trends but is in itself a noteworthy finding that warrants further investigation. Such differences likely reflect each patient's biological and genetic profile, as well as the pharmacodynamics and bioavailability of the drug. Studies have shown that complement levels in blood can vary with age, sex, genetic profile, inflammatory status, and body mass index.^2^^,^^38^^–^^40^ Although for most proteins we did not find remarkable differences in protein levels when stratifying on the most established AMD determinants, it is conceivable that individual factors influence treatment response and trial outcomes.^13^^,^^15^

Given that C3 levels alone are inconclusive, factor B and downstream complement components such as C5 and C9 may act as surrogate markers to assess the impact of C3 inhibition. Blocking C3 cleavage is expected to reduce C3a and C3b generation, lower C3 convertase (C3bBb) levels, and decrease downstream complement activation. In our study, the levels of most complement proteins were higher after the start of treatment. Although we were not able to analyze markers for the activated form of complement, a marked decrease in downstream native components would have provided stronger evidence for successful inhibition of C3 cleavage.

An alternative explanation for the slightly elevated levels may be a compensatory feedback mechanism following complement inhibition. For example, in patients with paroxysmal nocturnal hemoglobinuria, C3 inhibition paradoxically led to increased C3 levels, likely due to reduced cleavage.^41^ Our observation of minimal increases in downstream complement protein levels during treatment may indicate that similar compensatory feedback mechanisms are occurring here. However, these increases varied considerably across patients, warranting caution in drawing conclusions about effective C3 blockade.

Another possibility is that complement dysregulation in AMD is not solely local. If systemic complement activation is high, circulating complement proteins may enter the eye and limit the impact of intravitreal inhibition. This could explain the lack of reduction in C3 or C5 levels in the AH despite direct treatment as observed in our study. From this perspective, combining systemic and local complement inhibition may offer a more effective strategy: By lowering systemic complement levels, less complement would enter the eye, potentially allowing local therapy to act more efficiently on intraocular complement production. This raises the possibility that intravitreal complement inhibitors might achieve greater biological effect when paired with systemic modulation—reducing the overall complement burden within the eye and enabling better control of locally driven complement activity. We recognize, however, that these interpretations remain speculative, as they are based solely on AH measurements. Alternative explanations, including injection-related inflammation or natural longitudinal changes, independent of treatment, in complement proteins may also contribute to the observed findings. To strengthen the biological relevance of these results, future studies should validate the proteomics signatures in other ocular fluids and confirm them using complementary analytical techniques. Additional work including appropriately matched healthy controls, untreated GA patients, and extended follow-up will also be important to delineate these mechanisms.

What can be learned from proteins that are not direct components of the complement cascade? The proteins that were significantly changed in level after treatment appear to be remarkably interconnected. β2GPI is a multifunctional glycoprotein with known roles in blood coagulation, inflammation, and anti-phospholipid syndrome. The current notion is that β2GPI can help regulate complement, as it can bind to C3 and facilitate subsequent binding of CFH or even allow C3 cleavage by CFI in the absence of CFH.^42^ In addition, interaction between mannose-binding lectin and β2GPI bound to endothelial cells promotes complement activation and thrombin formation.^43^ Interestingly, β2GPI has been detected in the retina and choroid of AMD patients.^44^ KNG1 is a glycoprotein involved in the kinin–kallikrein system. A role for this system has been reported in ocular vascular diseases including AMD,^45^ and is associated with inflammation and pathological neovascularization.^46^ As high-molecular-weight kininogen (HK), it acts as a cofactor of the intrinsic coagulation pathway; as low-molecular-weight kininogen (LK), it is more involved in tissue protease inhibition and inflammation. It interacts with the complement cascade through the lectin pathway, where it is cleaved by mannose-binding lectin-associated serine protease 1 (MASP-1), leading to the release of the vasodilator bradykinin, a peptide that increases vascular permeability and promotes inflammation.^47^

Kallistatin is a serine proteinase inhibitor that inhibits kallikrein. It exerts its anti-inflammatory effects by inhibiting TNF-α, IL-6, and nuclear factor kappa B (NF-κB) and its effects on angiogenesis by inhibiting VEGF.^48^ By interfering with kallikrein, kallistatin prevents cleavage of KNG1, which reduces the release of bradykinin. Although kallistatin does not directly interact with complement, it relates to it by the mediators MASP-1 and factor XIIa. Prothrombin (coagulation factor II), the inactive precursor of thrombin, is a key enzyme in the coagulation cascade and also interacts with the complement system. Prothrombin can also be cleaved by MASP-1 of the lectin pathway, resulting in coagulation.^49^ Finally, serine protease inhibitors (serpins), a family of proteins that includes kallistatin, C1 inhibitor (C1-INH), and antithrombin, play critical roles in inhibiting proteases involved in inflammation, immunoregulation, and coagulation. C1-INH inhibits C1r and C1s in the classical pathway and MASP-1 and MASP-2 in the lectin pathway,^50^ and antithrombin serves as an anticoagulant by inhibiting thrombin.^51^

The protein functions described above question whether the observed changes in levels following treatment would be beneficial for AMD. An increase in β2GPI could help regulate complement but may also increase activation when anti-β2GPI antibodies are present. Increased levels of KNG1 suggest a more active kinin–kallikrein system and more bradykinin, which promotes neovascularization. Higher prothrombin levels might promote clotting in choroidal capillaries. Decreased kallistatin levels reduce VEGF inhibition. These changes could explain the increase in subretinal neovascularization observed in complement targeting trials.^13^^,^^52^ A decrease in C1-INH is particularly ominous. With reduced inhibition of C1r and C1s, C1q can bind to pathogens or antibodies and activate the classical complement pathway. The elevated levels of C1r subcomponent-like protein, C1qB, and C2 that were found in this study align with this notion.

In conclusion, our exploratory analyses of the AH proteome in GA patients prior to and during pegcetacoplan treatment provide new leads that warrant further investigation. In this limited cohort, levels of C3 and C5 remained stable during treatment, but five proteins related to complement, inflammation, and coagulation showed significant changes. These findings should be validated to clarify their relevance. Upon validation, the observed changes may help elucidate how complement inhibition at the level of C3 influences other biological systems. Despite its limitations, this study offers novel insights into the ocular effects of complement inhibition and may support the design of biomarker-driven therapeutic research in AMD.

## Supplementary Material

Supplement 1
