# Supplementary material for: Effect of Pegcetacoplan on Aqueous Humor Proteome in Geographic Atrophy: A Prospective Exploration
Source: Invest Ophthalmol Vis Sci. 2025 Dec 5;66(15):24. doi: 10.1167/iovs.66.15.24 (PMC12700176; doi:10.1167/iovs.66.15.24)
Supplement: Supplement 1 [file iovs-66-15-24_s001.pdf]

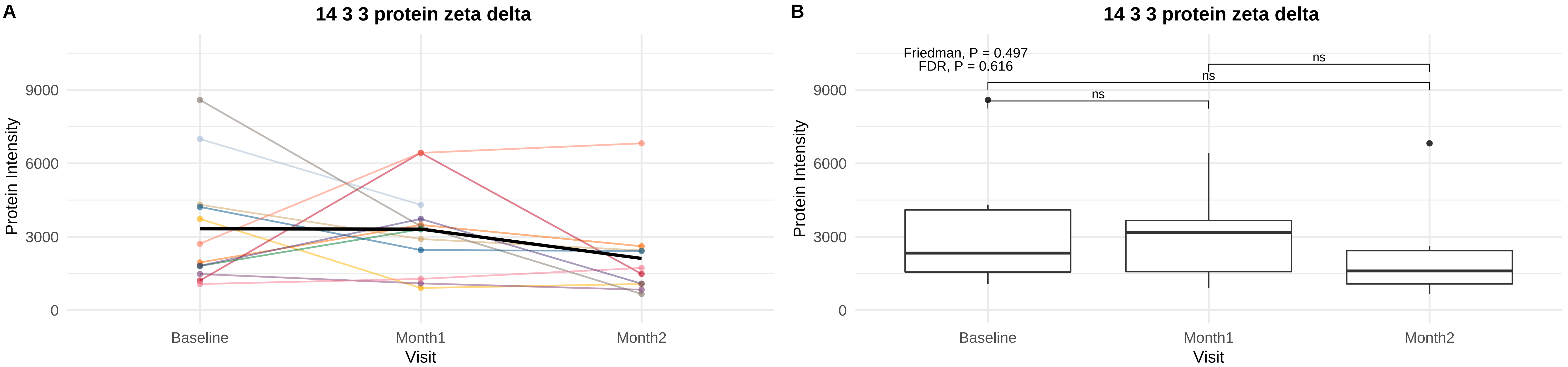

Supplementary Figure S 1

A) Line plot illustrating individual patient trajectories of 14 3 3 protein zeta delta intensity over time. The bold black line indicates the mean intensity over time. B) Box plots depicting the distribution of 14 3 3 protein zeta delta intensities at baseline, month 1, and month 2. Only AMD patients with measurements at all visits are included. The median, interquartile range, and outliers are displayed for each time point. Abbreviations: FDR, false discovery rate; ns, non−significant; * p < 0.05; ** p < 0.01; *** p < 0.001.

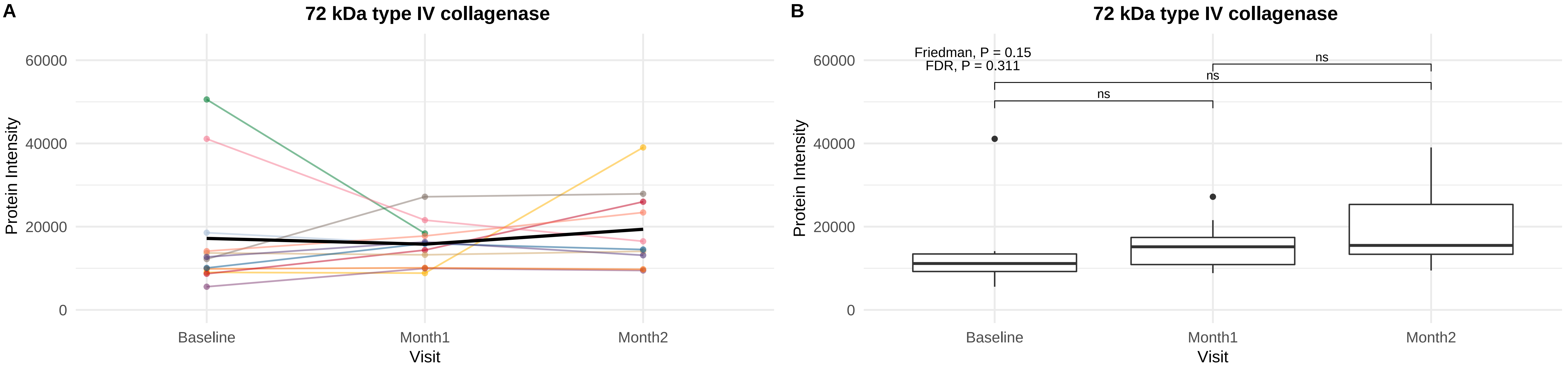

Supplementary Figure S 2

A) Line plot illustrating individual patient trajectories of 72 kDa type IV collagenase intensity over time. The bold black line indicates the mean intensity over time. B) Box plots depicting the distribution of 72 kDa type IV collagenase intensities at baseline, month 1, and month 2. Only AMD patients with measurements at all visits are included. The median, interquartile range, and outliers are displayed for each time point. Abbreviations: FDR, false discovery rate; ns, non−significant; * p < 0.05; ** p < 0.01; *** p < 0.001.

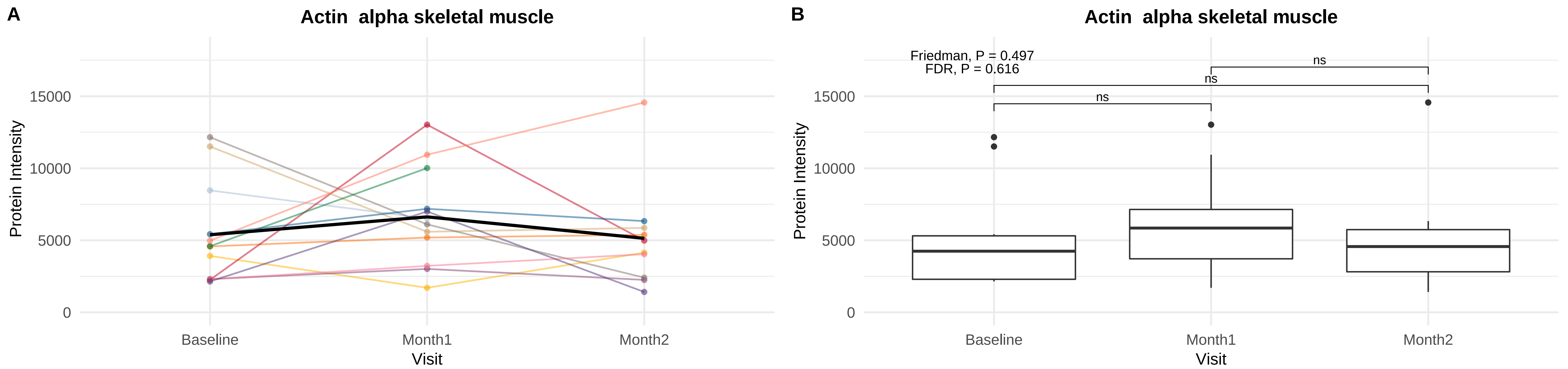

Supplementary Figure S 3

A) Line plot illustrating individual patient trajectories of Actin alpha skeletal muscle intensity over time. The bold black line indicates the mean intensity over time. B) Box plots depicting the distribution of Actin alpha skeletal muscle intensities at baseline, month 1, and month 2. Only AMD patients with measurements at all visits are included. The median, interquartile range, and outliers are displayed for each time point. Abbreviations: FDR, false discovery rate; ns, non−significant; * p < 0.05; ** p < 0.01; *** p < 0.001.

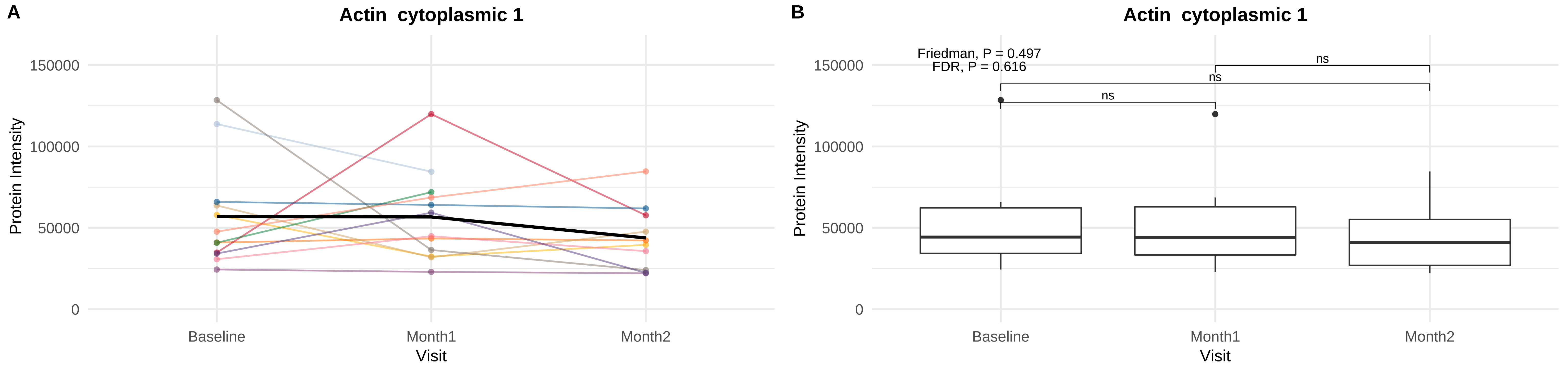

Supplementary Figure S 4

A) Line plot illustrating individual patient trajectories of Actin cytoplasmic 1 intensity over time. The bold black line indicates the mean intensity over time. B) Box plots depicting the distribution of Actin cytoplasmic 1 intensities at baseline, month 1, and month 2. Only AMD patients with measurements at all visits are included. The median, interquartile range, and outliers are displayed for each time point. Abbreviations: FDR, false discovery rate; ns, non−significant; * p < 0.05; ** p < 0.01; *** p < 0.001.

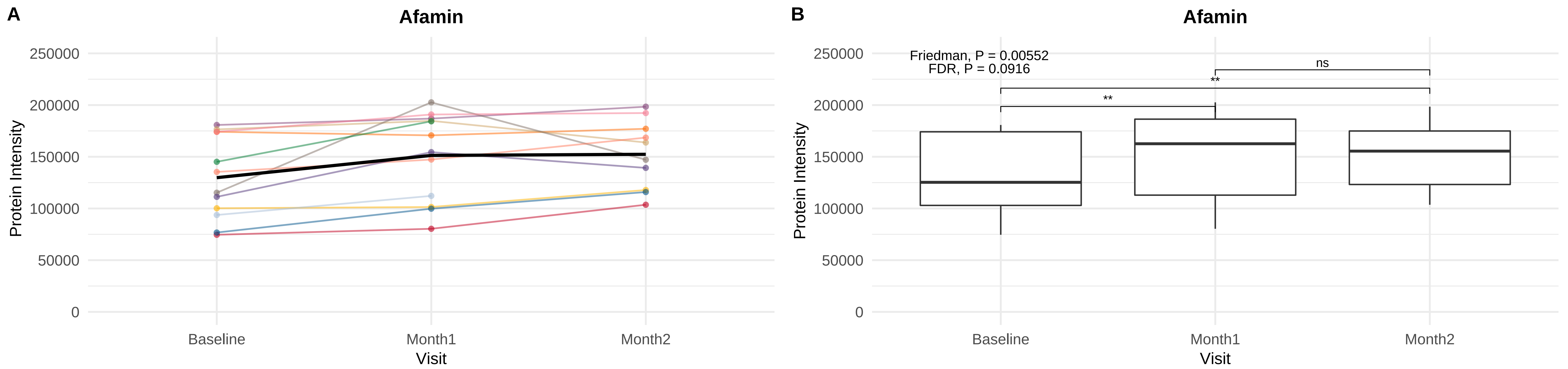

Supplementary Figure S 5

A) Line plot illustrating individual patient trajectories of Afamin intensity over time. The bold black line indicates the mean intensity over time. B) Box plots depicting the distribution of Afamin intensities at baseline, month 1, and month 2. Only AMD patients with measurements at all visits are included. The median, interquartile range, and outliers are displayed for each time point. Abbreviations: FDR, false discovery rate; ns, non−significant; * p < 0.05; ** p < 0.01; *** p < 0.001.

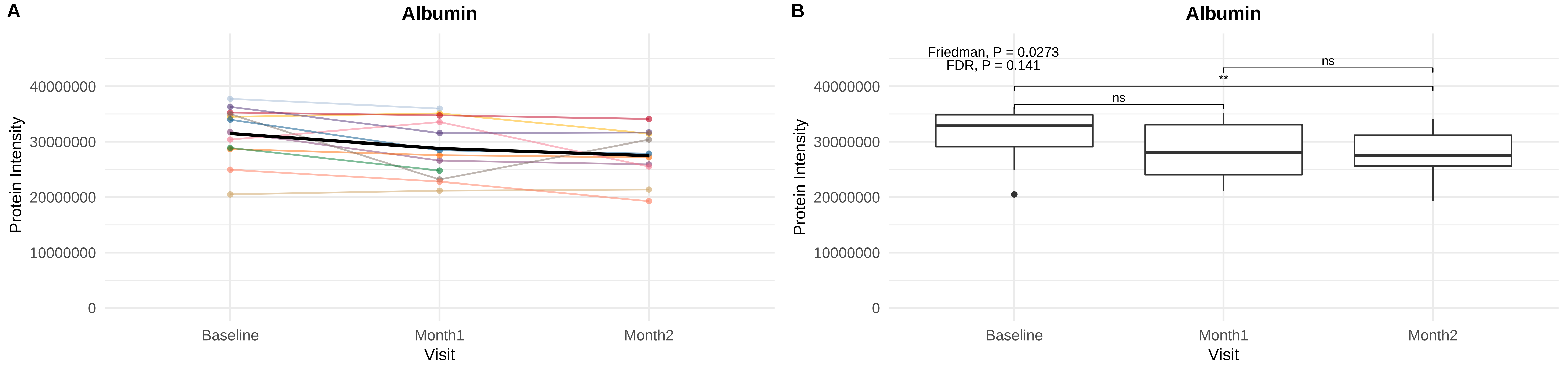

Supplementary Figure S 6

A) Line plot illustrating individual patient trajectories of Albumin intensity over time. The bold black line indicates the mean intensity over time. B) Box plots depicting the distribution of Albumin intensities at baseline, month 1, and month 2. Only AMD patients with measurements at all visits are included. The median, interquartile range, and outliers are displayed for each time point. Abbreviations: FDR, false discovery rate; ns, non−significant; * p < 0.05; ** p < 0.01; *** p < 0.001.

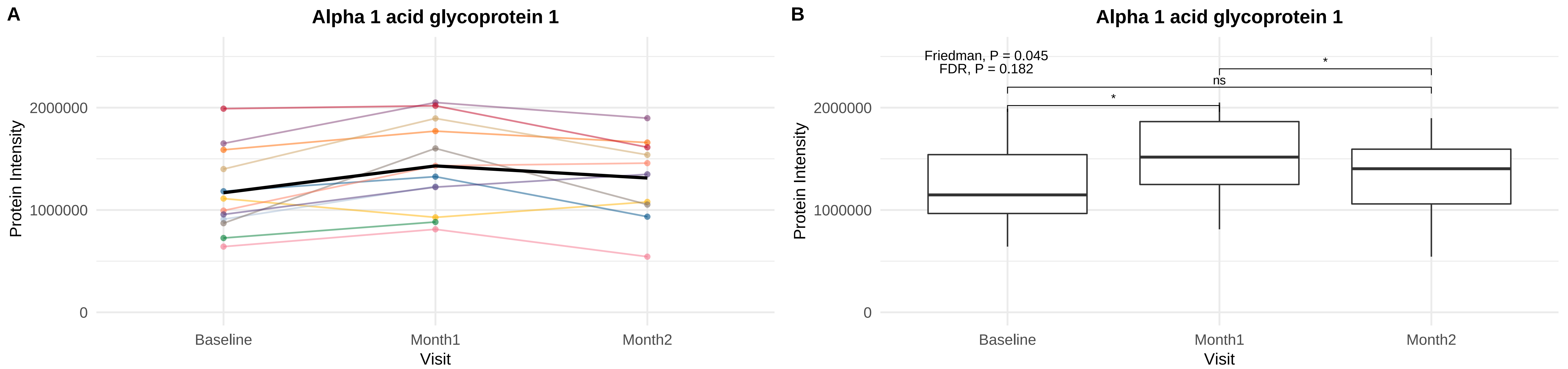

Supplementary Figure S 7

A) Line plot illustrating individual patient trajectories of Alpha 1 acid glycoprotein 1 intensity over time. The bold black line indicates the mean intensity over time. B) Box plots depicting the distribution of Alpha 1 acid glycoprotein 1 intensities at baseline, month 1, and month 2. Only AMD patients with measurements at all visits are included. The median, interquartile range, and outliers are displayed for each time point. Abbreviations: FDR, false discovery rate; ns, non−significant; * p < 0.05; ** p < 0.01; *** p < 0.001.

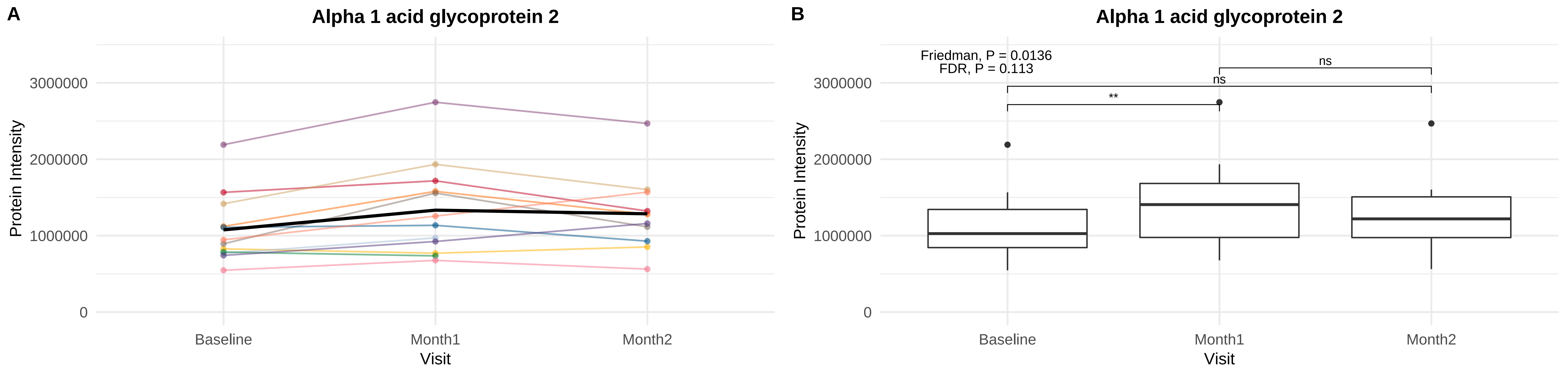

Supplementary Figure S 8

A) Line plot illustrating individual patient trajectories of Alpha 1 acid glycoprotein 2 intensity over time. The bold black line indicates the mean intensity over time. B) Box plots depicting the distribution of Alpha 1 acid glycoprotein 2 intensities at baseline, month 1, and month 2. Only AMD patients with measurements at all visits are included. The median, interquartile range, and outliers are displayed for each time point. Abbreviations: FDR, false discovery rate; ns, non−significant; * p < 0.05; ** p < 0.01; *** p < 0.001.

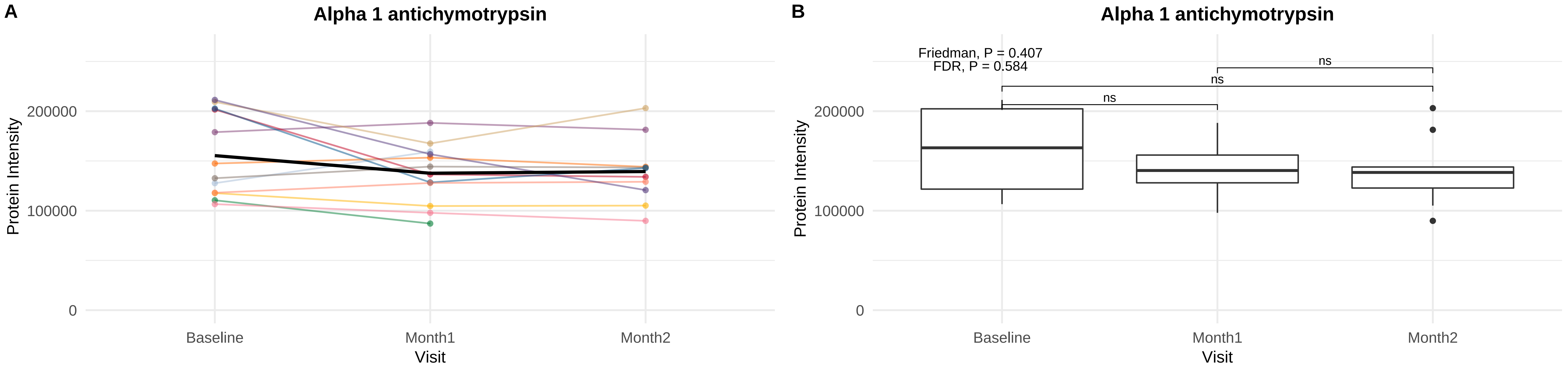

Supplementary Figure S 9

A) Line plot illustrating individual patient trajectories of Alpha 1 antichymotrypsin intensity over time. The bold black line indicates the mean intensity over time. B) Box plots depicting the distribution of Alpha 1 antichymotrypsin intensities at baseline, month 1, and month 2. Only AMD patients with measurements at all visits are included. The median, interquartile range, and outliers are displayed for each time point. Abbreviations: FDR, false discovery rate; ns, non−significant; * p < 0.05; ** p < 0.01; *** p < 0.001.

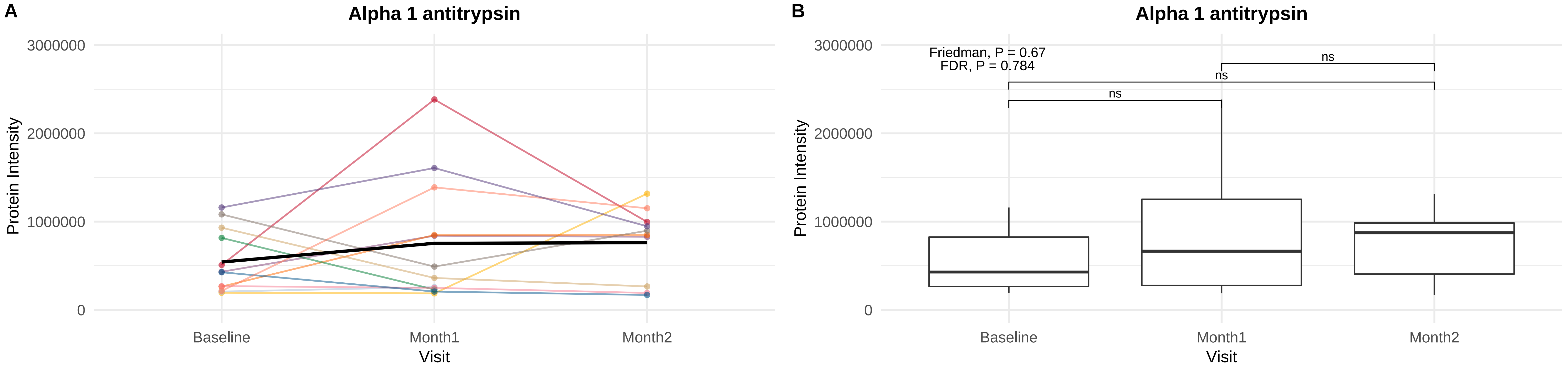

Supplementary Figure S 10

A) Line plot illustrating individual patient trajectories of Alpha 1 antitrypsin intensity over time. The bold black line indicates the mean intensity over time. B) Box plots depicting the distribution of Alpha 1 antitrypsin intensities at baseline, month 1, and month 2. Only AMD patients with measurements at all visits are included. The median, interquartile range, and outliers are displayed for each time point. Abbreviations: FDR, false discovery rate; ns, non−significant; * p < 0.05; ** p < 0.01; *** p < 0.001.

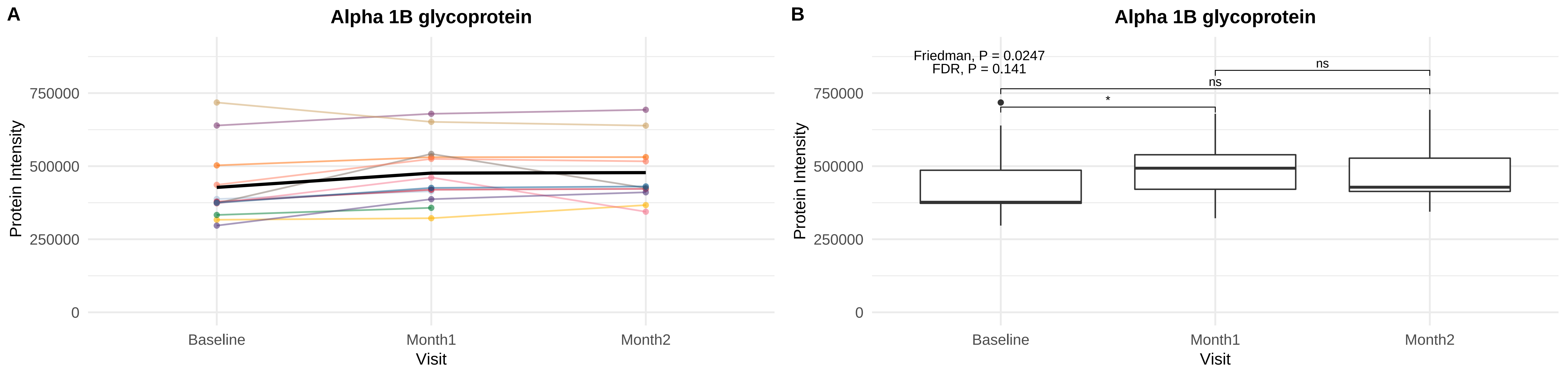

Supplementary Figure S 11

A) Line plot illustrating individual patient trajectories of Alpha 1B glycoprotein intensity over time. The bold black line indicates the mean intensity over time. B) Box plots depicting the distribution of Alpha 1B glycoprotein intensities at baseline, month 1, and month 2. Only AMD patients with measurements at all visits are included. The median, interquartile range, and outliers are displayed for each time point. Abbreviations: FDR, false discovery rate; ns, non−significant; * p < 0.05; ** p < 0.01; *** p < 0.001.

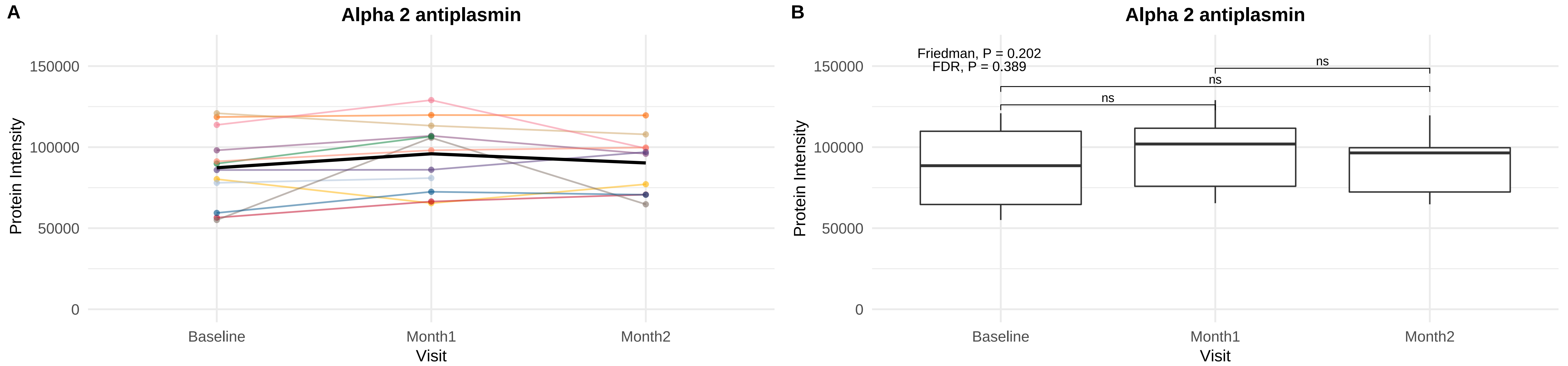

Supplementary Figure S 12

A) Line plot illustrating individual patient trajectories of Alpha 2 antiplasmin intensity over time. The bold black line indicates the mean intensity over time. B) Box plots depicting the distribution of Alpha 2 antiplasmin intensities at baseline, month 1, and month 2. Only AMD patients with measurements at all visits are included. The median, interquartile range, and outliers are displayed for each time point. Abbreviations: FDR, false discovery rate; ns, non−significant; * p < 0.05; ** p < 0.01; *** p < 0.001.

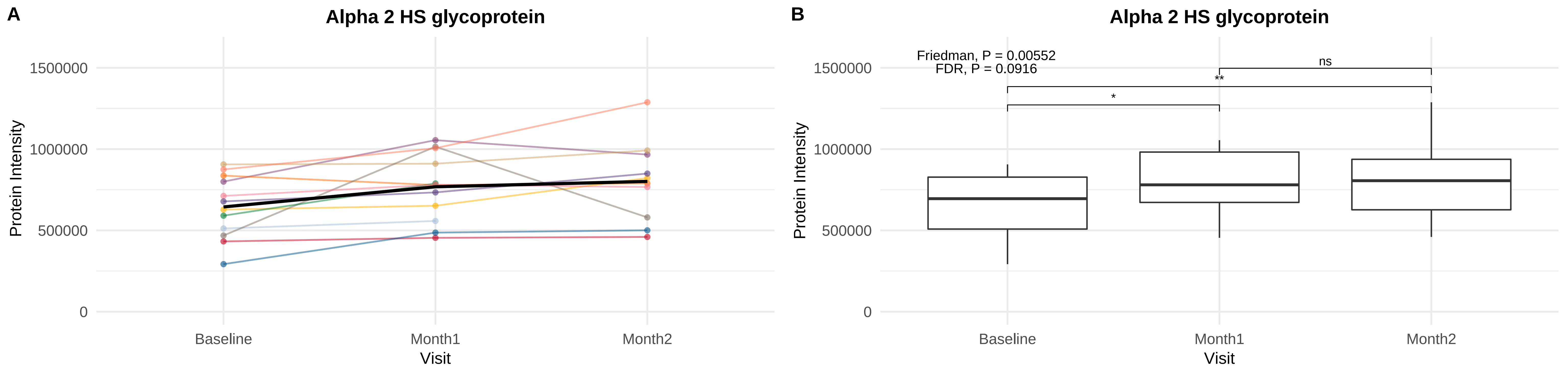

Supplementary Figure S 13

A) Line plot illustrating individual patient trajectories of Alpha 2 HS glycoprotein intensity over time. The bold black line indicates the mean intensity over time. B) Box plots depicting the distribution of Alpha 2 HS glycoprotein intensities at baseline, month 1, and month 2. Only AMD patients with measurements at all visits are included. The median, interquartile range, and outliers are displayed for each time point. Abbreviations: FDR, false discovery rate; ns, non−significant; * p < 0.05; ** p < 0.01; *** p < 0.001.

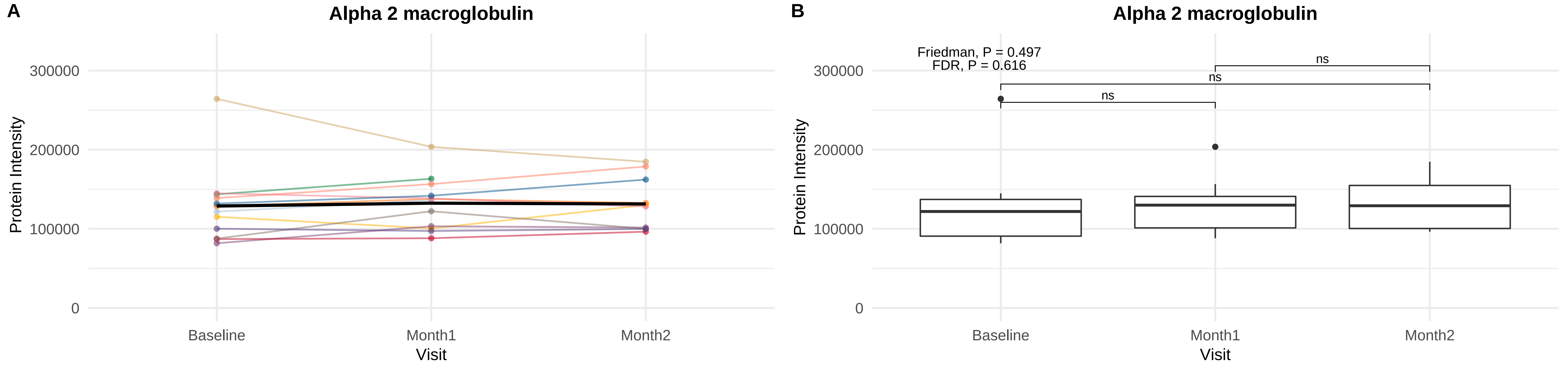

Supplementary Figure S 14

A) Line plot illustrating individual patient trajectories of Alpha 2 macroglobulin intensity over time. The bold black line indicates the mean intensity over time. B) Box plots depicting the distribution of Alpha 2 macroglobulin intensities at baseline, month 1, and month 2. Only AMD patients with measurements at all visits are included. The median, interquartile range, and outliers are displayed for each time point. Abbreviations: FDR, false discovery rate; ns, non−significant; * p < 0.05; ** p < 0.01; *** p < 0.001.

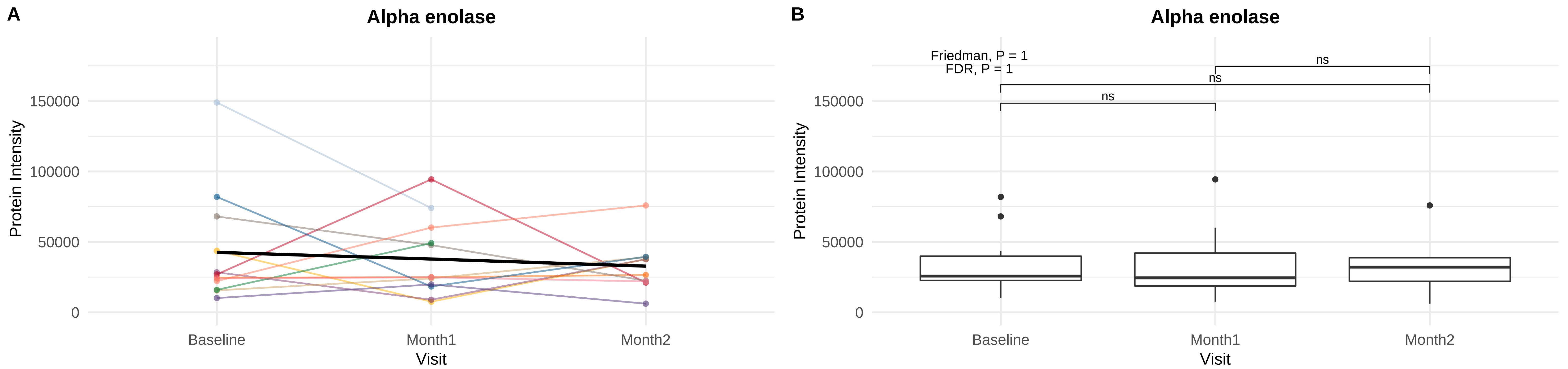

Supplementary Figure S 15

A) Line plot illustrating individual patient trajectories of Alpha enolase intensity over time. The bold black line indicates the mean intensity over time. B) Box plots depicting the distribution of Alpha enolase intensities at baseline, month 1, and month 2. Only AMD patients with measurement at all visits are included. The median, interquartile range, and outliers are displayed for each time point. Abbreviations: FDR, false discovery rate; ns, non−significant; * p < 0.05; ** p < 0.01; *** p < 0.001.

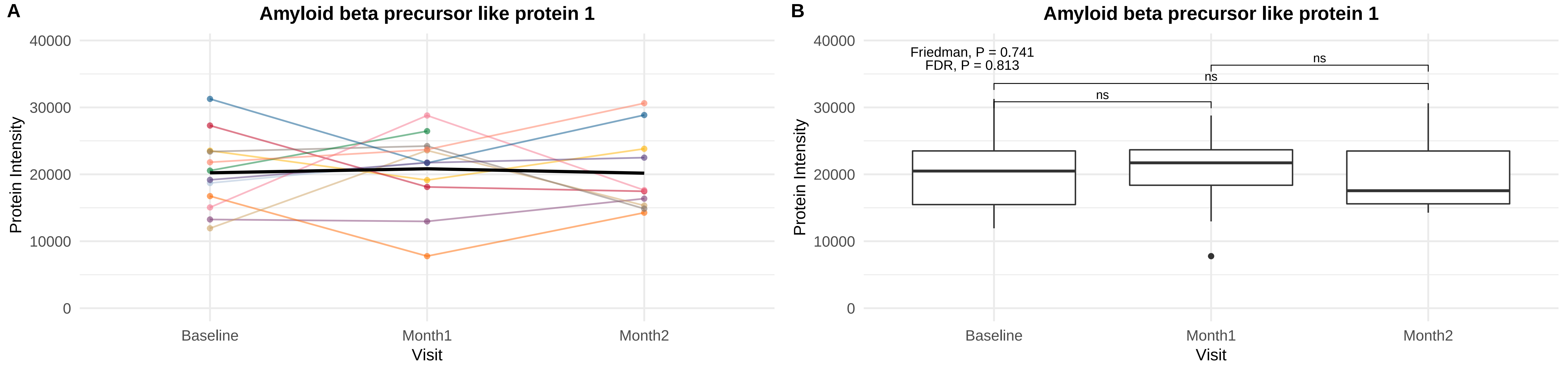

Supplementary Figure S 16

A) Line plot illustrating individual patient trajectories of Amyloid beta precursor like protein 1 intensity over time. The bold black line indicates the mean intensity over time. B) Box plots depicting the distribution of Amyloid beta precursor like protein 1 intensities at baseline, month 1, and month 2. Only AMD patients with measurements at all visits are included. The median, interquartile range, and outliers are displayed for each time point. Abbreviations: FDR, false discovery rate; ns, non−significant; * p < 0.05; ** p < 0.01; *** p < 0.001.

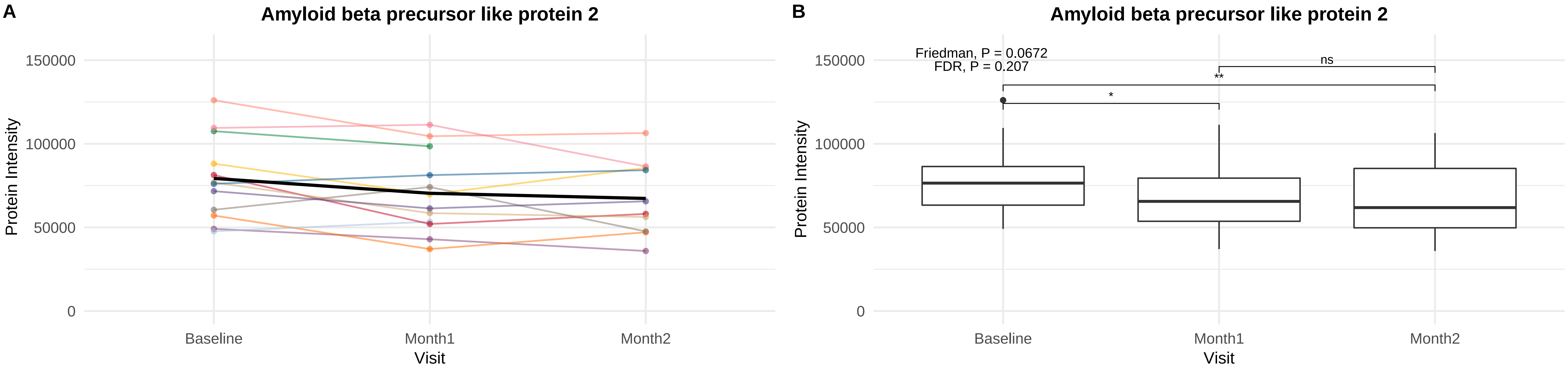

Supplementary Figure S 17

A) Line plot illustrating individual patient trajectories of Amyloid beta precursor like protein 2 intensity over time. The bold black line indicates the mean intensity over time. B) Box plots depicting the distribution of Amyloid beta precursor like protein 2 intensities at baseline, month 1, and month 2. Only AMD patients with measurements at all visits are included. The median, interquartile range, and outliers are displayed for each time point. Abbreviations: FDR, false discovery rate; ns, non−significant; * p < 0.05; ** p < 0.01; *** p < 0.001.

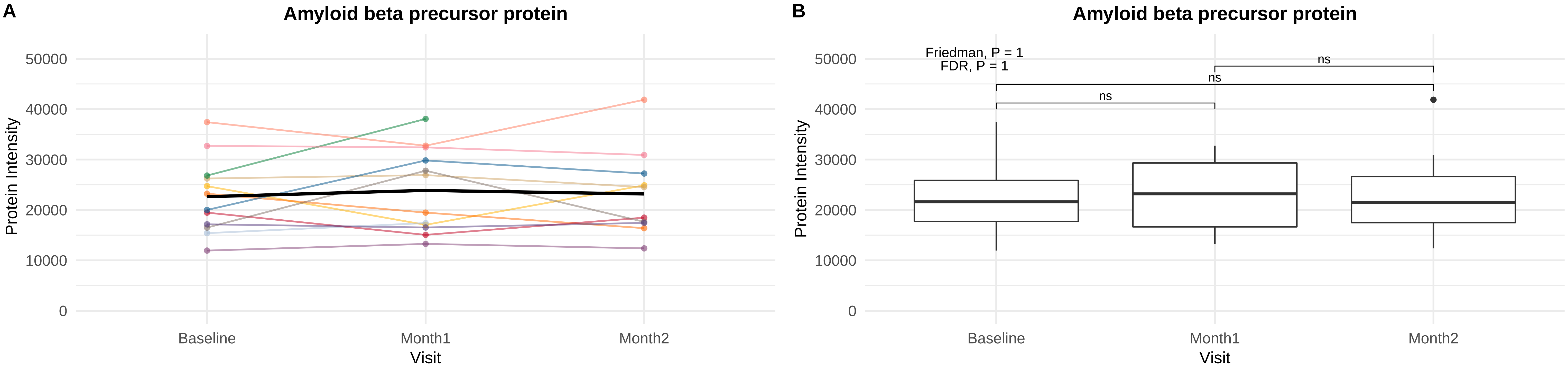

Supplementary Figure S 18

A) Line plot illustrating individual patient trajectories of Amyloid beta precursor protein intensity over time. The bold black line indicates the mean intensity over time. B) Box plots depicting the distribution of Amyloid beta precursor protein intensities at baseline, month 1, and month 2. Only AMD patients with measurements at all visits are included. The median, interquartile range, and outliers are displayed for each time point. Abbreviations: FDR, false discovery rate; ns, non−significant; * p < 0.05; ** p < 0.01; *** p < 0.001.

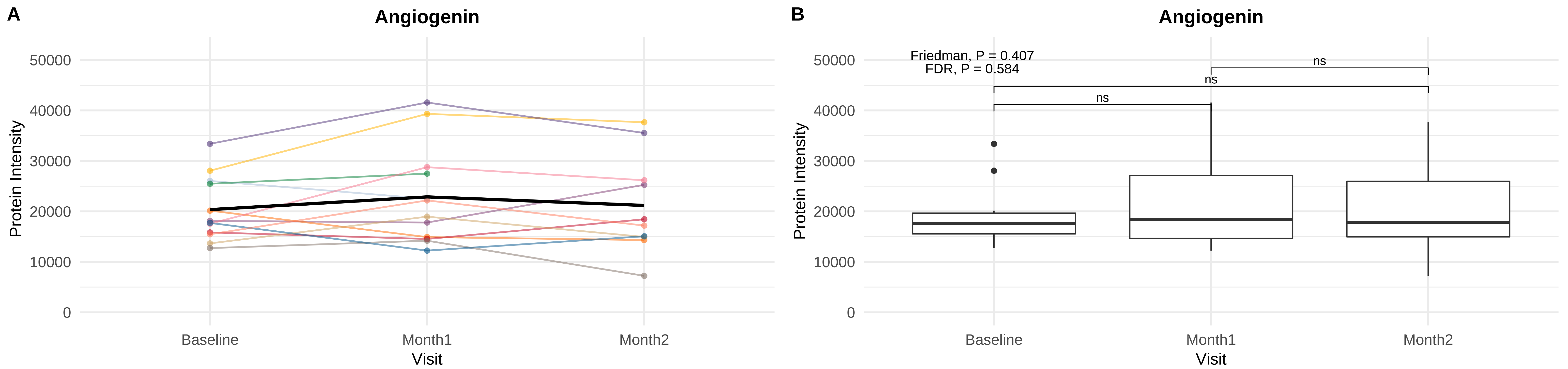

Supplementary Figure S 19

A) Line plot illustrating individual patient trajectories of Angiogenin intensity over time. The bold black line indicates the mean intensity over time. B) Box plots depicting the distribution of Angiogenin intensities at baseline, month 1, and month 2. Only AMD patients with measurements at all visits are included. The median, interquartile range, and outliers are displayed for each time point. Abbreviations: FDR, false discovery rate; ns, non−significant; * p < 0.05; ** p < 0.01; *** p < 0.001.

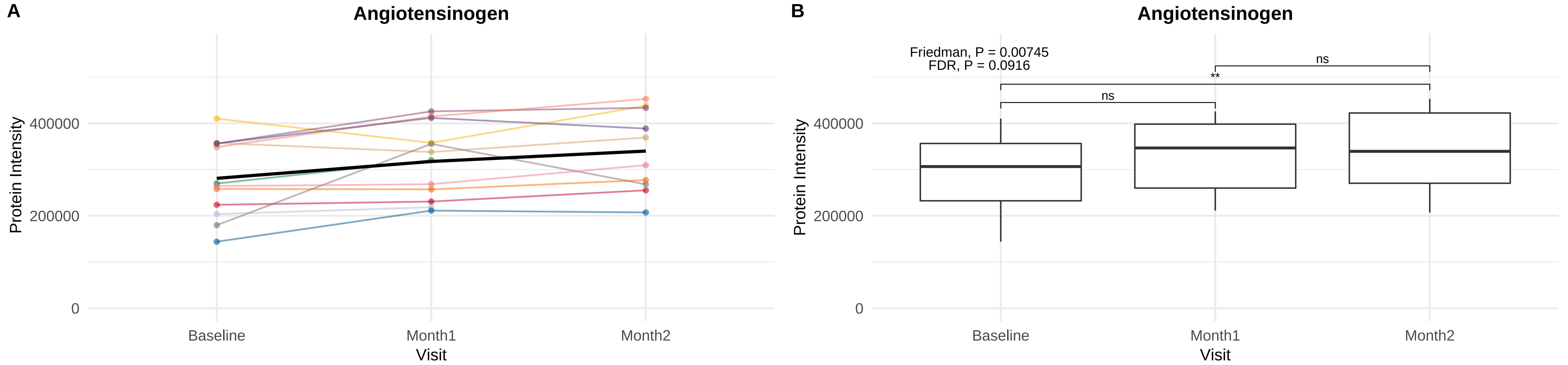

Supplementary Figure S 20

A) Line plot illustrating individual patient trajectories of Angiotensinogen intensity over time. The bold black line indicates the mean intensity over time. B) Box plots depicting the distribution of Angiotensinogen intensities at baseline, month 1, and month 2. Only AMD patients with measurements at all visits are included. The median, interquartile range, and outliers are displayed for each time point. Abbreviations: FDR, false discovery rate; ns, non−significant; * p < 0.05; ** p < 0.01; *** p < 0.001.

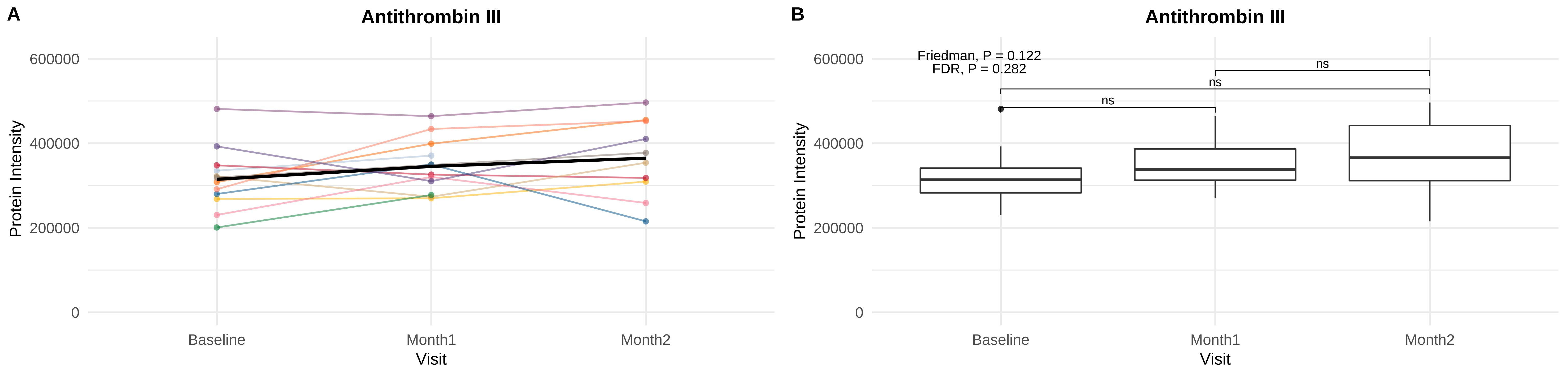

Supplementary Figure S 21

A) Line plot illustrating individual patient trajectories of Antithrombin III intensity over time. The bold black line indicates the mean intensity over time. B) Box plots depicting the distribution of Antithrombin III intensities at baseline, month 1, and month 2. Only AMD patients with measurements at all visits are included. The median, interquartile range, and outliers are displayed for each time point. Abbreviations: FDR, false discovery rate; ns, non−significant; * p < 0.05; ** p < 0.01; *** p < 0.001.

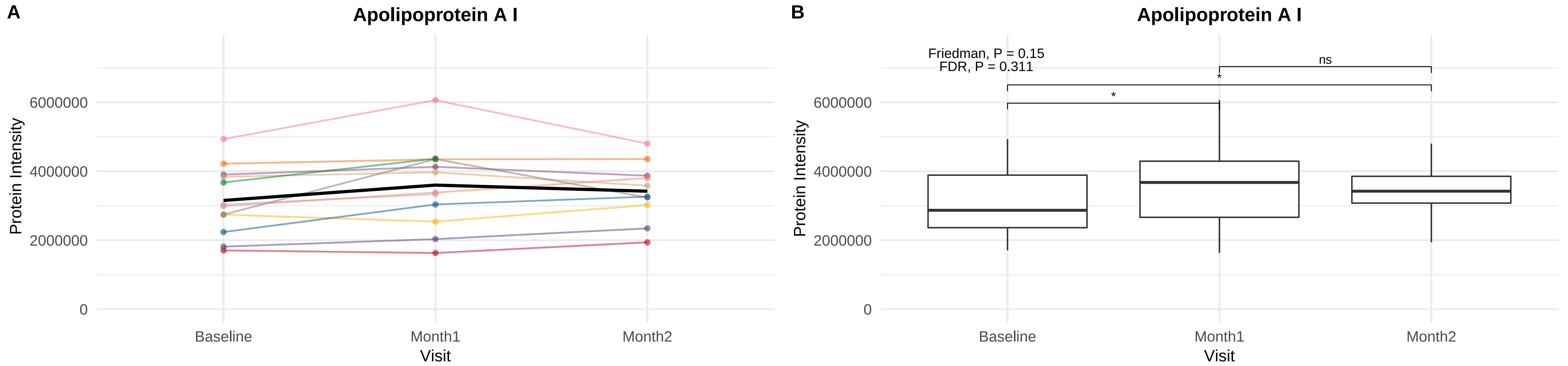

Supplementary Figure S 22

A) Line plot illustrating individual patient trajectories of Apolipoprotein A I intensity over time. The bold black line indicates the mean intensity over time. B) Box plots depicting the distribution of Apolipoprotein A I intensities at baseline, month 1, and month 2. Only AMD patients with measurements at all visits are included. The median, interquartile range, and outliers are displayed for each time point. Abbreviations: FDR, false discovery rate; ns, non−significant; * p < 0.05; ** p < 0.01; *** p < 0.001.

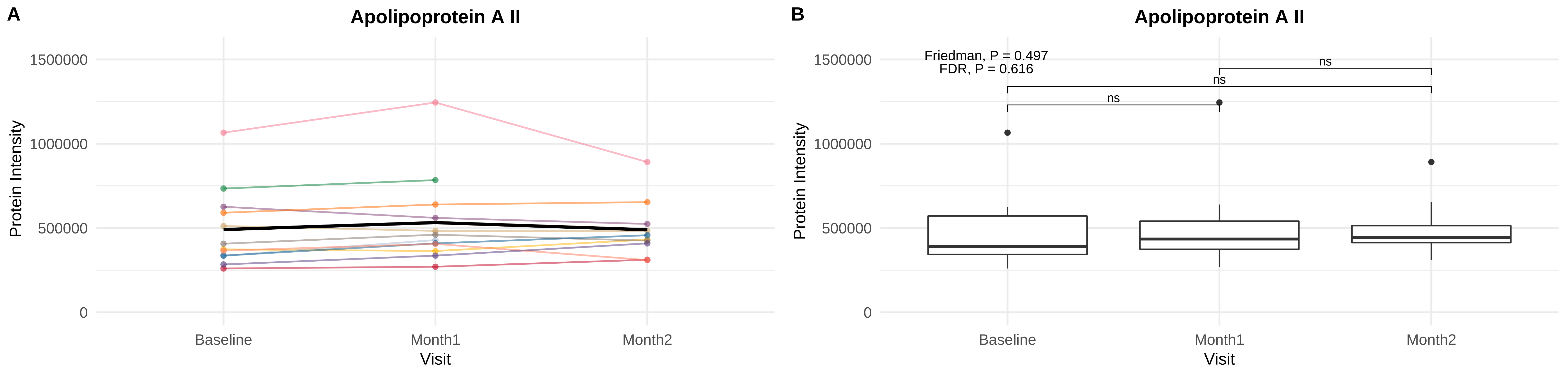

Supplementary Figure S 23

A) Line plot illustrating individual patient trajectories of Apolipoprotein A II intensity over time. The bold black line indicates the mean intensity over time. B) Box plots depicting the distribution of Apolipoprotein A II intensities at baseline, month 1, and month 2. Only AMD patients with measurements at all visits are included. The median, interquartile range, and outliers are displayed for each time point. Abbreviations: FDR, false discovery rate; ns, non−significant; * p < 0.05; ** p < 0.01; *** p < 0.001.

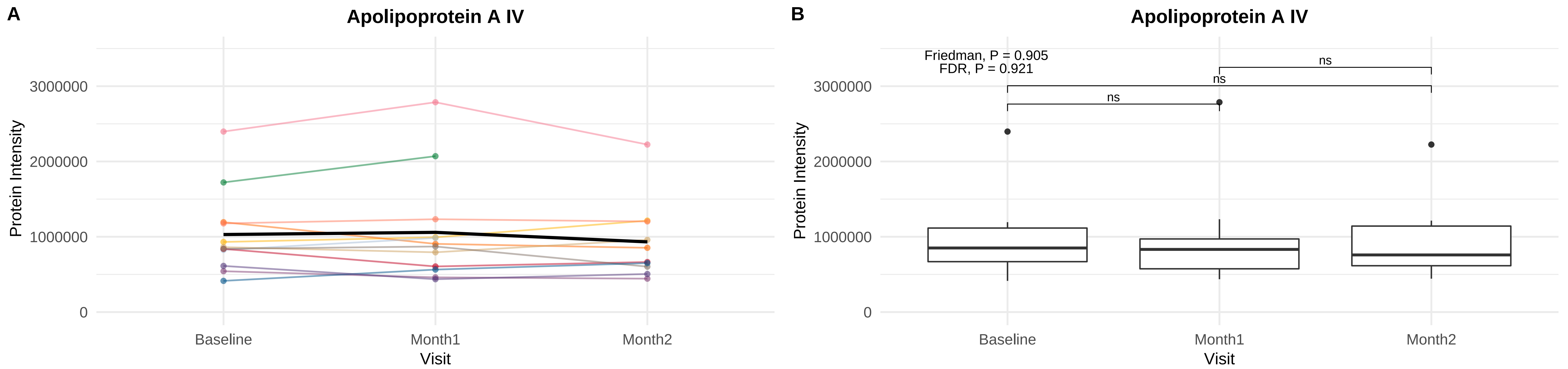

Supplementary Figure S 24

A) Line plot illustrating individual patient trajectories of Apolipoprotein A IV intensity over time. The bold black line indicates the mean intensity over time. B) Box plots depicting the distribution of Apolipoprotein A IV intensities at baseline, month 1, and month 2. Only AMD patients with measurements at all visits are included. The median, interquartile range, and outliers are displayed for each time point. Abbreviations: FDR, false discovery rate; ns, non−significant; * p < 0.05; ** p < 0.01; *** p < 0.001.

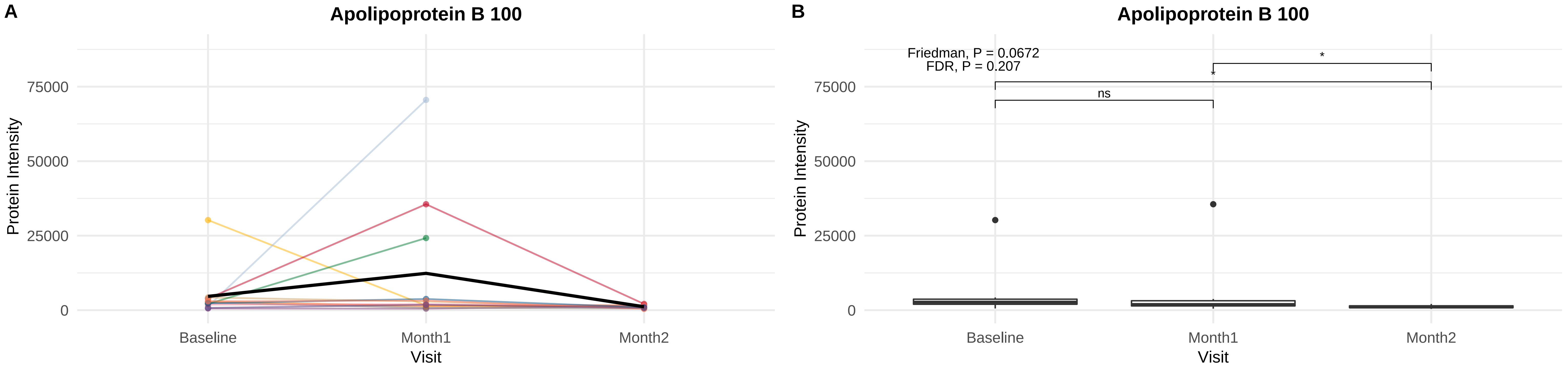

Supplementary Figure S 25

A) Line plot illustrating individual patient trajectories of Apolipoprotein B 100 intensity over time. The bold black line indicates the mean intensity over time. B) Box plots depicting the distribution of Apolipoprotein B 100 intensities at baseline, month 1, and month 2. Only AMD patients with measurements at all visits are included. The median, interquartile range, and outliers are displayed for each time point. Abbreviations: FDR, false discovery rate; ns, non−significant; * p < 0.05; ** p < 0.01; *** p < 0.001.

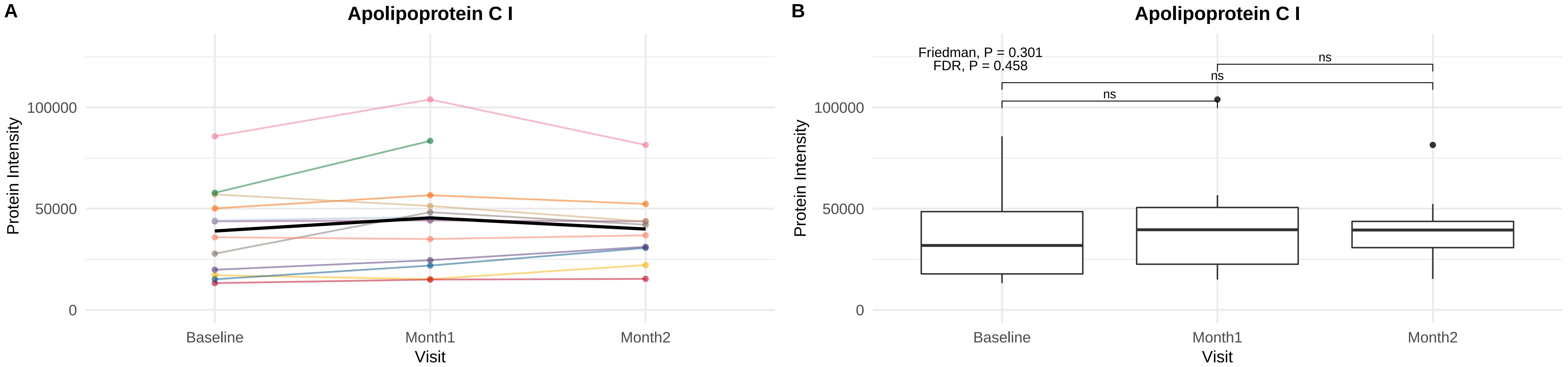

Supplementary Figure S 26

A) Line plot illustrating individual patient trajectories of Apolipoprotein C I intensity over time. The bold black line indicates the mean intensity over time. B) Box plots depicting the distribution of Apolipoprotein C I intensities at baseline, month 1, and month 2. Only AMD patients with measurements at all visits are included. The median, interquartile range, and outliers are displayed for each time point. Abbreviations: FDR, false discovery rate; ns, non−significant; * p < 0.05; ** p < 0.01; *** p < 0.001.

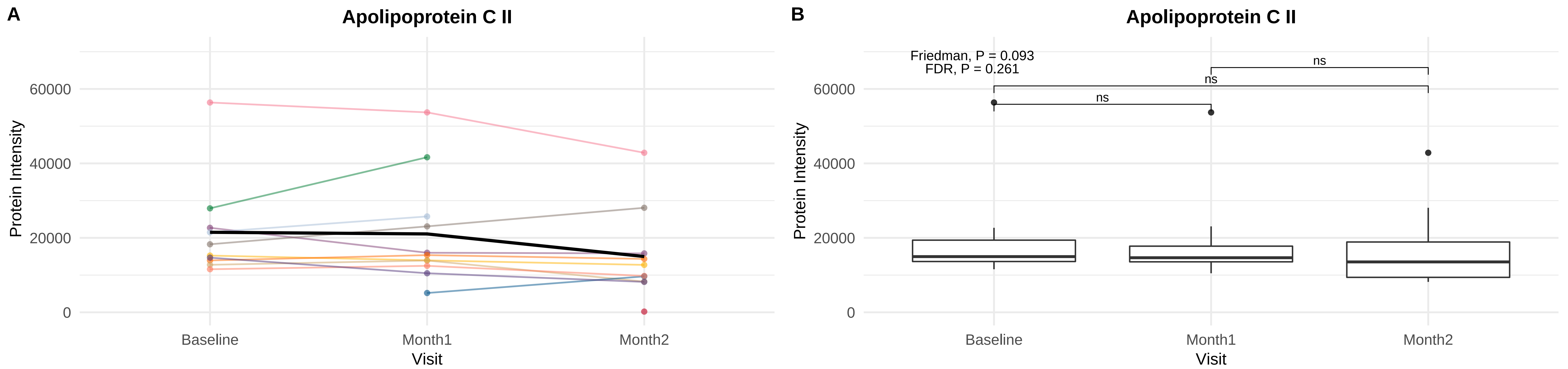

Supplementary Figure S 27

A) Line plot illustrating individual patient trajectories of Apolipoprotein C II intensity over time. The bold black line indicates the mean intensity over time. B) Box plots depicting the distribution of Apolipoprotein C II intensities at baseline, month 1, and month 2. Only AMD patients with measurements at all visits are included. The median, interquartile range, and outliers are displayed for each time point. Abbreviations: FDR, false discovery rate; ns, non−significant; * p < 0.05; ** p < 0.01; *** p < 0.001.

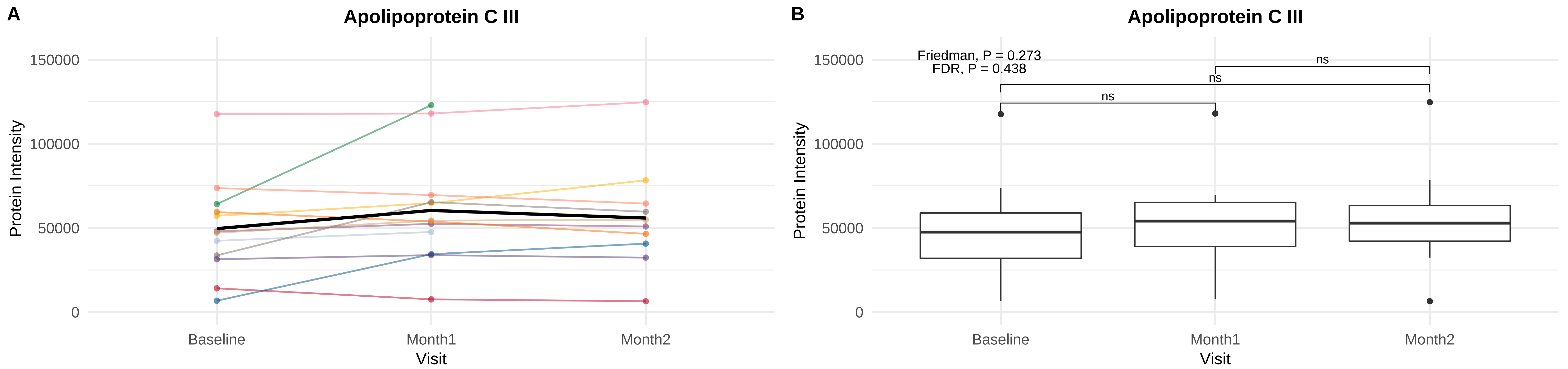

Supplementary Figure S 28

A) Line plot illustrating individual patient trajectories of Apolipoprotein C III intensity over time. The bold black line indicates the mean intensity over time. B) Box plots depicting the distribution of Apolipoprotein C III intensities at baseline, month 1, and month 2. Only AMD patients with measurements at all visits are included. The median, interquartile range, and outliers are displayed for each time point. Abbreviations: FDR, false discovery rate; ns, non−significant; * p < 0.05; ** p < 0.01; *** p < 0.001.

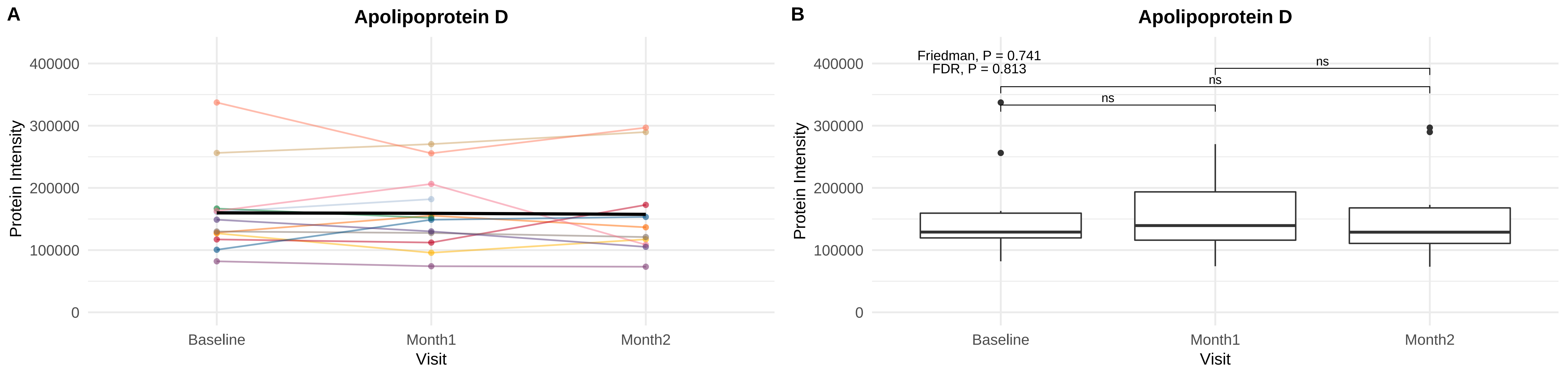

Supplementary Figure S 29

A) Line plot illustrating individual patient trajectories of Apolipoprotein D intensity over time. The bold black line indicates the mean intensity over time. B) Box plots depicting the distribution of Apolipoprotein D intensities at baseline, month 1, and month 2. Only AMD patients with measurements at all visits are included. The median, interquartile range, and outliers are displayed for each time point. Abbreviations: FDR, false discovery rate; ns, non−significant; * p < 0.05; ** p < 0.01; *** p < 0.001.

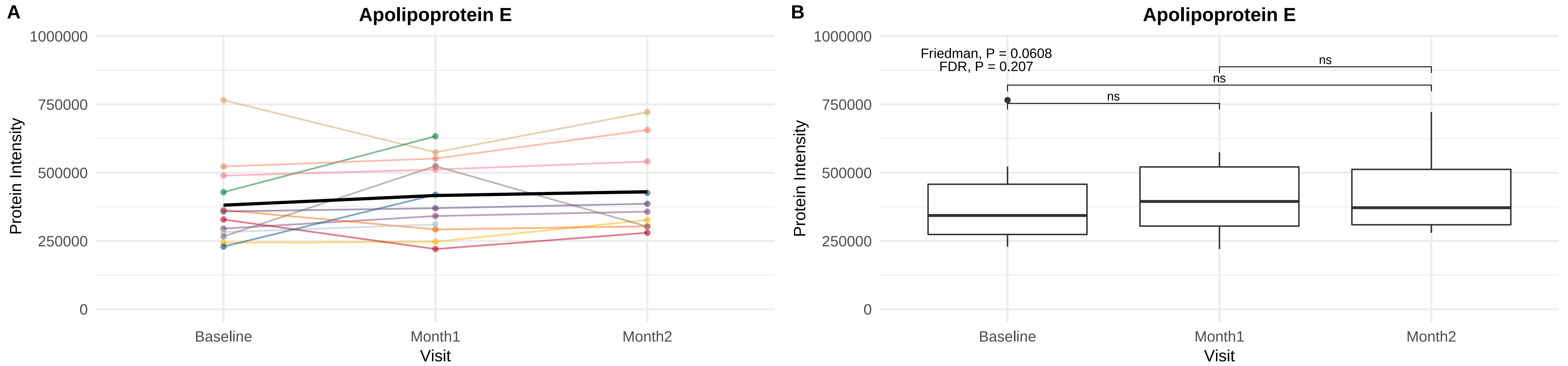

Supplementary Figure S 30

A) Line plot illustrating individual patient trajectories of Apolipoprotein E intensity over time. The bold black line indicates the mean intensity over time. B) Box plots depicting the distribution of Apolipoprotein E intensities at baseline, month 1, and month 2. Only AMD patients with measurements at all visits are included. The median, interquartile range, and outliers are displayed for each time point. Abbreviations: FDR, false discovery rate; ns, non−significant; * p < 0.05; ** p < 0.01; *** p < 0.001.

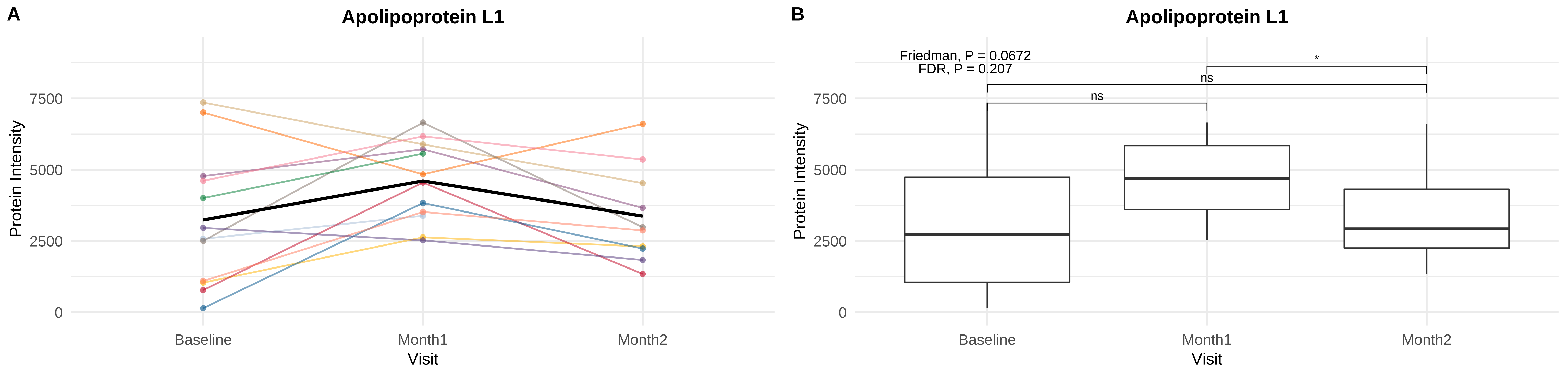

Supplementary Figure S 31

A) Line plot illustrating individual patient trajectories of Apolipoprotein L1 intensity over time. The bold black line indicates the mean intensity over time. B) Box plots depicting the distribution of Apolipoprotein L1 intensities at baseline, month 1, and month 2. Only AMD patients with measurements at all visits are included. The median, interquartile range, and outliers are displayed for each time point. Abbreviations: FDR, false discovery rate; ns, non−significant; * p < 0.05; ** p < 0.01; *** p < 0.001.

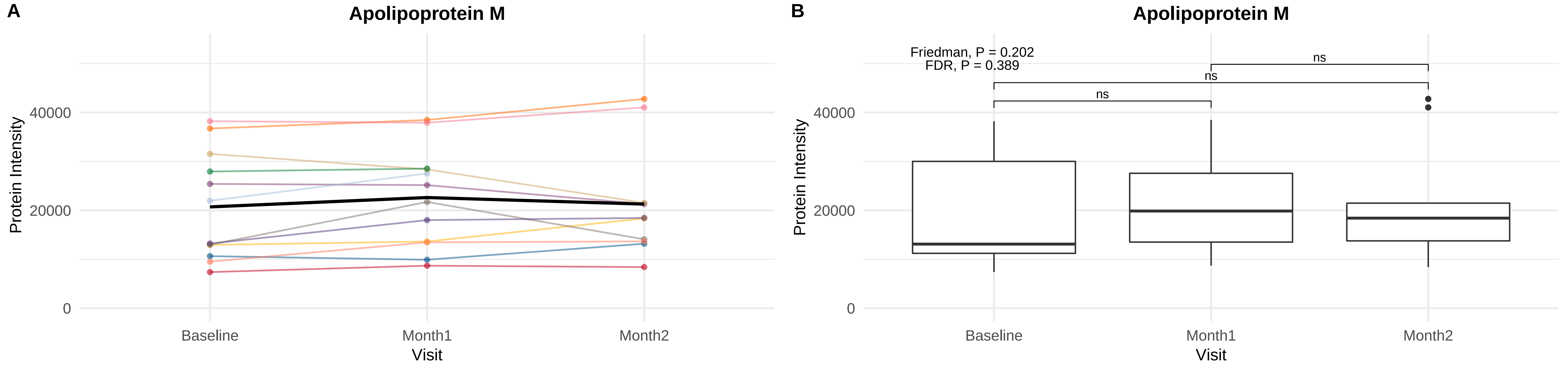

Supplementary Figure S 32

A) Line plot illustrating individual patient trajectories of Apolipoprotein M intensity over time. The bold black line indicates the mean intensity over time. B) Box plots depicting the distribution of Apolipoprotein M intensities at baseline, month 1, and month 2. Only AMD patients with measurements at all visits are included. The median, interquartile range, and outliers are displayed for each time point. Abbreviations: FDR, false discovery rate; ns, non−significant; * p < 0.05; ** p < 0.01; *** p < 0.001.

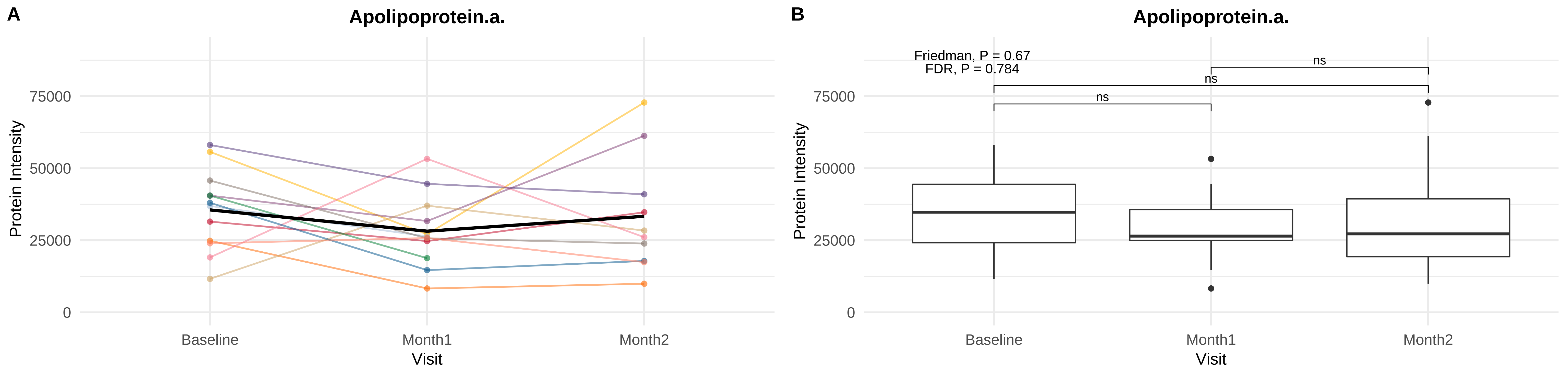

Supplementary Figure S 33

A) Line plot illustrating individual patient trajectories of Apolipoprotein.a. intensity over time. The bold black line indicates the mean intensity over time. B) Box plots depicting the distribution of Apolipoprotein.a. intensities at baseline, month 1, and month 2. Only AMD patients with measurements at all visits are included. The median, interquartile range, and outliers are displayed for each time point. Abbreviations: FDR, false discovery rate; ns, non−significant; * p < 0.05; ** p < 0.01; *** p < 0.001.

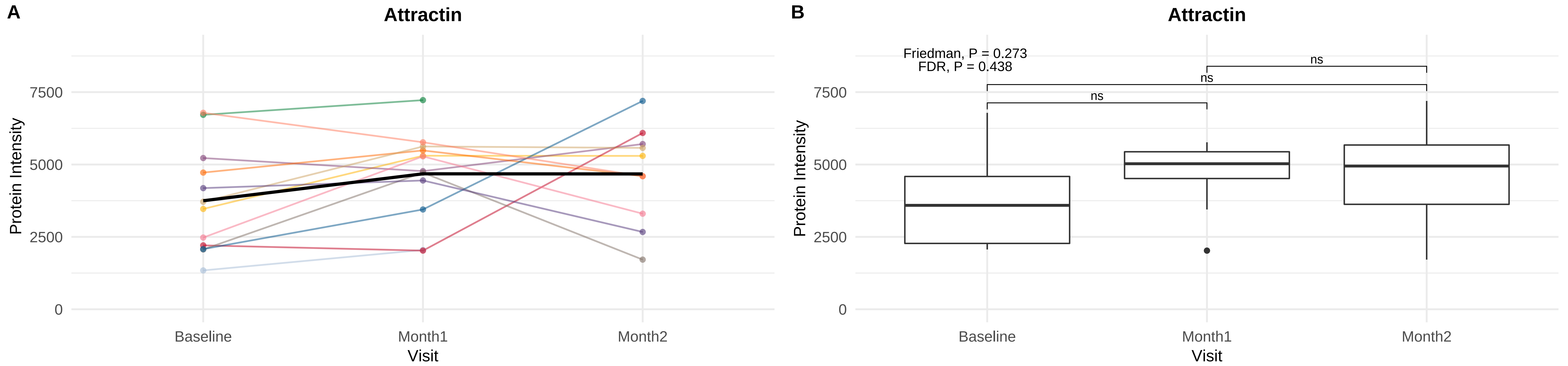

Supplementary Figure S 34

A) Line plot illustrating individual patient trajectories of Attractin intensity over time. The bold black line indicates the mean intensity over time. B) Box plots depicting the distribution of Attractin intensities at baseline, month 1, and month 2. Only AMD patients with measurements at all visits are included. The median, interquartile range, and outliers are displayed for each time point. Abbreviations: FDR, false discovery rate; ns, non−significant; * p < 0.05; ** p < 0.01; *** p < 0.001.

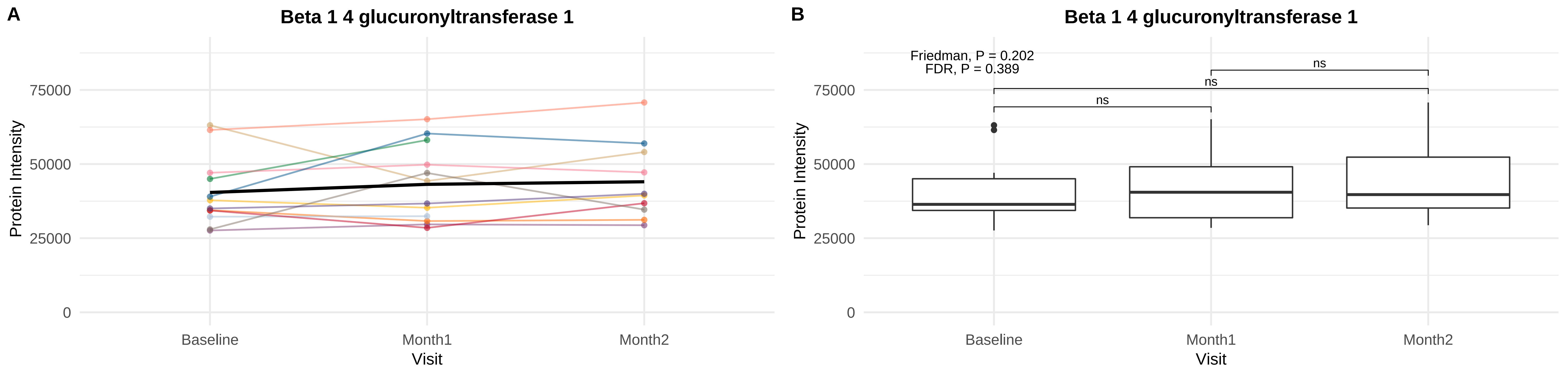

Supplementary Figure S 35

A) Line plot illustrating individual patient trajectories of Beta 1 4 glucuronyltransferase 1 intensity over time. The bold black line indicates the mean intensity over time. B) Box plots depicting the distribution of Beta 1 4 glucuronyltransferase 1 intensities at baseline, month 1, and month 2. Only AMD patients with measurements at all visits are included. The median, interquartile range, and outliers are displayed for each time point. Abbreviations: FDR, false discovery rate; ns, non−significant; * p < 0.05; ** p < 0.01; *** p < 0.001.

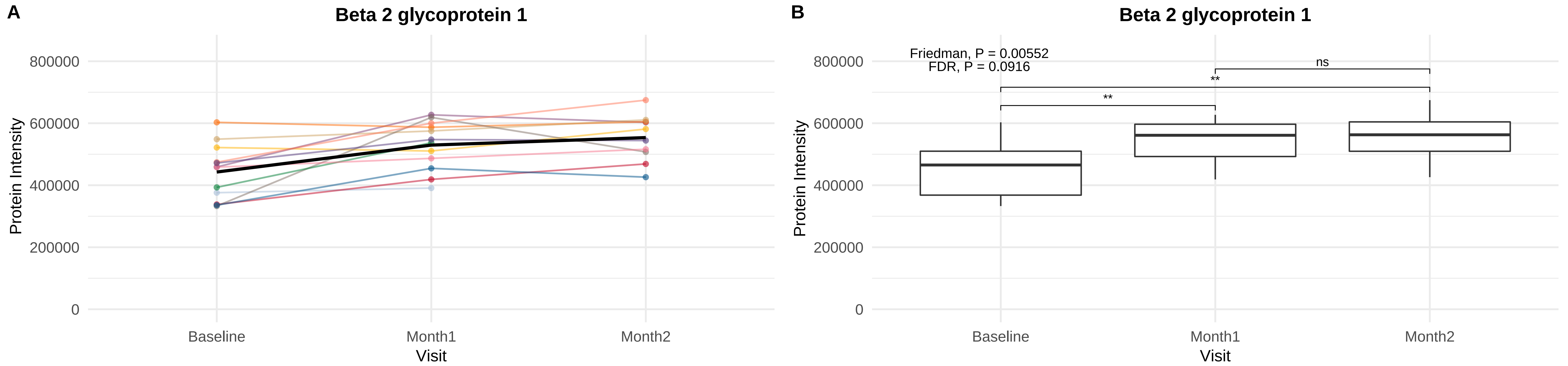

Supplementary Figure S 36

A) Line plot illustrating individual patient trajectories of Beta 2 glycoprotein 1 intensity over time. The bold black line indicates the mean intensity over time. B) Box plots depicting the distribution of Beta 2 glycoprotein 1 intensities at baseline, month 1, and month 2. Only AMD patients with measurements at all visits are included. The median, interquartile range, and outliers are displayed for each time point. Abbreviations: FDR, false discovery rate; ns, non−significant; * p < 0.05; ** p < 0.01; *** p < 0.001.

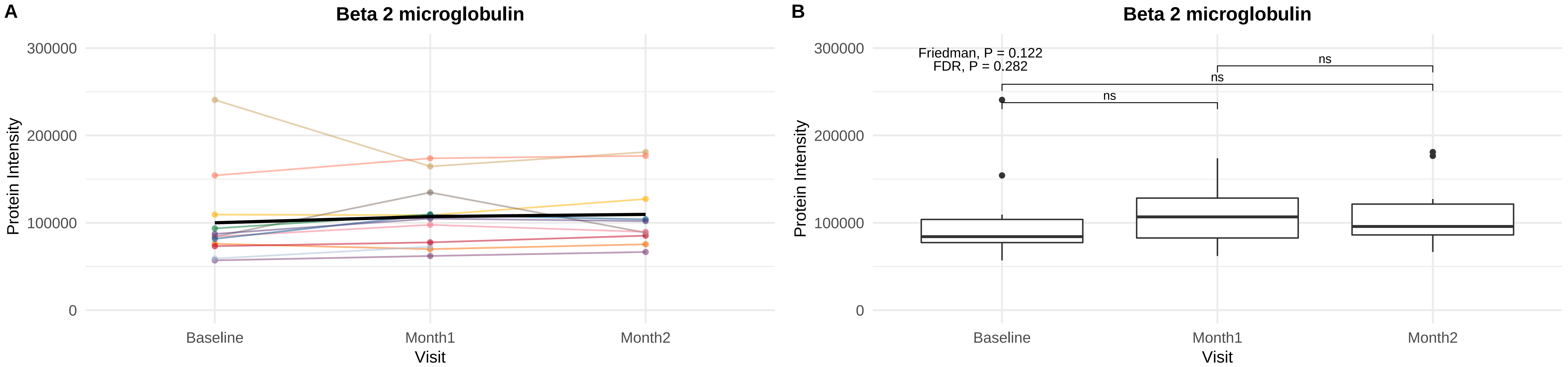

Supplementary Figure S 37

A) Line plot illustrating individual patient trajectories of Beta 2 microglobulin intensity over time. The bold black line indicates the mean intensity over time. B) Box plots depicting the distribution of Beta 2 microglobulin intensities at baseline, month 1, and month 2. Only AMD patients with measurements at all visits are included. The median, interquartile range, and outliers are displayed for each time point. Abbreviations: FDR, false discovery rate; ns, non−significant; * p < 0.05; ** p < 0.01; *** p < 0.001.

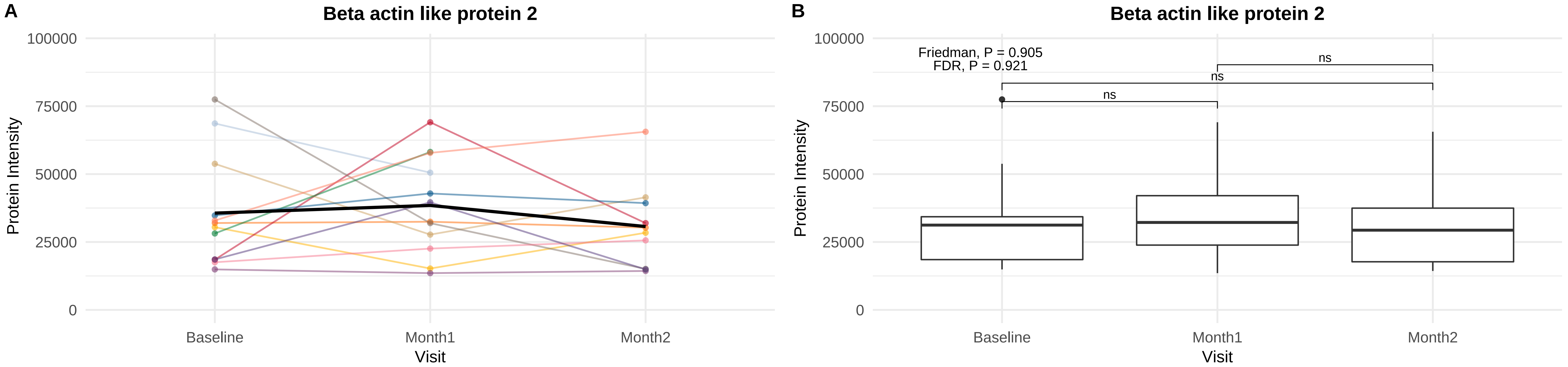

Supplementary Figure S 38

A) Line plot illustrating individual patient trajectories of Beta actin like protein 2 intensity over time. The bold black line indicates the mean intensity over time. B) Box plots depicting the distribution of Beta actin like protein 2 intensities at baseline, month 1, and month 2. Only AMD patients with measurements at all visits are included. The median, interquartile range, and outliers are displayed for each time point. Abbreviations: FDR, false discovery rate; ns, non−significant; * p < 0.05; ** p < 0.01; *** p < 0.001.

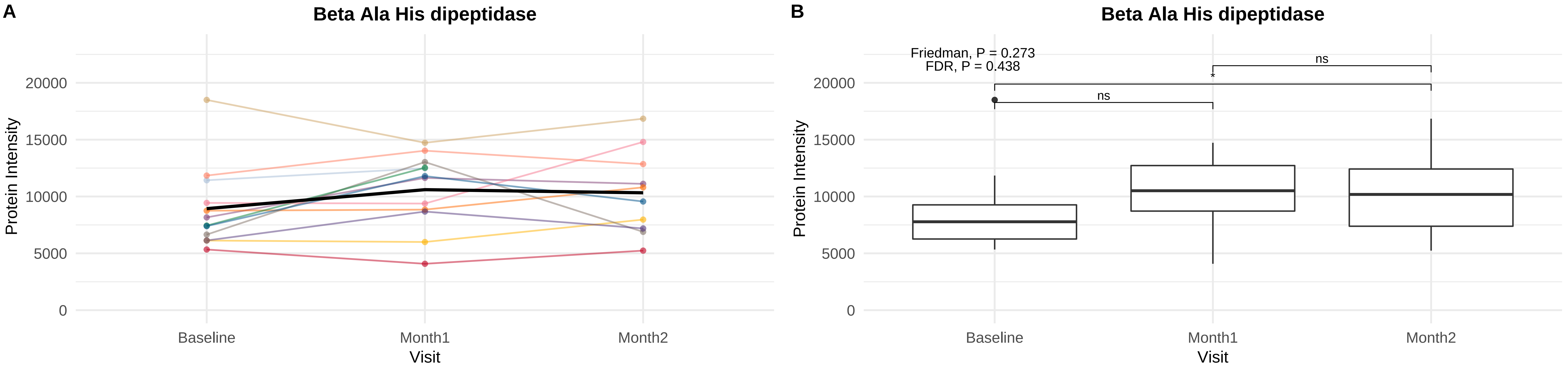

Supplementary Figure S 39

A) Line plot illustrating individual patient trajectories of Beta Ala His dipeptidase intensity over time. The bold black line indicates the mean intensity over time. B) Box plots depicting the distribution of Beta Ala His dipeptidase intensities at baseline, month 1, and month 2. Only AMD patients with measurements at all visits are included. The median, interquartile range, and outliers are displayed for each time point. Abbreviations: FDR, false discovery rate; ns, non−significant; * p < 0.05; ** p < 0.01; *** p < 0.001.

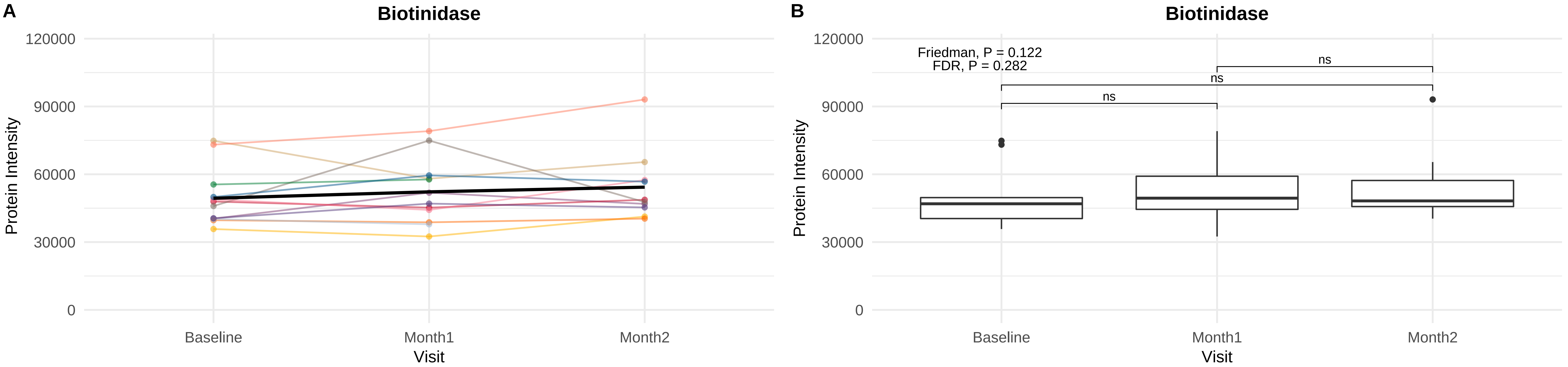

Supplementary Figure S 40

A) Line plot illustrating individual patient trajectories of Biotinidase intensity over time. The bold black line indicates the mean intensity over time. B) Box plots depicting the distribution of Biotinidase intensities at baseline, month 1, and month 2. Only AMD patients with measurements at all visits are included. The median, interquartile range, and outliers are displayed for each time point. Abbreviations: FDR, false discovery rate; ns, non−significant; * p < 0.05; ** p < 0.01; *** p < 0.001.

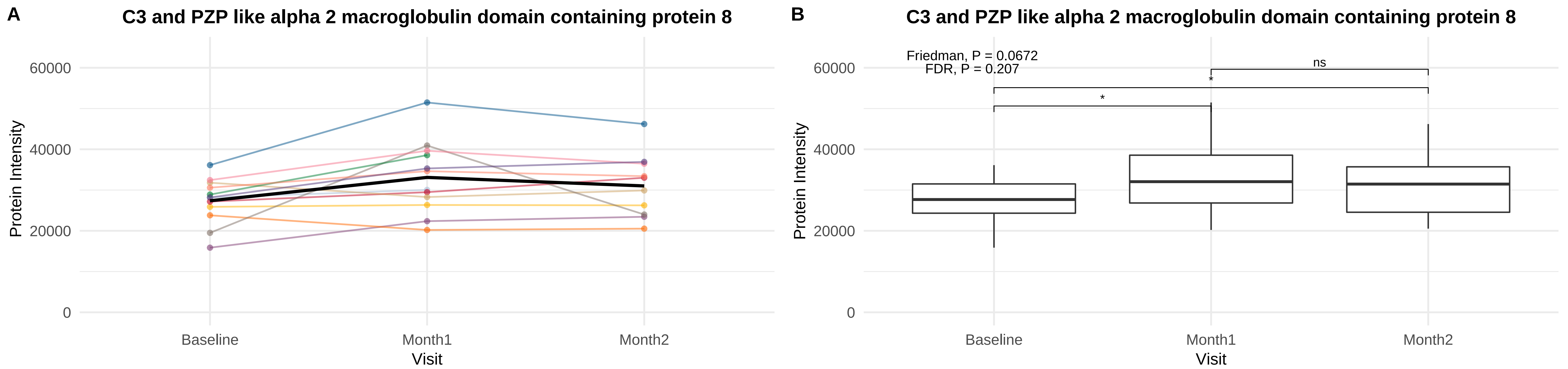

Supplementary Figure S 41

A) Line plot illustrating individual patient trajectories of C3 and PZP like alpha 2 macroglobulin domain containing protein 8 intensity over time. The bold black line indicates the mean intensity over time. B) Box plots depicting the distribution of C3 and PZP like alpha 2 macroglobulin domai containing protein 8 intensities at baseline, month 1, and month 2. Only AMD patients with measurements at all visits are included. The median, interquartile range, and outliers are displayed for each time point. Abbreviations: FDR, false discovery rate; ns, non−significant; * p < 0.05; ** p 0.01; *** p < 0.001.

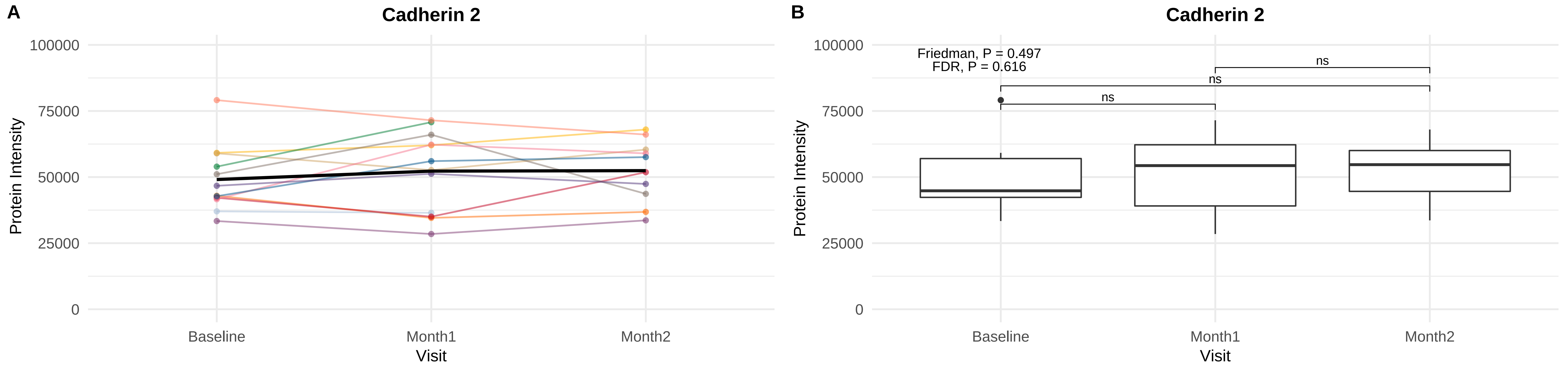

Supplementary Figure S 42

A) Line plot illustrating individual patient trajectories of Cadherin 2 intensity over time. The bold black line indicates the mean intensity over time. B) Box plots depicting the distribution of Cadherin 2 intensities at baseline, month 1, and month 2. Only AMD patients with measurements at all visits are included. The median, interquartile range, and outliers are displayed for each time point. Abbreviations: FDR, false discovery rate; ns, non−significant; * p < 0.05; ** p < 0.01; *** p < 0.001.

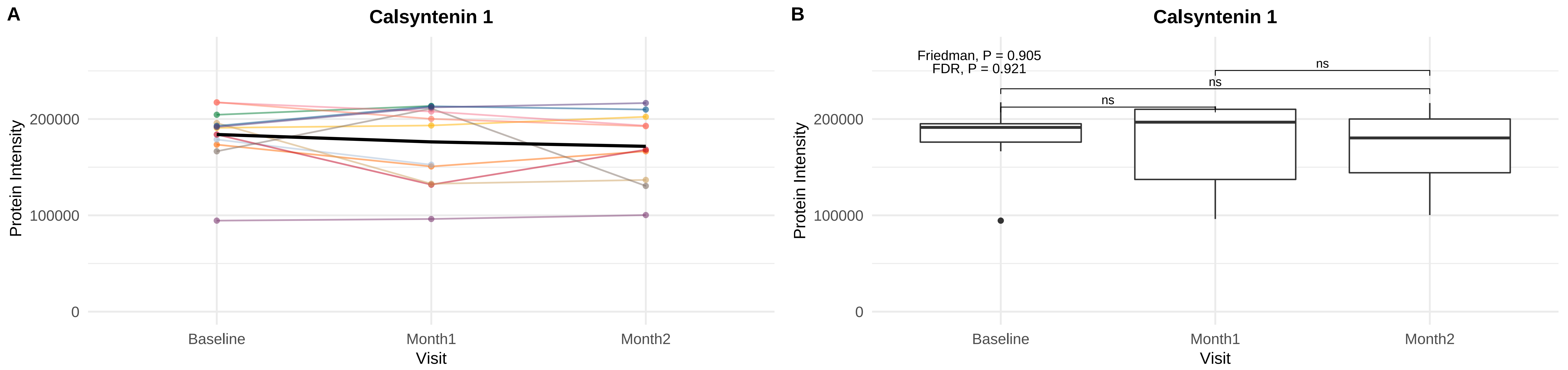

Supplementary Figure S 43

A) Line plot illustrating individual patient trajectories of Calsyntenin 1 intensity over time. The bold black line indicates the mean intensity over time. B) Box plots depicting the distribution of Calsyntenin 1 intensities at baseline, month 1, and month 2. Only AMD patients with measurements at all visits are included. The median, interquartile range, and outliers are displayed for each time point. Abbreviations: FDR, false discovery rate; ns, non−significant; * p < 0.05; ** p < 0.01; *** p < 0.001.

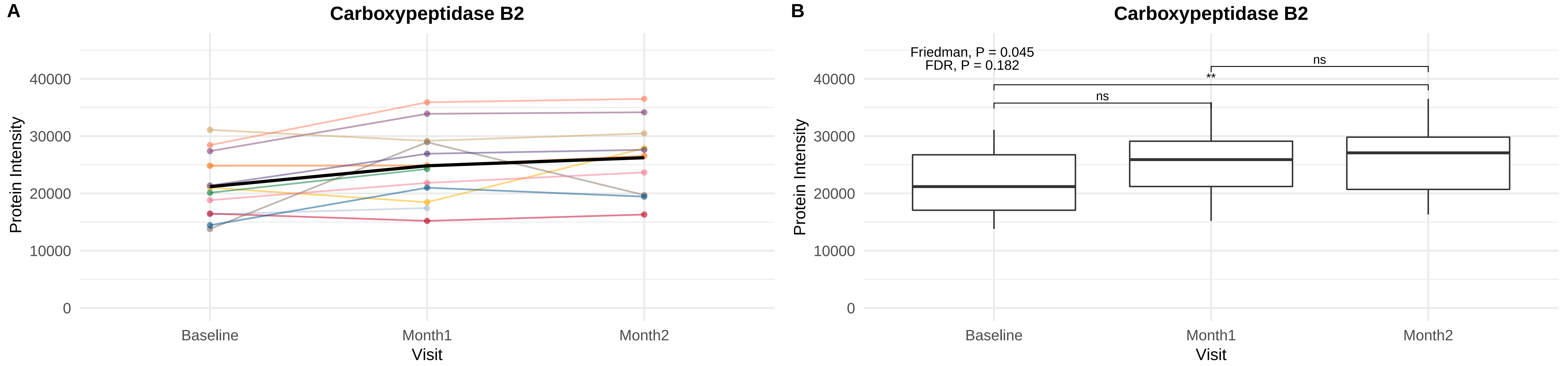

Supplementary Figure S 44

A) Line plot illustrating individual patient trajectories of Carboxypeptidase B2 intensity over time. The bold black line indicates the mean intensity over time. B) Box plots depicting the distribution of Carboxypeptidase B2 intensities at baseline, month 1, and month 2. Only AMD patients with measurements at all visits are included. The median, interquartile range, and outliers are displayed for each time point. Abbreviations: FDR, false discovery rate; ns, non−significant; * p < 0.05; ** p < 0.01; *** p < 0.001.

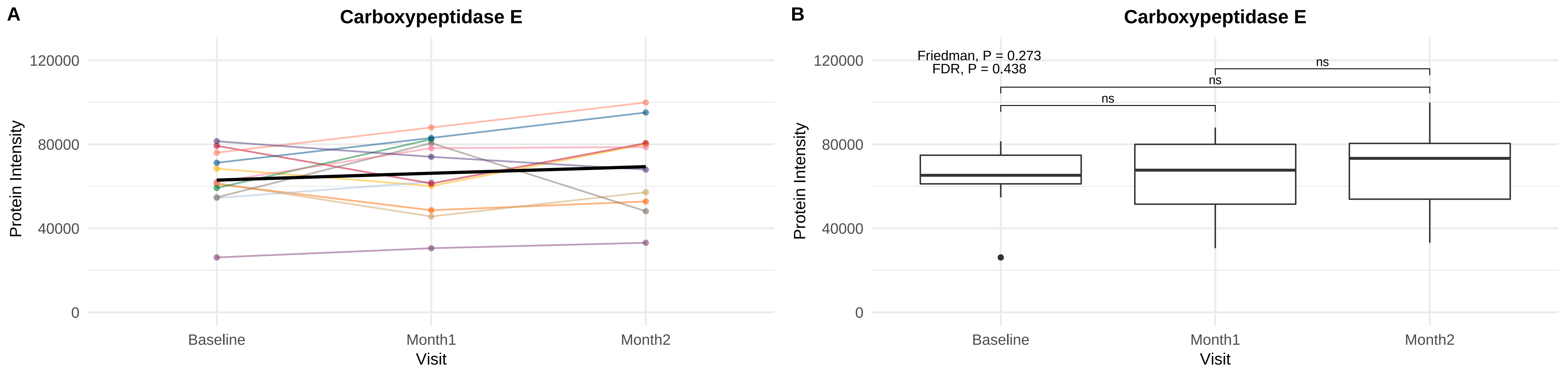

Supplementary Figure S 45

A) Line plot illustrating individual patient trajectories of Carboxypeptidase E intensity over time. The bold black line indicates the mean intensity over time. B) Box plots depicting the distribution of Carboxypeptidase E intensities at baseline, month 1, and month 2. Only AMD patients with measurements at all visits are included. The median, interquartile range, and outliers are displayed for each time point. Abbreviations: FDR, false discovery rate; ns, non−significant; * p < 0.05; ** p < 0.01; *** p < 0.001.

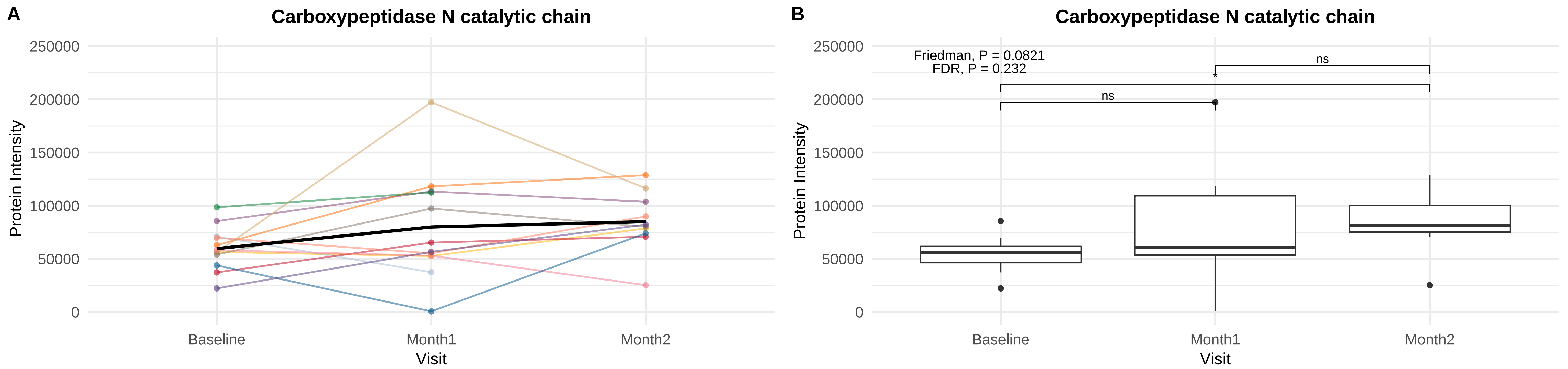

Supplementary Figure S 46

A) Line plot illustrating individual patient trajectories of Carboxypeptidase N catalytic chain intensity over time. The bold black line indicates the mean intensity over time. B) Box plots depicting the distribution of Carboxypeptidase N catalytic chain intensities at baseline, month 1, and month 2. Only AMD patients with measurements at all visits are included. The median, interquartile range, and outliers are displayed for each time point. Abbreviations: FDR, false discovery rate; ns, non−significant; * p < 0.05; ** p < 0.01; *** p < 0.001.

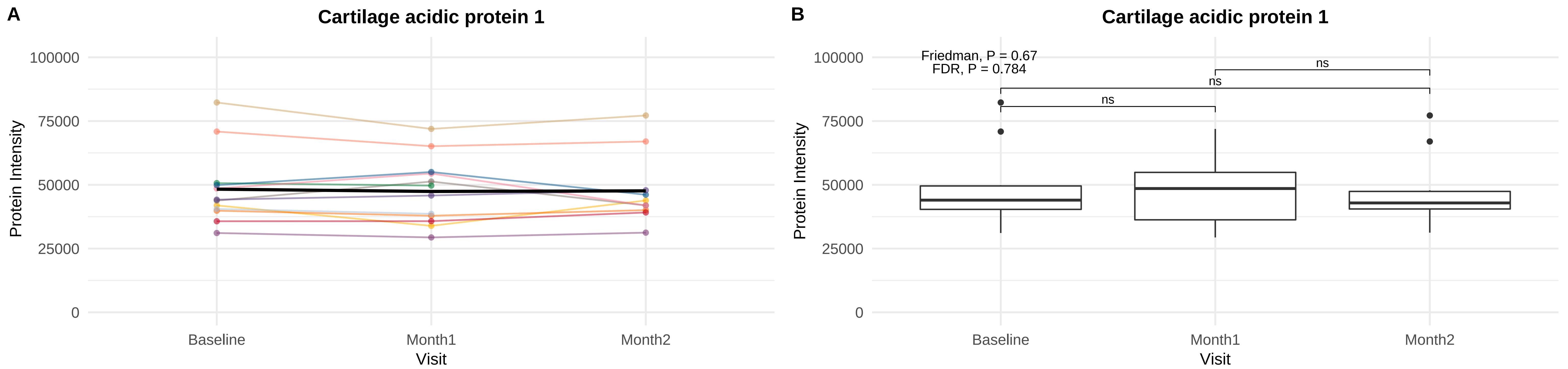

Supplementary Figure S 47

A) Line plot illustrating individual patient trajectories of Cartilage acidic protein 1 intensity over time. The bold black line indicates the mean intensity over time. B) Box plots depicting the distribution of Cartilage acidic protein 1 intensities at baseline, month 1, and month 2. Only AMD patients with measurements at all visits are included. The median, interquartile range, and outliers are displayed for each time point. Abbreviations: FDR, false discovery rate; ns, non−significant; * p < 0.05; ** p < 0.01; *** p < 0.001.

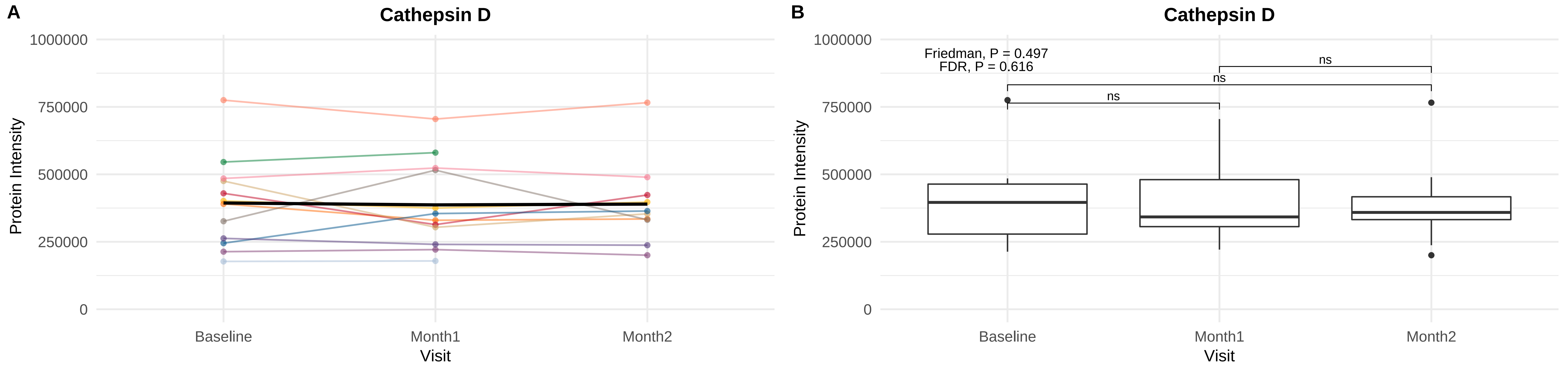

Supplementary Figure S 48

A) Line plot illustrating individual patient trajectories of Cathepsin D intensity over time. The bold black line indicates the mean intensity over time. B) Box plots depicting the distribution of Cathepsin D intensities at baseline, month 1, and month 2. Only AMD patients with measurements at all visits are included. The median, interquartile range, and outliers are displayed for each time point. Abbreviations: FDR, false discovery rate; ns, non−significant; * p < 0.05; ** p < 0.01; *** p < 0.001.

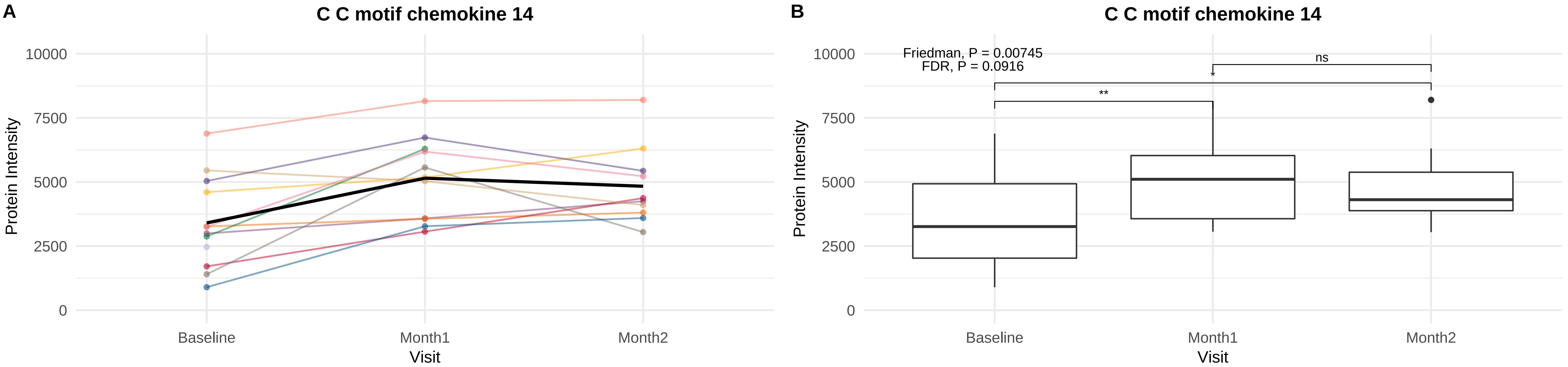

Supplementary Figure S 49

A) Line plot illustrating individual patient trajectories of C C motif chemokine 14 intensity over time. The bold black line indicates the mean intensity over time. B) Box plots depicting the distribution of C C motif chemokine 14 intensities at baseline, month 1, and month 2. Only AMD patients with measurements at all visits are included. The median, interquartile range, and outliers are displayed for each time point. Abbreviations: FDR, false discovery rate; ns, non−significant; * p < 0.05; ** p < 0.01; *** p < 0.001.

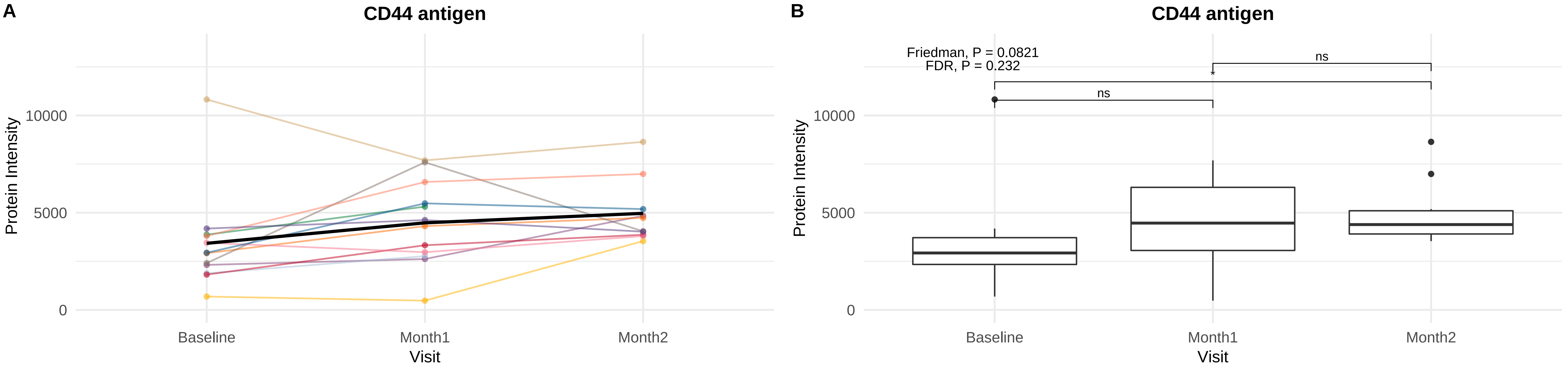

Supplementary Figure S 50

A) Line plot illustrating individual patient trajectories of CD44 antigen intensity over time. The bold black line indicates the mean intensity over time. B) Box plots depicting the distribution of CD44 antigen intensities at baseline, month 1, and month 2. Only AMD patients with measurements at all visits are included. The median, interquartile range, and outliers are displayed for each time point. Abbreviations: FDR, false discovery rate; ns, non−significant; * p < 0.05; ** p < 0.01; *** p < 0.001.

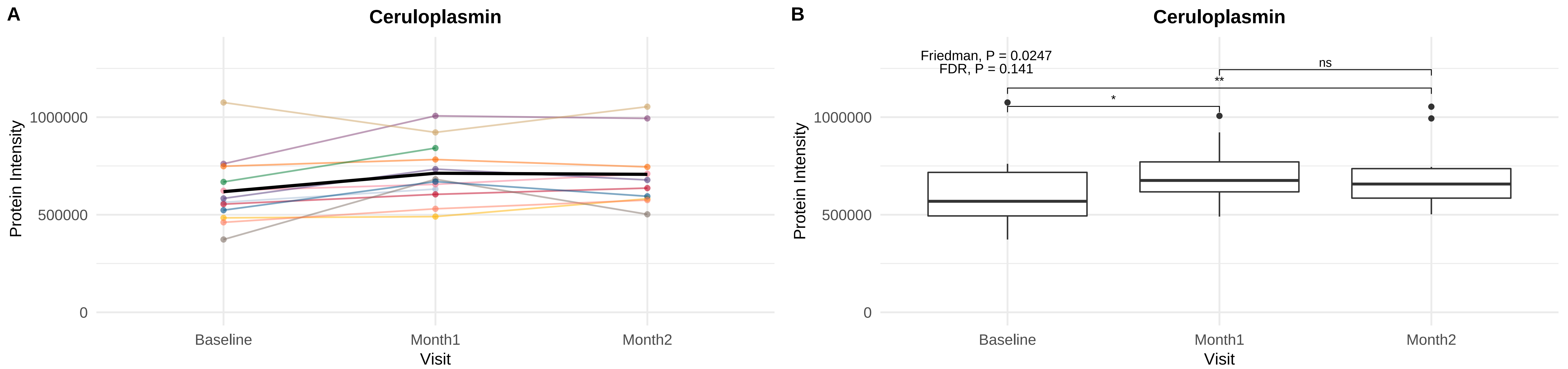

Supplementary Figure S 51

A) Line plot illustrating individual patient trajectories of Ceruloplasmin intensity over time. The bold black line indicates the mean intensity over time. B) Box plots depicting the distribution of Ceruloplasmin intensities at baseline, month 1, and month 2. Only AMD patients with measurement at all visits are included. The median, interquartile range, and outliers are displayed for each time point. Abbreviations: FDR, false discovery rate; ns, non−significant; * p < 0.05; ** p < 0.01; *** p < 0.001.

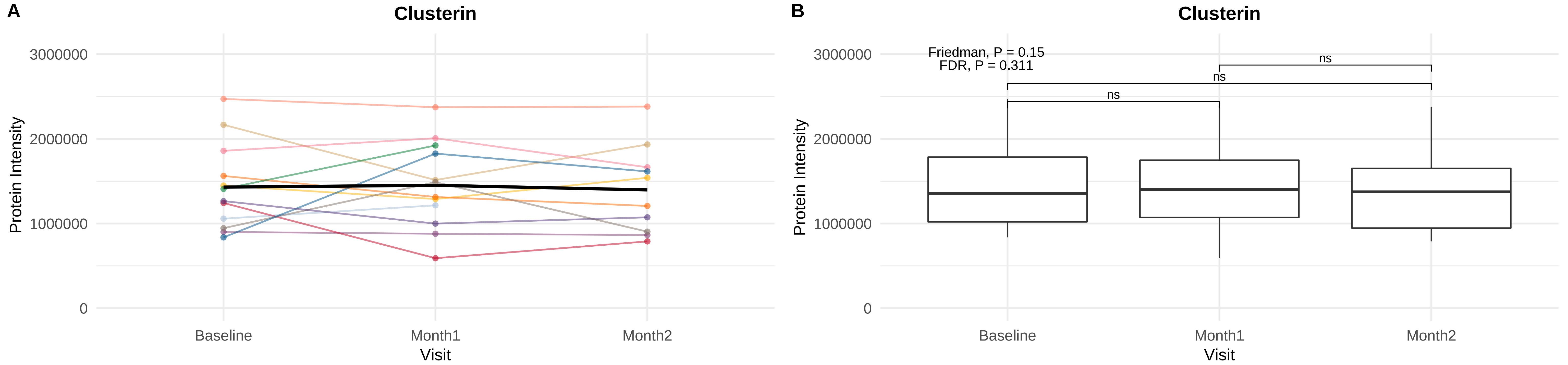

Supplementary Figure S 52

A) Line plot illustrating individual patient trajectories of Clusterin intensity over time. The bold black line indicates the mean intensity over time. B) Box plots depicting the distribution of Clusterin intensities at baseline, month 1, and month 2. Only AMD patients with measurements at all visits are included. The median, interquartile range, and outliers are displayed for each time point. Abbreviations: FDR, false discovery rate; ns, non−significant; * p < 0.05; ** p < 0.01; *** p < 0.001.

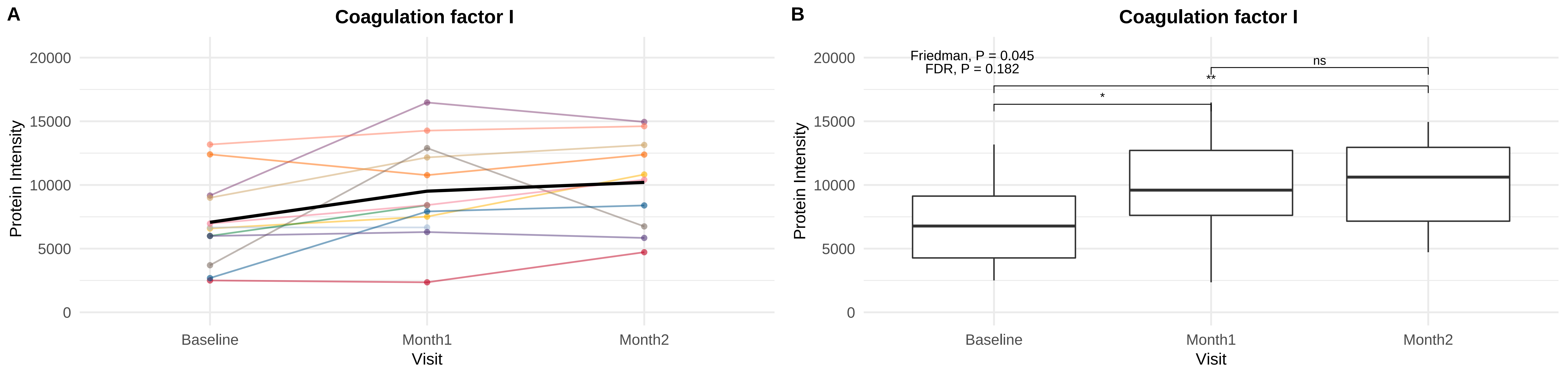

Supplementary Figure S 53

A) Line plot illustrating individual patient trajectories of Coagulation factor I intensity over time. The bold black line indicates the mean intensity over time. B) Box plots depicting the distribution of Coagulation factor I intensities at baseline, month 1, and month 2. Only AMD patients with measurements at all visits are included. The median, interquartile range, and outliers are displayed for each time point. Abbreviations: FDR, false discovery rate; ns, non−significant; * p < 0.05; ** p < 0.01; *** p < 0.001.

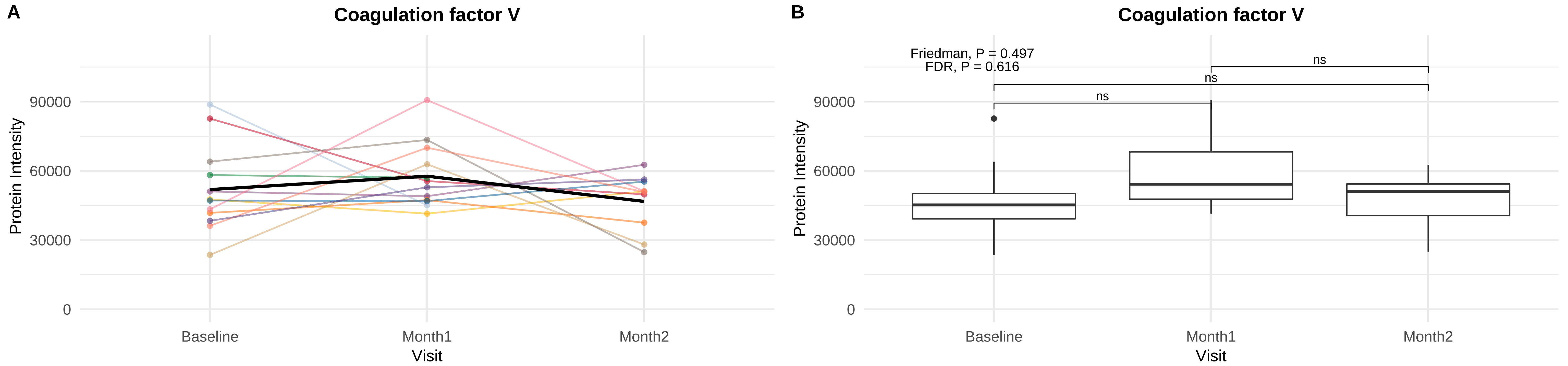

Supplementary Figure S 54

A) Line plot illustrating individual patient trajectories of Coagulation factor V intensity over time. The bold black line indicates the mean intensity over time. B) Box plots depicting the distribution of Coagulation factor V intensities at baseline, month 1, and month 2. Only AMD patients with measurements at all visits are included. The median, interquartile range, and outliers are displayed for each time point. Abbreviations: FDR, false discovery rate; ns, non−significant; * p < 0.05; ** p < 0.01; *** p < 0.001.

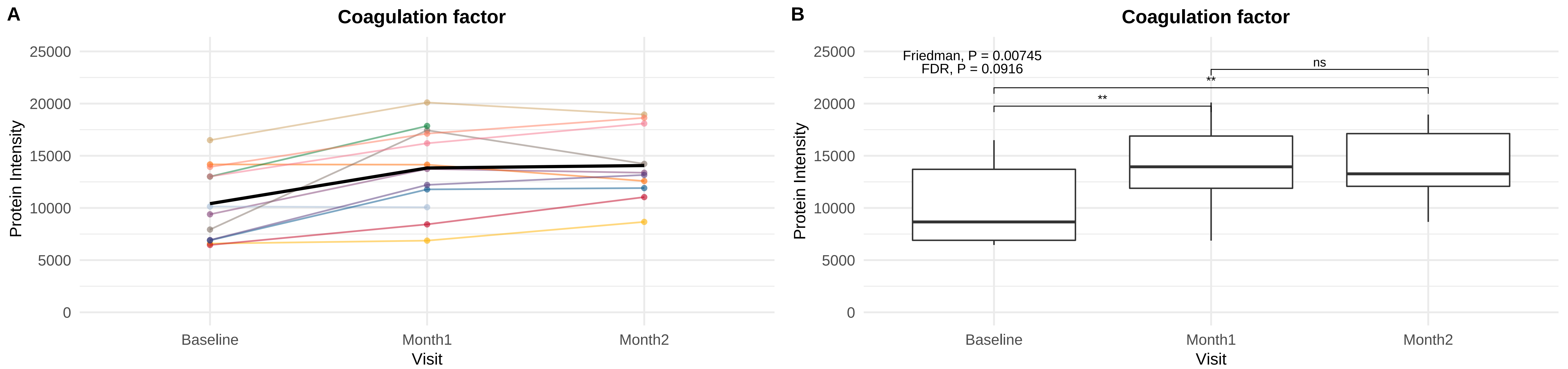

Supplementary Figure S 55

A) Line plot illustrating individual patient trajectories of Coagulation factor intensity over time. The bold black line indicates the mean intensity over time. B) Box plots depicting the distribution of Coagulation factor intensities at baseline, month 1, and month 2. Only AMD patients with measurements at all visits are included. The median, interquartile range, and outliers are displayed for each time point. Abbreviations: FDR, false discovery rate; ns, non−significant; * p < 0.05; ** p < 0.01; *** p < 0.001.

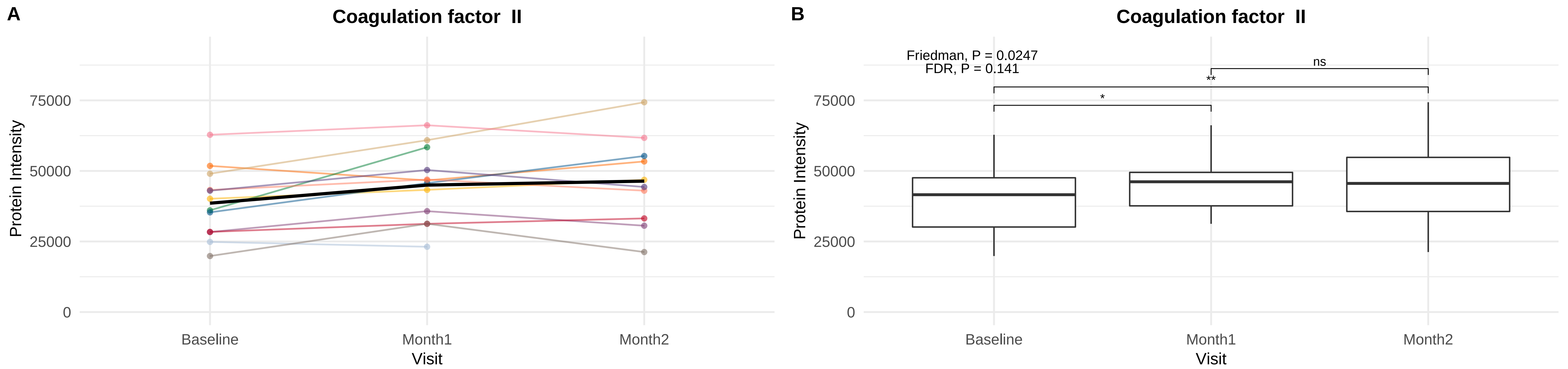

Supplementary Figure S 56

A) Line plot illustrating individual patient trajectories of Coagulation factor II intensity over time. The bold black line indicates the mean intensity over time. B) Box plots depicting the distribution of Coagulation factor II intensities at baseline, month 1, and month 2. Only AMD patients with measurements at all visits are included. The median, interquartile range, and outliers are displayed for each time point. Abbreviations: FDR, false discovery rate; ns, non−significant; * p < 0.05; ** p < 0.01; *** p < 0.001.

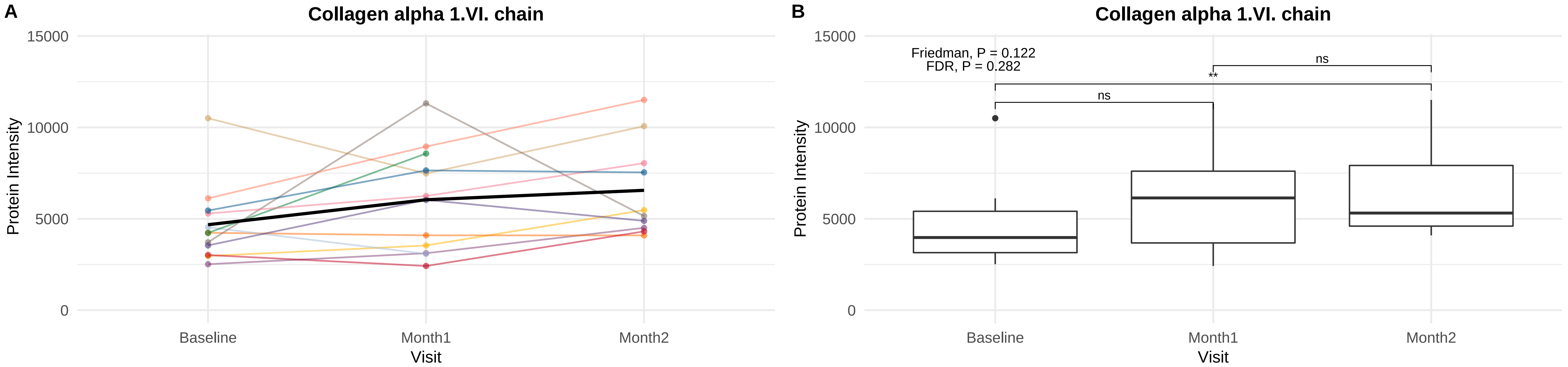

Supplementary Figure S 57

A) Line plot illustrating individual patient trajectories of Collagen alpha 1.VI. chain intensity over time. The bold black line indicates the mean intensity over time. B) Box plots depicting the distribution of Collagen alpha 1.VI. chain intensities at baseline, month 1, and month 2. Only AMD patients with measurements at all visits are included. The median, interquartile range, and outliers are displayed for each time point. Abbreviations: FDR, false discovery rate; ns, non−significant; * p < 0.05; ** p < 0.01; *** p < 0.001.

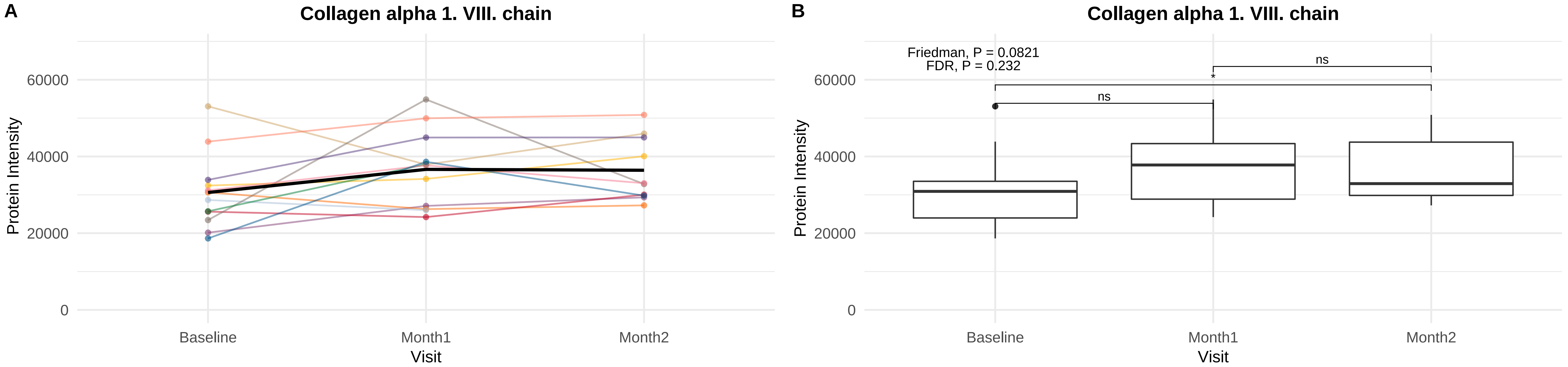

Supplementary Figure S 58

A) Line plot illustrating individual patient trajectories of Collagen alpha 1. VIII. chain intensity over time. The bold black line indicates the mean intensity over time. B) Box plots depicting the distribution of Collagen alpha 1. VIII. chain intensities at baseline, month 1, and month 2. Only AMD patients with measurements at all visits are included. The median, interquartile range, and outliers are displayed for each time point. Abbreviations: FDR, false discovery rate; ns, non−significant; * p < 0.05; ** p < 0.01; *** p < 0.001.

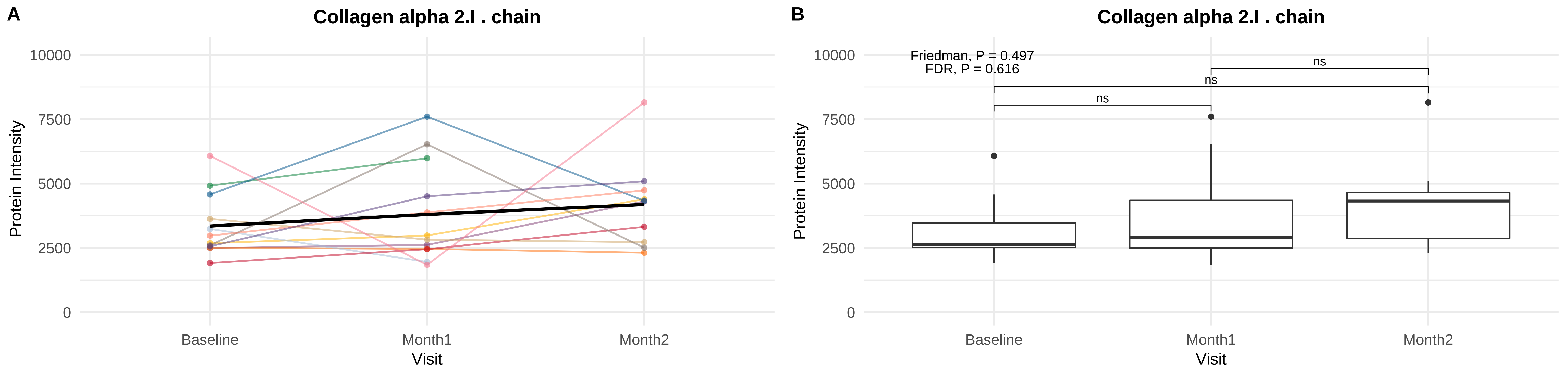

Supplementary Figure S 59

A) Line plot illustrating individual patient trajectories of Collagen alpha 2.I . chain intensity over time. The bold black line indicates the mean intensity over time. B) Box plots depicting the distribution of Collagen alpha 2.I . chain intensities at baseline, month 1, and month 2. Only AMD patients with measurements at all visits are included. The median, interquartile range, and outliers are displayed for each time point. Abbreviations: FDR, false discovery rate; ns, non−significant; * p < 0.05; ** p < 0.01; *** p < 0.001.

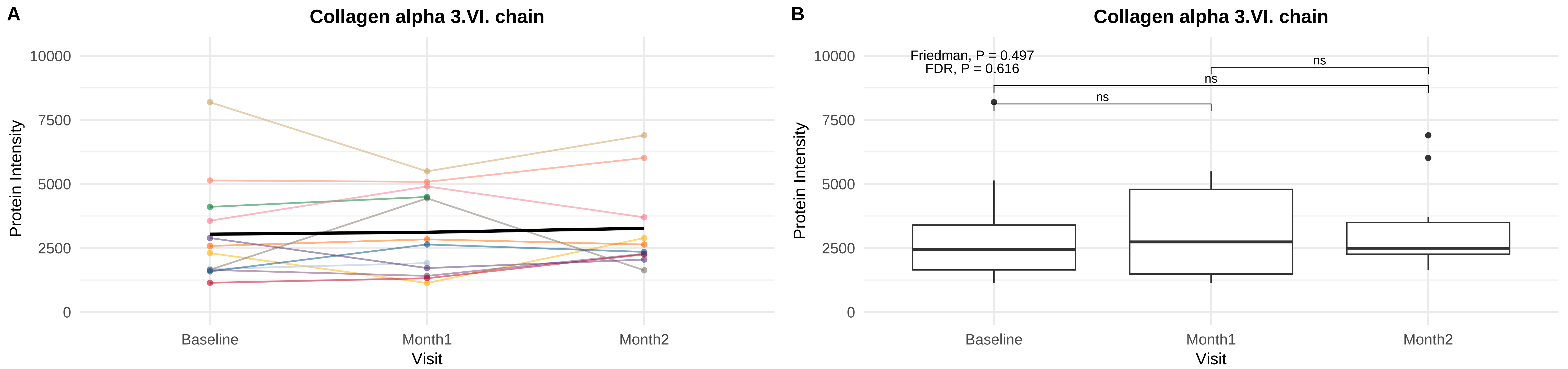

Supplementary Figure S 60

A) Line plot illustrating individual patient trajectories of Collagen alpha 3.VI. chain intensity over time. The bold black line indicates the mean intensity over time. B) Box plots depicting the distribution of Collagen alpha 3.VI. chain intensities at baseline, month 1, and month 2. Only AMD patients with measurements at all visits are included. The median, interquartile range, and outliers are displayed for each time point. Abbreviations: FDR, false discovery rate; ns, non−significant; * p < 0.05; ** p < 0.01; *** p < 0.001.

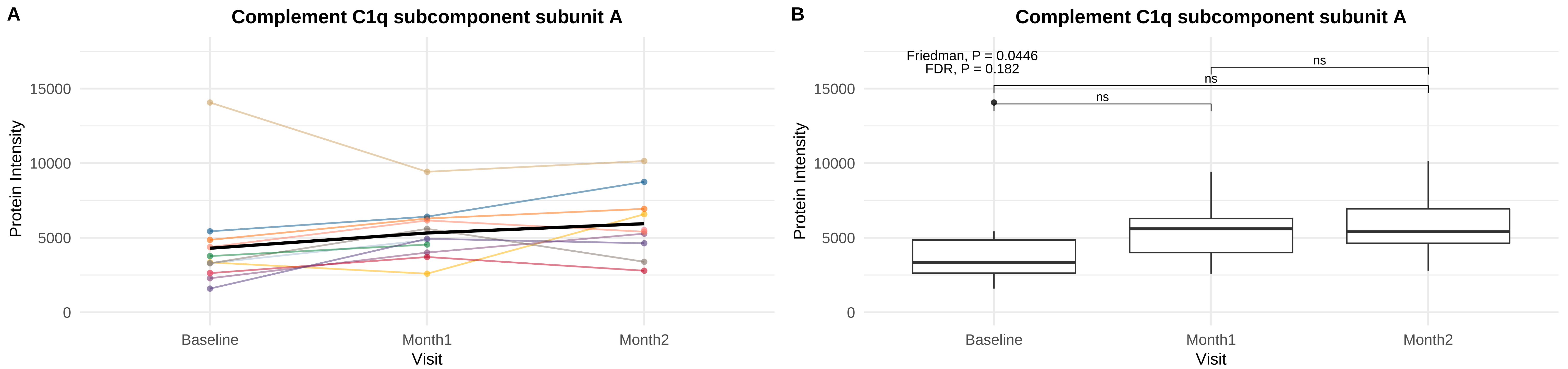

Supplementary Figure S 61

A) Line plot illustrating individual patient trajectories of Complement C1q subcomponent subunit A intensity over time. The bold black line indicates the mean intensity over time. B) Box plots depicting the distribution of Complement C1q subcomponent subunit A intensities at baseline, mor and month 2. Only AMD patients with measurements at all visits are included. The median, interquartile range, and outliers are displayed for each time point. Abbreviations: FDR, false discovery rate; ns, non−significant; * p < 0.05; ** p < 0.01; *** p < 0.001.

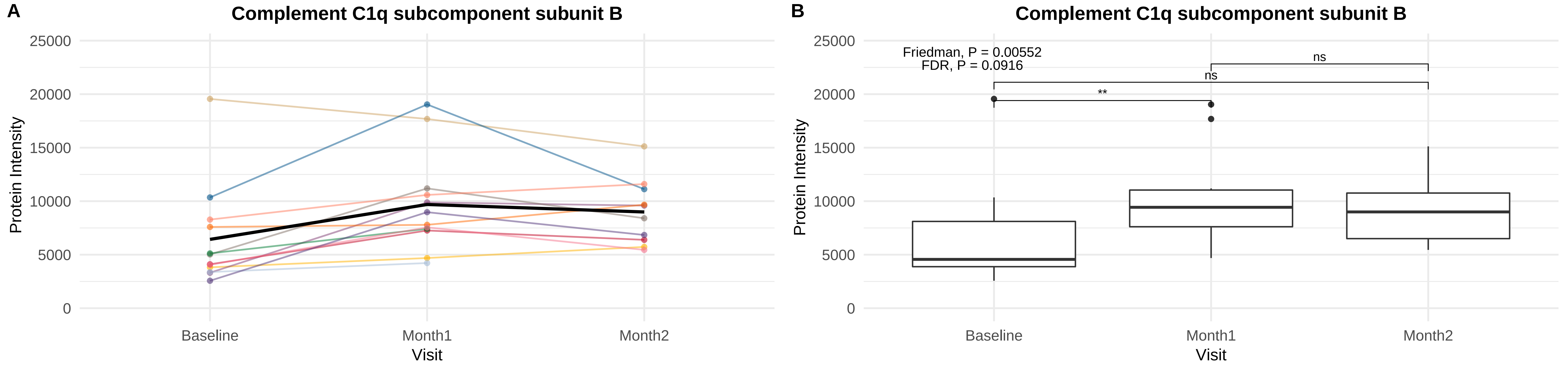

Supplementary Figure S 62

A) Line plot illustrating individual patient trajectories of Complement C1q subcomponent subunit B intensity over time. The bold black line indicates the mean intensity over time. B) Box plots depicting the distribution of Complement C1q subcomponent subunit B intensities at baseline, mor and month 2. Only AMD patients with measurements at all visits are included. The median, interquartile range, and outliers are displayed for each time point. Abbreviations: FDR, false discovery rate; ns, non−significant; * p < 0.05; ** p < 0.01; *** p < 0.001.

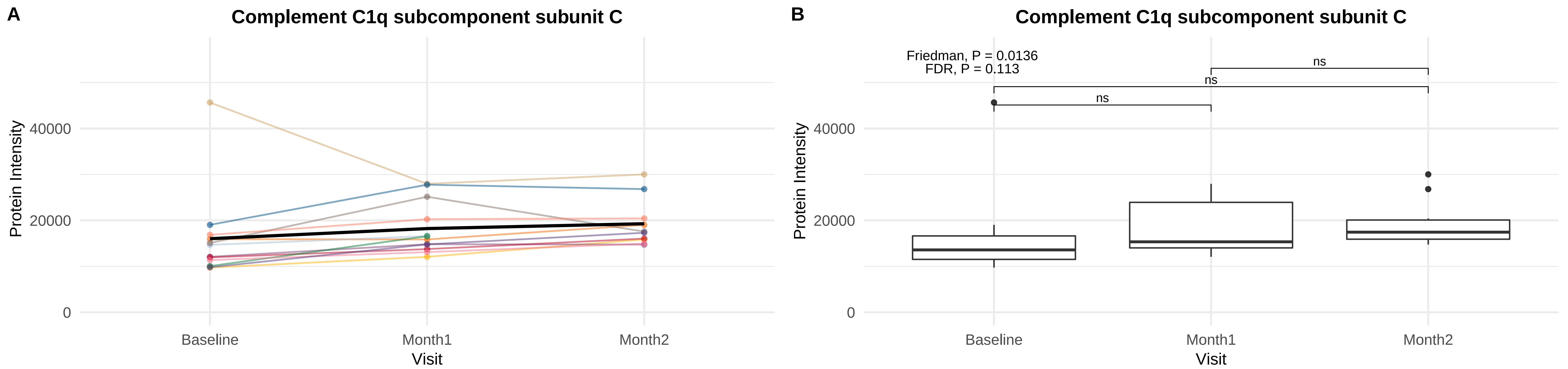

Supplementary Figure S 63

A) Line plot illustrating individual patient trajectories of Complement C1q subcomponent subunit C intensity over time. The bold black line indicates the mean intensity over time. B) Box plots depicting the distribution of Complement C1q subcomponent subunit C intensities at baseline, mon
and month 2. Only AMD patients with measurements at all visits are included. The median, interquartile range, and outliers are displayed for each time point. Abbreviations: FDR, false discovery rate; ns, non−significant; * p < 0.05; ** p < 0.01; *** p < 0.001.

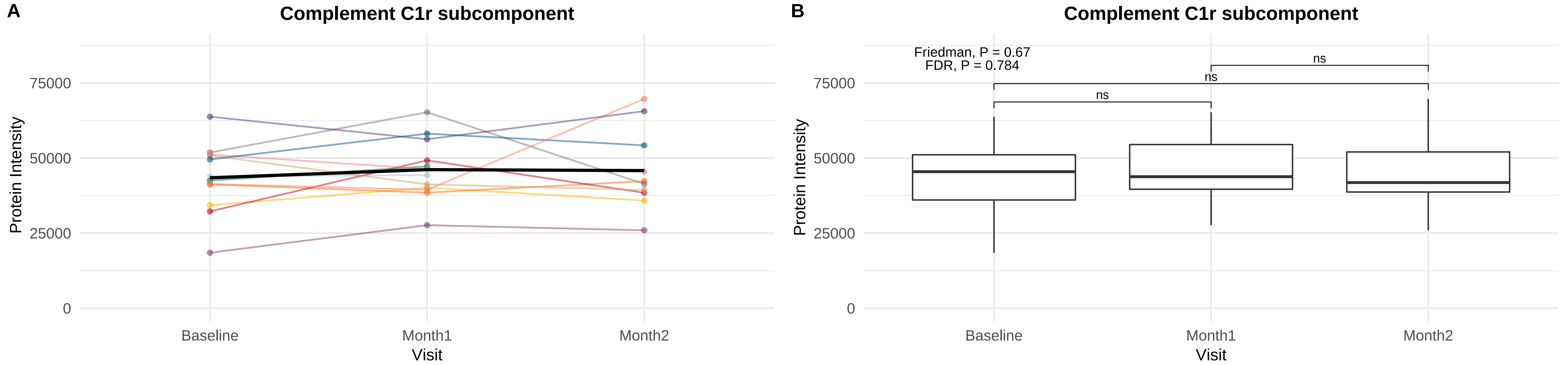

Supplementary Figure S 64

A) Line plot illustrating individual patient trajectories of Complement C1r subcomponent intensity over time. The bold black line indicates the mean intensity over time. B) Box plots depicting the distribution of Complement C1r subcomponent intensities at baseline, month 1, and month 2. O
AMD patients with measurements at all visits are included. The median, interquartile range, and outliers are displayed for each time point. Abbreviations: FDR, false discovery rate; ns, non−significant; * p < 0.05; ** p < 0.01; *** p < 0.001.

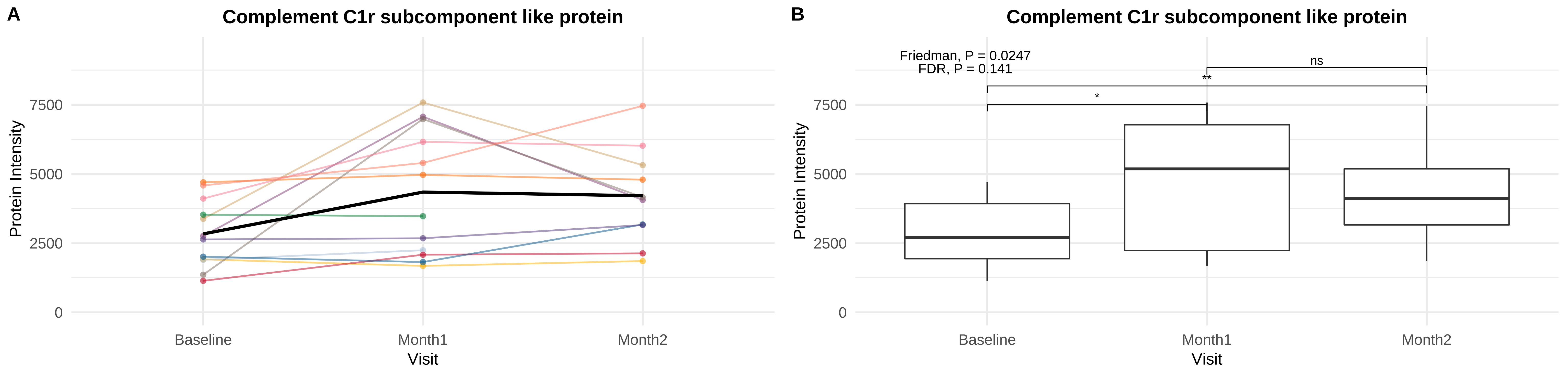

Supplementary Figure S 65

A) Line plot illustrating individual patient trajectories of Complement C1r subcomponent like protein intensity over time. The bold black line indicates the mean intensity over time. B) Box plots depicting the distribution of Complement C1r subcomponent like protein intensities at baseline, month 1, and month 2. Only AMD patients with measurements at all visits are included. The median, interquartile range, and outliers are displayed for each time point. Abbreviations: FDR, false discovery rate; ns, non−significant; * p < 0.05; ** p < 0.01; *** p < 0.001.

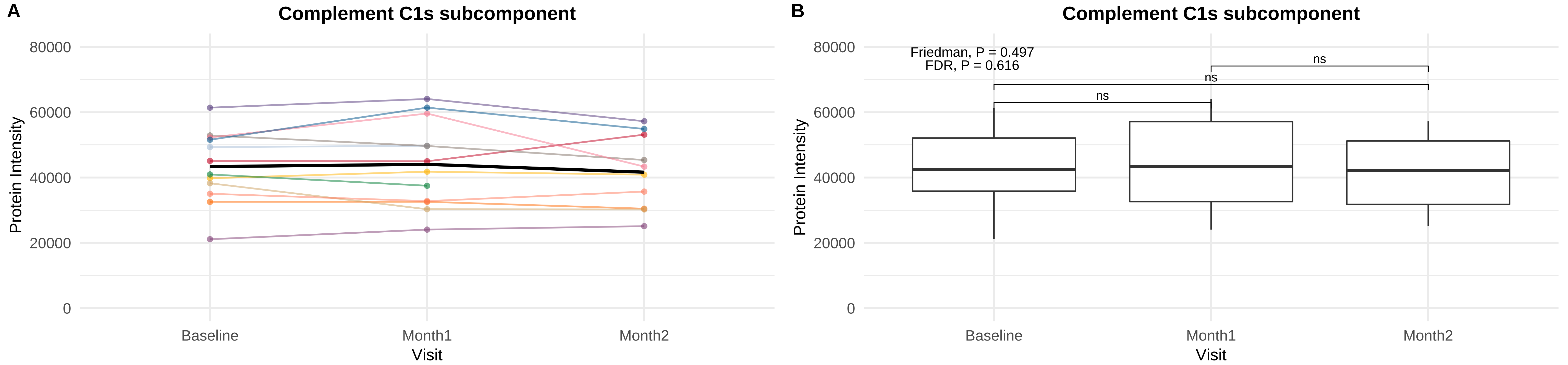

Supplementary Figure S 66

A) Line plot illustrating individual patient trajectories of Complement C1s subcomponent intensity over time. The bold black line indicates the mean intensity over time. B) Box plots depicting the distribution of Complement C1s subcomponent intensities at baseline, month 1, and month 2. [illegible]
AMD patients with measurements at all visits are included. The median, interquartile range, and outliers are displayed for each time point. Abbreviations: FDR, false discovery rate; ns, non−significant; * p < 0.05; ** p < 0.01; *** p < 0.001.

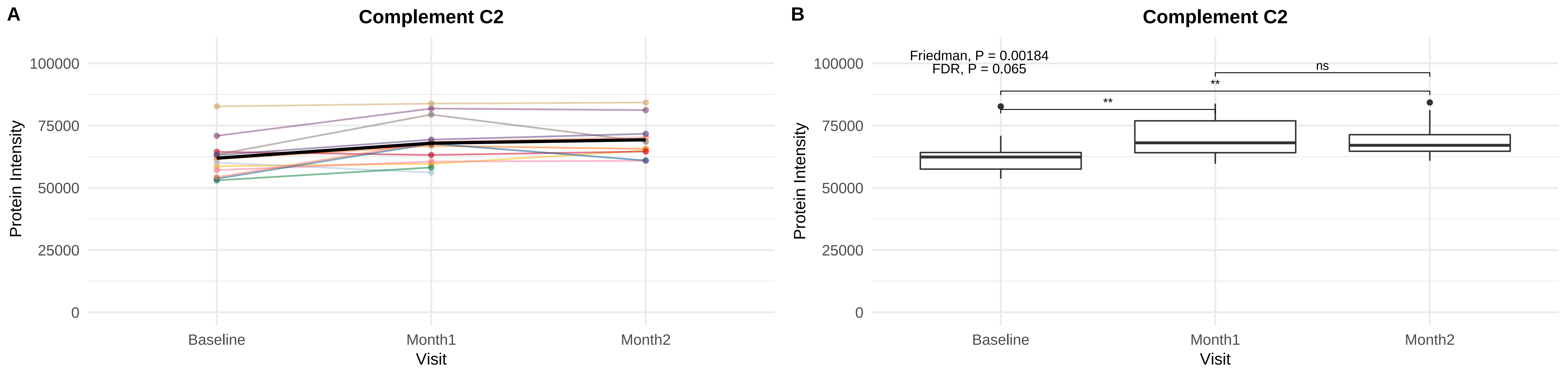

Supplementary Figure S 67

A) Line plot illustrating individual patient trajectories of Complement C2 intensity over time. The bold black line indicates the mean intensity over time. B) Box plots depicting the distribution of Complement C2 intensities at baseline, month 1, and month 2. Only AMD patients with measuren at all visits are included. The median, interquartile range, and outliers are displayed for each time point. Abbreviations: FDR, false discovery rate; ns, non−significant; * p < 0.05; ** p < 0.01; *** p < 0.001.

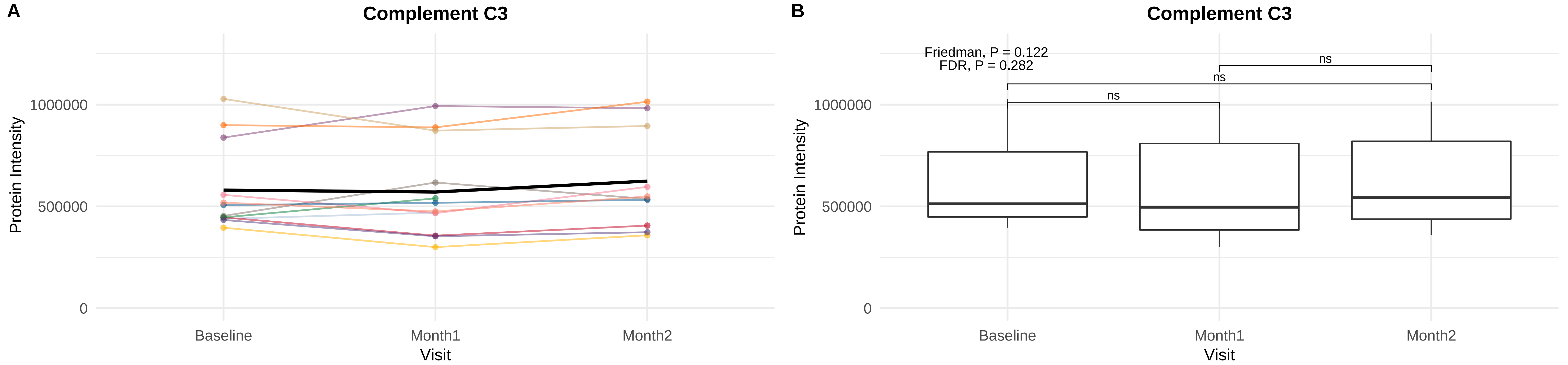

Supplementary Figure S 68

A) Line plot illustrating individual patient trajectories of Complement C3 intensity over time. The bold black line indicates the mean intensity over time. B) Box plots depicting the distribution of Complement C3 intensities at baseline, month 1, and month 2. Only AMD patients with measuren at all visits are included. The median, interquartile range, and outliers are displayed for each time point. Abbreviations: FDR, false discovery rate; ns, non−significant; * p < 0.05; ** p < 0.01; *** p < 0.001.

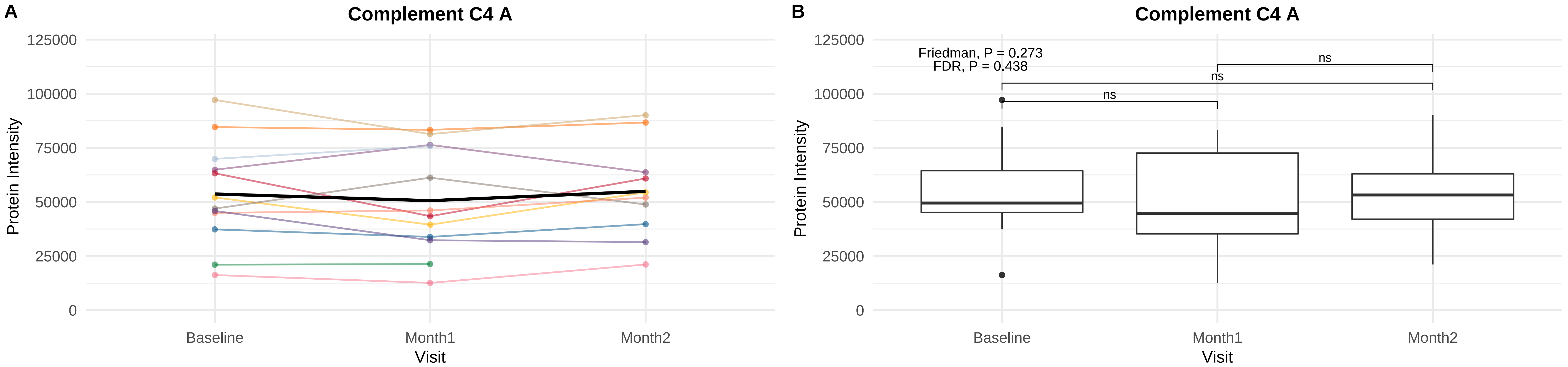

Supplementary Figure S 69

A) Line plot illustrating individual patient trajectories of Complement C4 A intensity over time. The bold black line indicates the mean intensity over time. B) Box plots depicting the distribution of Complement C4 A intensities at baseline, month 1, and month 2. Only AMD patients with measurements at all visits are included. The median, interquartile range, and outliers are displayed for each time point. Abbreviations: FDR, false discovery rate; ns, non−significant; * p < 0.05; ** p < 0.01; *** p < 0.001.

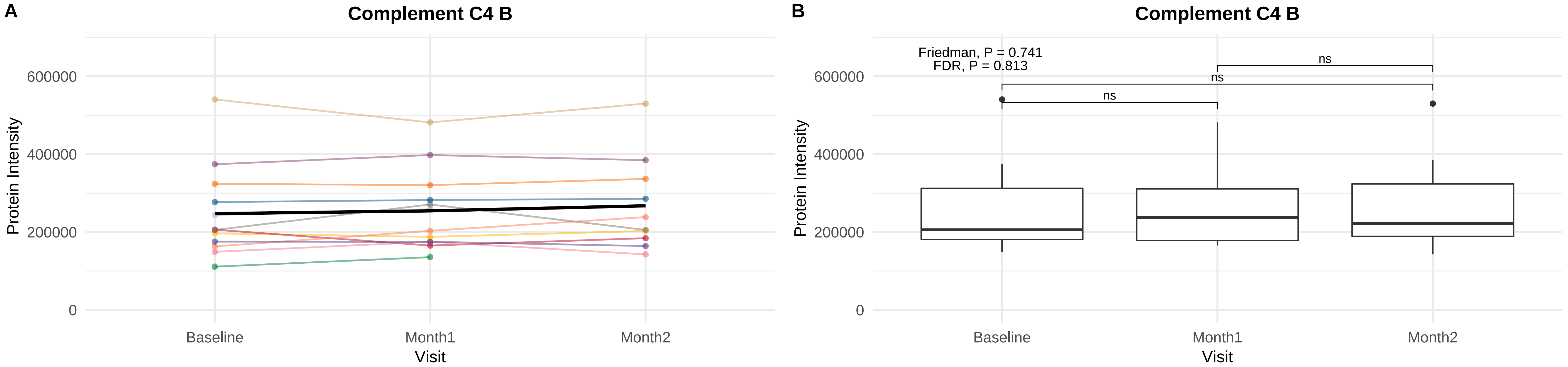

Supplementary Figure S 70

A) Line plot illustrating individual patient trajectories of Complement C4 B intensity over time. The bold black line indicates the mean intensity over time. B) Box plots depicting the distribution of Complement C4 B intensities at baseline, month 1, and month 2. Only AMD patients with measurements at all visits are included. The median, interquartile range, and outliers are displayed for each time point. Abbreviations: FDR, false discovery rate; ns, non−significant; * p < 0.05; ** p < 0.01; *** p < 0.001.

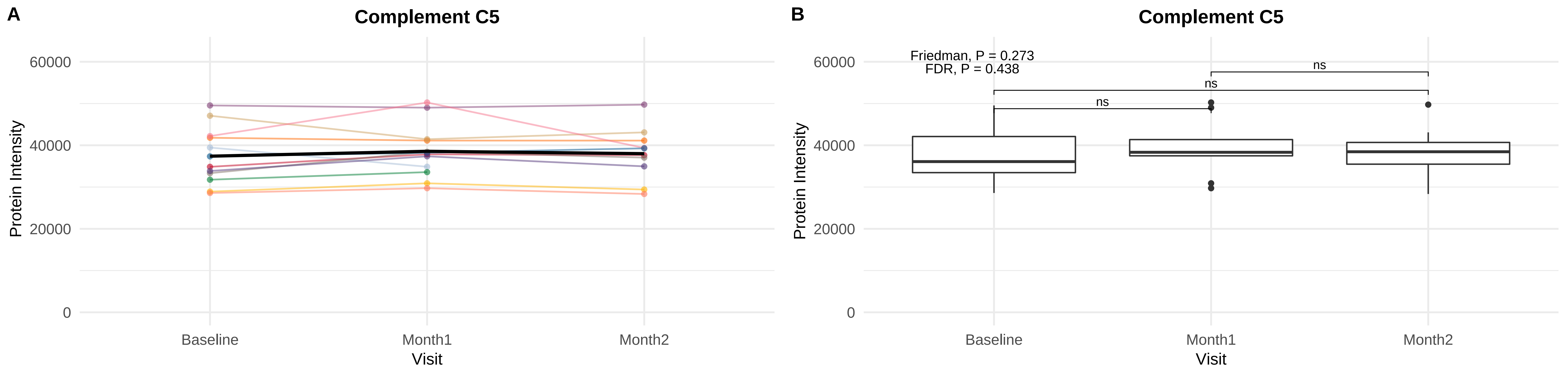

Supplementary Figure S 71

A) Line plot illustrating individual patient trajectories of Complement C5 intensity over time. The bold black line indicates the mean intensity over time. B) Box plots depicting the distribution of Complement C5 intensities at baseline, month 1, and month 2. Only AMD patients with measuren at all visits are included. The median, interquartile range, and outliers are displayed for each time point. Abbreviations: FDR, false discovery rate; ns, non−significant; * p < 0.05; ** p < 0.01; *** p < 0.001.

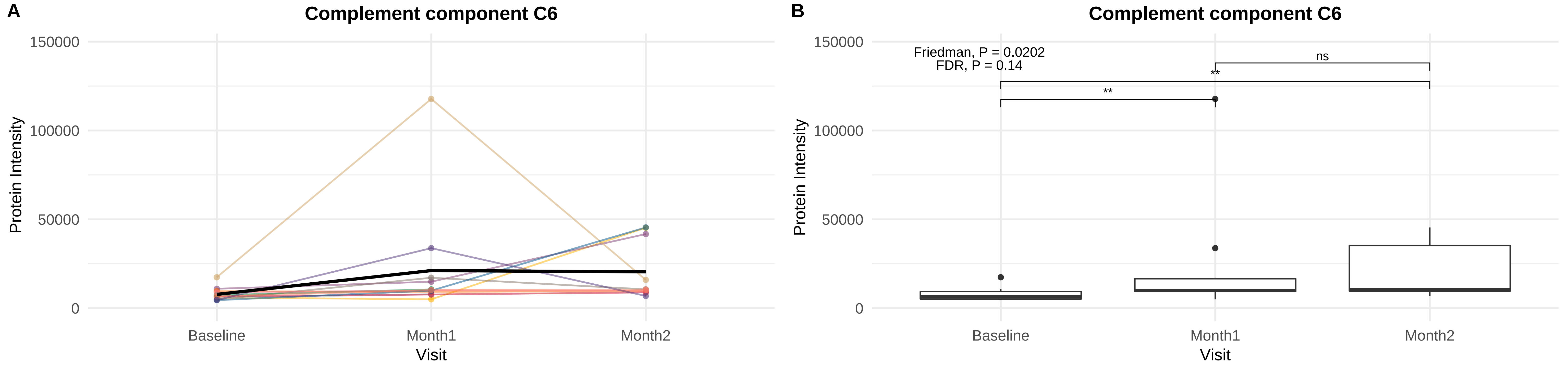

Supplementary Figure S 72

A) Line plot illustrating individual patient trajectories of Complement component C6 intensity over time. The bold black line indicates the mean intensity over time. B) Box plots depicting the distribution of Complement component C6 intensities at baseline, month 1, and month 2. Only AMD patients with measurements at all visits are included. The median, interquartile range, and outliers are displayed for each time point. Abbreviations: FDR, false discovery rate; ns, non−significant; * p < 0.05; ** p < 0.01; *** p < 0.001.

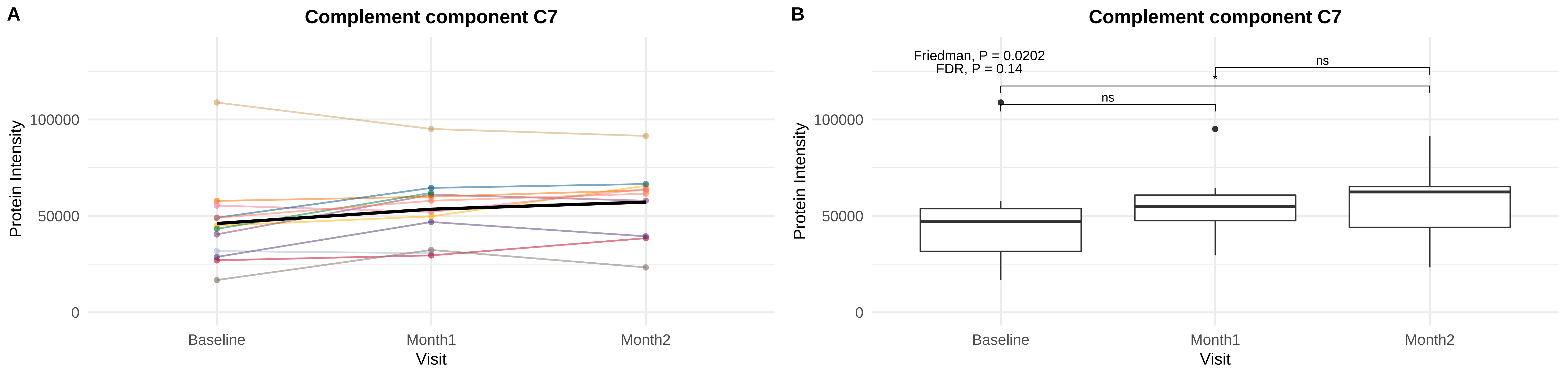

Supplementary Figure S 73

A) Line plot illustrating individual patient trajectories of Complement component C7 intensity over time. The bold black line indicates the mean intensity over time. B) Box plots depicting the distribution of Complement component C7 intensities at baseline, month 1, and month 2. Only AMD patients with measurements at all visits are included. The median, interquartile range, and outliers are displayed for each time point. Abbreviations: FDR, false discovery rate; ns, non−significant; * p < 0.05; ** p < 0.01; *** p < 0.001.

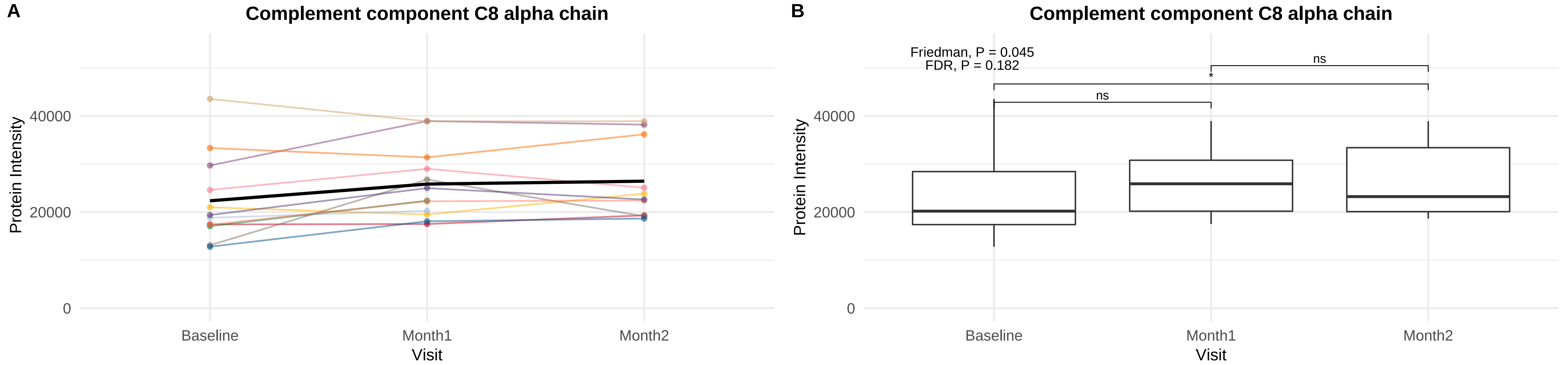

Supplementary Figure S 74

A) Line plot illustrating individual patient trajectories of Complement component C8 alpha chain intensity over time. The bold black line indicates the mean intensity over time. B) Box plots depicting the distribution of Complement component C8 alpha chain intensities at baseline, month 1, month 2. Only AMD patients with measurements at all visits are included. The median, interquartile range, and outliers are displayed for each time point. Abbreviations: FDR, false discovery rate; ns, non−significant; * p < 0.05; ** p < 0.01; *** p < 0.001.

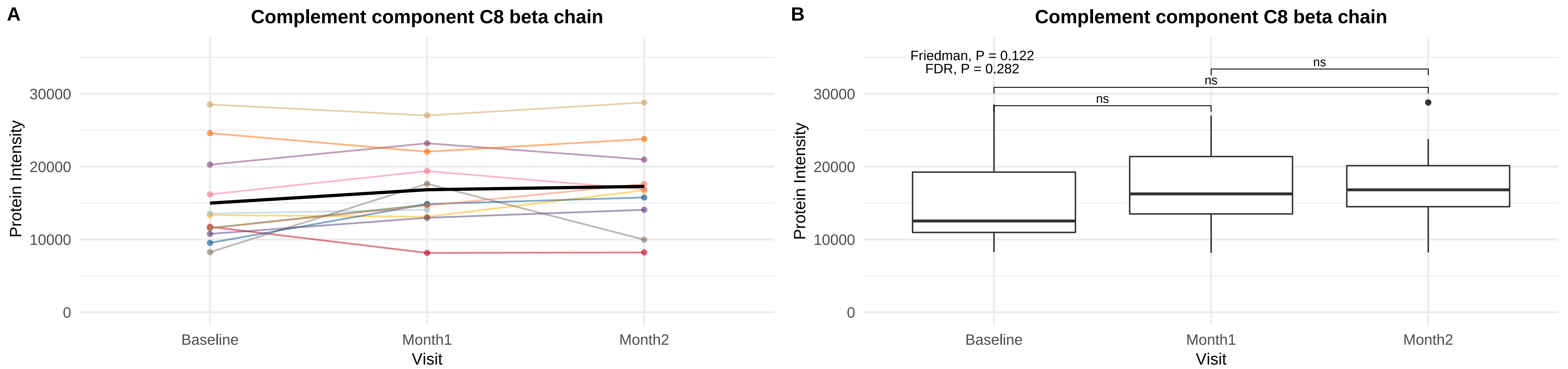

Supplementary Figure S 75

A) Line plot illustrating individual patient trajectories of Complement component C8 beta chain intensity over time. The bold black line indicates the mean intensity over time. B) Box plots depicting the distribution of Complement component C8 beta chain intensities at baseline, month 1, ar month 2. Only AMD patients with measurements at all visits are included. The median, interquartile range, and outliers are displayed for each time point. Abbreviations: FDR, false discovery rate; ns, non−significant; * p < 0.05; ** p < 0.01; *** p < 0.001.

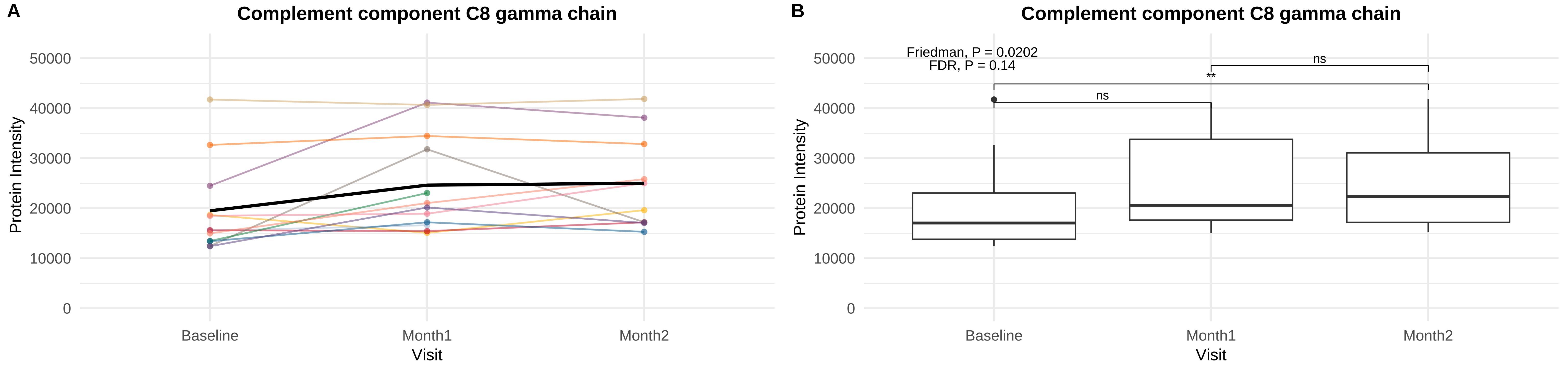

Supplementary Figure S 76

A) Line plot illustrating individual patient trajectories of Complement component C8 gamma chain intensity over time. The bold black line indicates the mean intensity over time. B) Box plots depicting the distribution of Complement component C8 gamma chain intensities at baseline, mont month 2. Only AMD patients with measurements at all visits are included. The median, interquartile range, and outliers are displayed for each time point. Abbreviations: FDR, false discovery rate; ns, non−significant; * p < 0.05; ** p < 0.01; *** p < 0.001.

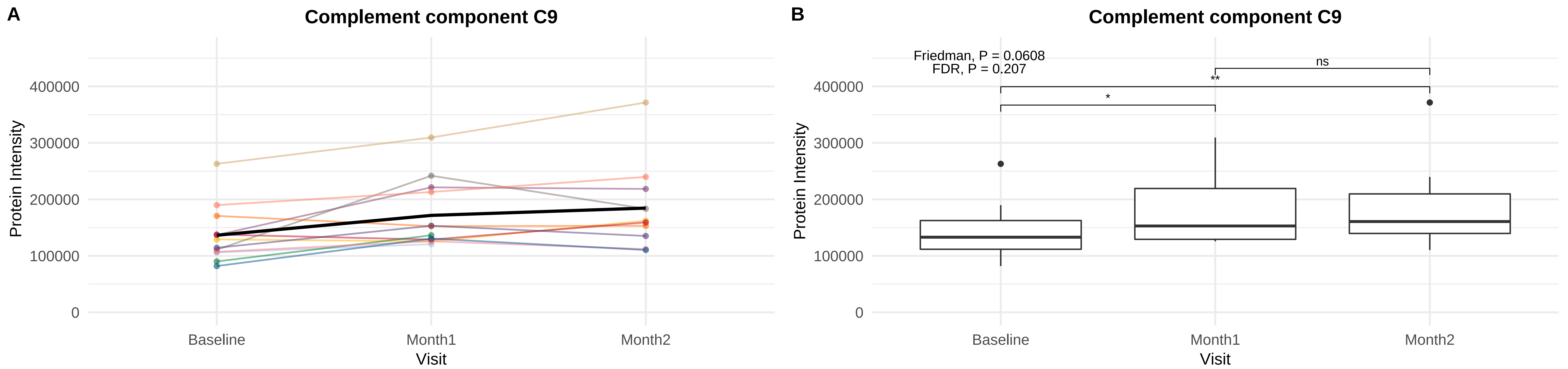

Supplementary Figure S 77

A) Line plot illustrating individual patient trajectories of Complement component C9 intensity over time. The bold black line indicates the mean intensity over time. B) Box plots depicting the distribution of Complement component C9 intensities at baseline, month 1, and month 2. Only AMD patients with measurements at all visits are included. The median, interquartile range, and outliers are displayed for each time point. Abbreviations: FDR, false discovery rate; ns, non−significant; * p < 0.05; ** p < 0.01; *** p < 0.001.

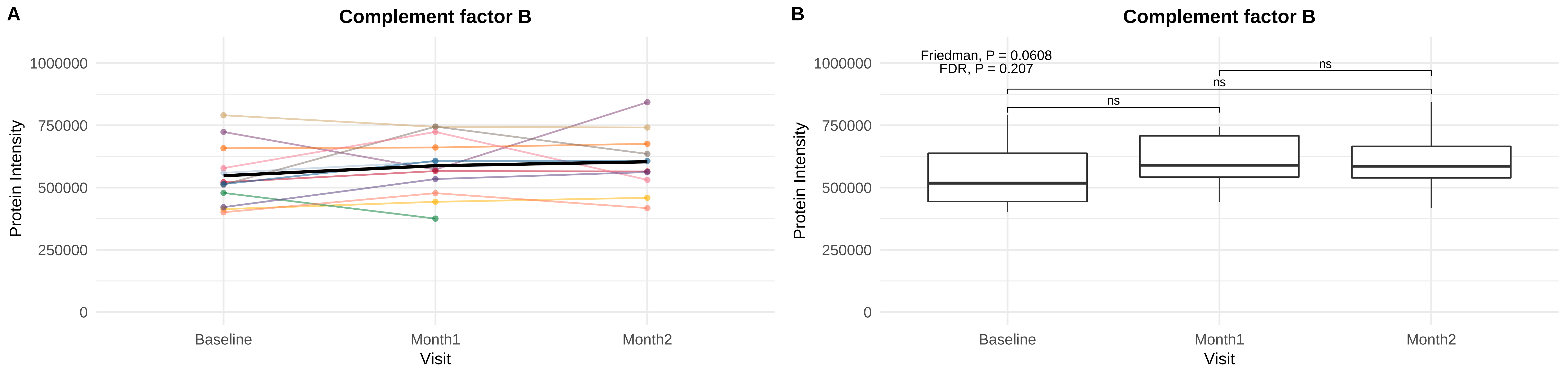

Supplementary Figure S 78

A) Line plot illustrating individual patient trajectories of Complement factor B intensity over time. The bold black line indicates the mean intensity over time. B) Box plots depicting the distribution of Complement factor B intensities at baseline, month 1, and month 2. Only AMD patients with measurements at all visits are included. The median, interquartile range, and outliers are displayed for each time point. Abbreviations: FDR, false discovery rate; ns, non−significant; * p < 0.05; ** p < 0.01; *** p < 0.001.

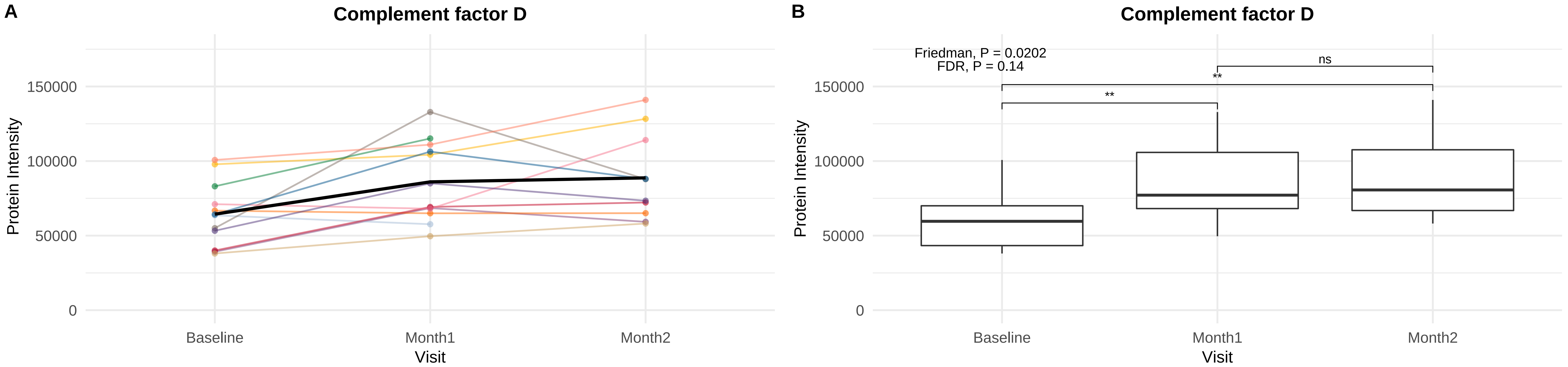

Supplementary Figure S 79

A) Line plot illustrating individual patient trajectories of Complement factor D intensity over time. The bold black line indicates the mean intensity over time. B) Box plots depicting the distribution of Complement factor D intensities at baseline, month 1, and month 2. Only AMD patients with measurements at all visits are included. The median, interquartile range, and outliers are displayed for each time point. Abbreviations: FDR, false discovery rate; ns, non−significant; * p < 0.05; ** p < 0.01; *** p < 0.001.

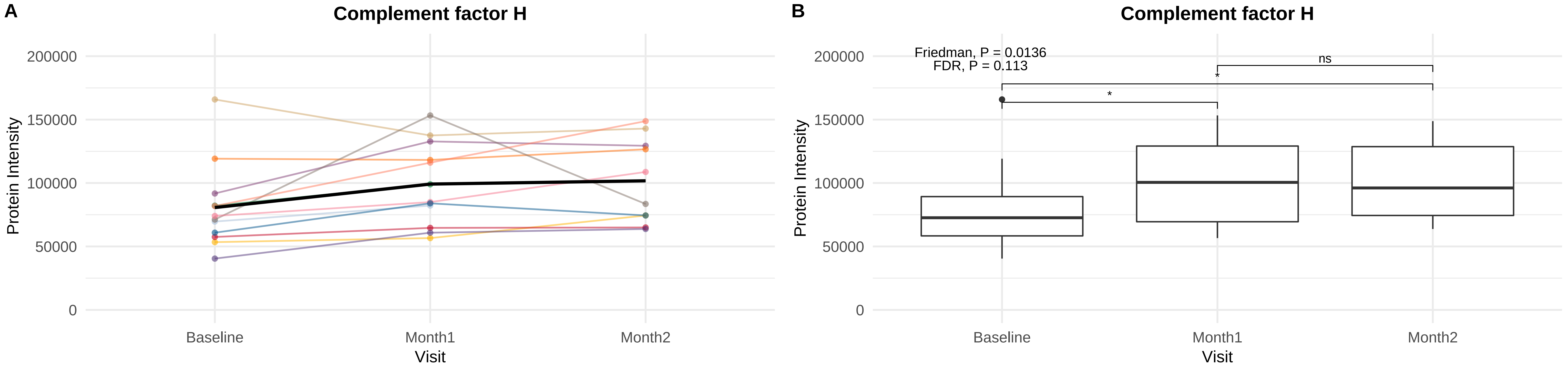

Supplementary Figure S 80

A) Line plot illustrating individual patient trajectories of Complement factor H intensity over time. The bold black line indicates the mean intensity over time. B) Box plots depicting the distribution of Complement factor H intensities at baseline, month 1, and month 2. Only AMD patients with measurements at all visits are included. The median, interquartile range, and outliers are displayed for each time point. Abbreviations: FDR, false discovery rate; ns, non−significant; * p < 0.05; ** p < 0.01; *** p < 0.001.

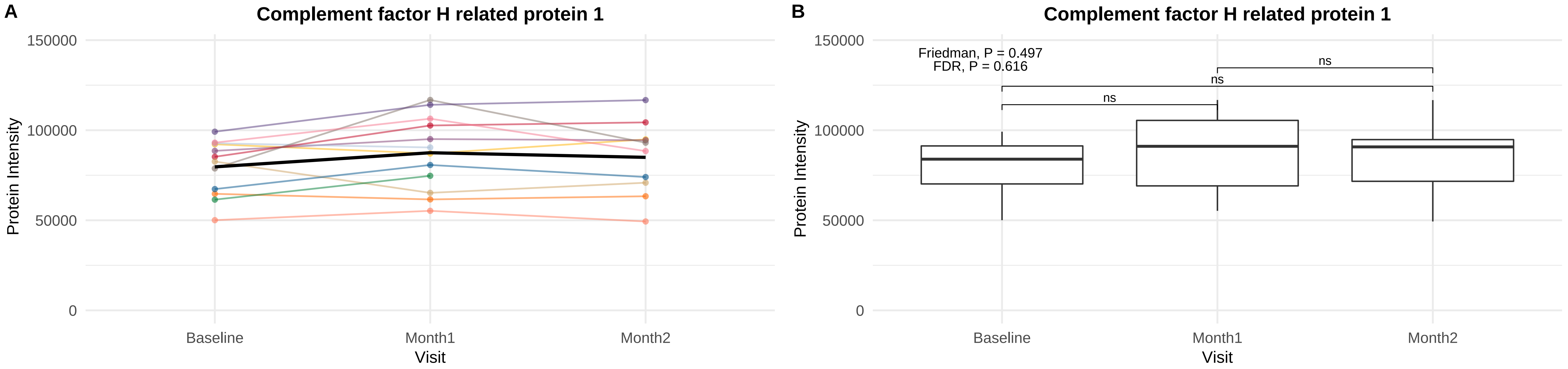

Supplementary Figure S 81

A) Line plot illustrating individual patient trajectories of Complement factor H related protein 1 intensity over time. The bold black line indicates the mean intensity over time. B) Box plots depicting the distribution of Complement factor H related protein 1 intensities at baseline, month 1, and month 2. Only AMD patients with measurements at all visits are included. The median, interquartile range, and outliers are displayed for each time point. Abbreviations: FDR, false discovery rate; ns, non−significant; * p < 0.05; ** p < 0.01; *** p < 0.001.

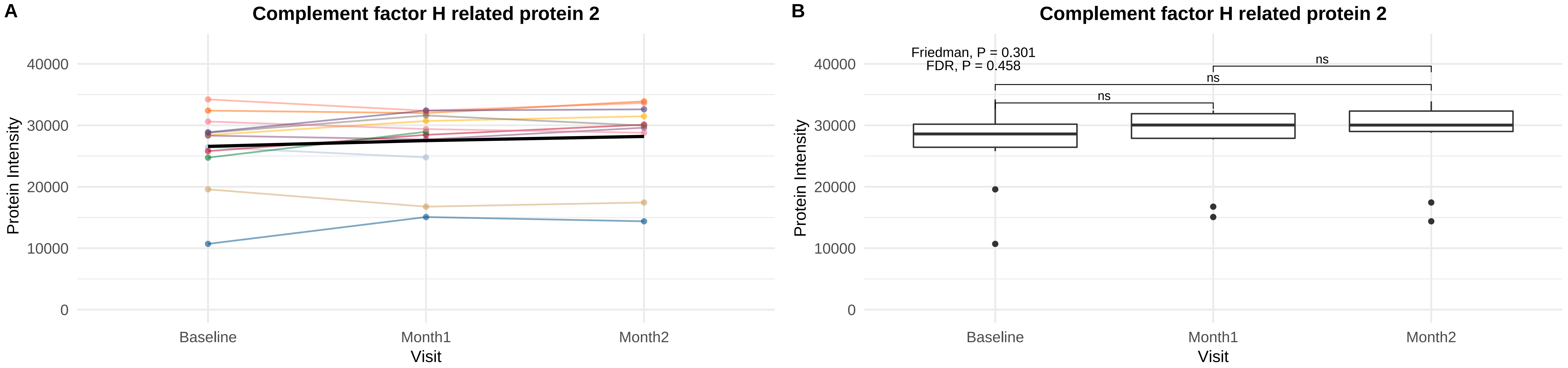

Supplementary Figure S 82

A) Line plot illustrating individual patient trajectories of Complement factor H related protein 2 intensity over time. The bold black line indicates the mean intensity over time. B) Box plots depicting the distribution of Complement factor H related protein 2 intensities at baseline, month 1, and month 2. Only AMD patients with measurements at all visits are included. The median, interquartile range, and outliers are displayed for each time point. Abbreviations: FDR, false discovery rate; ns, non−significant; * p < 0.05; ** p < 0.01; *** p < 0.001.

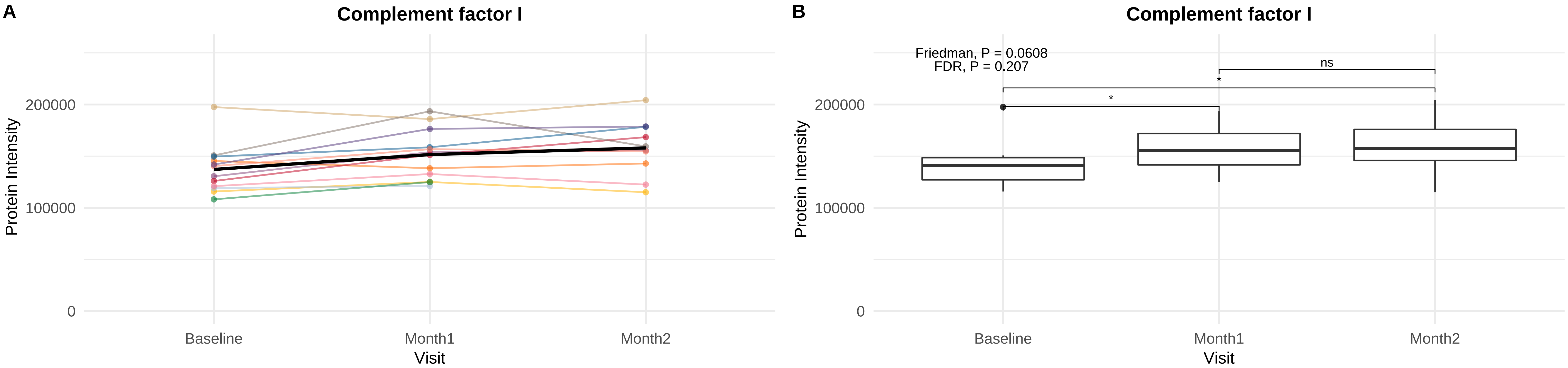

Supplementary Figure S 83

A) Line plot illustrating individual patient trajectories of Complement factor I intensity over time. The bold black line indicates the mean intensity over time. B) Box plots depicting the distribution of Complement factor I intensities at baseline, month 1, and month 2. Only AMD patients with measurements at all visits are included. The median, interquartile range, and outliers are displayed for each time point. Abbreviations: FDR, false discovery rate; ns, non−significant; * p < 0.05; ** p < 0.01; *** p < 0.001.

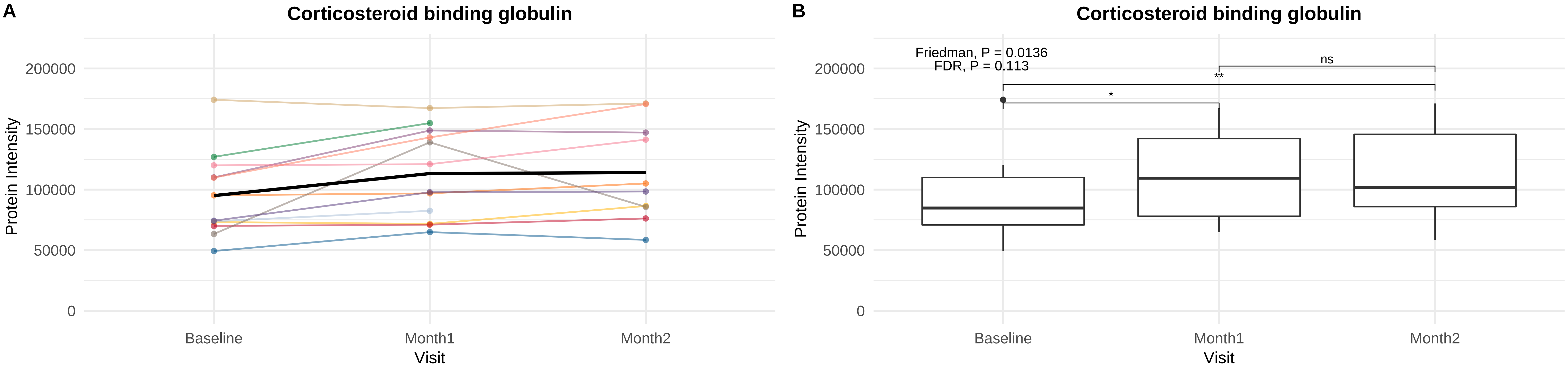

Supplementary Figure S 84

A) Line plot illustrating individual patient trajectories of Corticosteroid binding globulin intensity over time. The bold black line indicates the mean intensity over time. B) Box plots depicting the distribution of Corticosteroid binding globulin intensities at baseline, month 1, and month 2. Only AMD patients with measurements at all visits are included. The median, interquartile range, and outliers are displayed for each time point. Abbreviations: FDR, false discovery rate; ns, non−significant; * p < 0.05; ** p < 0.01; *** p < 0.001.

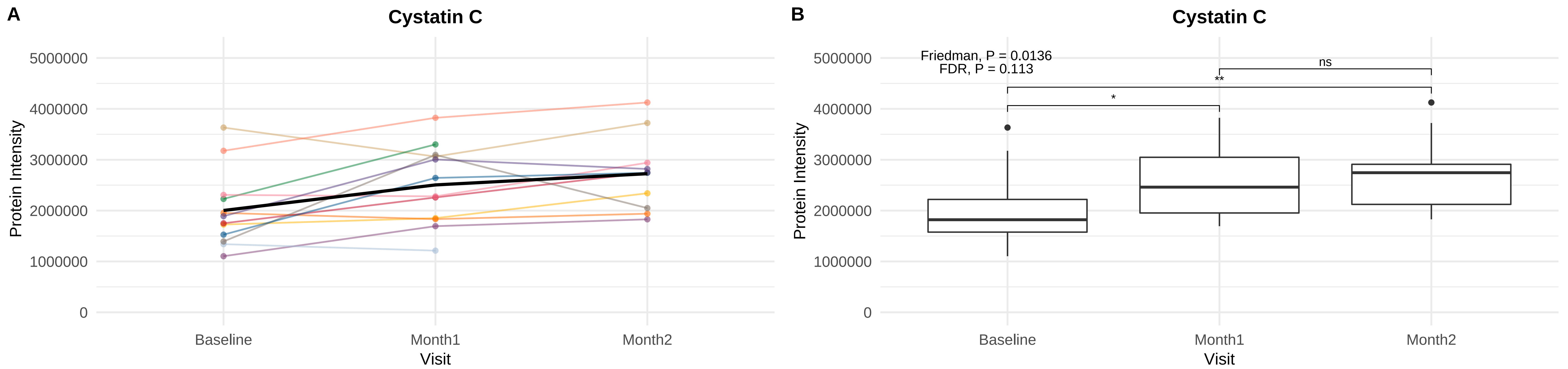

Supplementary Figure S 85

A) Line plot illustrating individual patient trajectories of Cystatin C intensity over time. The bold black line indicates the mean intensity over time. B) Box plots depicting the distribution of Cystatin C intensities at baseline, month 1, and month 2. Only AMD patients with measurements at all visits are included. The median, interquartile range, and outliers are displayed for each time point. Abbreviations: FDR, false discovery rate; ns, non−significant; * p < 0.05; ** p < 0.01; *** p < 0.001.

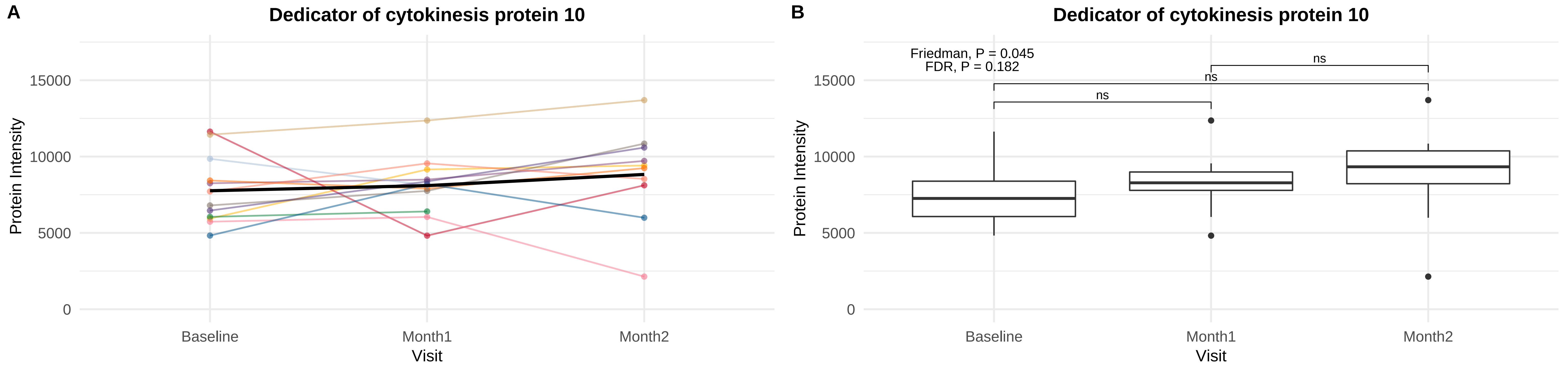

Supplementary Figure S 86

A) Line plot illustrating individual patient trajectories of Dedicator of cytokinesis protein 10 intensity over time. The bold black line indicates the mean intensity over time. B) Box plots depicting the distribution of Dedicator of cytokinesis protein 10 intensities at baseline, month 1, and month 2. Only AMD patients with measurements at all visits are included. The median, interquartile range, and outliers are displayed for each time point. Abbreviations: FDR, false discovery rate; ns, non−significant; * p < 0.05; ** p < 0.01; *** p < 0.001.

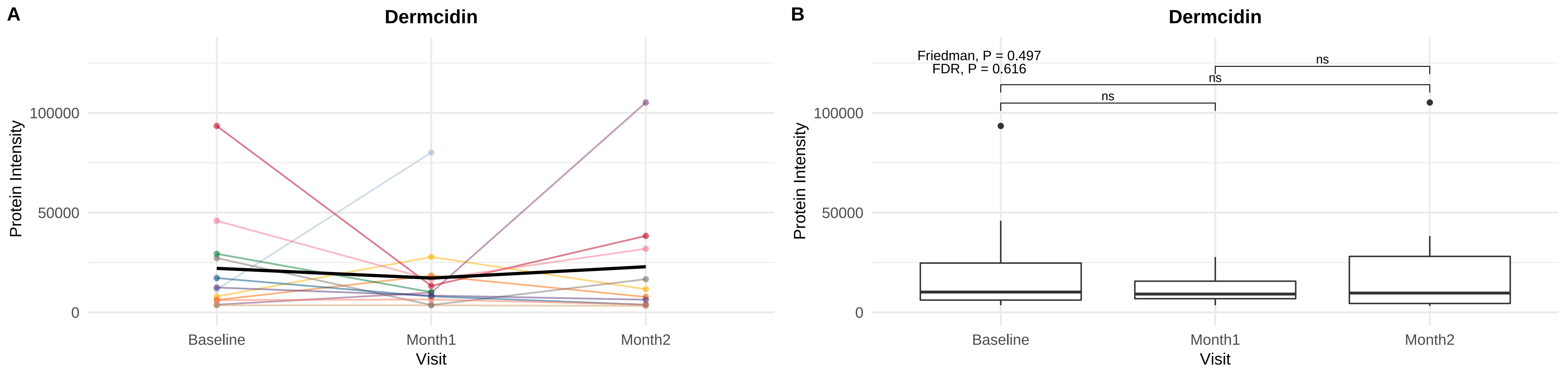

Supplementary Figure S 87

A) Line plot illustrating individual patient trajectories of Dermcidin intensity over time. The bold black line indicates the mean intensity over time. B) Box plots depicting the distribution of Dermcidin intensities at baseline, month 1, and month 2. Only AMD patients with measurements at all visits are included. The median, interquartile range, and outliers are displayed for each time point. Abbreviations: FDR, false discovery rate; ns, non−significant; * p < 0.05; ** p < 0.01; *** p < 0.001.

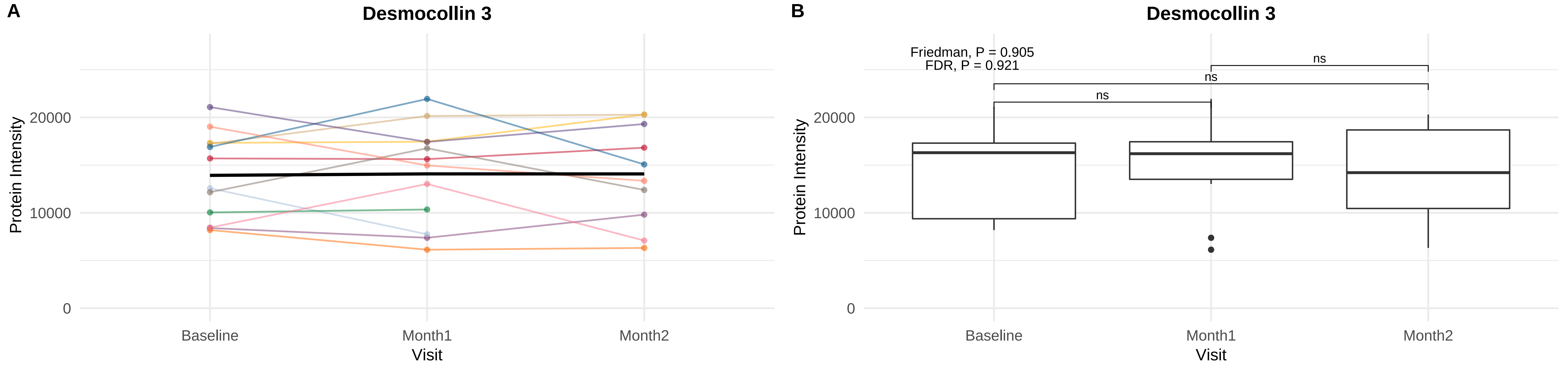

Supplementary Figure S 88

A) Line plot illustrating individual patient trajectories of Desmocollin 3 intensity over time. The bold black line indicates the mean intensity over time. B) Box plots depicting the distribution of Desmocollin 3 intensities at baseline, month 1, and month 2. Only AMD patients with measurements at all visits are included. The median, interquartile range, and outliers are displayed for each time point. Abbreviations: FDR, false discovery rate; ns, non−significant; * p < 0.05; ** p < 0.01; *** p < 0.001.

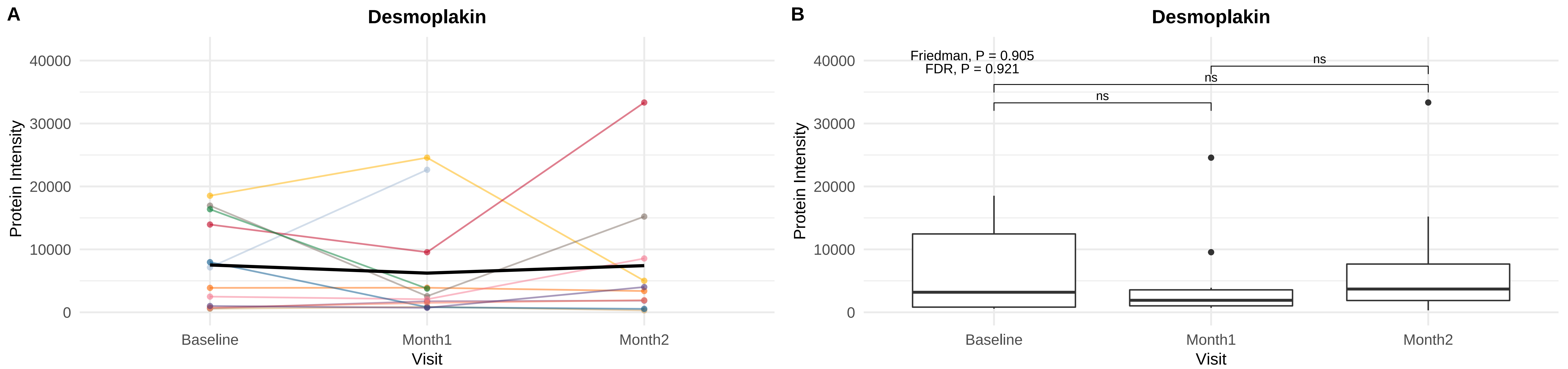

Supplementary Figure S 89

A) Line plot illustrating individual patient trajectories of Desmoplakin intensity over time. The bold black line indicates the mean intensity over time. B) Box plots depicting the distribution of Desmoplakin intensities at baseline, month 1, and month 2. Only AMD patients with measurements a all visits are included. The median, interquartile range, and outliers are displayed for each time point. Abbreviations: FDR, false discovery rate; ns, non−significant; * p < 0.05; ** p < 0.01; *** p < 0.001.

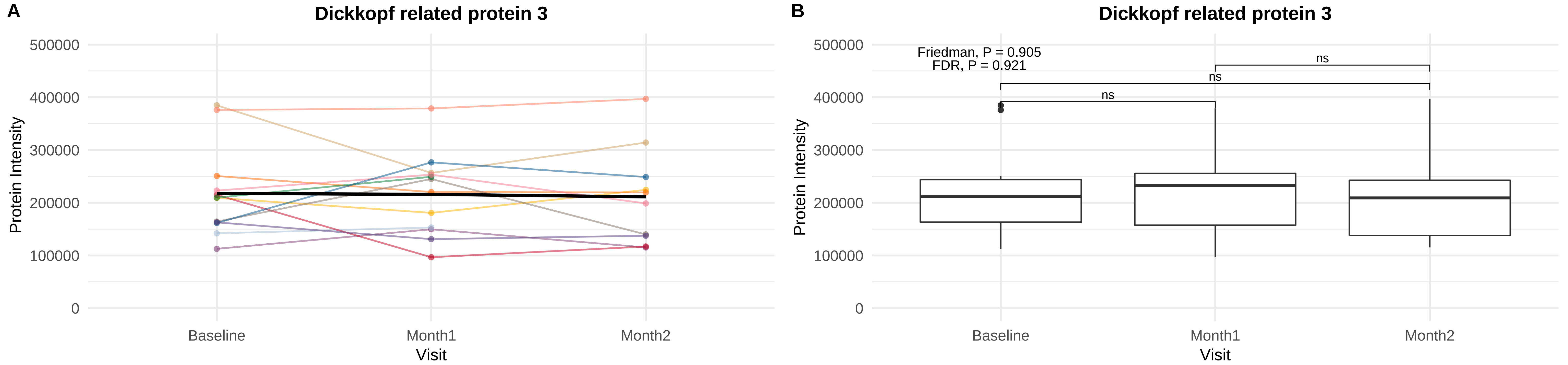

Supplementary Figure S 90

A) Line plot illustrating individual patient trajectories of Dickkopf related protein 3 intensity over time. The bold black line indicates the mean intensity over time. B) Box plots depicting the distribution of Dickkopf related protein 3 intensities at baseline, month 1, and month 2. Only AMD patients with measurements at all visits are included. The median, interquartile range, and outliers are displayed for each time point. Abbreviations: FDR, false discovery rate; ns, non−significant; * p < 0.05; ** p < 0.01; *** p < 0.001.

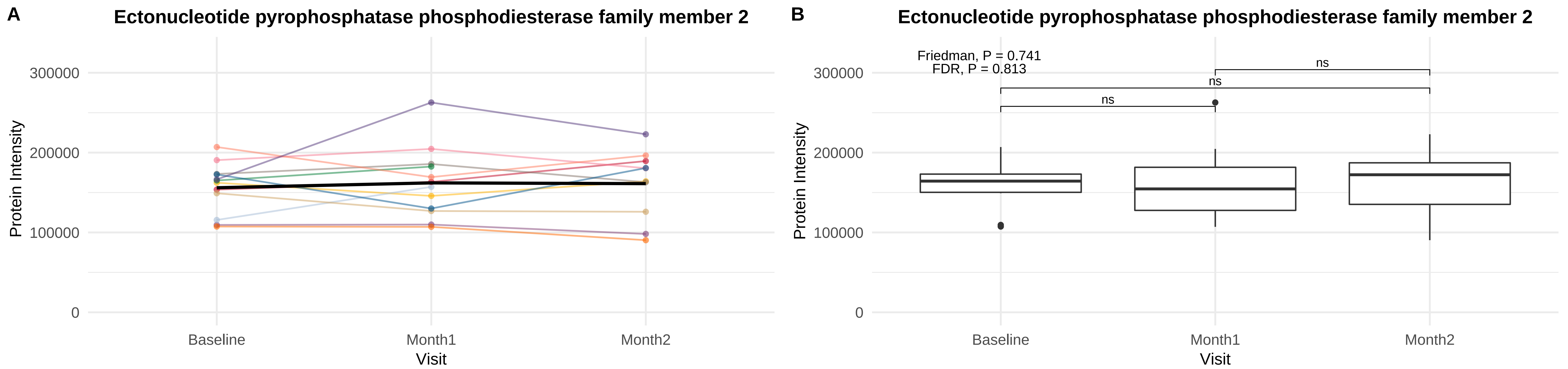

Supplementary Figure S 91

A) Line plot illustrating individual patient trajectories of Ectonucleotide pyrophosphatase phosphodiesterase family member 2 intensity over time. The bold black line indicates the mean intensity over time. B) Box plots depicting the distribution of Ectonucleotide pyrophosphatase phosphodiesterase family member 2 intensities at baseline, month 1, and month 2. Only AMD patients with measurements at all visits are included. The median, interquartile range, and outliers are displayed for each time point. Abbreviations: FDR, false discovery rate; ns, non−significant 0.05; ** p < 0.01; *** p < 0.001.

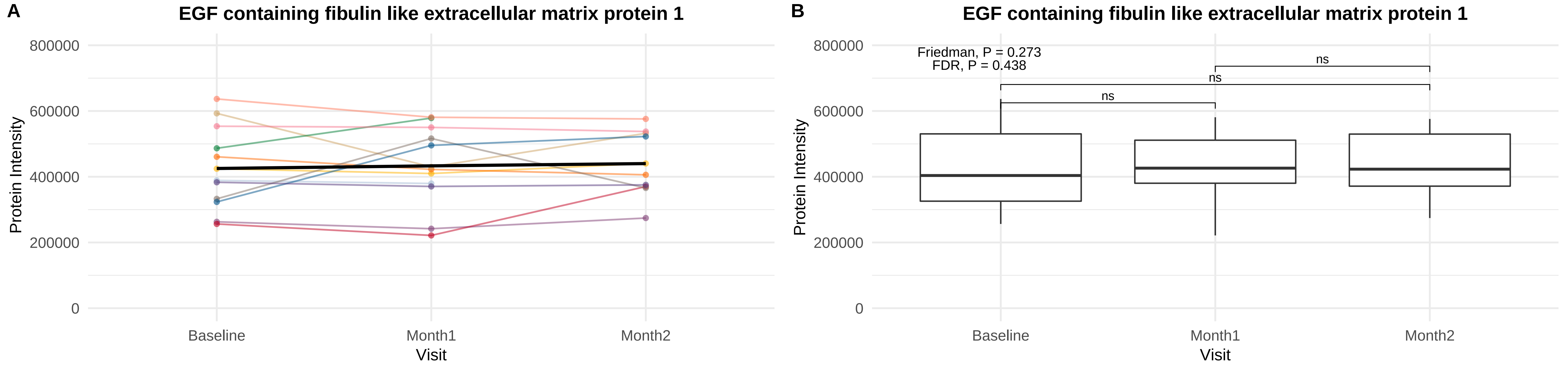

Supplementary Figure S 92

A) Line plot illustrating individual patient trajectories of EGF containing fibulin like extracellular matrix protein 1 intensity over time. The bold black line indicates the mean intensity over time. B) Box plots depicting the distribution of EGF containing fibulin like extracellular matrix protein 1 intensities at baseline, month 1, and month 2. Only AMD patients with measurements at all visits are included. The median, interquartile range, and outliers are displayed for each time point. Abbreviations: FDR, false discovery rate; ns, non−significant; * p < 0.05; ** p < 0.01; *** p < 0.001.

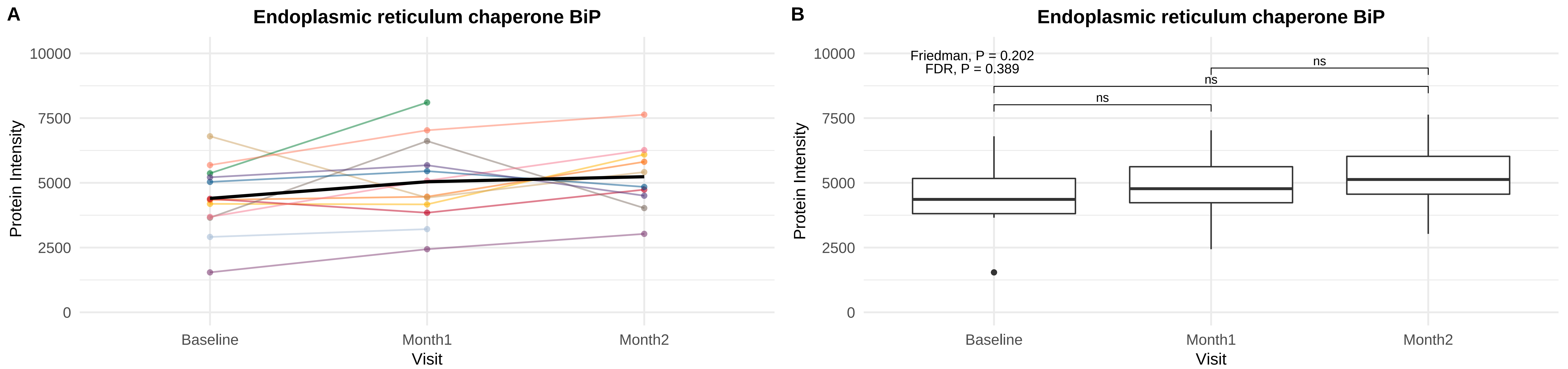

Supplementary Figure S 93

A) Line plot illustrating individual patient trajectories of Endoplasmic reticulum chaperone BiP intensity over time. The bold black line indicates the mean intensity over time. B) Box plots depicting the distribution of Endoplasmic reticulum chaperone BiP intensities at baseline, month 1, and month 2. Only AMD patients with measurements at all visits are included. The median, interquartile range, and outliers are displayed for each time point. Abbreviations: FDR, false discovery rate; ns, non−significant; * p < 0.05; ** p < 0.01; *** p < 0.001.

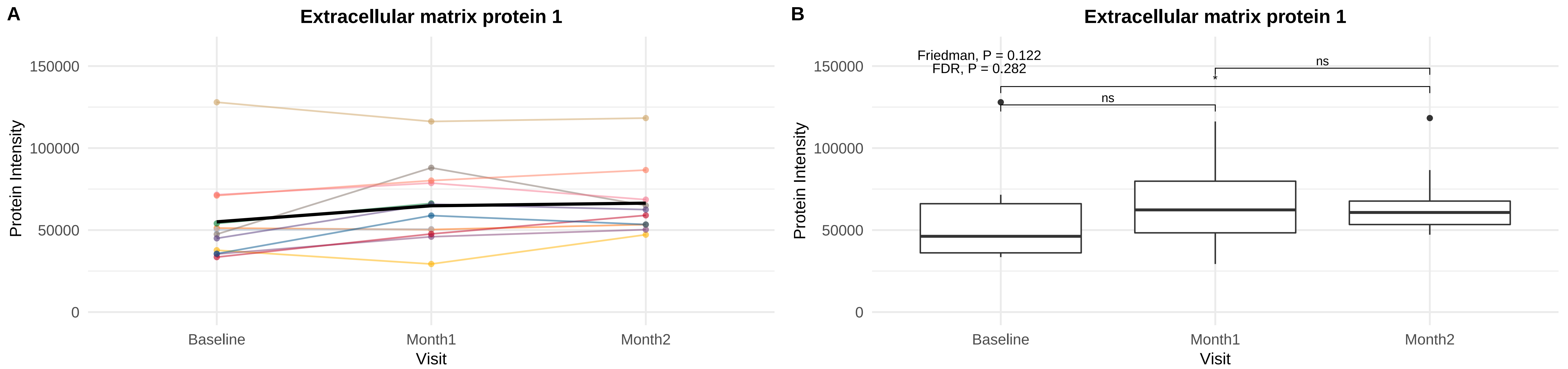

Supplementary Figure S 94

A) Line plot illustrating individual patient trajectories of Extracellular matrix protein 1 intensity over time. The bold black line indicates the mean intensity over time. B) Box plots depicting the distribution of Extracellular matrix protein 1 intensities at baseline, month 1, and month 2. Only AMD patients with measurements at all visits are included. The median, interquartile range, and outliers are displayed for each time point. Abbreviations: FDR, false discovery rate; ns, non−significant; * p < 0.05; ** p < 0.01; *** p < 0.001.

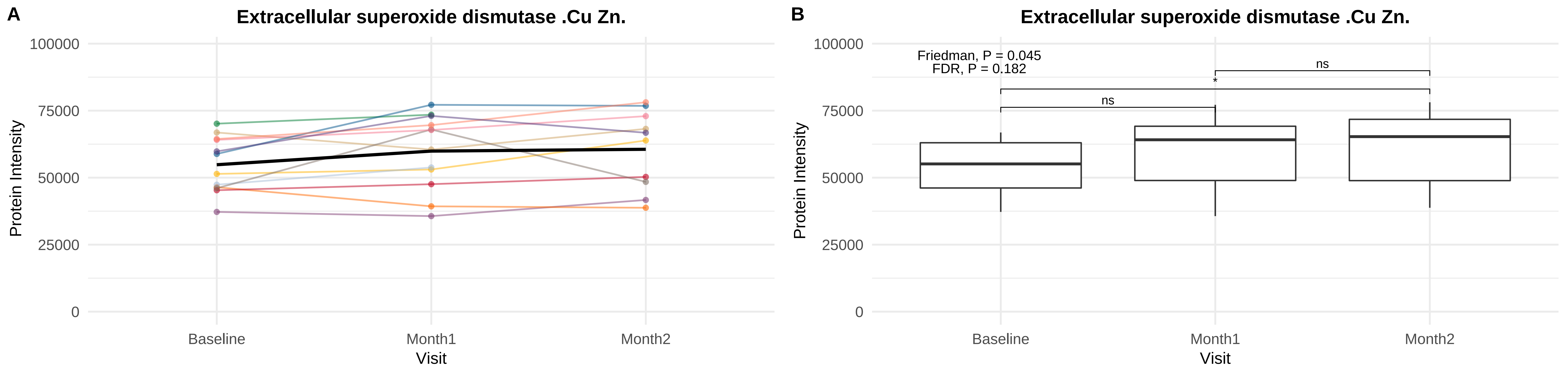

Supplementary Figure S 95

A) Line plot illustrating individual patient trajectories of Extracellular superoxide dismutase .Cu Zn. intensity over time. The bold black line indicates the mean intensity over time. B) Box plots depicting the distribution of Extracellular superoxide dismutase .Cu Zn. intensities at baseline, month 1, and month 2. Only AMD patients with measurements at all visits are included. The median, interquartile range, and outliers are displayed for each time point. Abbreviations: FDR, false discovery rate; ns, non−significant; * p < 0.05; ** p < 0.01; *** p < 0.001.

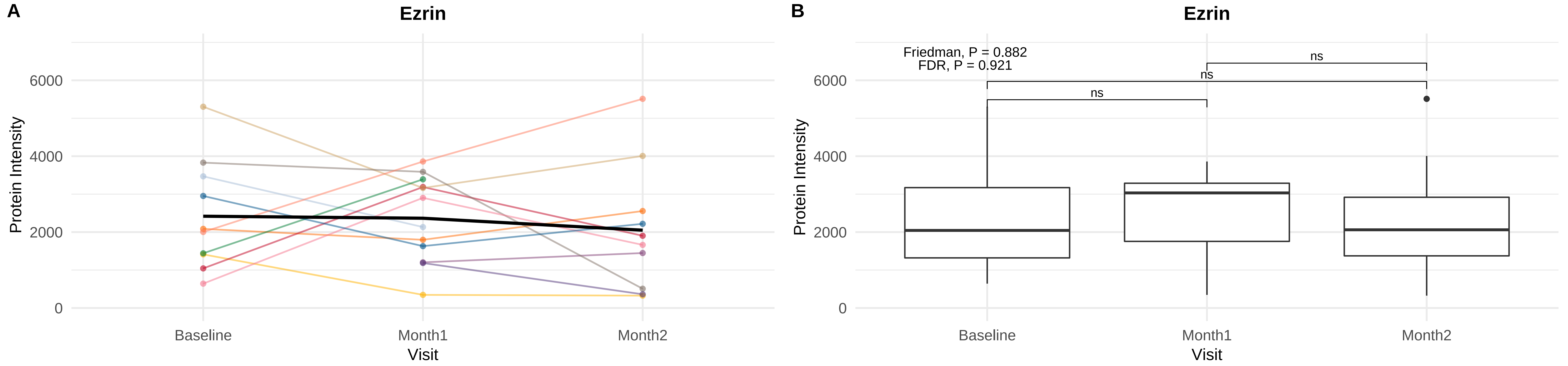

Supplementary Figure S 96

A) Line plot illustrating individual patient trajectories of Ezrin intensity over time. The bold black line indicates the mean intensity over time. B) Box plots depicting the distribution of Ezrin intensities at baseline, month 1, and month 2. Only AMD patients with measurements at all visits are included. The median, interquartile range, and outliers are displayed for each time point. Abbreviations: FDR, false discovery rate; ns, non−significant; * p < 0.05; ** p < 0.01; *** p < 0.001.

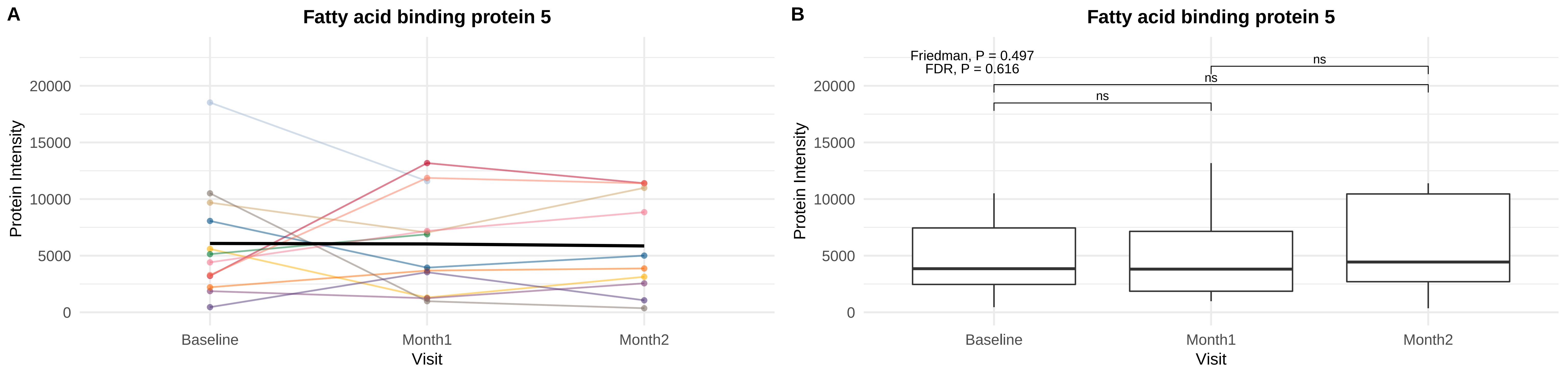

Supplementary Figure S 97

A) Line plot illustrating individual patient trajectories of Fatty acid binding protein 5 intensity over time. The bold black line indicates the mean intensity over time. B) Box plots depicting the distribution of Fatty acid binding protein 5 intensities at baseline, month 1, and month 2. Only AMD patients with measurements at all visits are included. The median, interquartile range, and outliers are displayed for each time point. Abbreviations: FDR, false discovery rate; ns, non−significant; * p < 0.05; ** p < 0.01; *** p < 0.001.

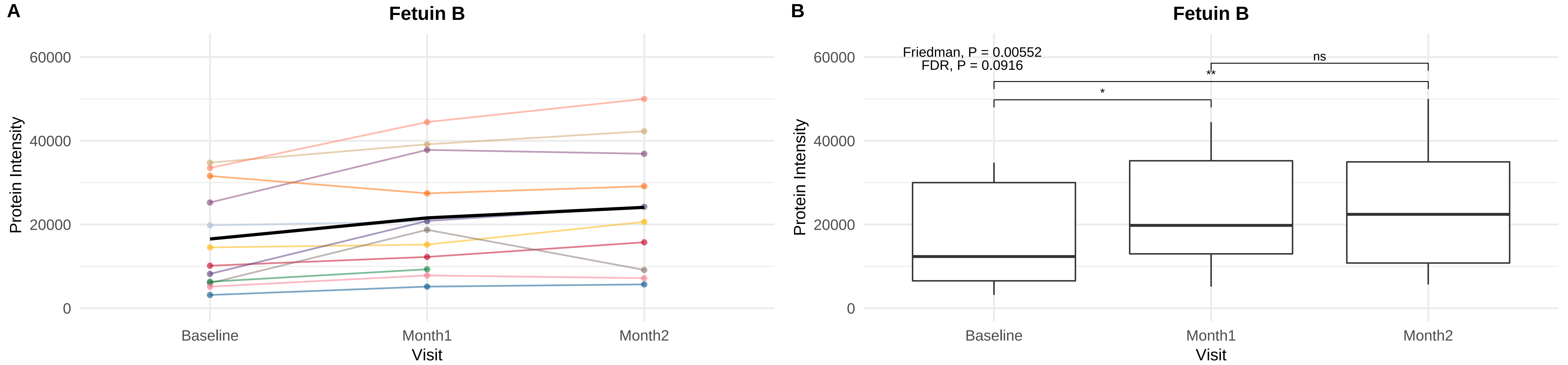

Supplementary Figure S 98

A) Line plot illustrating individual patient trajectories of Fetuin B intensity over time. The bold black line indicates the mean intensity over time. B) Box plots depicting the distribution of Fetuin B intensities at baseline, month 1, and month 2. Only AMD patients with measurements at all visits are included. The median, interquartile range, and outliers are displayed for each time point. Abbreviations: FDR, false discovery rate; ns, non−significant; * p < 0.05; ** p < 0.01; *** p < 0.001.

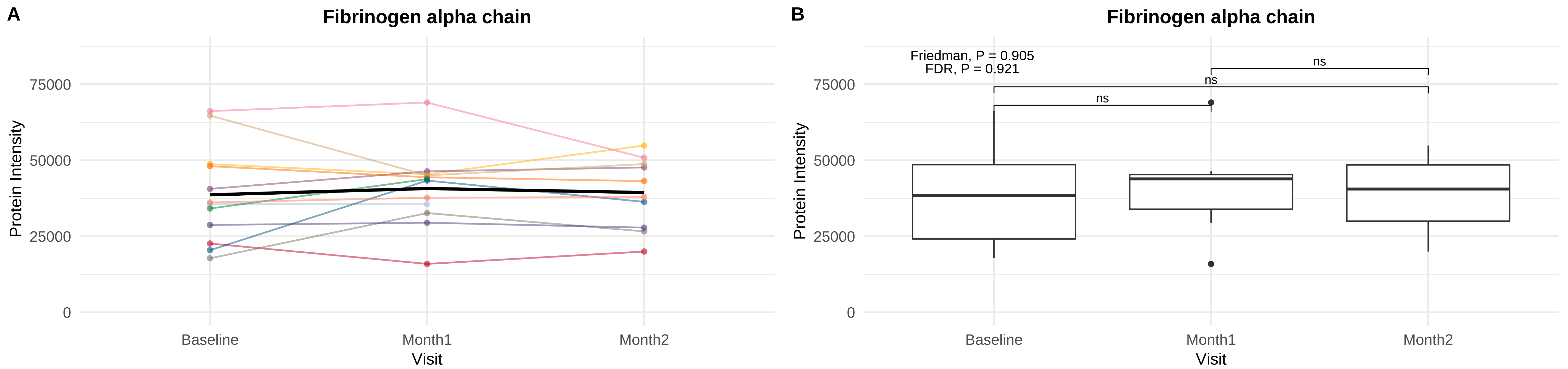

Supplementary Figure S 99

A) Line plot illustrating individual patient trajectories of Fibrinogen alpha chain intensity over time. The bold black line indicates the mean intensity over time. B) Box plots depicting the distribution of Fibrinogen alpha chain intensities at baseline, month 1, and month 2. Only AMD patients with measurements at all visits are included. The median, interquartile range, and outliers are displayed for each time point. Abbreviations: FDR, false discovery rate; ns, non−significant; * p < 0.05; ** p < 0.01; *** p < 0.001.

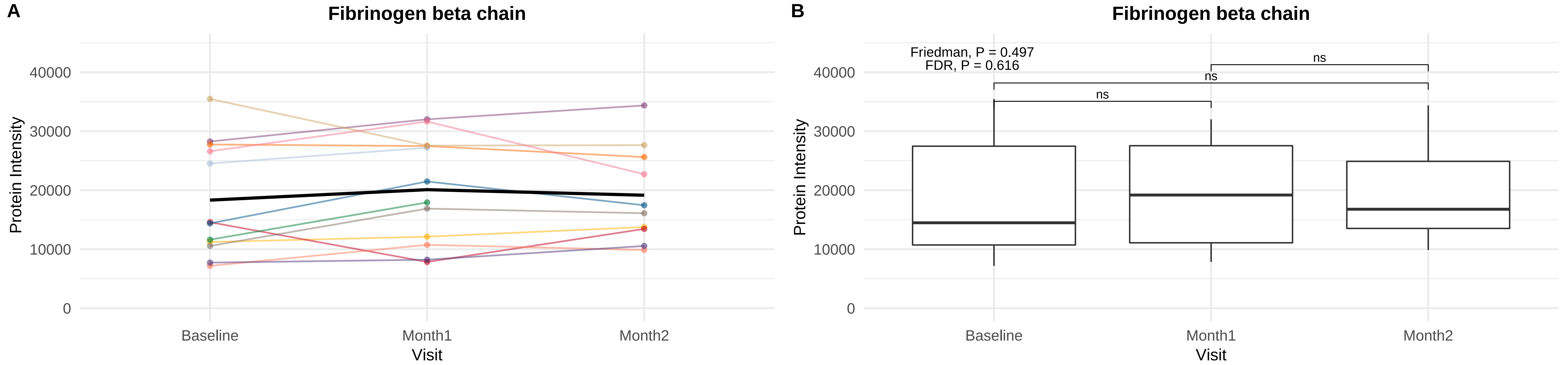

Supplementary Figure S 100

A) Line plot illustrating individual patient trajectories of Fibrinogen beta chain intensity over time. The bold black line indicates the mean intensity over time. B) Box plots depicting the distribution of Fibrinogen beta chain intensities at baseline, month 1, and month 2. Only AMD patients with measurements at all visits are included. The median, interquartile range, and outliers are displayed for each time point. Abbreviations: FDR, false discovery rate; ns, non−significant; * p < 0.05; ** p < 0.01; *** p < 0.001.

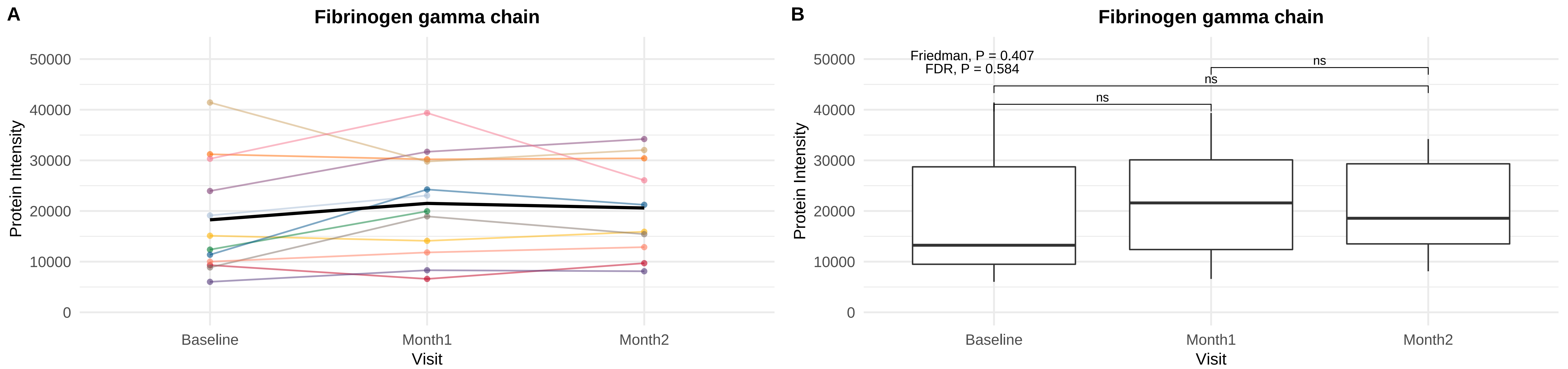

Supplementary Figure S 101

A) Line plot illustrating individual patient trajectories of Fibrinogen gamma chain intensity over time. The bold black line indicates the mean intensity over time. B) Box plots depicting the distribution of Fibrinogen gamma chain intensities at baseline, month 1, and month 2. Only AMD patien[illegible] with measurements at all visits are included. The median, interquartile range, and outliers are displayed for each time point. Abbreviations: FDR, false discovery rate; ns, non−significant; * p < 0.05; ** p < 0.01; *** p < 0.001.

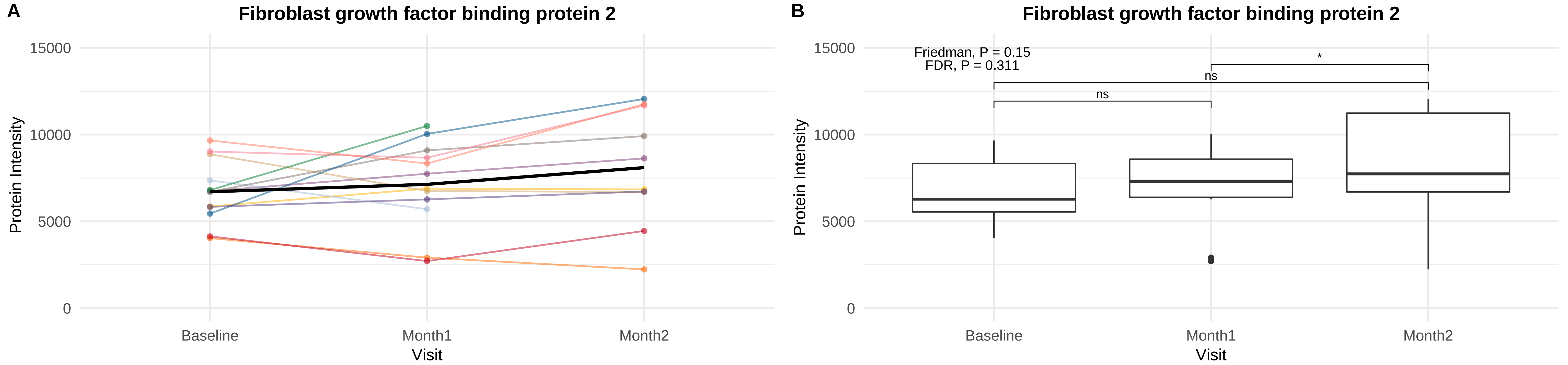

Supplementary Figure S 102

A) Line plot illustrating individual patient trajectories of Fibroblast growth factor binding protein 2 intensity over time. The bold black line indicates the mean intensity over time. B) Box plots depicting the distribution of Fibroblast growth factor binding protein 2 intensities at baseline, month 1, and month 2. Only AMD patients with measurements at all visits are included. The median, interquartile range, and outliers are displayed for each time point. Abbreviations: FDR, false discovery rate; ns, non−significant; * p < 0.05; ** p < 0.01; *** p < 0.001.

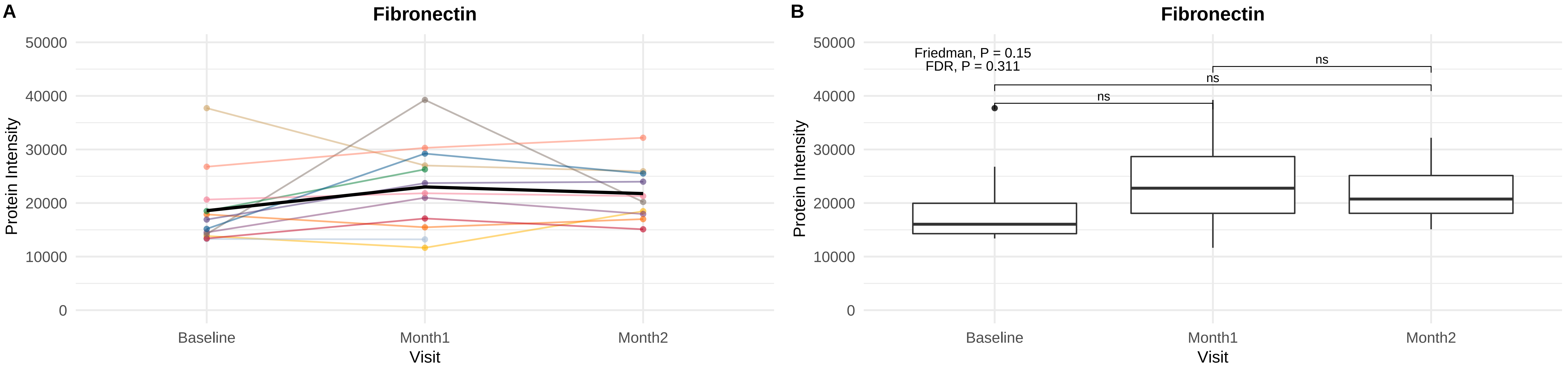

Supplementary Figure S 103

A) Line plot illustrating individual patient trajectories of Fibronectin intensity over time. The bold black line indicates the mean intensity over time. B) Box plots depicting the distribution of Fibronectin intensities at baseline, month 1, and month 2. Only AMD patients with measurements at all visits are included. The median, interquartile range, and outliers are displayed for each time point. Abbreviations: FDR, false discovery rate; ns, non−significant; * p < 0.05; ** p < 0.01; *** p < 0.001.

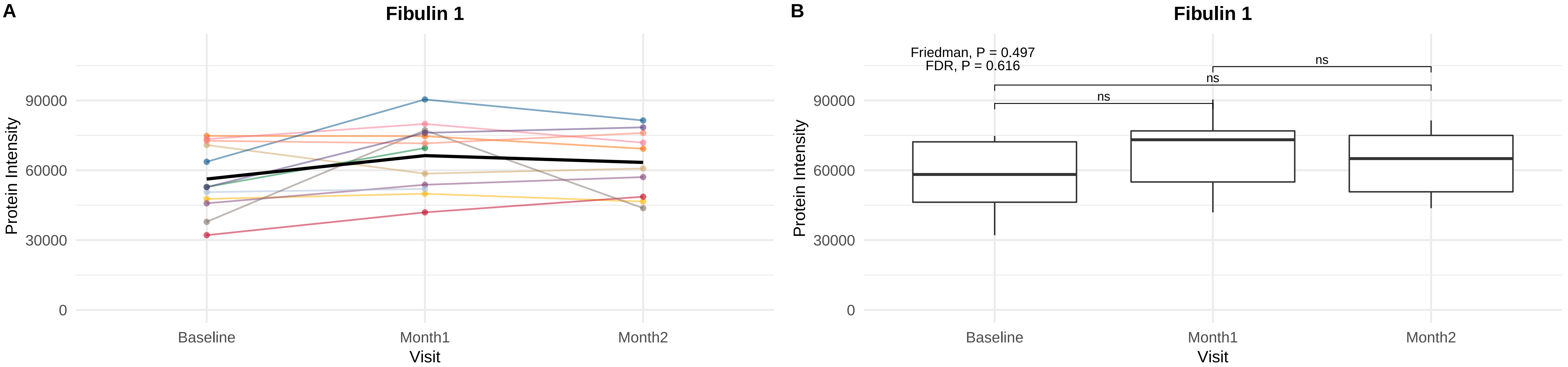

Supplementary Figure S 104

A) Line plot illustrating individual patient trajectories of Fibulin 1 intensity over time. The bold black line indicates the mean intensity over time. B) Box plots depicting the distribution of Fibulin 1 intensities at baseline, month 1, and month 2. Only AMD patients with measurements at all visits are included. The median, interquartile range, and outliers are displayed for each time point. Abbreviations: FDR, false discovery rate; ns, non−significant; * p < 0.05; ** p < 0.01; *** p < 0.001.

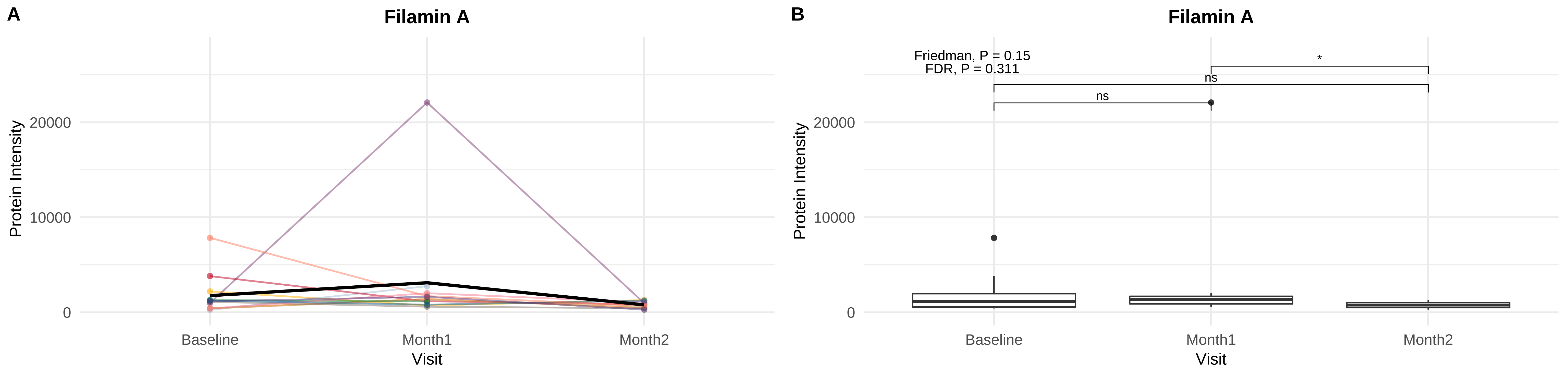

Supplementary Figure S 105

A) Line plot illustrating individual patient trajectories of Filamin A intensity over time. The bold black line indicates the mean intensity over time. B) Box plots depicting the distribution of Filamin A intensities at baseline, month 1, and month 2. Only AMD patients with measurements at all visits are included. The median, interquartile range, and outliers are displayed for each time point. Abbreviations: FDR, false discovery rate; ns, non−significant; * p < 0.05; ** p < 0.01; *** p < 0.001.

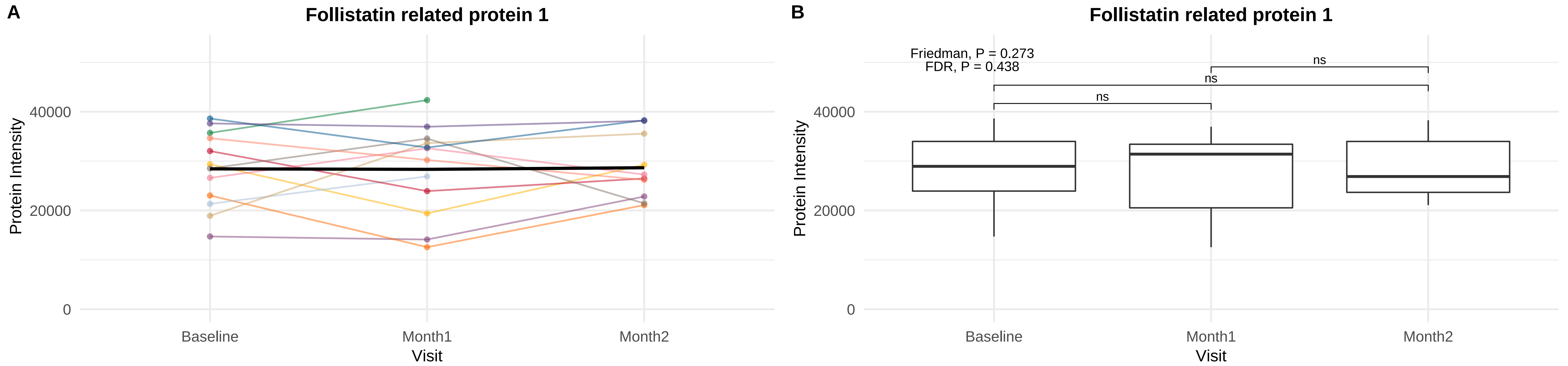

Supplementary Figure S 106

A) Line plot illustrating individual patient trajectories of Follistatin related protein 1 intensity over time. The bold black line indicates the mean intensity over time. B) Box plots depicting the distribution of Follistatin related protein 1 intensities at baseline, month 1, and month 2. Only AMD patients with measurements at all visits are included. The median, interquartile range, and outliers are displayed for each time point. Abbreviations: FDR, false discovery rate; ns, non−significant; * p < 0.05; ** p < 0.01; *** p < 0.001.

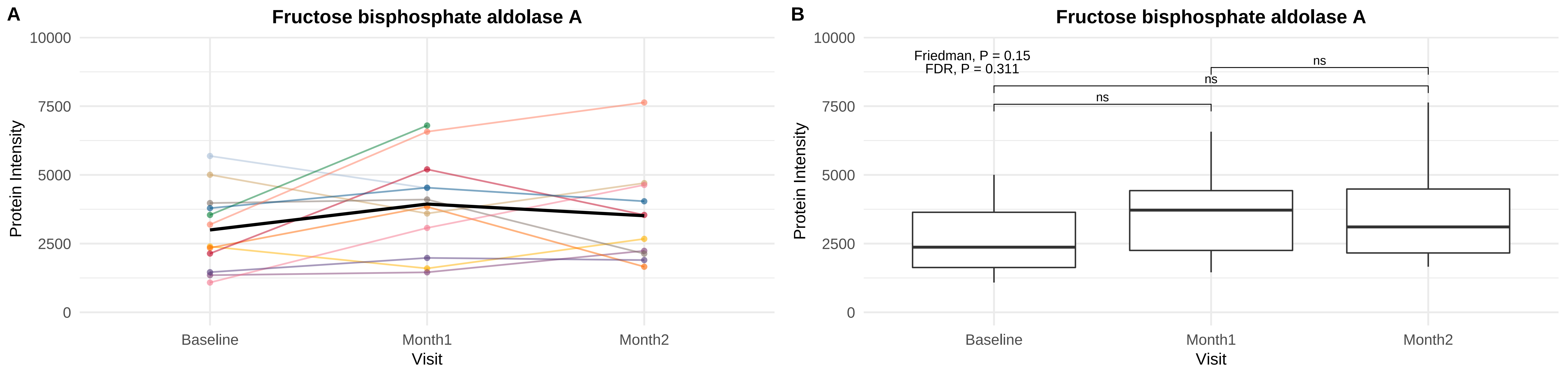

Supplementary Figure S 107

A) Line plot illustrating individual patient trajectories of Fructose bisphosphate aldolase A intensity over time. The bold black line indicates the mean intensity over time. B) Box plots depicting the distribution of Fructose bisphosphate aldolase A intensities at baseline, month 1, and month 2. Only AMD patients with measurements at all visits are included. The median, interquartile range, and outliers are displayed for each time point. Abbreviations: FDR, false discovery rate; ns, non−significant; * p < 0.05; ** p < 0.01; *** p < 0.001.

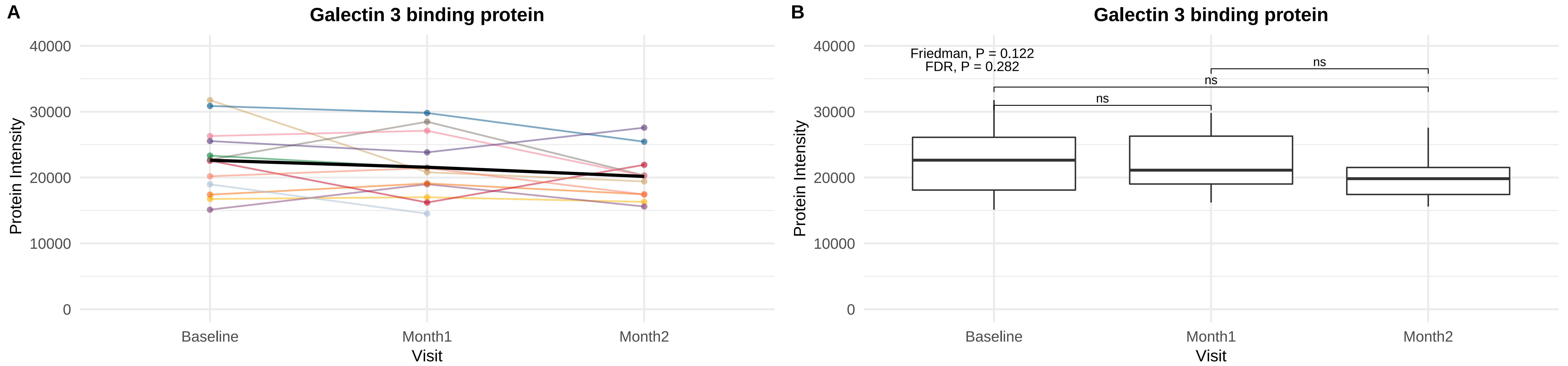

Supplementary Figure S 108

A) Line plot illustrating individual patient trajectories of Galectin 3 binding protein intensity over time. The bold black line indicates the mean intensity over time. B) Box plots depicting the distribution of Galectin 3 binding protein intensities at baseline, month 1, and month 2. Only AMD patients with measurements at all visits are included. The median, interquartile range, and outliers are displayed for each time point. Abbreviations: FDR, false discovery rate; ns, non−significant; * p < 0.05; ** p < 0.01; *** p < 0.001.

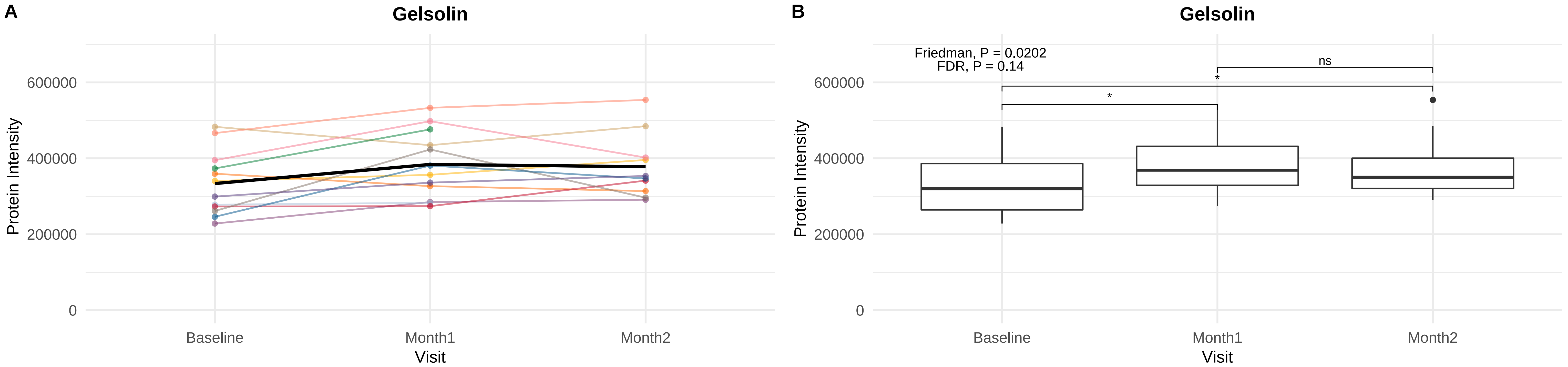

Supplementary Figure S 109

A) Line plot illustrating individual patient trajectories of Gelsolin intensity over time. The bold black line indicates the mean intensity over time. B) Box plots depicting the distribution of Gelsolin intensities at baseline, month 1, and month 2. Only AMD patients with measurements at all visits are included. The median, interquartile range, and outliers are displayed for each time point. Abbreviations: FDR, false discovery rate; ns, non−significant; * p < 0.05; ** p < 0.01; *** p < 0.001.

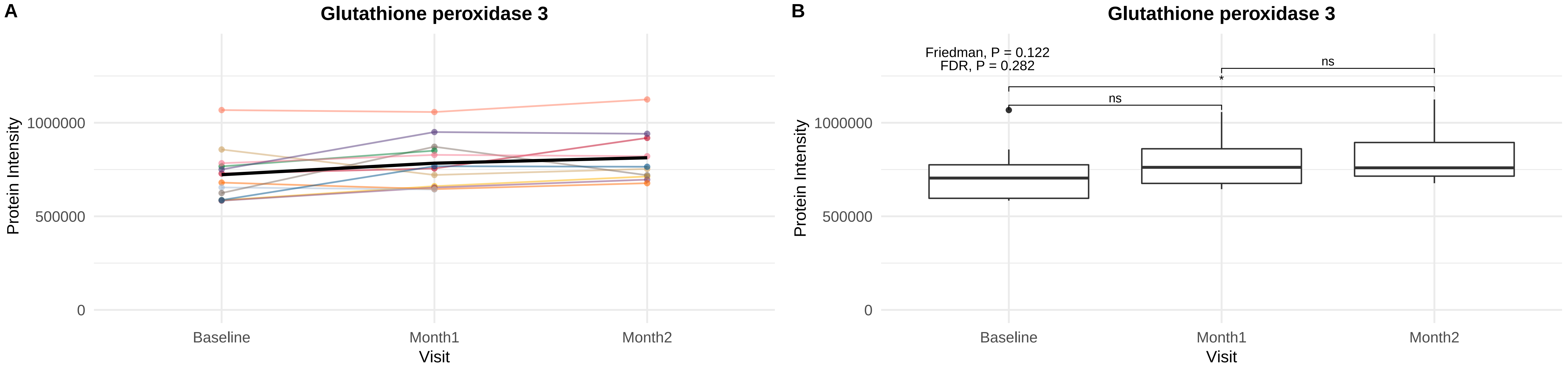

Supplementary Figure S 110

A) Line plot illustrating individual patient trajectories of Glutathione peroxidase 3 intensity over time. The bold black line indicates the mean intensity over time. B) Box plots depicting the distribution of Glutathione peroxidase 3 intensities at baseline, month 1, and month 2. Only AMD patients with measurements at all visits are included. The median, interquartile range, and outliers are displayed for each time point. Abbreviations: FDR, false discovery rate; ns, non−significant; * p < 0.05; ** p < 0.01; *** p < 0.001.

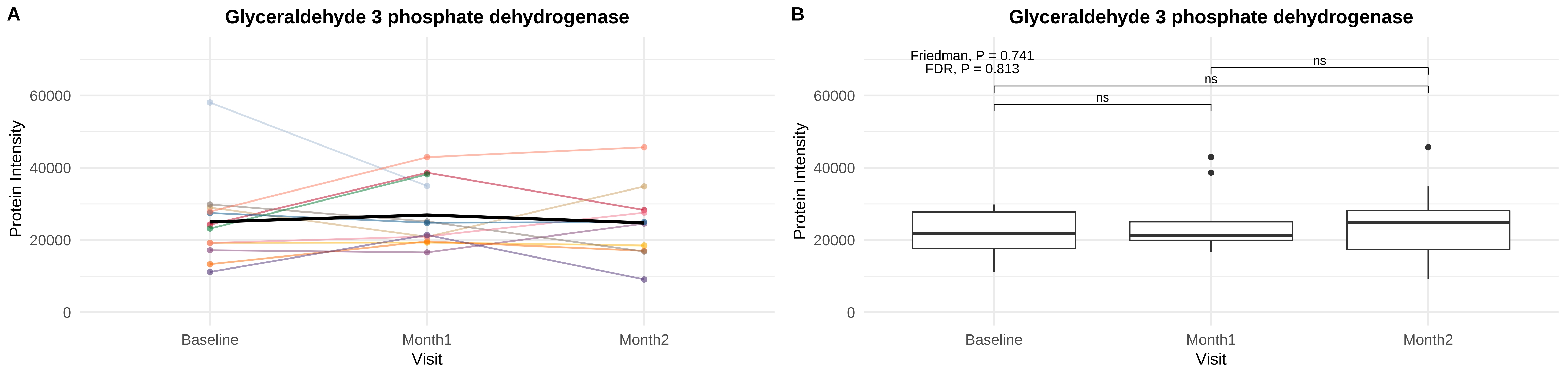

Supplementary Figure S 111

A) Line plot illustrating individual patient trajectories of Glyceraldehyde 3 phosphate dehydrogenase intensity over time. The bold black line indicates the mean intensity over time. B) Box plots depicting the distribution of Glyceraldehyde 3 phosphate dehydrogenase intensities at baseline, month 1, and month 2. Only AMD patients with measurements at all visits are included. The median, interquartile range, and outliers are displayed for each time point. Abbreviations: FDR, false discovery rate; ns, non−significant; * p < 0.05; ** p < 0.01; *** p < 0.001.

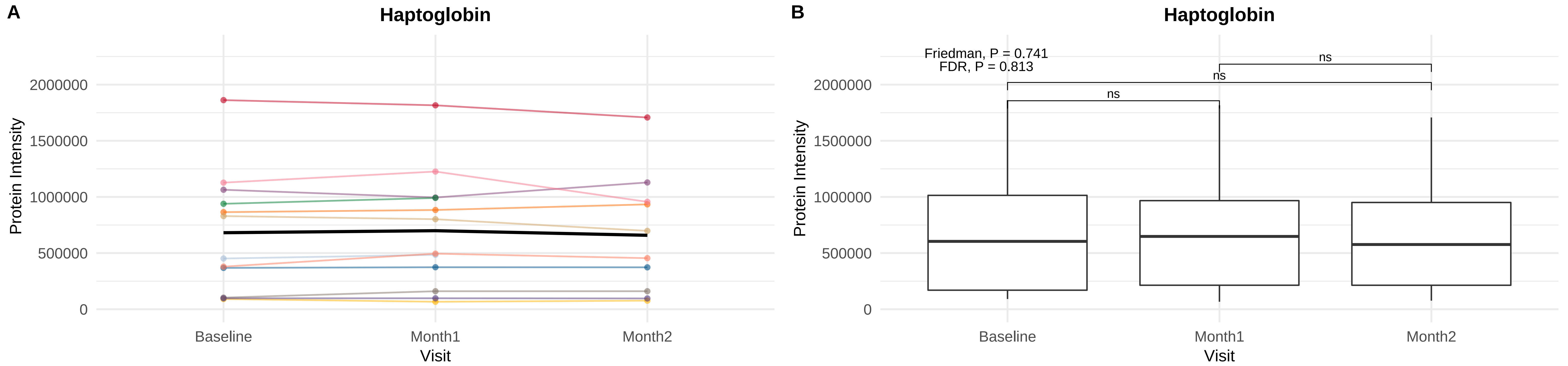

Supplementary Figure S 112

A) Line plot illustrating individual patient trajectories of Haptoglobin intensity over time. The bold black line indicates the mean intensity over time. B) Box plots depicting the distribution of Haptoglobin intensities at baseline, month 1, and month 2. Only AMD patients with measurements at all visits are included. The median, interquartile range, and outliers are displayed for each time point. Abbreviations: FDR, false discovery rate; ns, non−significant; * p < 0.05; ** p < 0.01; *** p < 0.001.

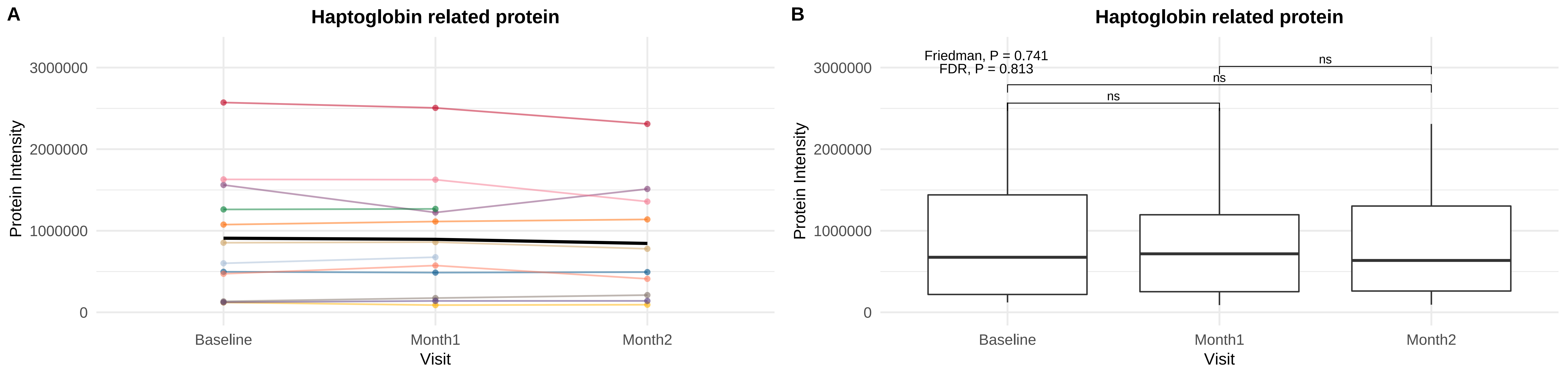

Supplementary Figure S 113

A) Line plot illustrating individual patient trajectories of Haptoglobin related protein intensity over time. The bold black line indicates the mean intensity over time. B) Box plots depicting the distribution of Haptoglobin related protein intensities at baseline, month 1, and month 2. Only AMD patients with measurements at all visits are included. The median, interquartile range, and outliers are displayed for each time point. Abbreviations: FDR, false discovery rate; ns, non−significant; * p < 0.05; ** p < 0.01; *** p < 0.001.

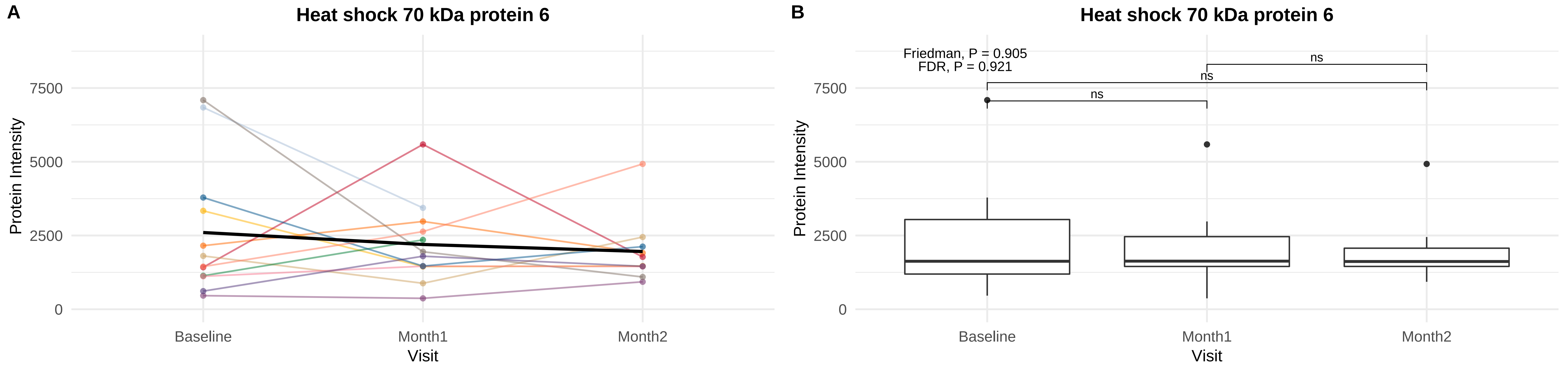

Supplementary Figure S 114

A) Line plot illustrating individual patient trajectories of Heat shock 70 kDa protein 6 intensity over time. The bold black line indicates the mean intensity over time. B) Box plots depicting the distribution of Heat shock 70 kDa protein 6 intensities at baseline, month 1, and month 2. Only AMD patients with measurements at all visits are included. The median, interquartile range, and outliers are displayed for each time point. Abbreviations: FDR, false discovery rate; ns, non−significant; * p < 0.05; ** p < 0.01; *** p < 0.001.

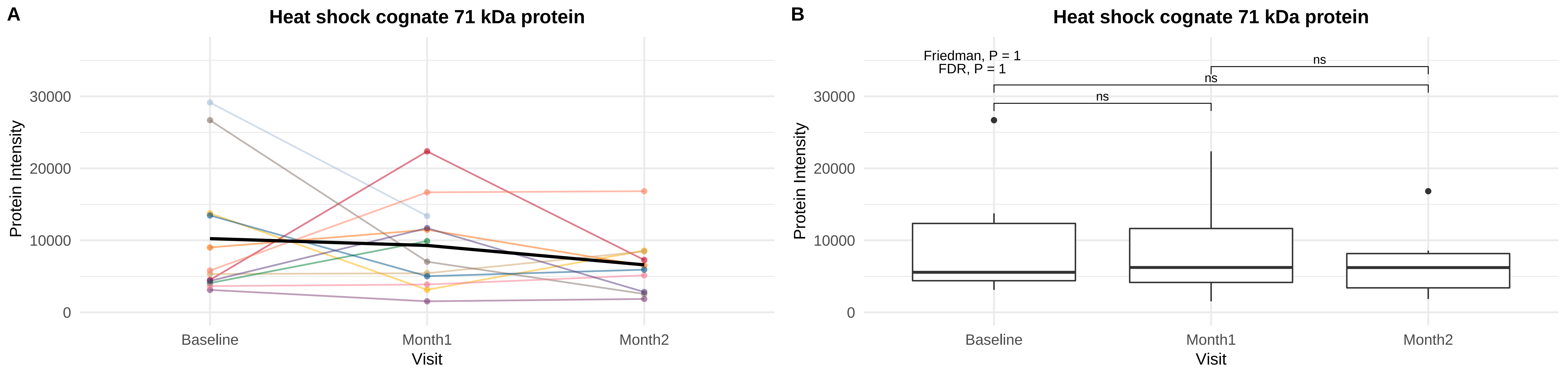

Supplementary Figure S 115

A) Line plot illustrating individual patient trajectories of Heat shock cognate 71 kDa protein intensity over time. The bold black line indicates the mean intensity over time. B) Box plots depicting the distribution of Heat shock cognate 71 kDa protein intensities at baseline, month 1, and month 2. Only AMD patients with measurements at all visits are included. The median, interquartile range, and outliers are displayed for each time point. Abbreviations: FDR, false discovery rate; ns, non−significant; * p < 0.05; ** p < 0.01; *** p < 0.001.

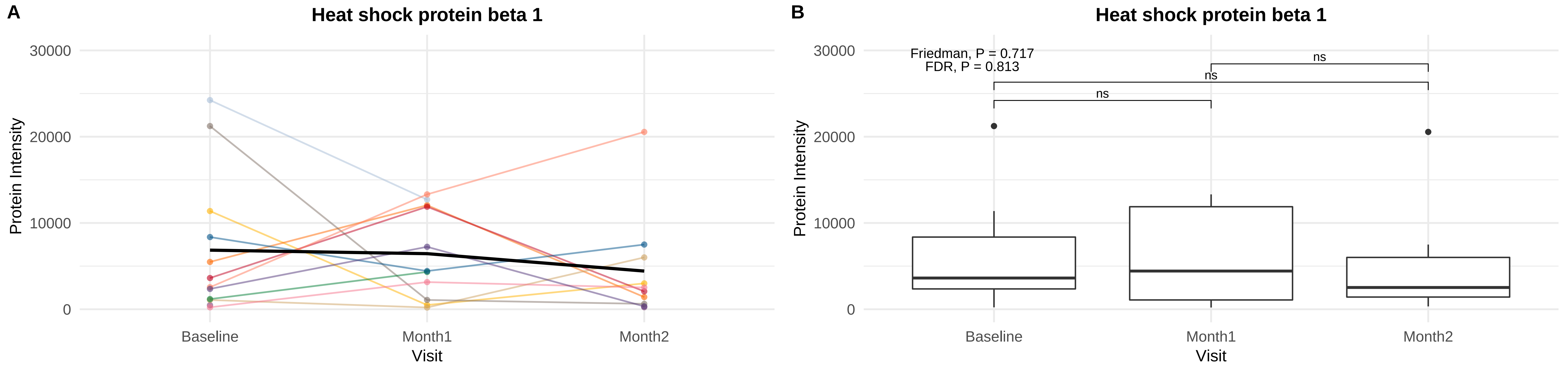

Supplementary Figure S 116

A) Line plot illustrating individual patient trajectories of Heat shock protein beta 1 intensity over time. The bold black line indicates the mean intensity over time. B) Box plots depicting the distribution of Heat shock protein beta 1 intensities at baseline, month 1, and month 2. Only AMD patients with measurements at all visits are included. The median, interquartile range, and outliers are displayed for each time point. Abbreviations: FDR, false discovery rate; ns, non−significant; * p < 0.05; ** p < 0.01; *** p < 0.001.

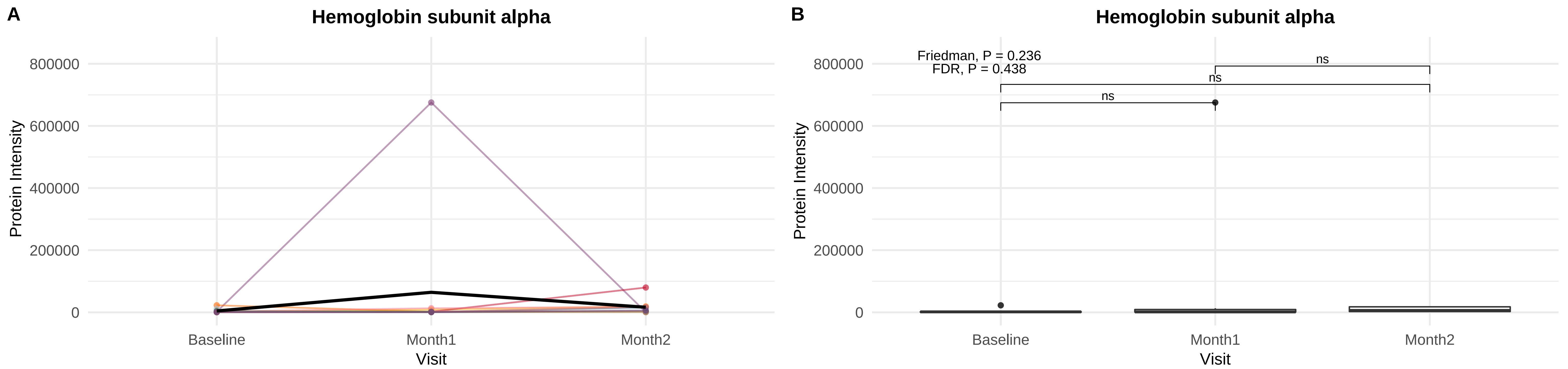

Supplementary Figure S 117

A) Line plot illustrating individual patient trajectories of Hemoglobin subunit alpha intensity over time. The bold black line indicates the mean intensity over time. B) Box plots depicting the distribution of Hemoglobin subunit alpha intensities at baseline, month 1, and month 2. Only AMD patients with measurements at all visits are included. The median, interquartile range, and outliers are displayed for each time point. Abbreviations: FDR, false discovery rate; ns, non−significant; * p < 0.05; ** p < 0.01; *** p < 0.001.

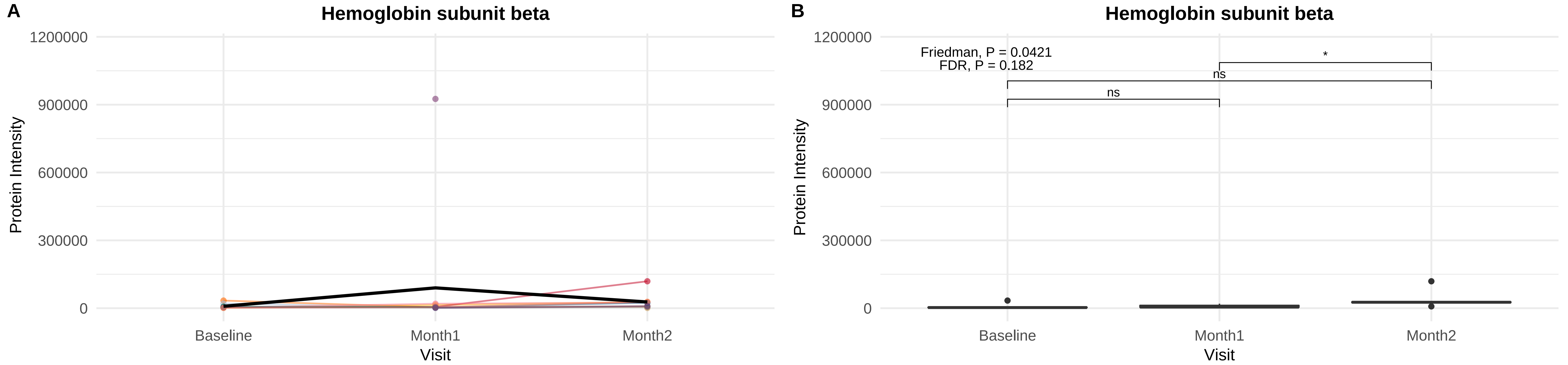

Supplementary Figure S 118

A) Line plot illustrating individual patient trajectories of Hemoglobin subunit beta intensity over time. The bold black line indicates the mean intensity over time. B) Box plots depicting the distribution of Hemoglobin subunit beta intensities at baseline, month 1, and month 2. Only AMD patients with measurements at all visits are included. The median, interquartile range, and outliers are displayed for each time point. Abbreviations: FDR, false discovery rate; ns, non−significant; * p < 0.05; ** p < 0.01; *** p < 0.001.

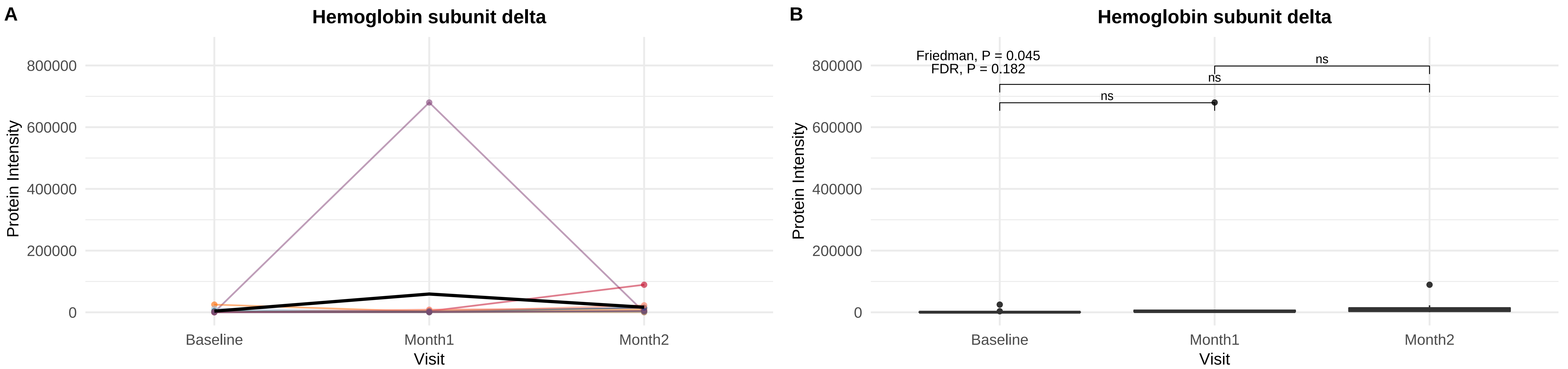

Supplementary Figure S 119

A) Line plot illustrating individual patient trajectories of Hemoglobin subunit delta intensity over time. The bold black line indicates the mean intensity over time. B) Box plots depicting the distribution of Hemoglobin subunit delta intensities at baseline, month 1, and month 2. Only AMD patients with measurements at all visits are included. The median, interquartile range, and outliers are displayed for each time point. Abbreviations: FDR, false discovery rate; ns, non−significant; * p < 0.05; ** p < 0.01; *** p < 0.001.

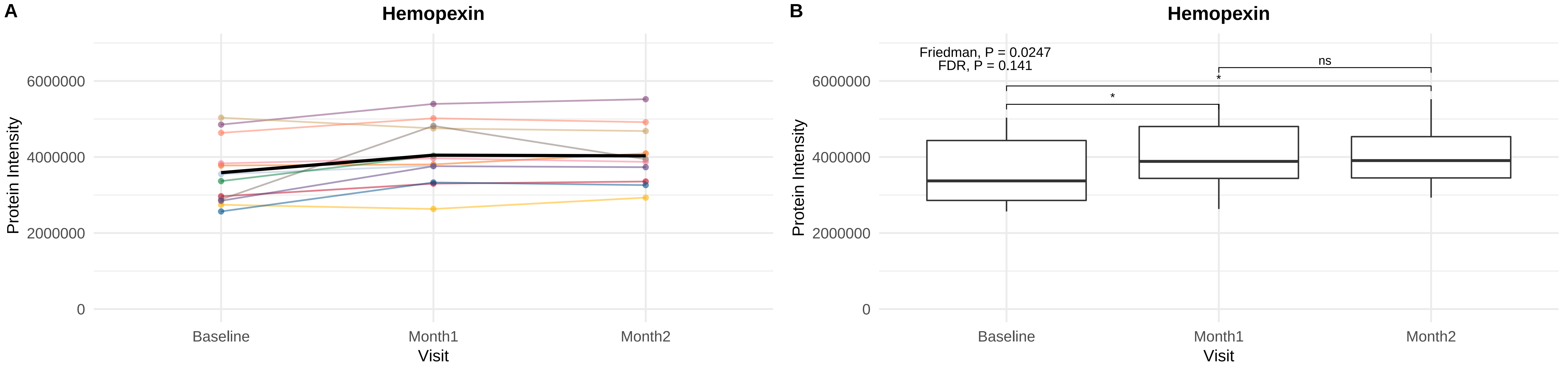

Supplementary Figure S 120

A) Line plot illustrating individual patient trajectories of Hemopexin intensity over time. The bold black line indicates the mean intensity over time. B) Box plots depicting the distribution of Hemopexin intensities at baseline, month 1, and month 2. Only AMD patients with measurements at al visits are included. The median, interquartile range, and outliers are displayed for each time point. Abbreviations: FDR, false discovery rate; ns, non−significant; * p < 0.05; ** p < 0.01; *** p < 0.001.

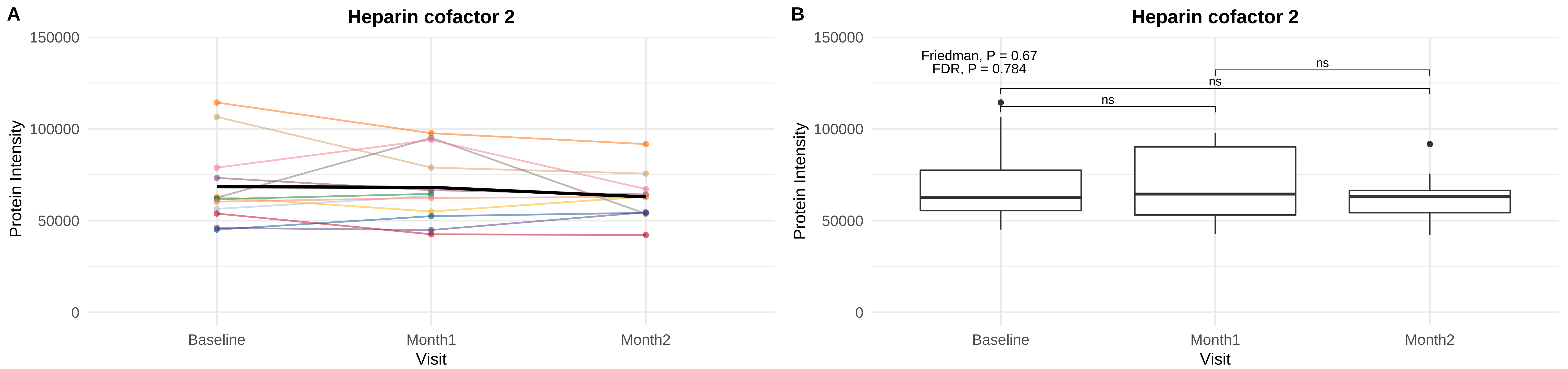

Supplementary Figure S 121

A) Line plot illustrating individual patient trajectories of Heparin cofactor 2 intensity over time. The bold black line indicates the mean intensity over time. B) Box plots depicting the distribution of Heparin cofactor 2 intensities at baseline, month 1, and month 2. Only AMD patients with measurements at all visits are included. The median, interquartile range, and outliers are displayed for each time point. Abbreviations: FDR, false discovery rate; ns, non−significant; * p < 0.05; ** p < 0.01; *** p < 0.001.

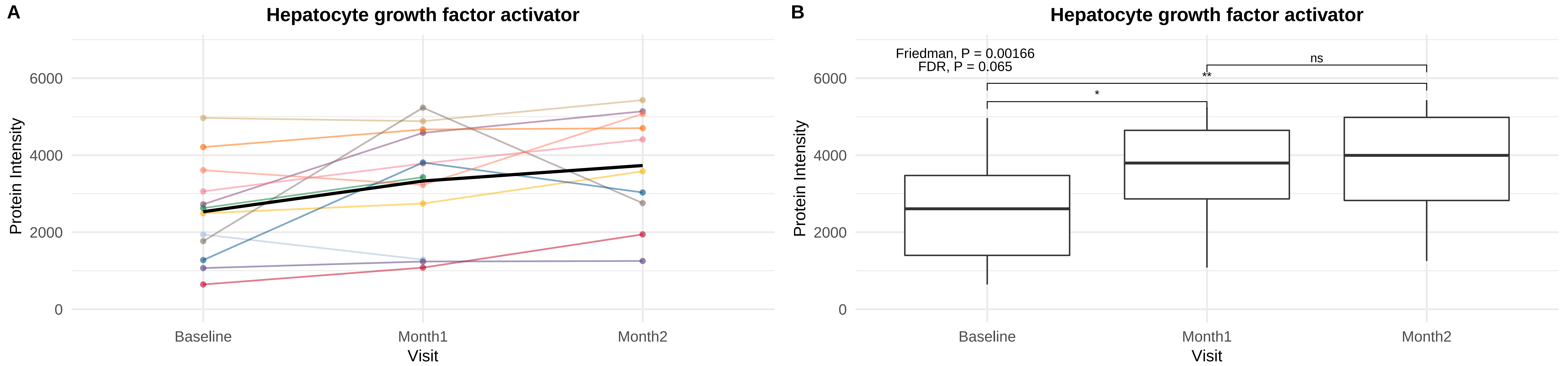

Supplementary Figure S 122

A) Line plot illustrating individual patient trajectories of Hepatocyte growth factor activator intensity over time. The bold black line indicates the mean intensity over time. B) Box plots depicting the distribution of Hepatocyte growth factor activator intensities at baseline, month 1, and month 2. Only AMD patients with measurements at all visits are included. The median, interquartile range, and outliers are displayed for each time point. Abbreviations: FDR, false discovery rate; ns, non−significant; * p < 0.05; ** p < 0.01; *** p < 0.001.

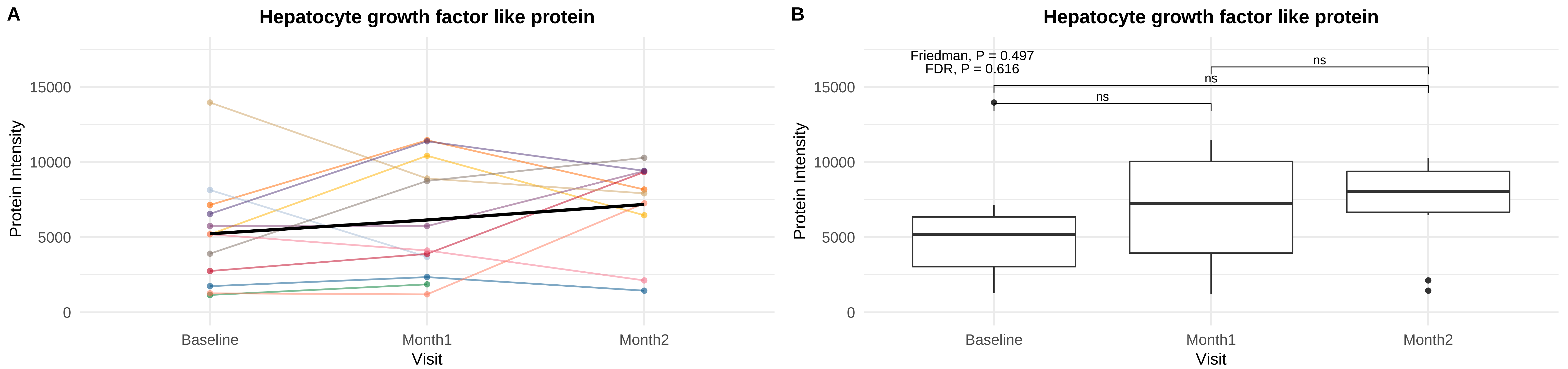

Supplementary Figure S 123

A) Line plot illustrating individual patient trajectories of Hepatocyte growth factor like protein intensity over time. The bold black line indicates the mean intensity over time. B) Box plots depicting the distribution of Hepatocyte growth factor like protein intensities at baseline, month 1, and month 2. Only AMD patients with measurements at all visits are included. The median, interquartile range, and outliers are displayed for each time point. Abbreviations: FDR, false discovery rate; ns, non−significant; * p < 0.05; ** p < 0.01; *** p < 0.001.

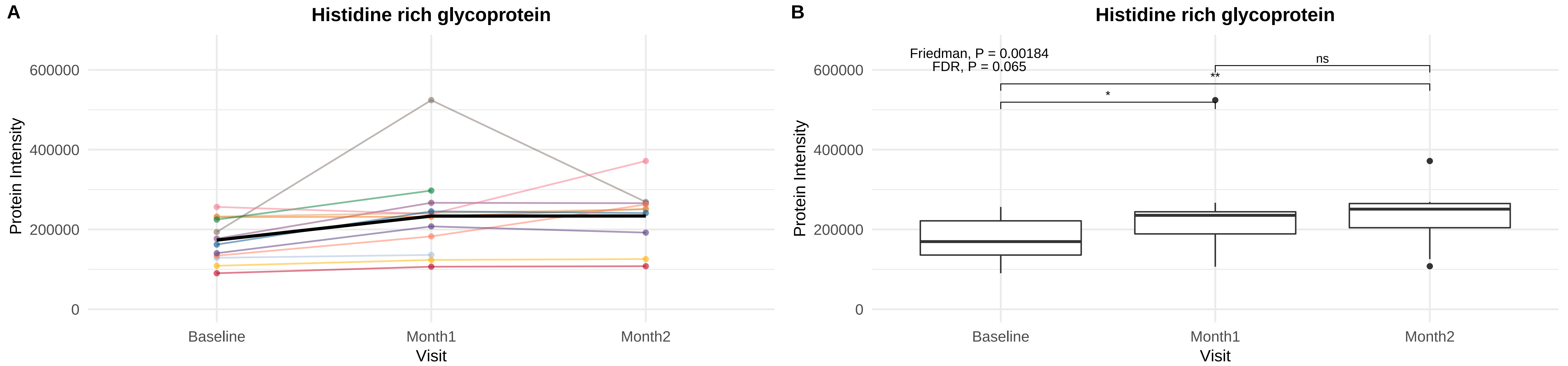

Supplementary Figure S 124

A) Line plot illustrating individual patient trajectories of Histidine rich glycoprotein intensity over time. The bold black line indicates the mean intensity over time. B) Box plots depicting the distribution of Histidine rich glycoprotein intensities at baseline, month 1, and month 2. Only AMD patients with measurements at all visits are included. The median, interquartile range, and outliers are displayed for each time point. Abbreviations: FDR, false discovery rate; ns, non−significant; * p < 0.05; ** p < 0.01; *** p < 0.001.

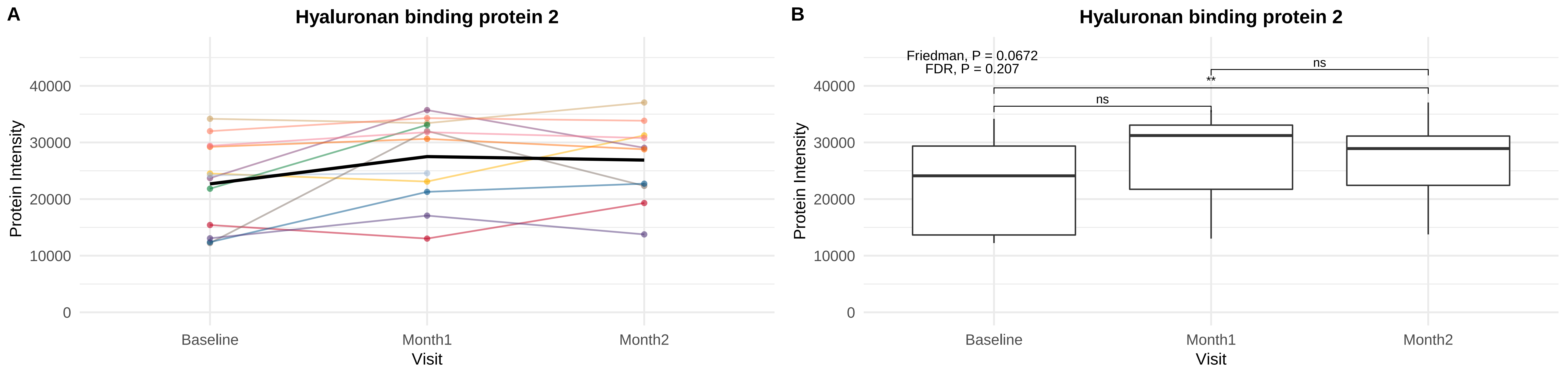

Supplementary Figure S 125

A) Line plot illustrating individual patient trajectories of Hyaluronan binding protein 2 intensity over time. The bold black line indicates the mean intensity over time. B) Box plots depicting the distribution of Hyaluronan binding protein 2 intensities at baseline, month 1, and month 2. Only AMD patients with measurements at all visits are included. The median, interquartile range, and outliers are displayed for each time point. Abbreviations: FDR, false discovery rate; ns, non−significant; * p < 0.05; ** p < 0.01; *** p < 0.001.

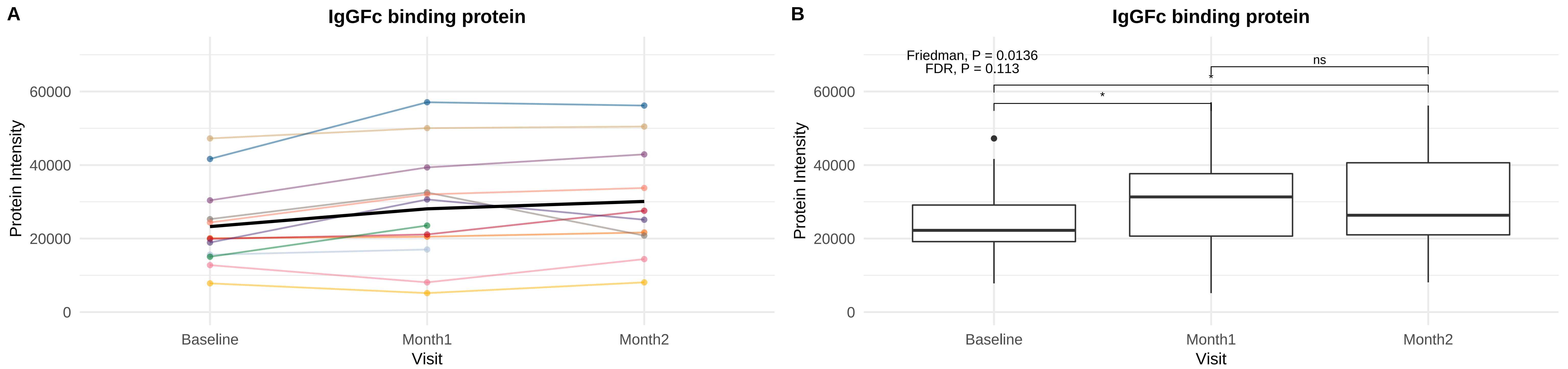

Supplementary Figure S 126

A) Line plot illustrating individual patient trajectories of IgGFc binding protein intensity over time. The bold black line indicates the mean intensity over time. B) Box plots depicting the distribution of IgGFc binding protein intensities at baseline, month 1, and month 2. Only AMD patients with measurements at all visits are included. The median, interquartile range, and outliers are displayed for each time point. Abbreviations: FDR, false discovery rate; ns, non−significant; * p < 0.05; ** p < 0.01; *** p < 0.001.

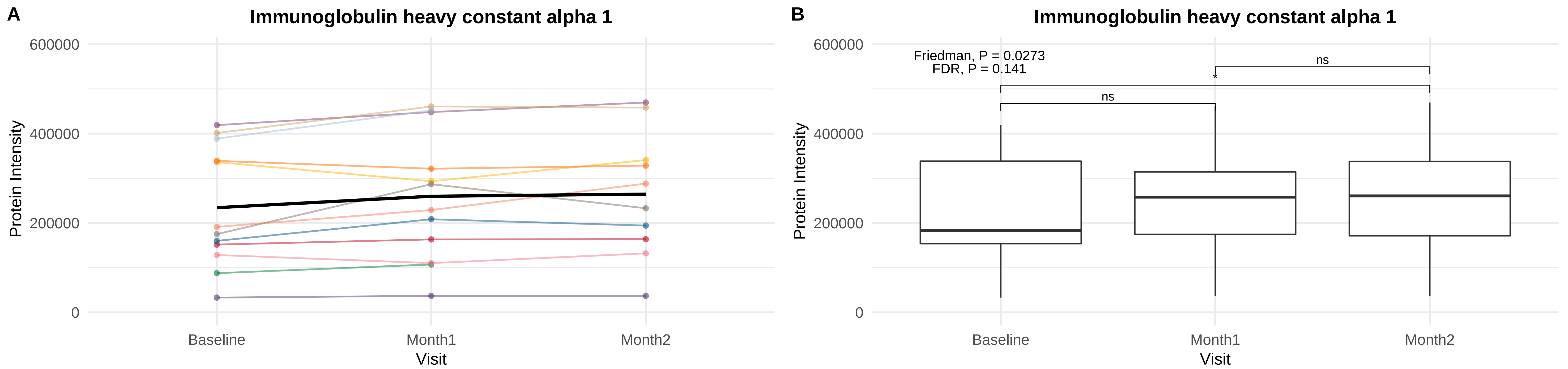

Supplementary Figure S 127

A) Line plot illustrating individual patient trajectories of Immunoglobulin heavy constant alpha 1 intensity over time. The bold black line indicates the mean intensity over time. B) Box plots depicting the distribution of Immunoglobulin heavy constant alpha 1 intensities at baseline, month 1, and month 2. Only AMD patients with measurements at all visits are included. The median, interquartile range, and outliers are displayed for each time point. Abbreviations: FDR, false discovery rate; ns, non−significant; * p < 0.05; ** p < 0.01; *** p < 0.001.

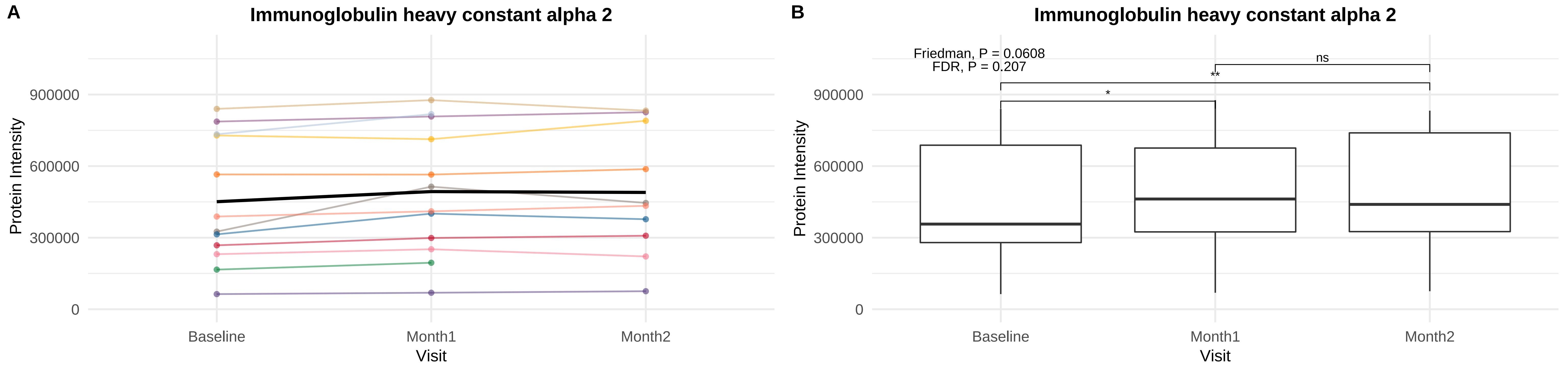

Supplementary Figure S 128

A) Line plot illustrating individual patient trajectories of Immunoglobulin heavy constant alpha 2 intensity over time. The bold black line indicates the mean intensity over time. B) Box plots depicting the distribution of Immunoglobulin heavy constant alpha 2 intensities at baseline, month 1, and month 2. Only AMD patients with measurements at all visits are included. The median, interquartile range, and outliers are displayed for each time point. Abbreviations: FDR, false discovery rate; ns, non−significant; * p < 0.05; ** p < 0.01; *** p < 0.001.

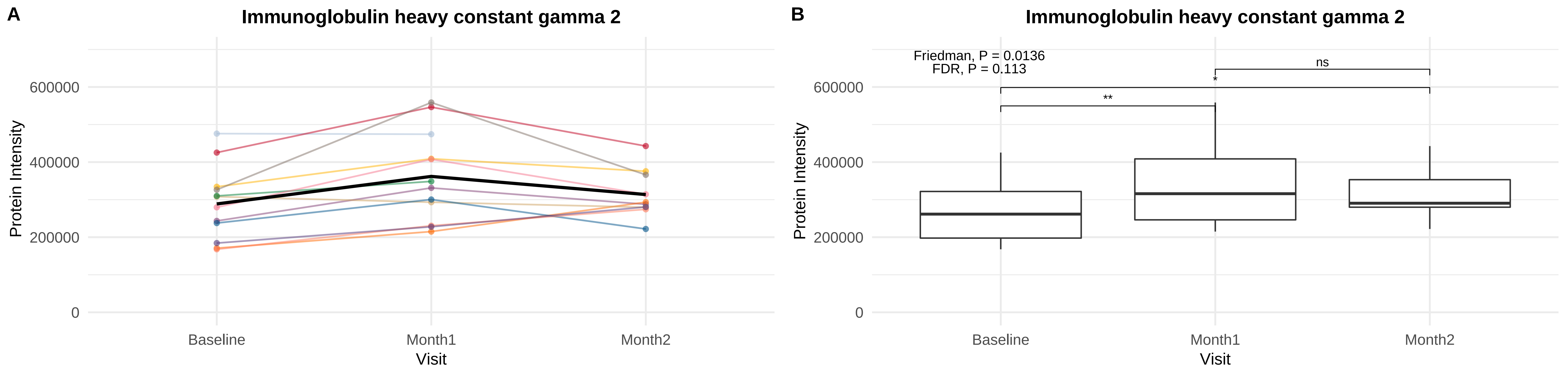

Supplementary Figure S 129

A) Line plot illustrating individual patient trajectories of Immunoglobulin heavy constant gamma 2 intensity over time. The bold black line indicates the mean intensity over time. B) Box plots depicting the distribution of Immunoglobulin heavy constant gamma 2 intensities at baseline, month and month 2. Only AMD patients with measurements at all visits are included. The median, interquartile range, and outliers are displayed for each time point. Abbreviations: FDR, false discovery rate; ns, non−significant; * p < 0.05; ** p < 0.01; *** p < 0.001.

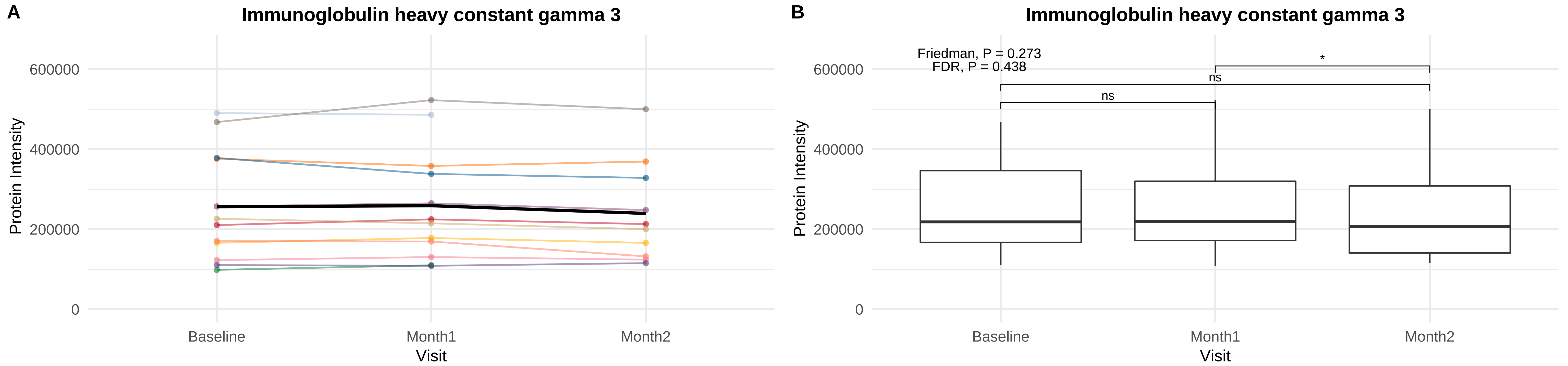

Supplementary Figure S 130

A) Line plot illustrating individual patient trajectories of Immunoglobulin heavy constant gamma 3 intensity over time. The bold black line indicates the mean intensity over time. B) Box plots depicting the distribution of Immunoglobulin heavy constant gamma 3 intensities at baseline, month and month 2. Only AMD patients with measurements at all visits are included. The median, interquartile range, and outliers are displayed for each time point. Abbreviations: FDR, false discovery rate; ns, non−significant; * p < 0.05; ** p < 0.01; *** p < 0.001.

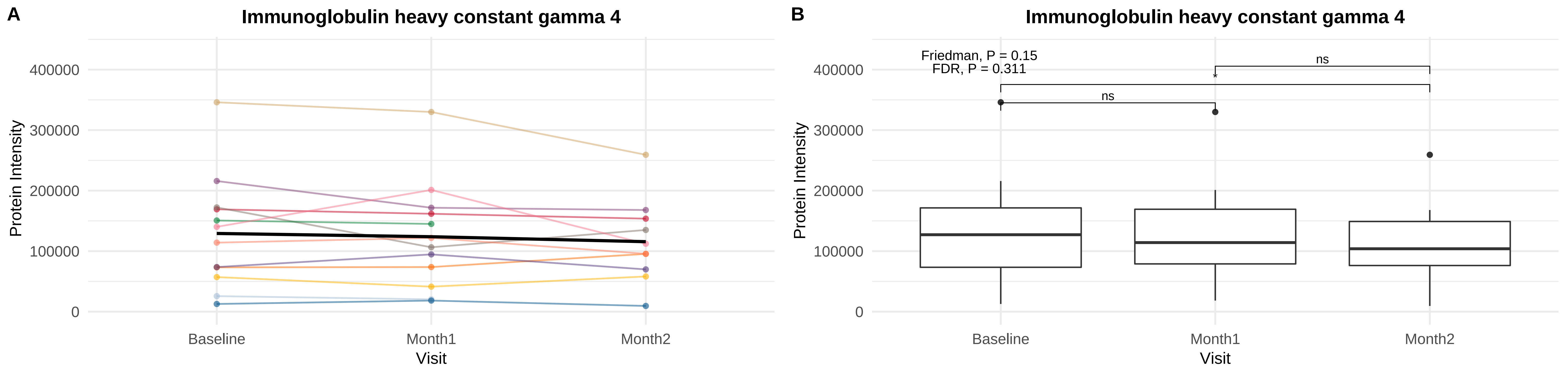

Supplementary Figure S 131

A) Line plot illustrating individual patient trajectories of Immunoglobulin heavy constant gamma 4 intensity over time. The bold black line indicates the mean intensity over time. B) Box plots depicting the distribution of Immunoglobulin heavy constant gamma 4 intensities at baseline, month and month 2. Only AMD patients with measurements at all visits are included. The median, interquartile range, and outliers are displayed for each time point. Abbreviations: FDR, false discovery rate; ns, non−significant; * p < 0.05; ** p < 0.01; *** p < 0.001.

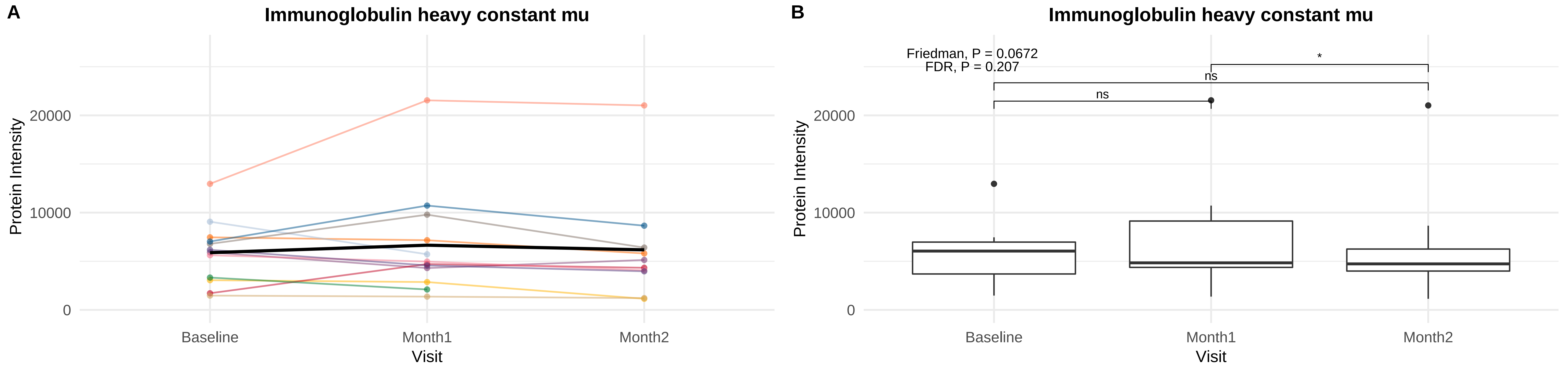

Supplementary Figure S 132

A) Line plot illustrating individual patient trajectories of Immunoglobulin heavy constant mu intensity over time. The bold black line indicates the mean intensity over time. B) Box plots depicting the distribution of Immunoglobulin heavy constant mu intensities at baseline, month 1, and mont 2. Only AMD patients with measurements at all visits are included. The median, interquartile range, and outliers are displayed for each time point. Abbreviations: FDR, false discovery rate; ns, non−significant; * p < 0.05; ** p < 0.01; *** p < 0.001.

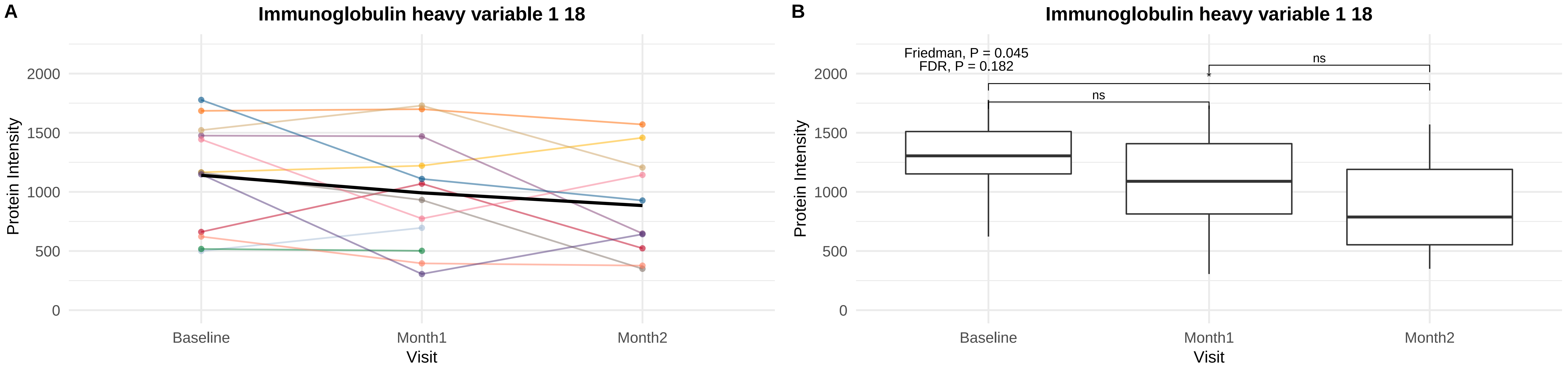

Supplementary Figure S 133

A) Line plot illustrating individual patient trajectories of Immunoglobulin heavy variable 1 18 intensity over time. The bold black line indicates the mean intensity over time. B) Box plots depicting the distribution of Immunoglobulin heavy variable 1 18 intensities at baseline, month 1, and month 2. Only AMD patients with measurements at all visits are included. The median, interquartile range, and outliers are displayed for each time point. Abbreviations: FDR, false discovery rate; ns, non−significant; * p < 0.05; ** p < 0.01; *** p < 0.001.

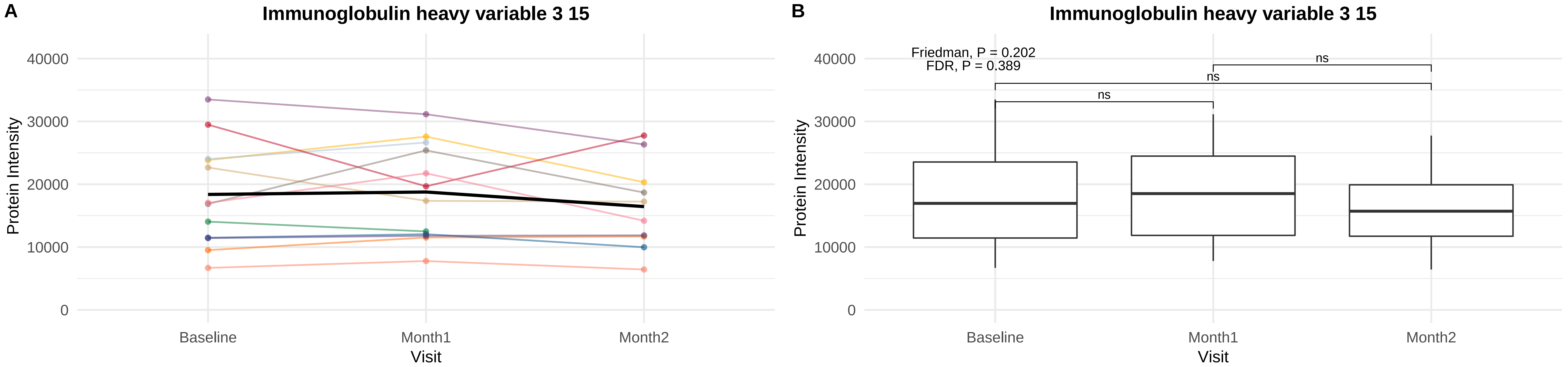

Supplementary Figure S 134

A) Line plot illustrating individual patient trajectories of Immunoglobulin heavy variable 3 15 intensity over time. The bold black line indicates the mean intensity over time. B) Box plots depicting the distribution of Immunoglobulin heavy variable 3 15 intensities at baseline, month 1, and month 2. Only AMD patients with measurements at all visits are included. The median, interquartile range, and outliers are displayed for each time point. Abbreviations: FDR, false discovery rate; ns, non−significant; * p < 0.05; ** p < 0.01; *** p < 0.001.

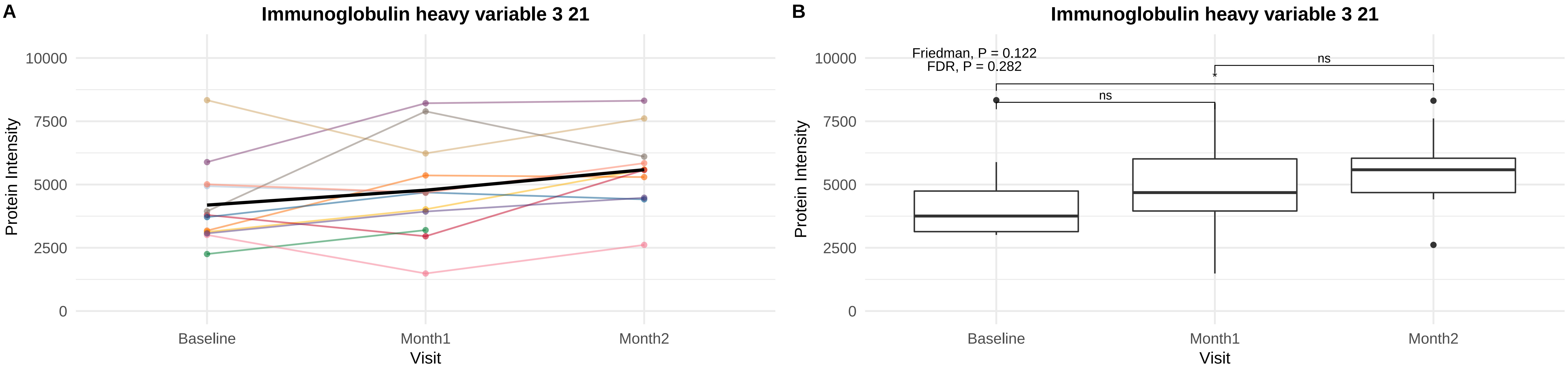

Supplementary Figure S 135

A) Line plot illustrating individual patient trajectories of Immunoglobulin heavy variable 3 21 intensity over time. The bold black line indicates the mean intensity over time. B) Box plots depicting the distribution of Immunoglobulin heavy variable 3 21 intensities at baseline, month 1, and month 2. Only AMD patients with measurements at all visits are included. The median, interquartile range, and outliers are displayed for each time point. Abbreviations: FDR, false discovery rate; ns, non−significant; * p < 0.05; ** p < 0.01; *** p < 0.001.

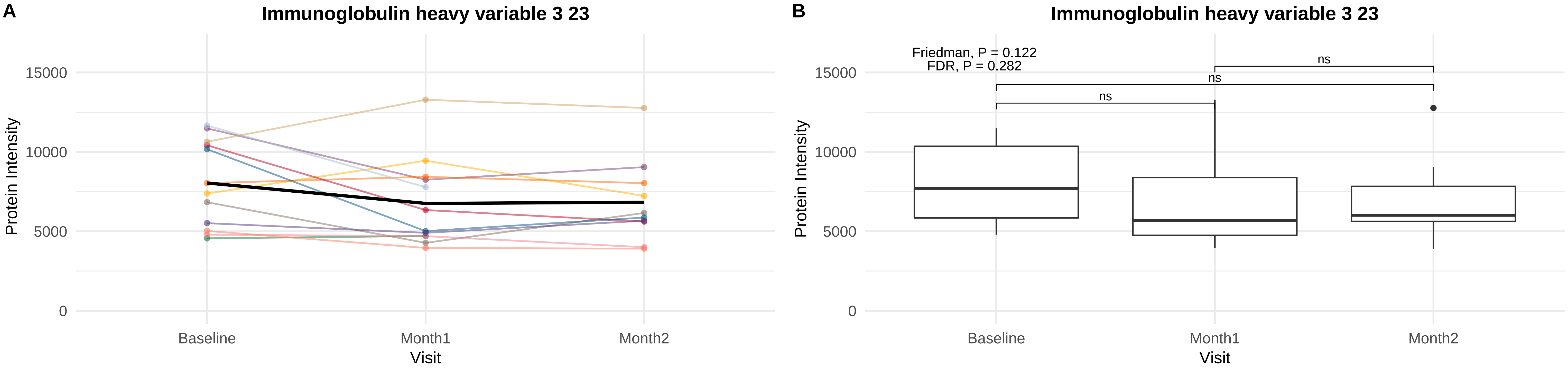

Supplementary Figure S 136

A) Line plot illustrating individual patient trajectories of Immunoglobulin heavy variable 3 23 intensity over time. The bold black line indicates the mean intensity over time. B) Box plots depicting the distribution of Immunoglobulin heavy variable 3 23 intensities at baseline, month 1, and month 2. Only AMD patients with measurements at all visits are included. The median, interquartile range, and outliers are displayed for each time point. Abbreviations: FDR, false discovery rate; ns, non−significant; * p < 0.05; ** p < 0.01; *** p < 0.001.

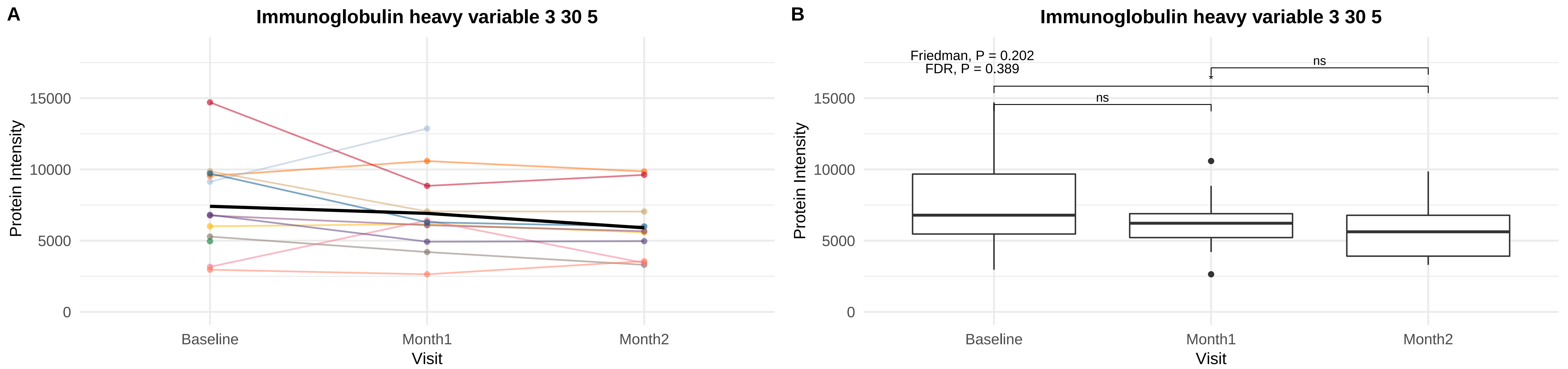

Supplementary Figure S 137

A) Line plot illustrating individual patient trajectories of Immunoglobulin heavy variable 3 30 5 intensity over time. The bold black line indicates the mean intensity over time. B) Box plots depicting the distribution of Immunoglobulin heavy variable 3 30 5 intensities at baseline, month 1, and month 2. Only AMD patients with measurements at all visits are included. The median, interquartile range, and outliers are displayed for each time point. Abbreviations: FDR, false discovery rate; ns, non−significant; * p < 0.05; ** p < 0.01; *** p < 0.001.

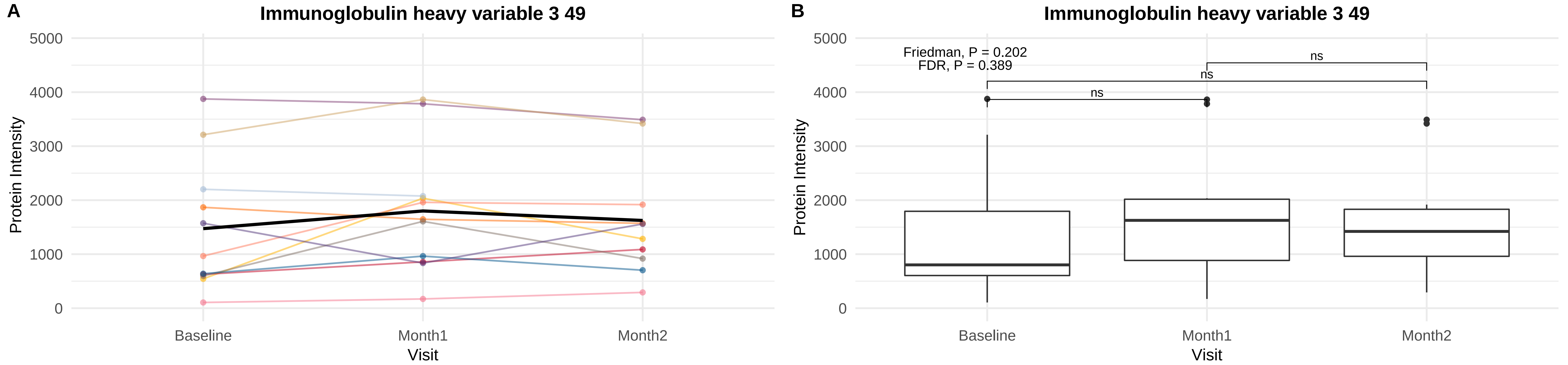

Supplementary Figure S 138

A) Line plot illustrating individual patient trajectories of Immunoglobulin heavy variable 3 49 intensity over time. The bold black line indicates the mean intensity over time. B) Box plots depicting the distribution of Immunoglobulin heavy variable 3 49 intensities at baseline, month 1, and month 2. Only AMD patients with measurements at all visits are included. The median, interquartile range, and outliers are displayed for each time point. Abbreviations: FDR, false discovery rate; ns, non−significant; * p < 0.05; ** p < 0.01; *** p < 0.001.

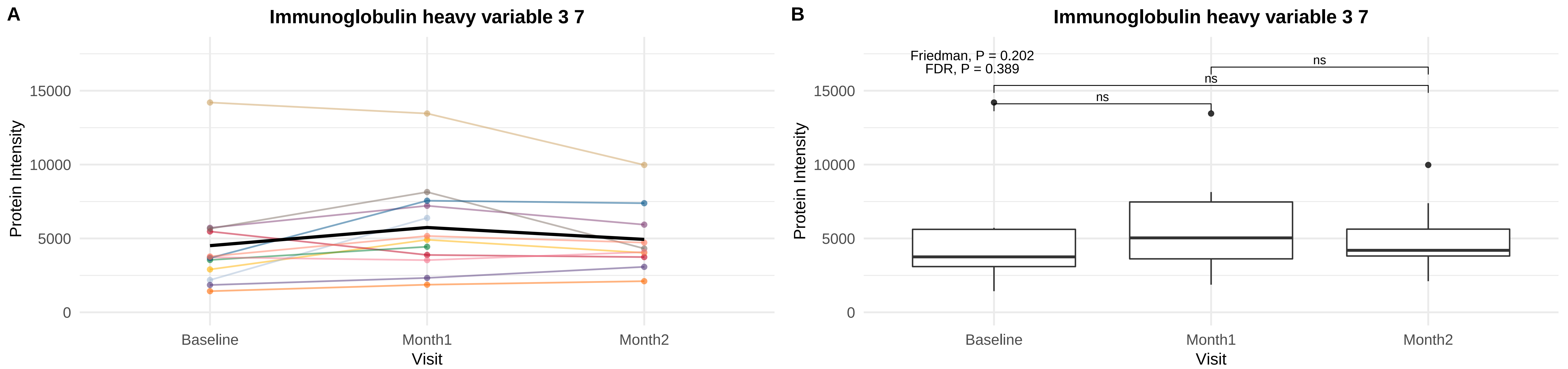

Supplementary Figure S 139

A) Line plot illustrating individual patient trajectories of Immunoglobulin heavy variable 3 7 intensity over time. The bold black line indicates the mean intensity over time. B) Box plots depicting the distribution of Immunoglobulin heavy variable 3 7 intensities at baseline, month 1, and month 2. Only AMD patients with measurements at all visits are included. The median, interquartile range, and outliers are displayed for each time point. Abbreviations: FDR, false discovery rate; ns, non−significant; * p < 0.05; ** p < 0.01; *** p < 0.001.

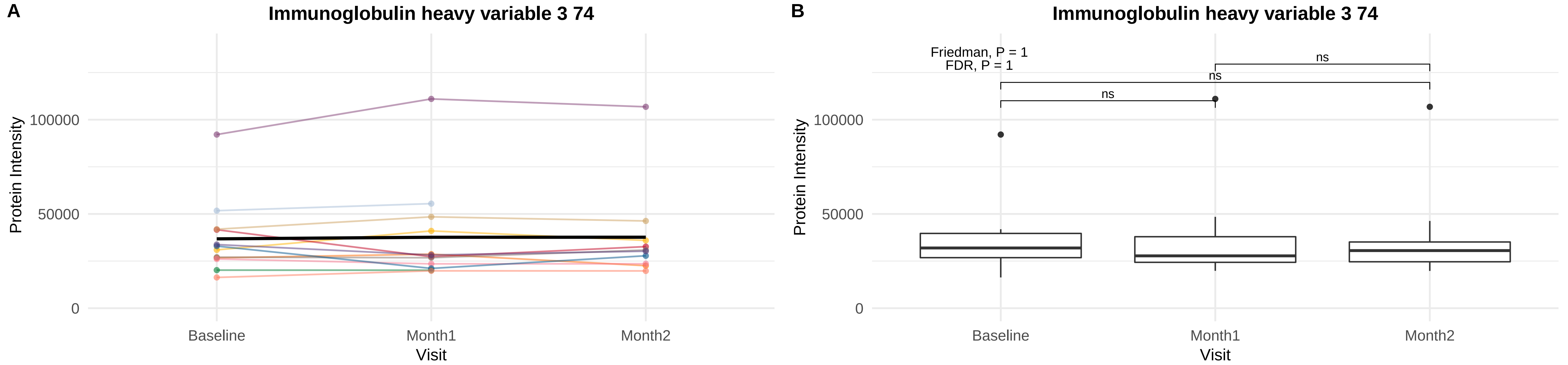

Supplementary Figure S 140

A) Line plot illustrating individual patient trajectories of Immunoglobulin heavy variable 3 74 intensity over time. The bold black line indicates the mean intensity over time. B) Box plots depicting the distribution of Immunoglobulin heavy variable 3 74 intensities at baseline, month 1, and month 2. Only AMD patients with measurements at all visits are included. The median, interquartile range, and outliers are displayed for each time point. Abbreviations: FDR, false discovery rate; ns, non−significant; * p < 0.05; ** p < 0.01; *** p < 0.001.

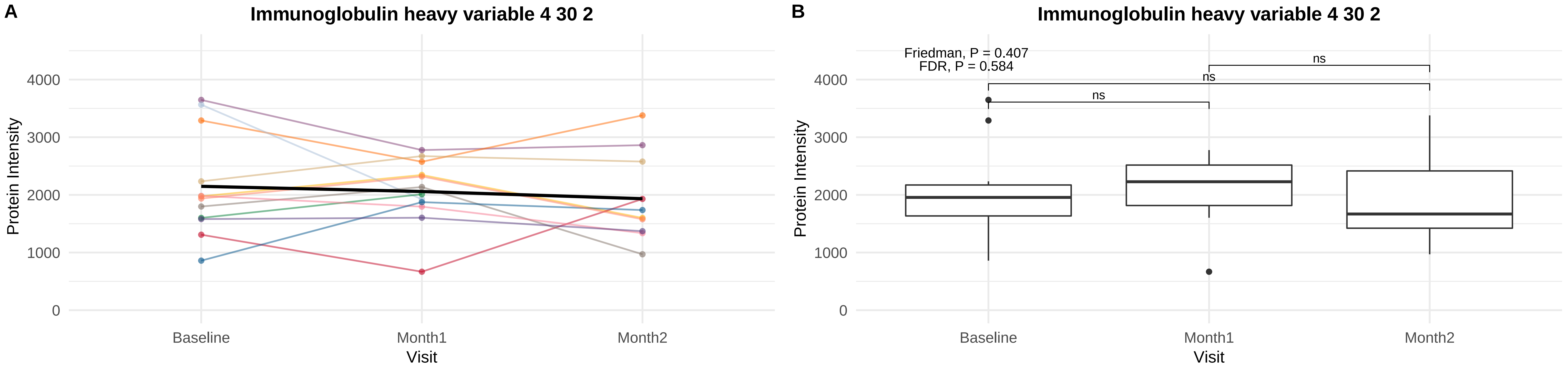

Supplementary Figure S 141

A) Line plot illustrating individual patient trajectories of Immunoglobulin heavy variable 4 30 2 intensity over time. The bold black line indicates the mean intensity over time. B) Box plots depicting the distribution of Immunoglobulin heavy variable 4 30 2 intensities at baseline, month 1, and month 2. Only AMD patients with measurements at all visits are included. The median, interquartile range, and outliers are displayed for each time point. Abbreviations: FDR, false discovery rate; ns, non−significant; * p < 0.05; ** p < 0.01; *** p < 0.001.

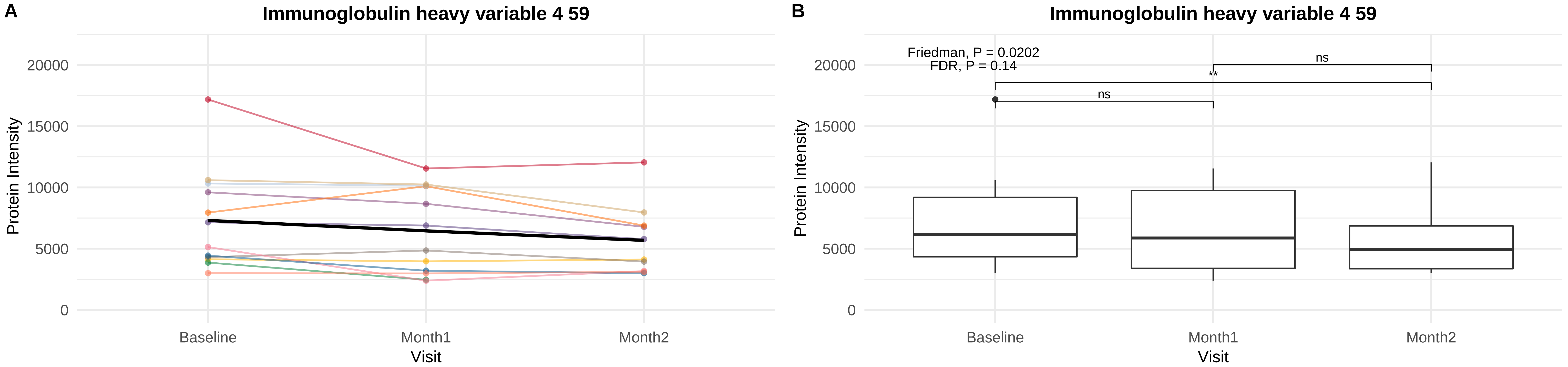

Supplementary Figure S 142

A) Line plot illustrating individual patient trajectories of Immunoglobulin heavy variable 4 59 intensity over time. The bold black line indicates the mean intensity over time. B) Box plots depicting the distribution of Immunoglobulin heavy variable 4 59 intensities at baseline, month 1, and month 2. Only AMD patients with measurements at all visits are included. The median, interquartile range, and outliers are displayed for each time point. Abbreviations: FDR, false discovery rate; ns, non−significant; * p < 0.05; ** p < 0.01; *** p < 0.001.

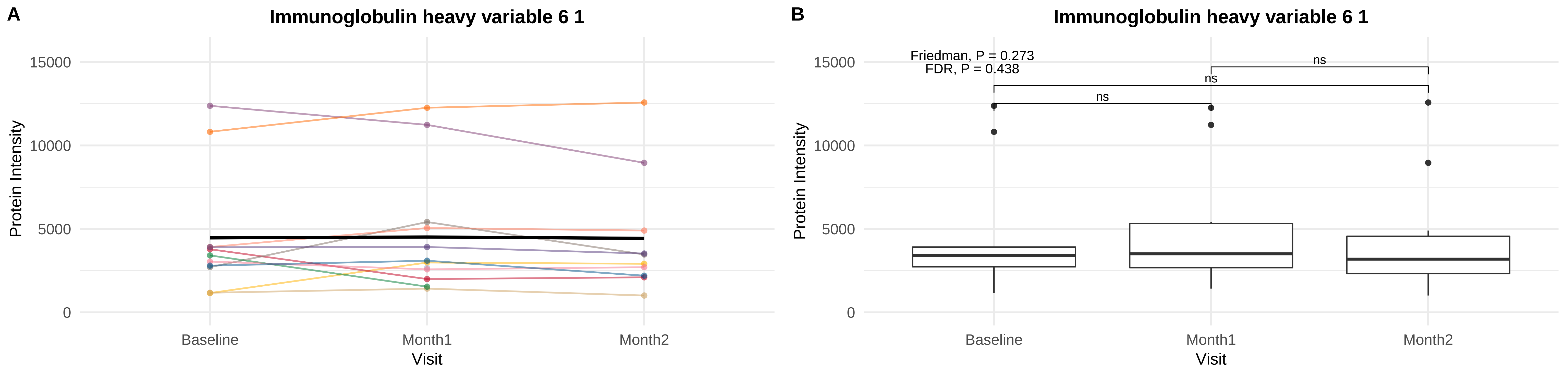

Supplementary Figure S 143

A) Line plot illustrating individual patient trajectories of Immunoglobulin heavy variable 6 1 intensity over time. The bold black line indicates the mean intensity over time. B) Box plots depicting the distribution of Immunoglobulin heavy variable 6 1 intensities at baseline, month 1, and month 2. Only AMD patients with measurements at all visits are included. The median, interquartile range, and outliers are displayed for each time point. Abbreviations: FDR, false discovery rate; ns, non−significant; * p < 0.05; ** p < 0.01; *** p < 0.001.

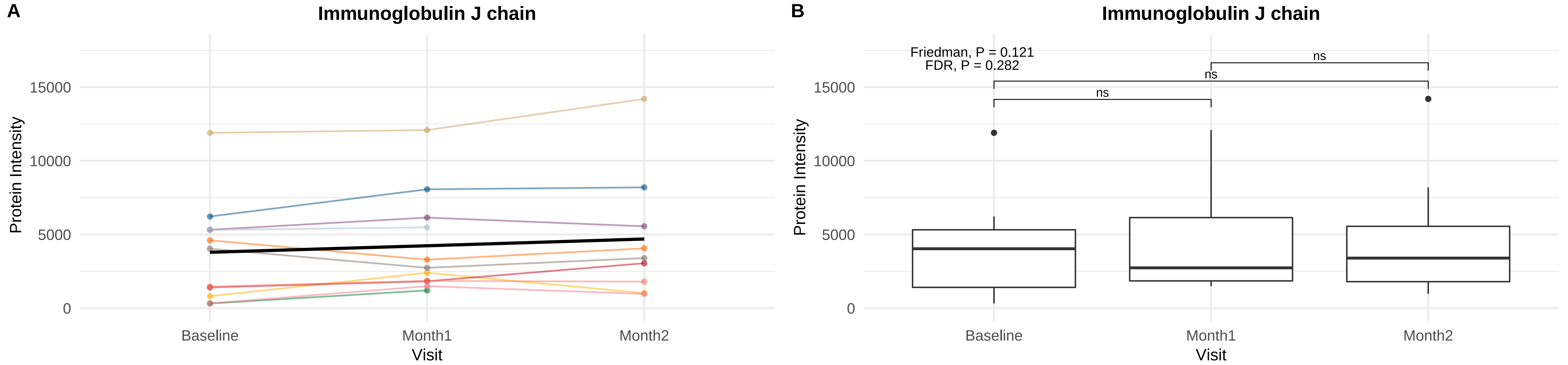

Supplementary Figure S 144

A) Line plot illustrating individual patient trajectories of Immunoglobulin J chain intensity over time. The bold black line indicates the mean intensity over time. B) Box plots depicting the distribution of Immunoglobulin J chain intensities at baseline, month 1, and month 2. Only AMD patients with measurements at all visits are included. The median, interquartile range, and outliers are displayed for each time point. Abbreviations: FDR, false discovery rate; ns, non−significant; * p < 0.05; ** p < 0.01; *** p < 0.001.

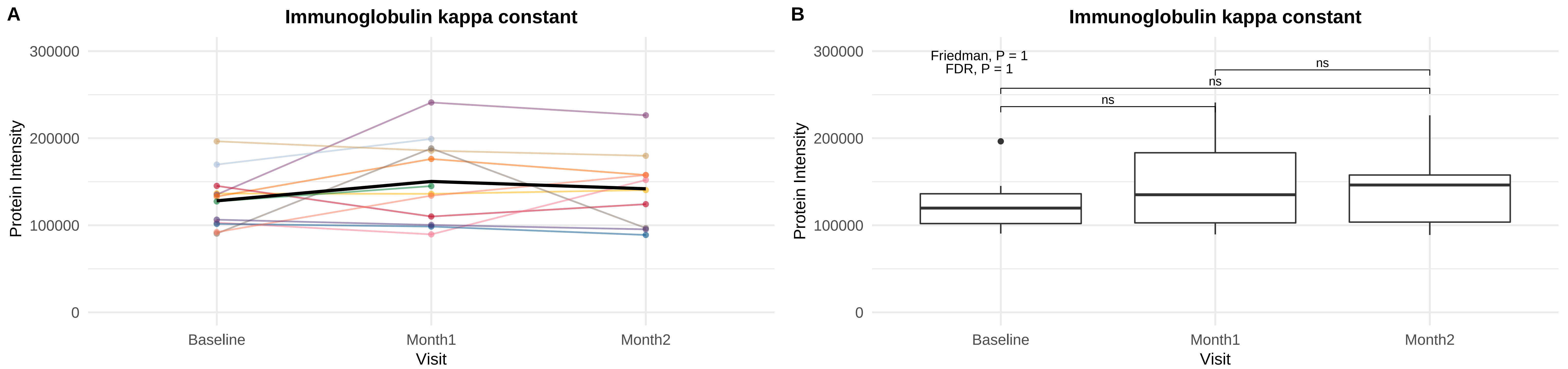

Supplementary Figure S 145

A) Line plot illustrating individual patient trajectories of Immunoglobulin kappa constant intensity over time. The bold black line indicates the mean intensity over time. B) Box plots depicting the distribution of Immunoglobulin kappa constant intensities at baseline, month 1, and month 2. Only AMD patients with measurements at all visits are included. The median, interquartile range, and outliers are displayed for each time point. Abbreviations: FDR, false discovery rate; ns, non−significant; * p < 0.05; ** p < 0.01; *** p < 0.001.

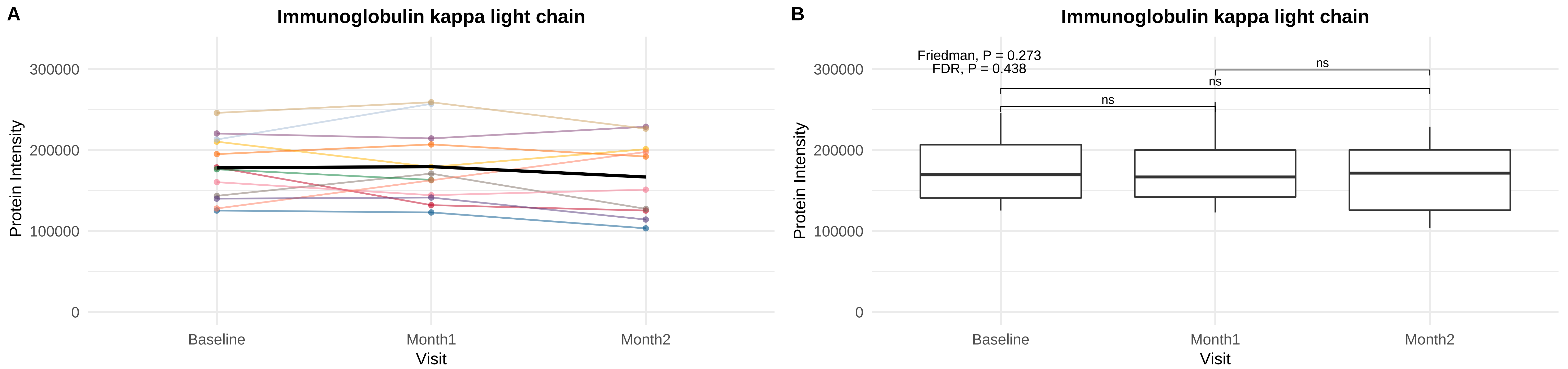

Supplementary Figure S 146

A) Line plot illustrating individual patient trajectories of Immunoglobulin kappa light chain intensity over time. The bold black line indicates the mean intensity over time. B) Box plots depicting the distribution of Immunoglobulin kappa light chain intensities at baseline, month 1, and month 2. Only AMD patients with measurements at all visits are included. The median, interquartile range, and outliers are displayed for each time point. Abbreviations: FDR, false discovery rate; ns, non−significant; * p < 0.05; ** p < 0.01; *** p < 0.001.

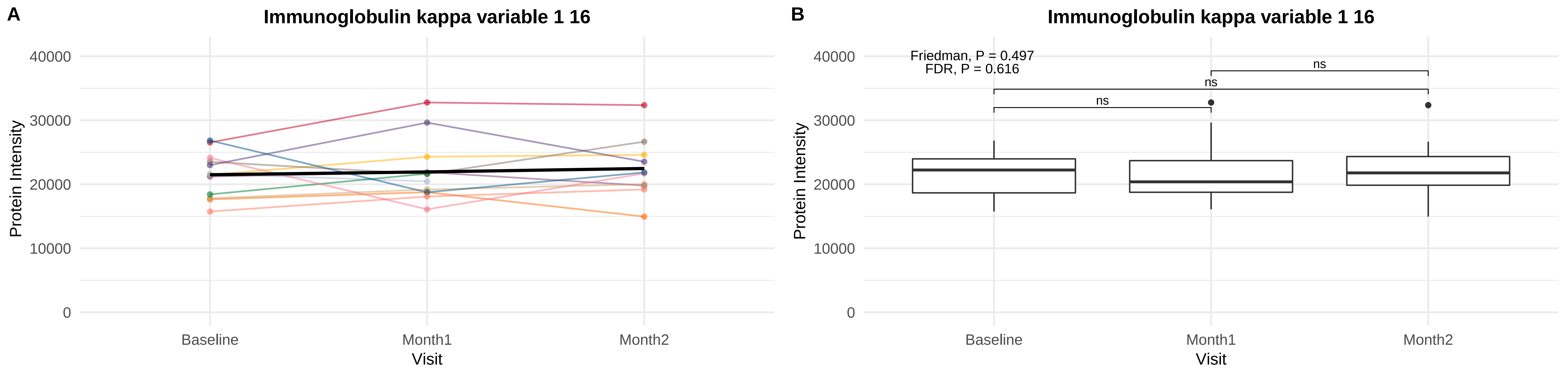

Supplementary Figure S 147

A) Line plot illustrating individual patient trajectories of Immunoglobulin kappa variable 1 16 intensity over time. The bold black line indicates the mean intensity over time. B) Box plots depicting the distribution of Immunoglobulin kappa variable 1 16 intensities at baseline, month 1, and month 2. Only AMD patients with measurements at all visits are included. The median, interquartile range, and outliers are displayed for each time point. Abbreviations: FDR, false discovery rate; ns, non−significant; * p < 0.05; ** p < 0.01; *** p < 0.001.

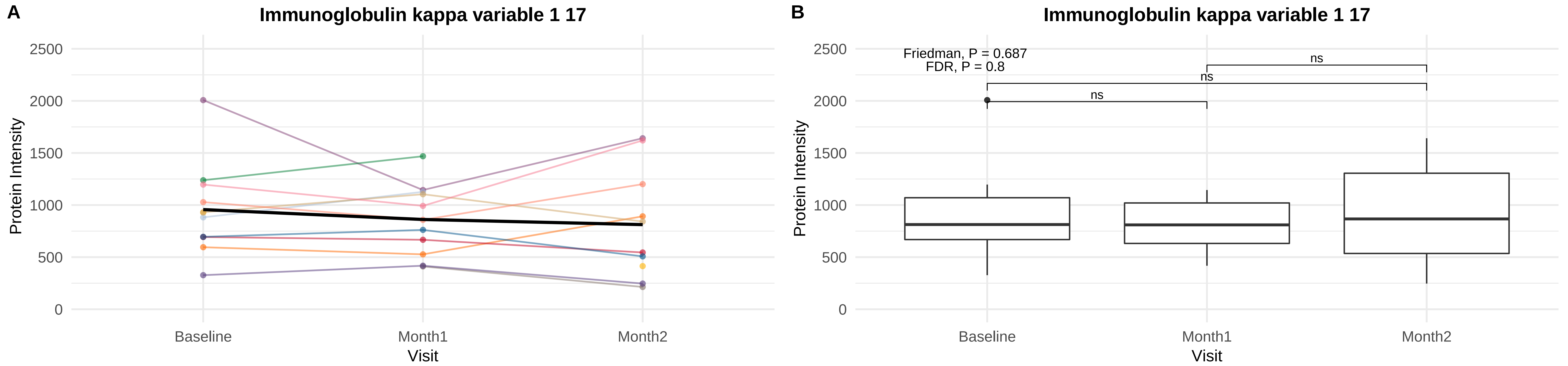

Supplementary Figure S 148

A) Line plot illustrating individual patient trajectories of Immunoglobulin kappa variable 1 17 intensity over time. The bold black line indicates the mean intensity over time. B) Box plots depicting the distribution of Immunoglobulin kappa variable 1 17 intensities at baseline, month 1, and month 2. Only AMD patients with measurements at all visits are included. The median, interquartile range, and outliers are displayed for each time point. Abbreviations: FDR, false discovery rate; ns, non−significant; * p < 0.05; ** p < 0.01; *** p < 0.001.

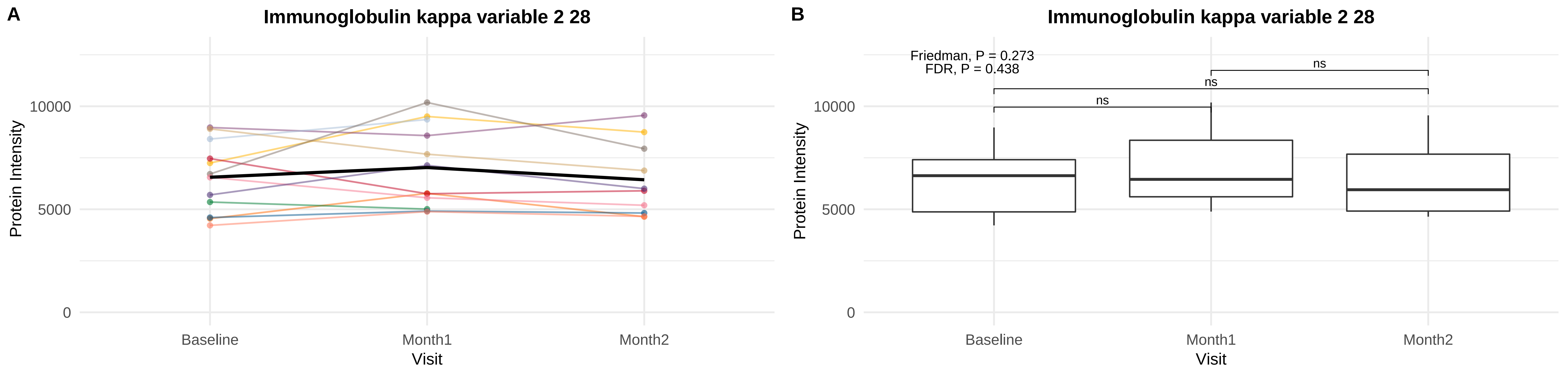

Supplementary Figure S 149

A) Line plot illustrating individual patient trajectories of Immunoglobulin kappa variable 2 28 intensity over time. The bold black line indicates the mean intensity over time. B) Box plots depicting the distribution of Immunoglobulin kappa variable 2 28 intensities at baseline, month 1, and month 2. Only AMD patients with measurements at all visits are included. The median, interquartile range, and outliers are displayed for each time point. Abbreviations: FDR, false discovery rate; ns, non−significant; * p < 0.05; ** p < 0.01; *** p < 0.001.

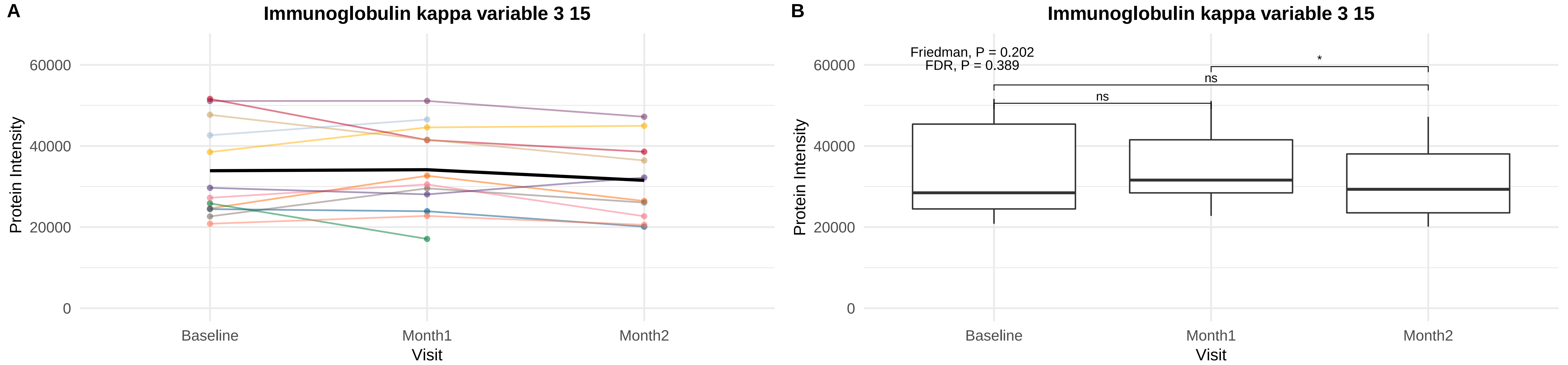

Supplementary Figure S 150

A) Line plot illustrating individual patient trajectories of Immunoglobulin kappa variable 3 15 intensity over time. The bold black line indicates the mean intensity over time. B) Box plots depicting the distribution of Immunoglobulin kappa variable 3 15 intensities at baseline, month 1, and month 2. Only AMD patients with measurements at all visits are included. The median, interquartile range, and outliers are displayed for each time point. Abbreviations: FDR, false discovery rate; ns, non−significant; * p < 0.05; ** p < 0.01; *** p < 0.001.

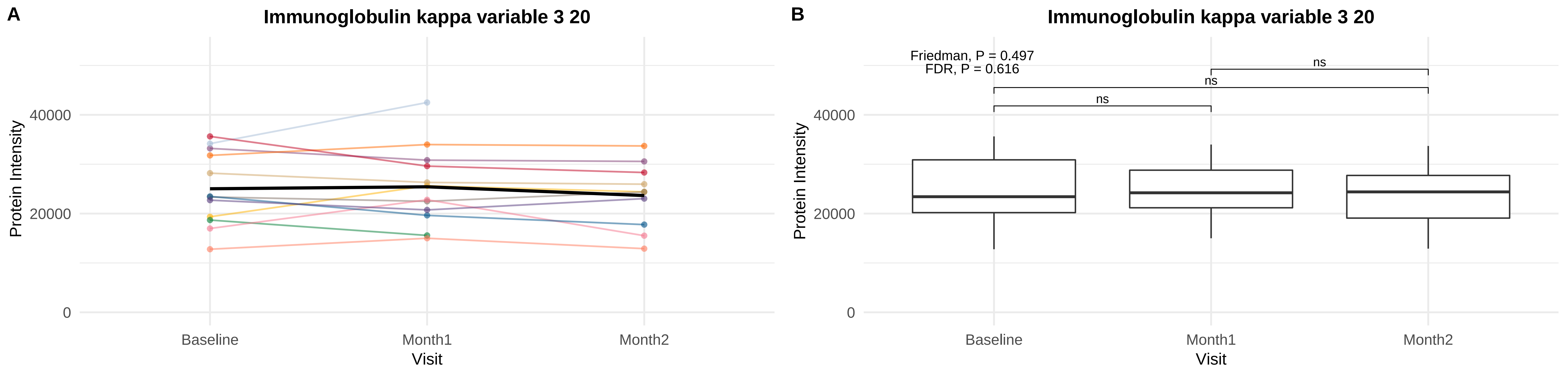

Supplementary Figure S 151

A) Line plot illustrating individual patient trajectories of Immunoglobulin kappa variable 3 20 intensity over time. The bold black line indicates the mean intensity over time. B) Box plots depicting the distribution of Immunoglobulin kappa variable 3 20 intensities at baseline, month 1, and month 2. Only AMD patients with measurements at all visits are included. The median, interquartile range, and outliers are displayed for each time point. Abbreviations: FDR, false discovery rate; ns, non−significant; * p < 0.05; ** p < 0.01; *** p < 0.001.

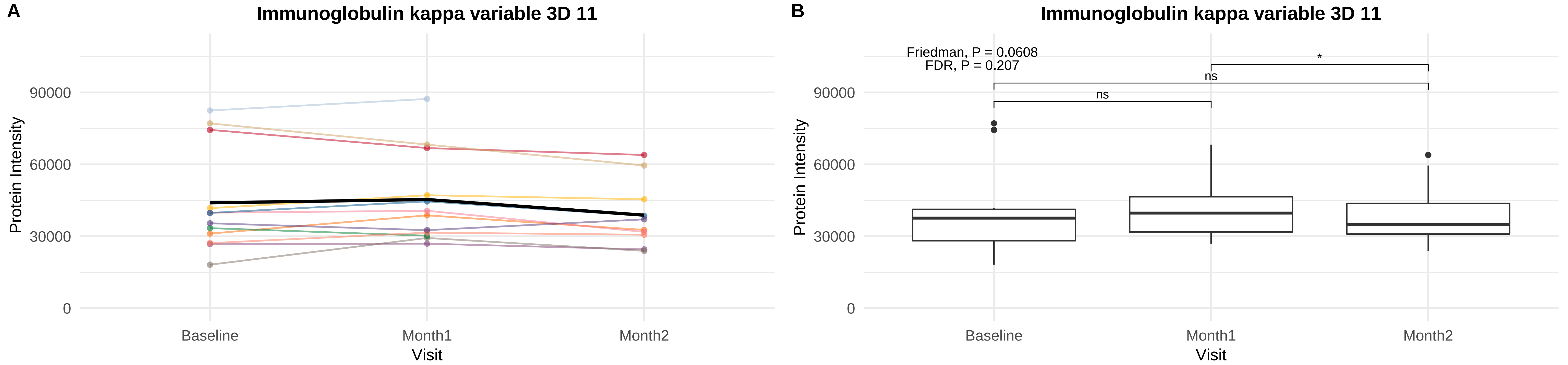

Supplementary Figure S 152

A) Line plot illustrating individual patient trajectories of Immunoglobulin kappa variable 3D 11 intensity over time. The bold black line indicates the mean intensity over time. B) Box plots depicting the distribution of Immunoglobulin kappa variable 3D 11 intensities at baseline, month 1, and month 2. Only AMD patients with measurements at all visits are included. The median, interquartile range, and outliers are displayed for each time point. Abbreviations: FDR, false discovery rate; ns, non−significant; * p < 0.05; ** p < 0.01; *** p < 0.001.

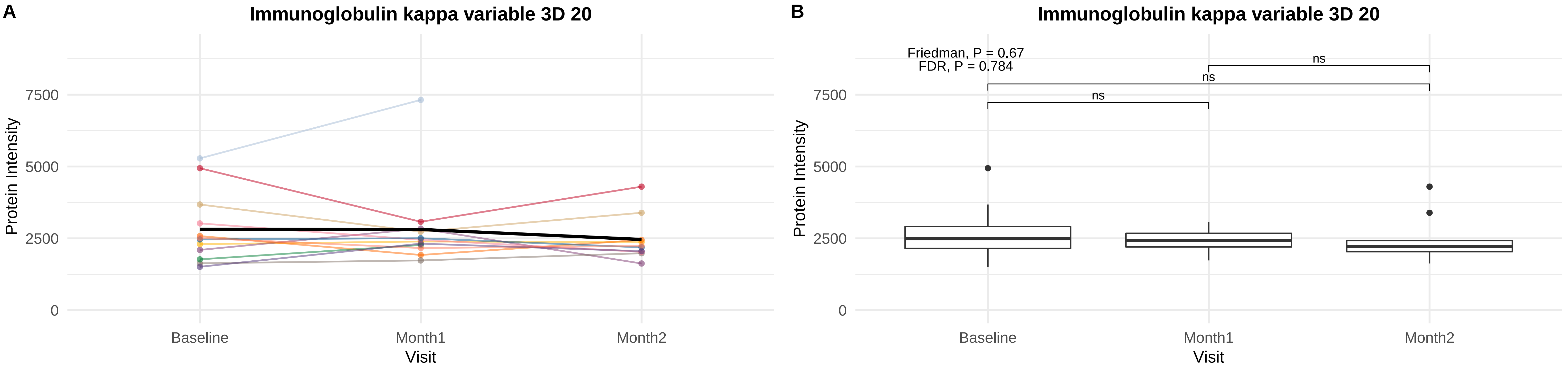

Supplementary Figure S 153

A) Line plot illustrating individual patient trajectories of Immunoglobulin kappa variable 3D 20 intensity over time. The bold black line indicates the mean intensity over time. B) Box plots depicting the distribution of Immunoglobulin kappa variable 3D 20 intensities at baseline, month 1, and month 2. Only AMD patients with measurements at all visits are included. The median, interquartile range, and outliers are displayed for each time point. Abbreviations: FDR, false discovery rate; ns, non−significant; * p < 0.05; ** p < 0.01; *** p < 0.001.

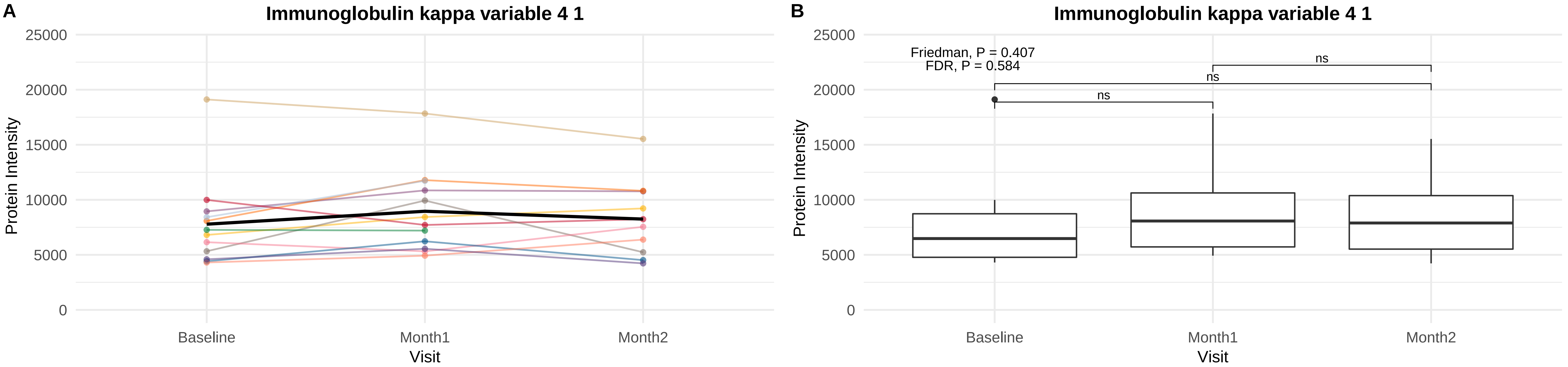

Supplementary Figure S 154

A) Line plot illustrating individual patient trajectories of Immunoglobulin kappa variable 4 1 intensity over time. The bold black line indicates the mean intensity over time. B) Box plots depicting the distribution of Immunoglobulin kappa variable 4 1 intensities at baseline, month 1, and month 2. Only AMD patients with measurements at all visits are included. The median, interquartile range, and outliers are displayed for each time point. Abbreviations: FDR, false discovery rate; ns, non−significant; * p < 0.05; ** p < 0.01; *** p < 0.001.

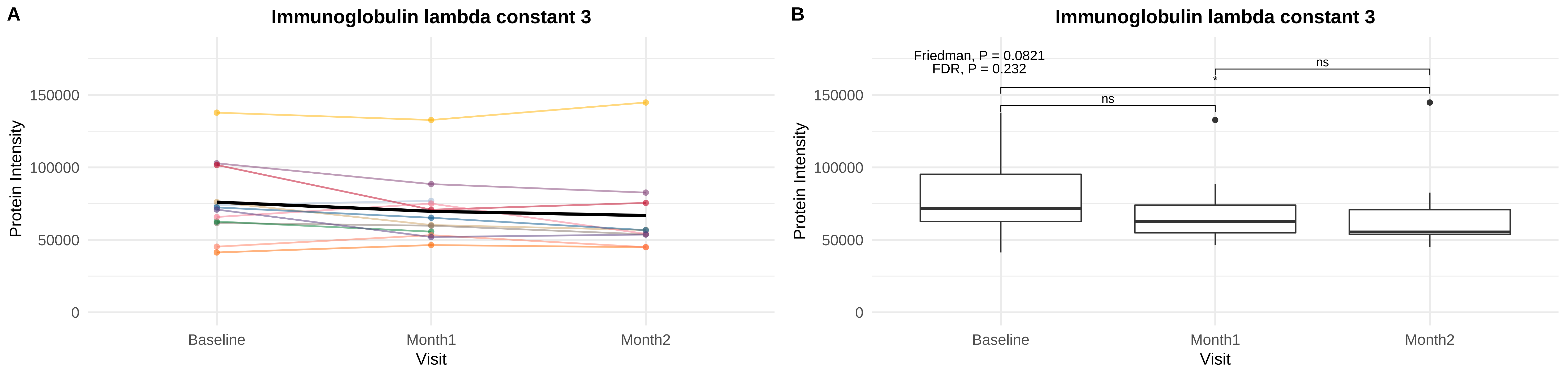

Supplementary Figure S 155

A) Line plot illustrating individual patient trajectories of Immunoglobulin lambda constant 3 intensity over time. The bold black line indicates the mean intensity over time. B) Box plots depicting the distribution of Immunoglobulin lambda constant 3 intensities at baseline, month 1, and month 2. Only AMD patients with measurements at all visits are included. The median, interquartile range, and outliers are displayed for each time point. Abbreviations: FDR, false discovery rate; ns, non−significant; * p < 0.05; ** p < 0.01; *** p < 0.001.

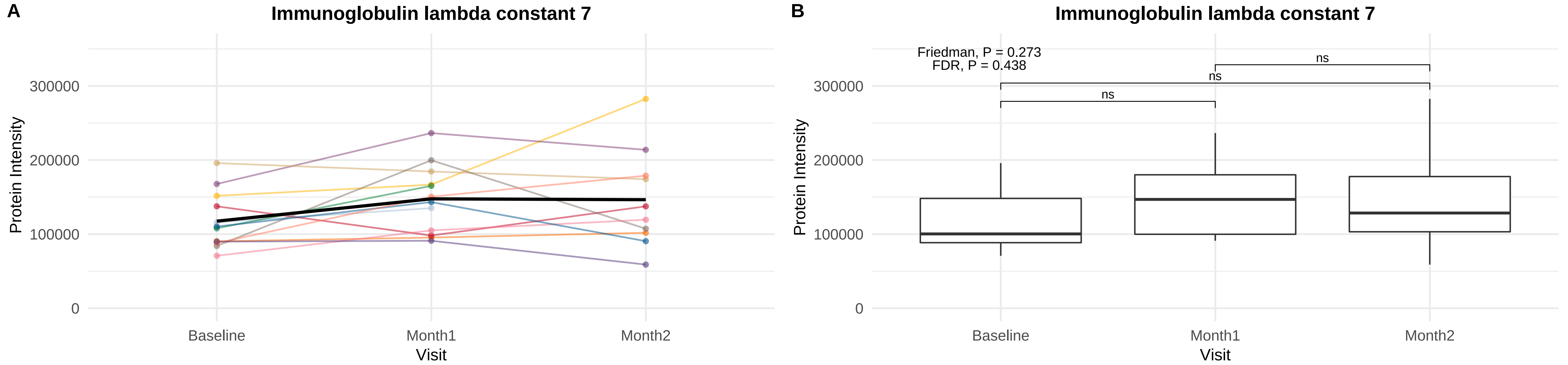

Supplementary Figure S 156

A) Line plot illustrating individual patient trajectories of Immunoglobulin lambda constant 7 intensity over time. The bold black line indicates the mean intensity over time. B) Box plots depicting the distribution of Immunoglobulin lambda constant 7 intensities at baseline, month 1, and month 2. Only AMD patients with measurements at all visits are included. The median, interquartile range, and outliers are displayed for each time point. Abbreviations: FDR, false discovery rate; ns, non−significant; * p < 0.05; ** p < 0.01; *** p < 0.001.

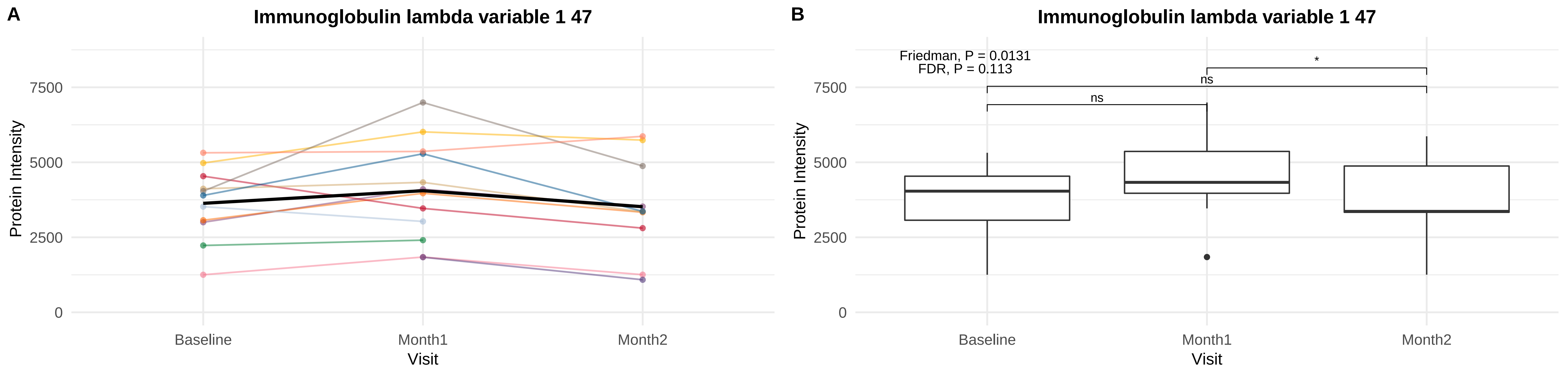

Supplementary Figure S 157

A) Line plot illustrating individual patient trajectories of Immunoglobulin lambda variable 1 47 intensity over time. The bold black line indicates the mean intensity over time. B) Box plots depicting the distribution of Immunoglobulin lambda variable 1 47 intensities at baseline, month 1, and month 2. Only AMD patients with measurements at all visits are included. The median, interquartile range, and outliers are displayed for each time point. Abbreviations: FDR, false discovery rate; ns, non−significant; * p < 0.05; ** p < 0.01; *** p < 0.001.

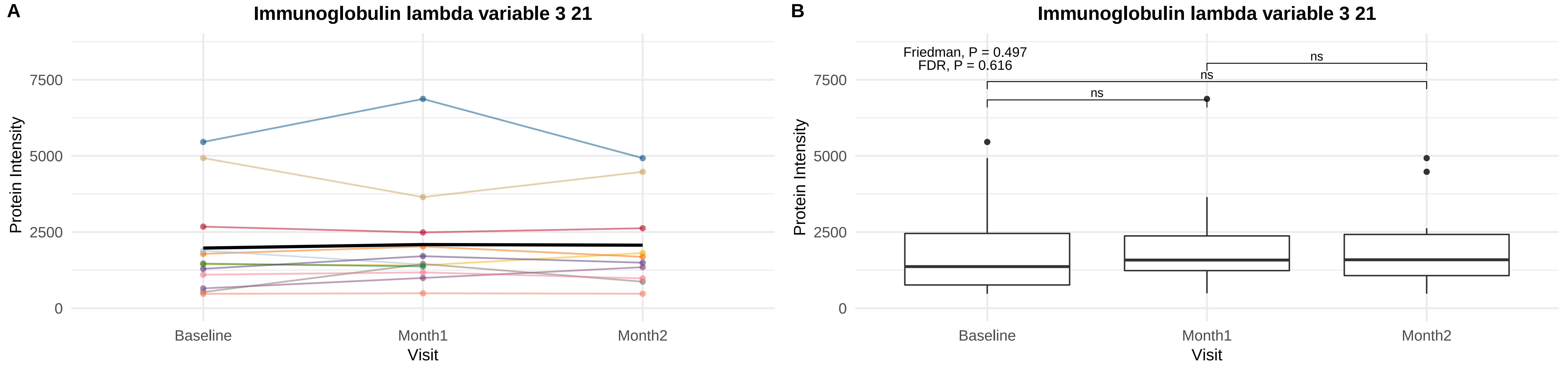

Supplementary Figure S 158

A) Line plot illustrating individual patient trajectories of Immunoglobulin lambda variable 3 21 intensity over time. The bold black line indicates the mean intensity over time. B) Box plots depicting the distribution of Immunoglobulin lambda variable 3 21 intensities at baseline, month 1, and month 2. Only AMD patients with measurements at all visits are included. The median, interquartile range, and outliers are displayed for each time point. Abbreviations: FDR, false discovery rate; ns, non−significant; * p < 0.05; ** p < 0.01; *** p < 0.001.

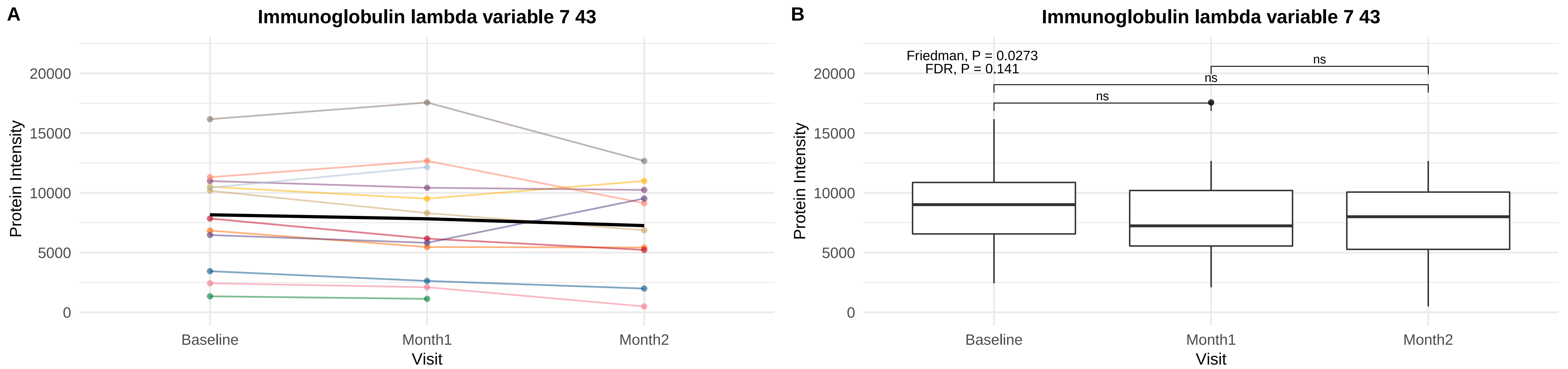

Supplementary Figure S 159

A) Line plot illustrating individual patient trajectories of Immunoglobulin lambda variable 7 43 intensity over time. The bold black line indicates the mean intensity over time. B) Box plots depicting the distribution of Immunoglobulin lambda variable 7 43 intensities at baseline, month 1, and month 2. Only AMD patients with measurements at all visits are included. The median, interquartile range, and outliers are displayed for each time point. Abbreviations: FDR, false discovery rate; ns, non−significant; * p < 0.05; ** p < 0.01; *** p < 0.001.

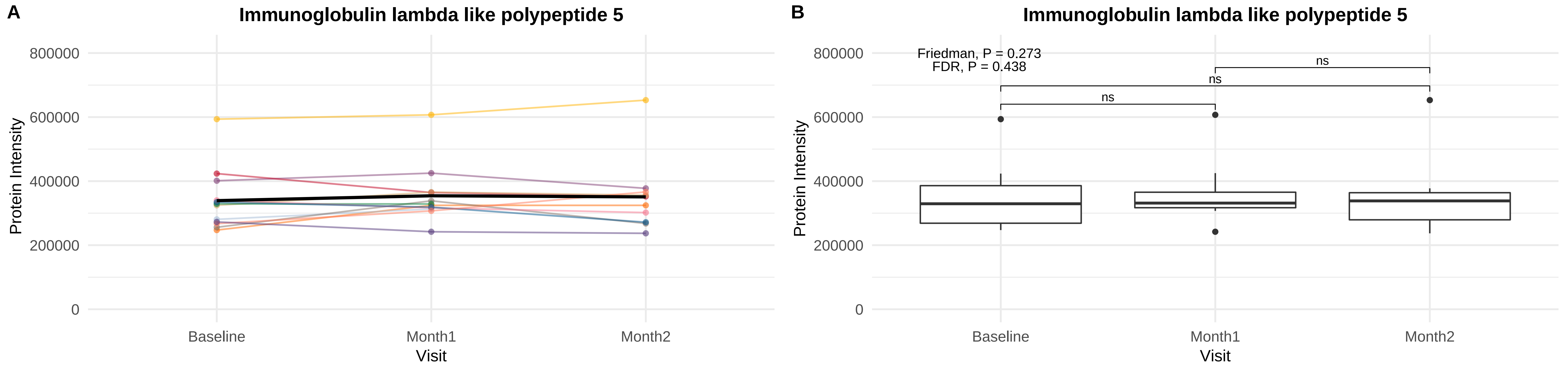

Supplementary Figure S 160

A) Line plot illustrating individual patient trajectories of Immunoglobulin lambda like polypeptide 5 intensity over time. The bold black line indicates the mean intensity over time. B) Box plots depicting the distribution of Immunoglobulin lambda like polypeptide 5 intensities at baseline, month 1, and month 2. Only AMD patients with measurements at all visits are included. The median, interquartile range, and outliers are displayed for each time point. Abbreviations: FDR, false discovery rate; ns, non−significant; * p < 0.05; ** p < 0.01; *** p < 0.001.

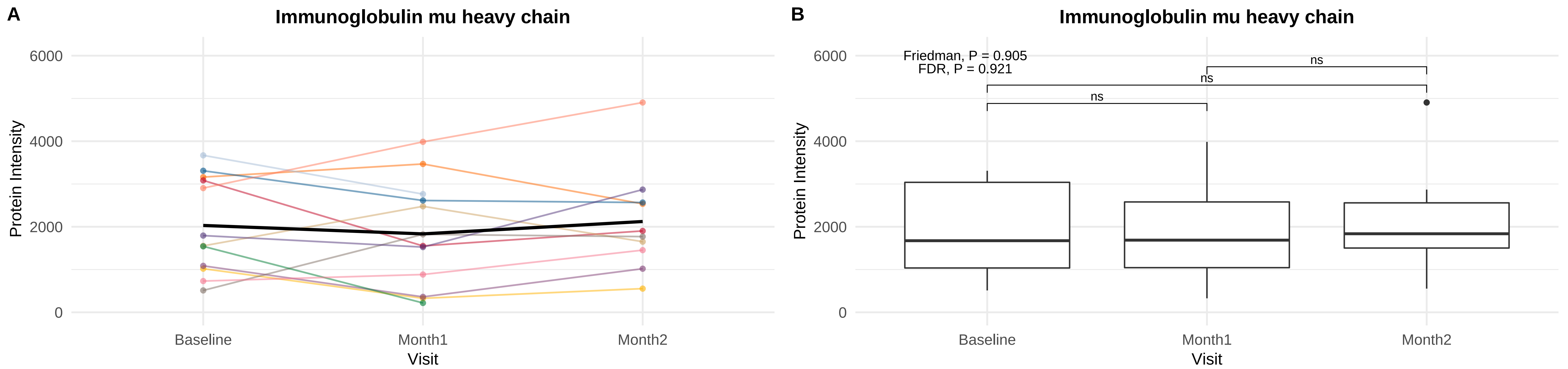

Supplementary Figure S 161

A) Line plot illustrating individual patient trajectories of Immunoglobulin mu heavy chain intensity over time. The bold black line indicates the mean intensity over time. B) Box plots depicting the distribution of Immunoglobulin mu heavy chain intensities at baseline, month 1, and month 2. Only AMD patients with measurements at all visits are included. The median, interquartile range, and outliers are displayed for each time point. Abbreviations: FDR, false discovery rate; ns, non−significant; * p < 0.05; ** p < 0.01; *** p < 0.001.

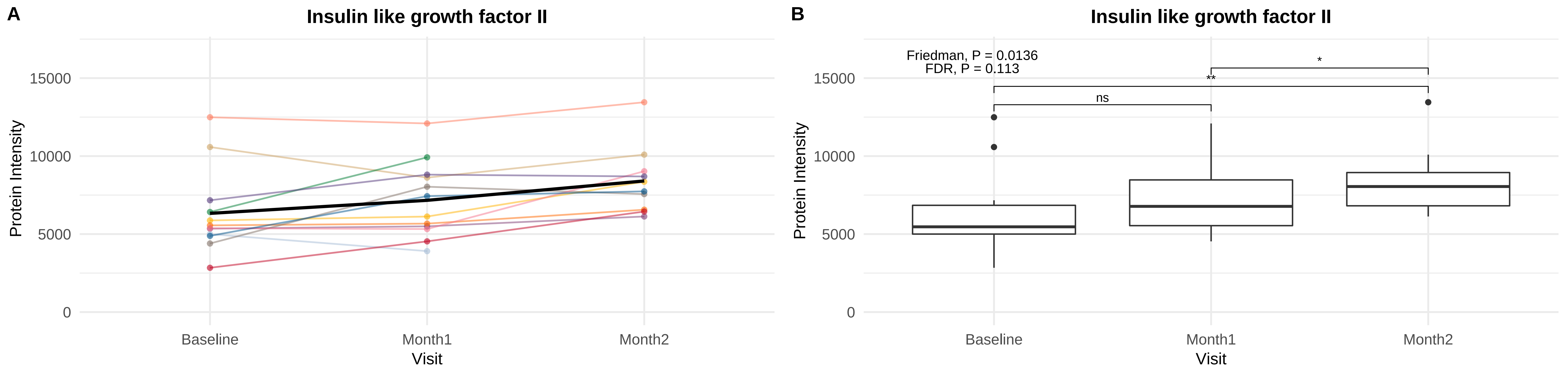

Supplementary Figure S 162

A) Line plot illustrating individual patient trajectories of Insulin like growth factor II intensity over time. The bold black line indicates the mean intensity over time. B) Box plots depicting the distribution of Insulin like growth factor II intensities at baseline, month 1, and month 2. Only AMD patients with measurements at all visits are included. The median, interquartile range, and outliers are displayed for each time point. Abbreviations: FDR, false discovery rate; ns, non−significant; * p < 0.05; ** p < 0.01; *** p < 0.001.

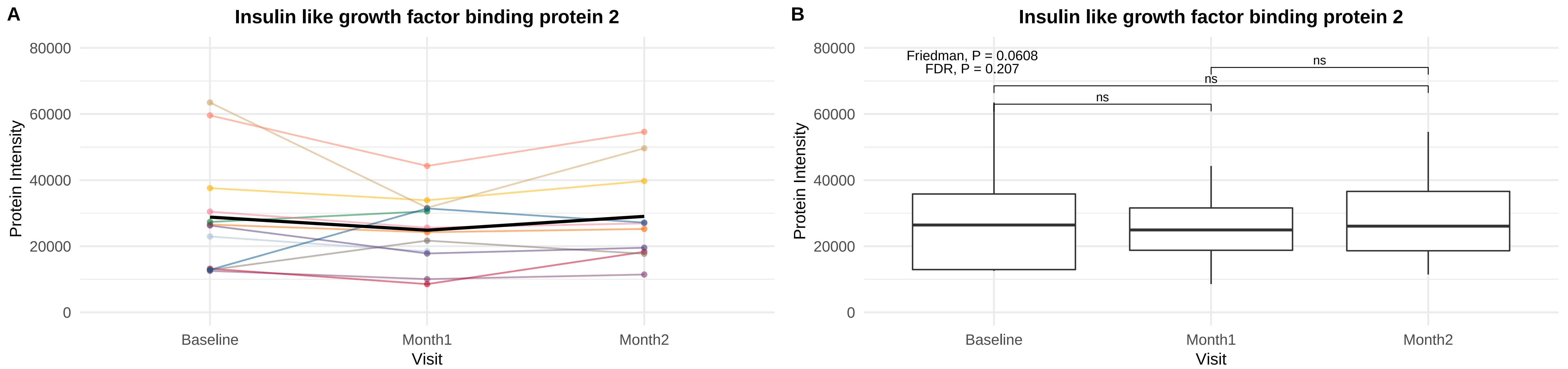

Supplementary Figure S 163

A) Line plot illustrating individual patient trajectories of Insulin like growth factor binding protein 2 intensity over time. The bold black line indicates the mean intensity over time. B) Box plots depicting the distribution of Insulin like growth factor binding protein 2 intensities at baseline, month 1, and month 2. Only AMD patients with measurements at all visits are included. The median, interquartile range, and outliers are displayed for each time point. Abbreviations: FDR, false discovery rate; ns, non−significant; * p < 0.05; ** p < 0.01; *** p < 0.001.

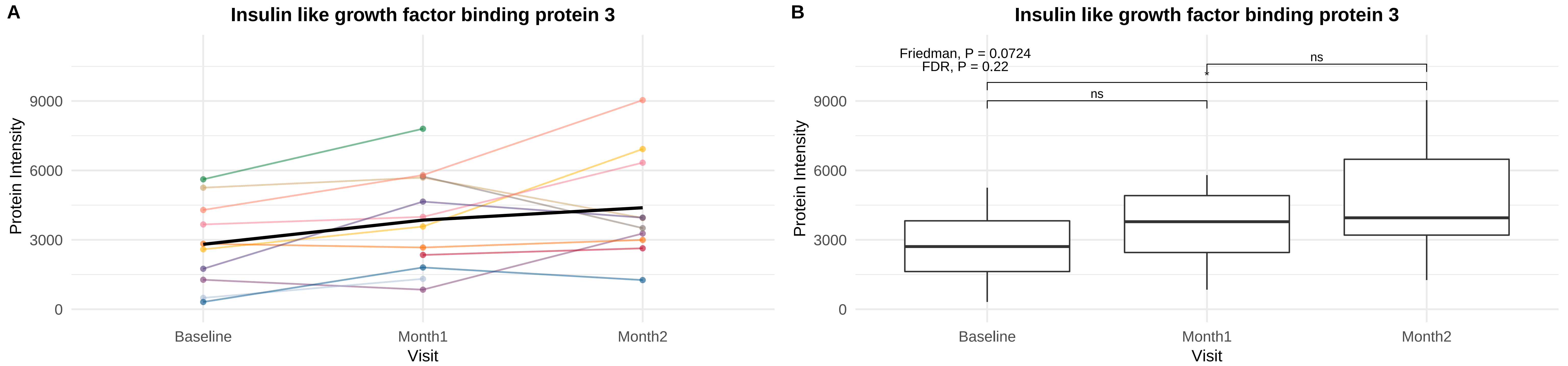

Supplementary Figure S 164

A) Line plot illustrating individual patient trajectories of Insulin like growth factor binding protein 3 intensity over time. The bold black line indicates the mean intensity over time. B) Box plots depicting the distribution of Insulin like growth factor binding protein 3 intensities at baseline, month 1, and month 2. Only AMD patients with measurements at all visits are included. The median, interquartile range, and outliers are displayed for each time point. Abbreviations: FDR, false discovery rate; ns, non−significant; * p < 0.05; ** p < 0.01; *** p < 0.001.

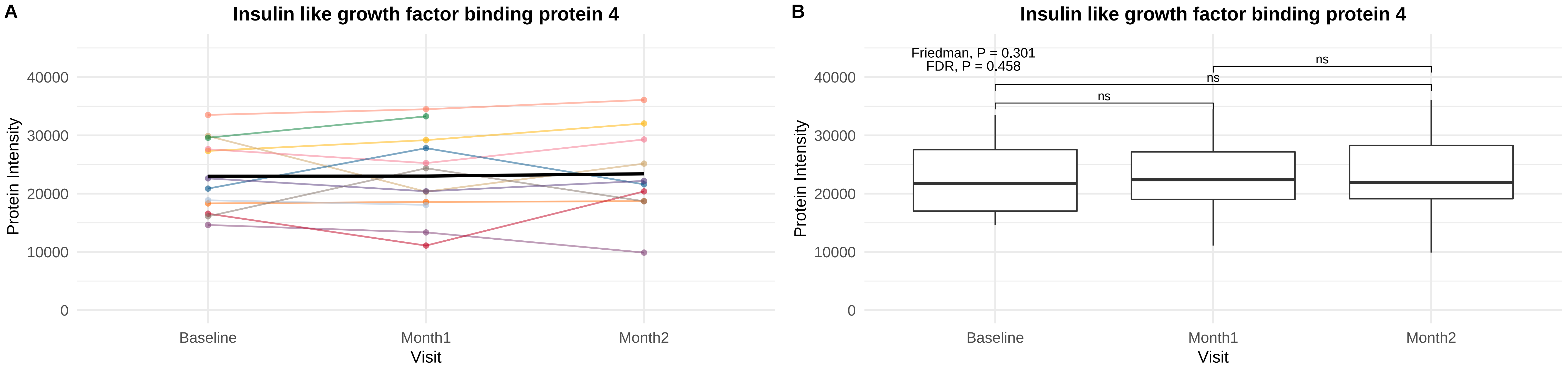

Supplementary Figure S 165

A) Line plot illustrating individual patient trajectories of Insulin like growth factor binding protein 4 intensity over time. The bold black line indicates the mean intensity over time. B) Box plots depicting the distribution of Insulin like growth factor binding protein 4 intensities at baseline, month 1, and month 2. Only AMD patients with measurements at all visits are included. The median, interquartile range, and outliers are displayed for each time point. Abbreviations: FDR, false discovery rate; ns, non−significant; * p < 0.05; ** p < 0.01; *** p < 0.001.

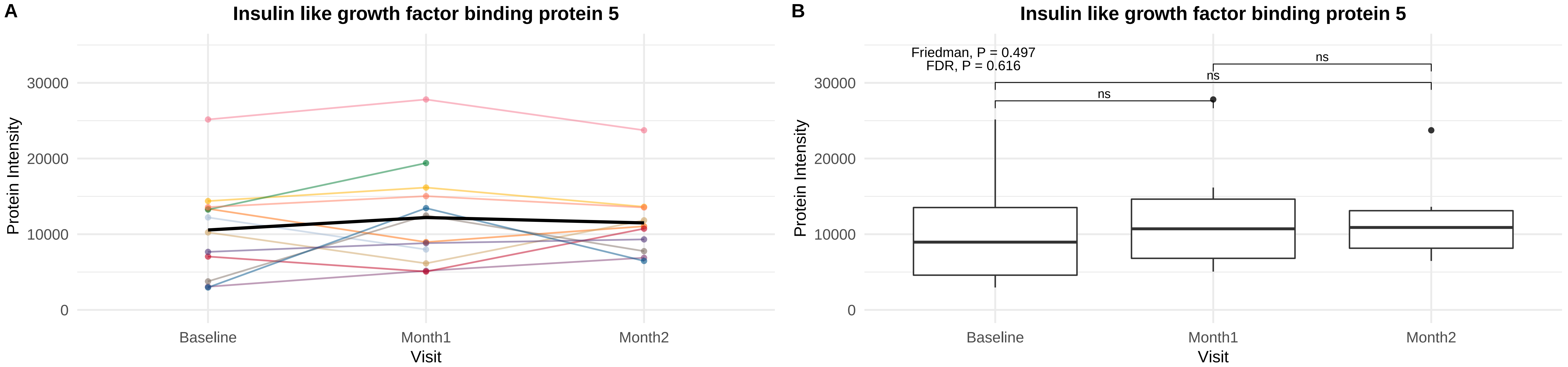

Supplementary Figure S 166

A) Line plot illustrating individual patient trajectories of Insulin like growth factor binding protein 5 intensity over time. The bold black line indicates the mean intensity over time. B) Box plots depicting the distribution of Insulin like growth factor binding protein 5 intensities at baseline, month 1, and month 2. Only AMD patients with measurements at all visits are included. The median, interquartile range, and outliers are displayed for each time point. Abbreviations: FDR, false discovery rate; ns, non−significant; * p < 0.05; ** p < 0.01; *** p < 0.001.

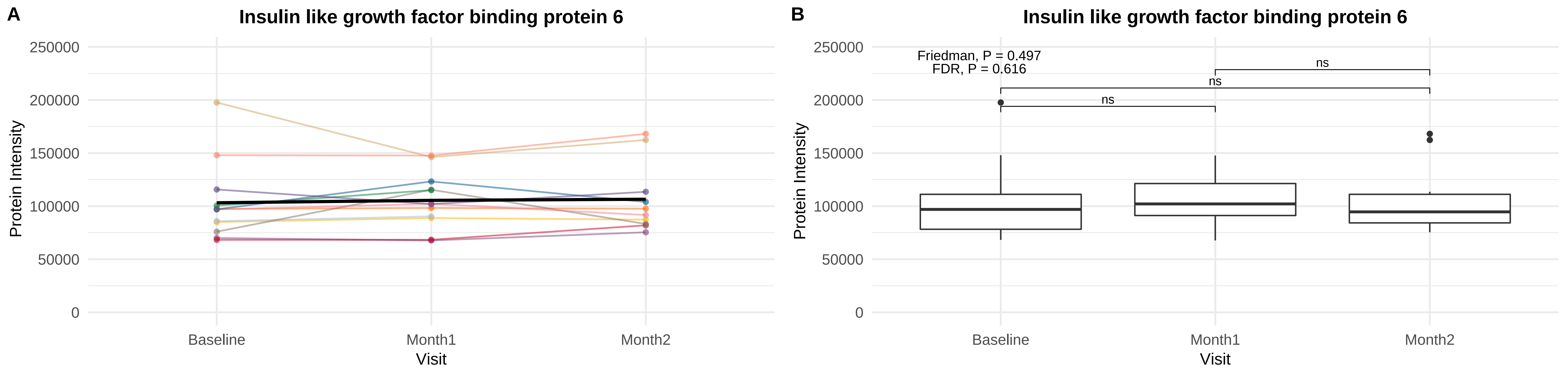

Supplementary Figure S 167

A) Line plot illustrating individual patient trajectories of Insulin like growth factor binding protein 6 intensity over time. The bold black line indicates the mean intensity over time. B) Box plots depicting the distribution of Insulin like growth factor binding protein 6 intensities at baseline, month 1, and month 2. Only AMD patients with measurements at all visits are included. The median, interquartile range, and outliers are displayed for each time point. Abbreviations: FDR, false discovery rate; ns, non−significant; * p < 0.05; ** p < 0.01; *** p < 0.001.

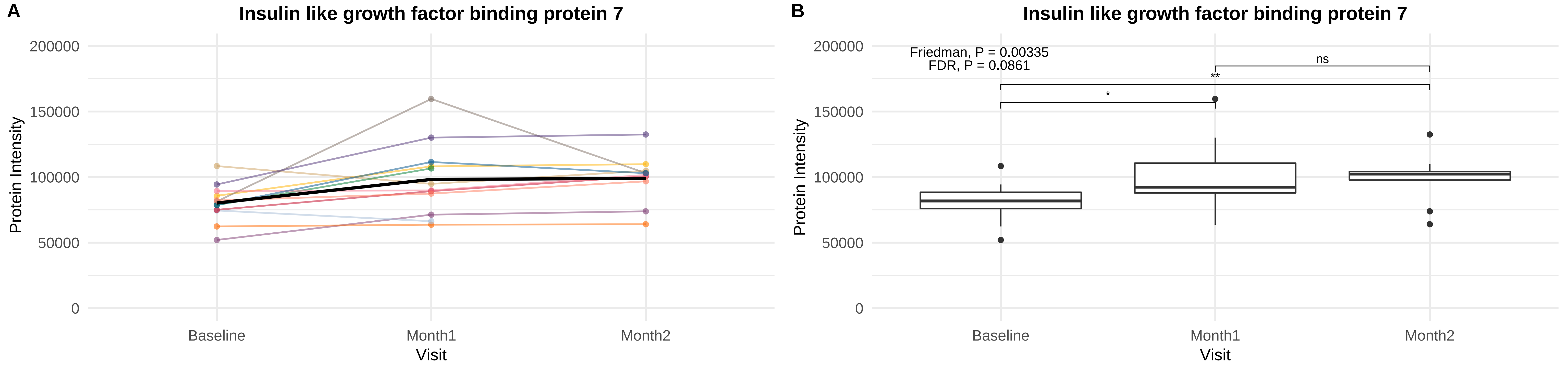

Supplementary Figure S 168

A) Line plot illustrating individual patient trajectories of Insulin like growth factor binding protein 7 intensity over time. The bold black line indicates the mean intensity over time. B) Box plots depicting the distribution of Insulin like growth factor binding protein 7 intensities at baseline, month 1, and month 2. Only AMD patients with measurements at all visits are included. The median, interquartile range, and outliers are displayed for each time point. Abbreviations: FDR, false discovery rate; ns, non−significant; * p < 0.05; ** p < 0.01; *** p < 0.001.

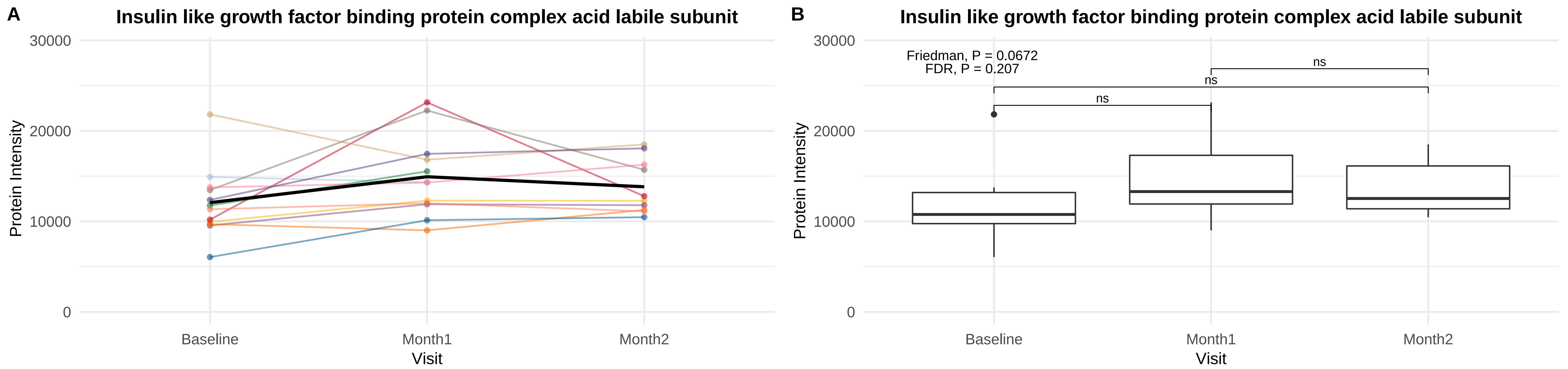

Supplementary Figure S 169

A) Line plot illustrating individual patient trajectories of Insulin like growth factor binding protein complex acid labile subunit intensity over time. The bold black line indicates the mean intensity over time. B) Box plots depicting the distribution of Insulin like growth factor binding protein complex acid labile subunit intensities at baseline, month 1, and month 2. Only AMD patients with measurements at all visits are included. The median, interquartile range, and outliers are displayed for each time point. Abbreviations: FDR, false discovery rate; ns, non−significant; * p < 0.05; ** p < 0.01; *** p < 0.001.

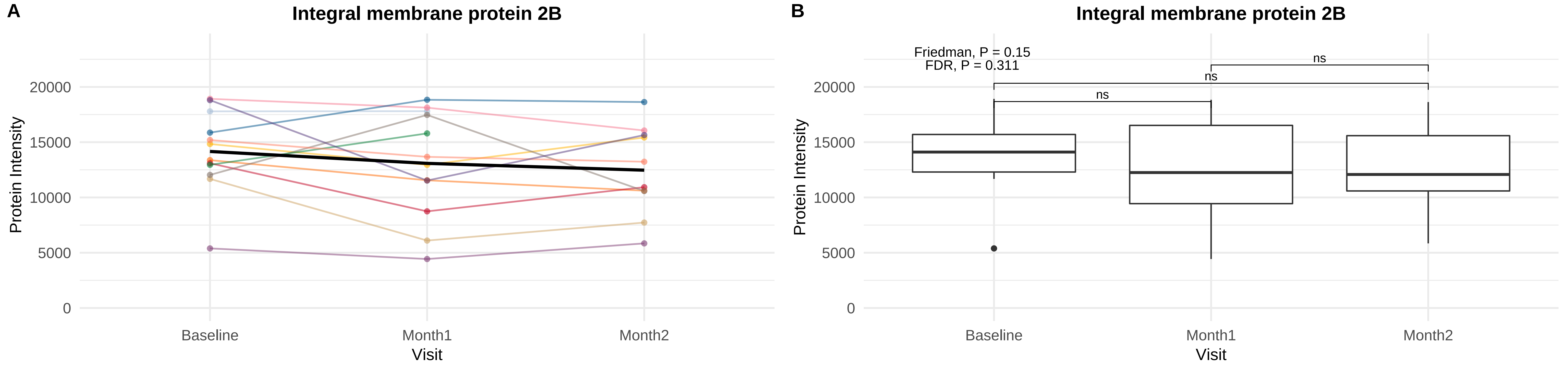

Supplementary Figure S 170

A) Line plot illustrating individual patient trajectories of Integral membrane protein 2B intensity over time. The bold black line indicates the mean intensity over time. B) Box plots depicting the distribution of Integral membrane protein 2B intensities at baseline, month 1, and month 2. Only AMD patients with measurements at all visits are included. The median, interquartile range, and outliers are displayed for each time point. Abbreviations: FDR, false discovery rate; ns, non−significant; * p < 0.05; ** p < 0.01; *** p < 0.001.

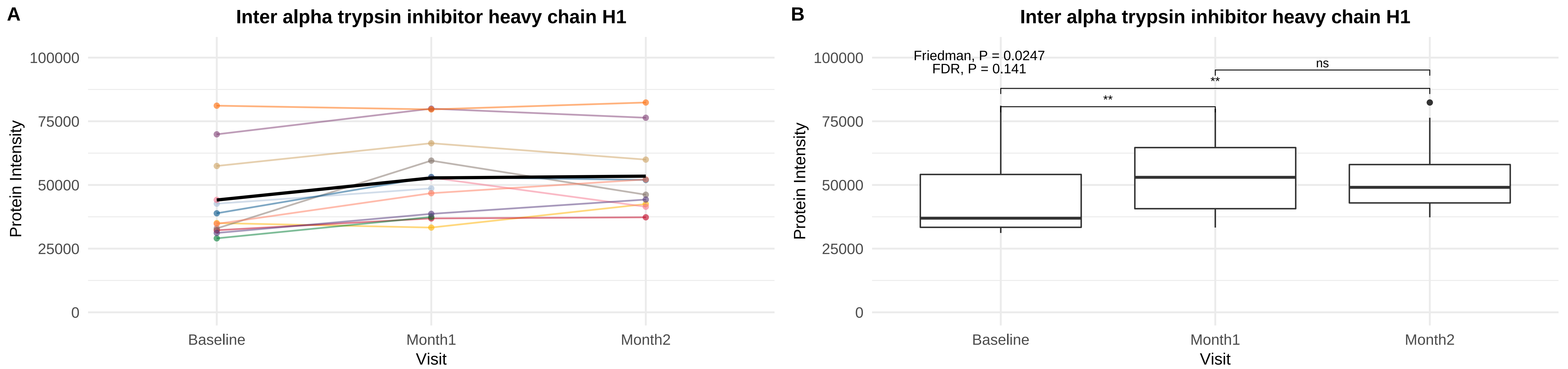

Supplementary Figure S 171

A) Line plot illustrating individual patient trajectories of Inter alpha trypsin inhibitor heavy chain H1 intensity over time. The bold black line indicates the mean intensity over time. B) Box plots depicting the distribution of Inter alpha trypsin inhibitor heavy chain H1 intensities at baseline, month 1, and month 2. Only AMD patients with measurements at all visits are included. The median, interquartile range, and outliers are displayed for each time point. Abbreviations: FDR, false discovery rate; ns, non−significant; * p < 0.05; ** p < 0.01; *** p < 0.001.

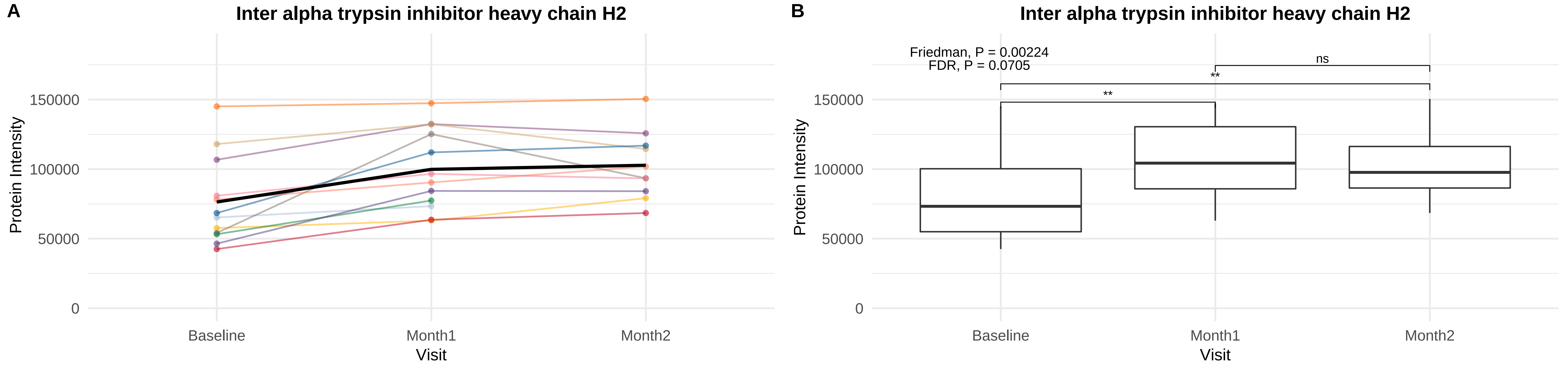

Supplementary Figure S 172

A) Line plot illustrating individual patient trajectories of Inter alpha trypsin inhibitor heavy chain H2 intensity over time. The bold black line indicates the mean intensity over time. B) Box plots depicting the distribution of Inter alpha trypsin inhibitor heavy chain H2 intensities at baseline, month 1, and month 2. Only AMD patients with measurements at all visits are included. The median, interquartile range, and outliers are displayed for each time point. Abbreviations: FDR, false discovery rate; ns, non−significant; * p < 0.05; ** p < 0.01; *** p < 0.001.

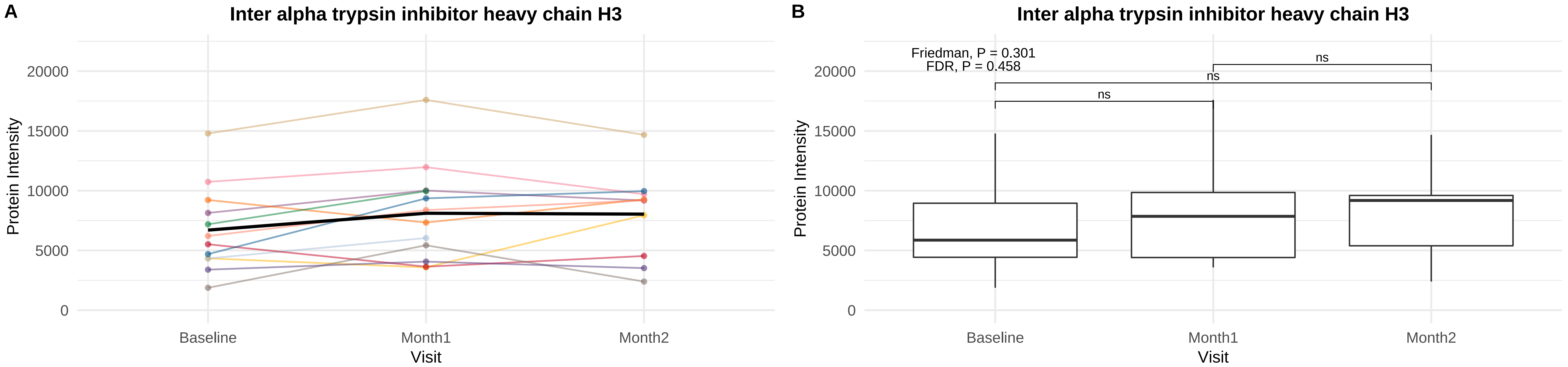

Supplementary Figure S 173

A) Line plot illustrating individual patient trajectories of Inter alpha trypsin inhibitor heavy chain H3 intensity over time. The bold black line indicates the mean intensity over time. B) Box plots depicting the distribution of Inter alpha trypsin inhibitor heavy chain H3 intensities at baseline, month 1, and month 2. Only AMD patients with measurements at all visits are included. The median, interquartile range, and outliers are displayed for each time point. Abbreviations: FDR, false discovery rate; ns, non−significant; * p < 0.05; ** p < 0.01; *** p < 0.001.

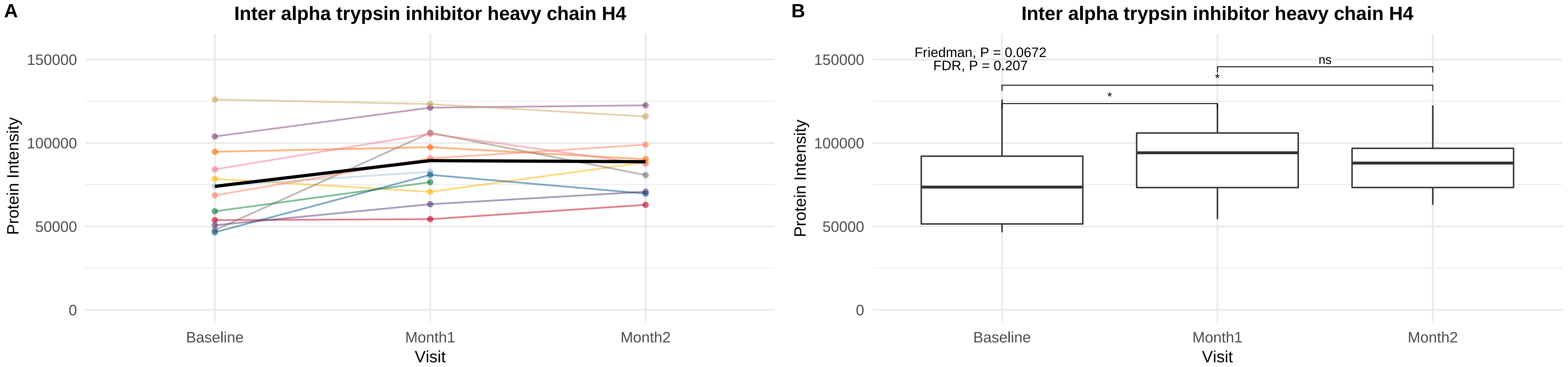

Supplementary Figure S 174

A) Line plot illustrating individual patient trajectories of Inter alpha trypsin inhibitor heavy chain H4 intensity over time. The bold black line indicates the mean intensity over time. B) Box plots depicting the distribution of Inter alpha trypsin inhibitor heavy chain H4 intensities at baseline, month 1, and month 2. Only AMD patients with measurements at all visits are included. The median, interquartile range, and outliers are displayed for each time point. Abbreviations: FDR, false discovery rate; ns, non−significant; * p < 0.05; ** p < 0.01; *** p < 0.001.

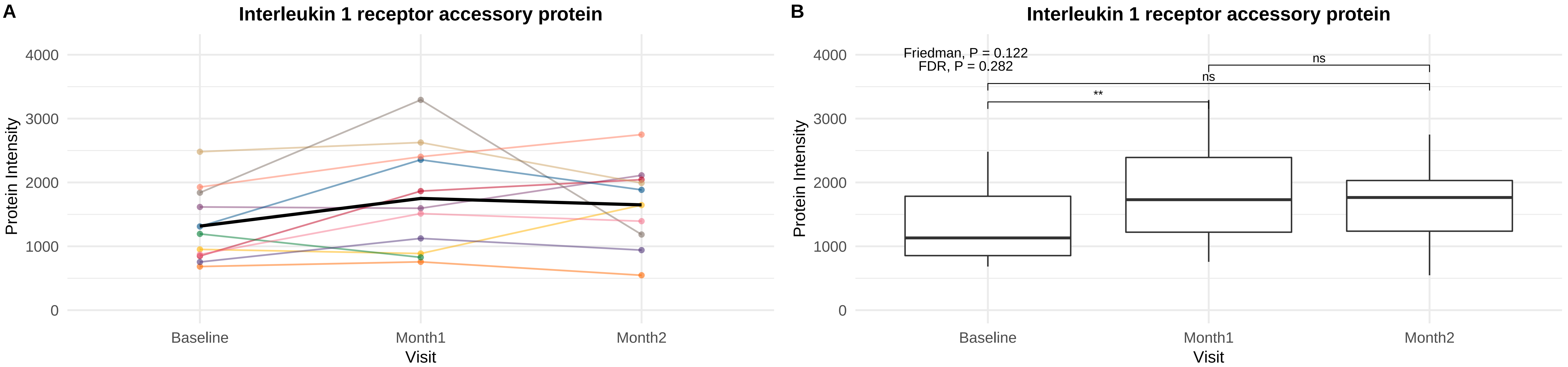

Supplementary Figure S 175

A) Line plot illustrating individual patient trajectories of Interleukin 1 receptor accessory protein intensity over time. The bold black line indicates the mean intensity over time. B) Box plots depicting the distribution of Interleukin 1 receptor accessory protein intensities at baseline, month 1, and month 2. Only AMD patients with measurements at all visits are included. The median, interquartile range, and outliers are displayed for each time point. Abbreviations: FDR, false discovery rate; ns, non−significant; * p < 0.05; ** p < 0.01; *** p < 0.001.

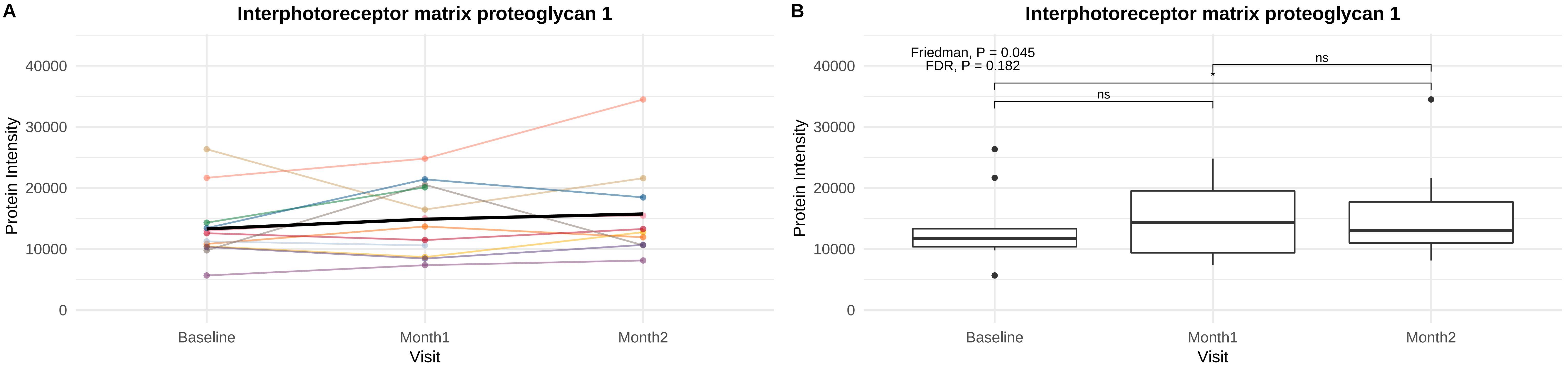

Supplementary Figure S 176

A) Line plot illustrating individual patient trajectories of Interphotoreceptor matrix proteoglycan 1 intensity over time. The bold black line indicates the mean intensity over time. B) Box plots depicting the distribution of Interphotoreceptor matrix proteoglycan 1 intensities at baseline, month 1, and month 2. Only AMD patients with measurements at all visits are included. The median, interquartile range, and outliers are displayed for each time point. Abbreviations: FDR, false discovery rate; ns, non−significant; * p < 0.05; ** p < 0.01; *** p < 0.001.

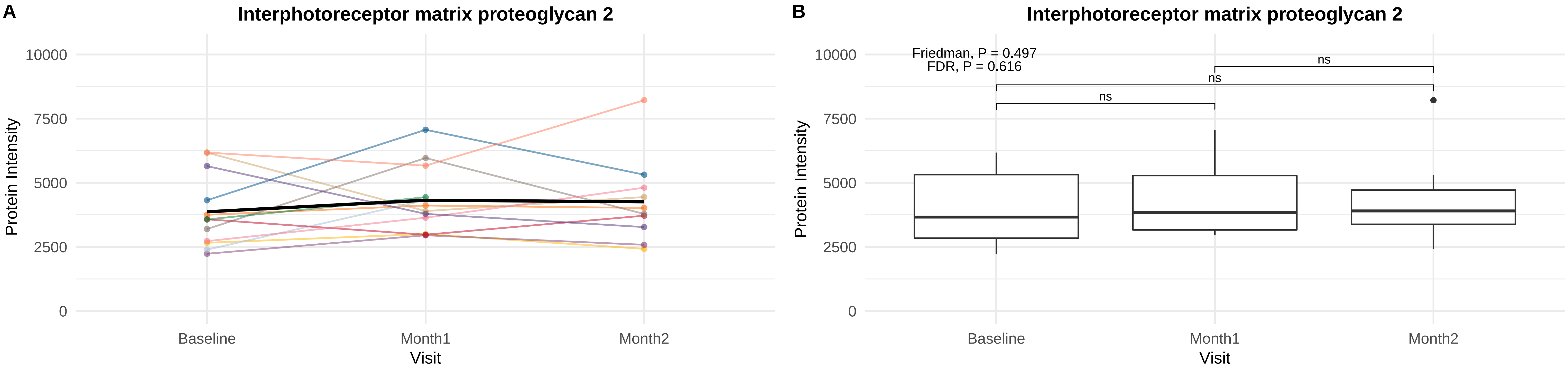

Supplementary Figure S 177

A) Line plot illustrating individual patient trajectories of Interphotoreceptor matrix proteoglycan 2 intensity over time. The bold black line indicates the mean intensity over time. B) Box plots depicting the distribution of Interphotoreceptor matrix proteoglycan 2 intensities at baseline, month 1, and month 2. Only AMD patients with measurements at all visits are included. The median, interquartile range, and outliers are displayed for each time point. Abbreviations: FDR, false discovery rate; ns, non−significant; * p < 0.05; ** p < 0.01; *** p < 0.001.

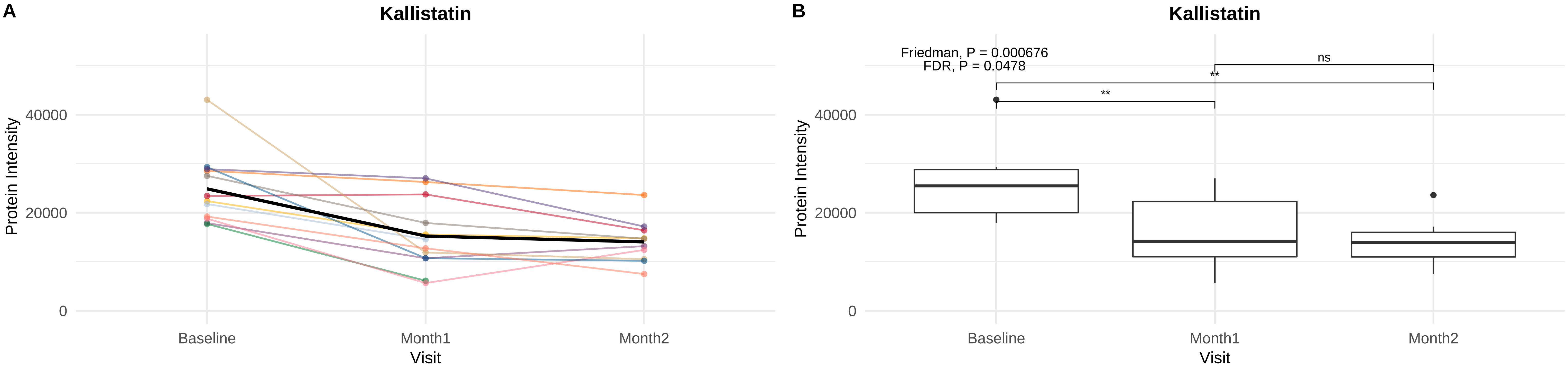

Supplementary Figure S 178

A) Line plot illustrating individual patient trajectories of Kallistatin intensity over time. The bold black line indicates the mean intensity over time. B) Box plots depicting the distribution of Kallistatin intensities at baseline, month 1, and month 2. Only AMD patients with measurements at all visits are included. The median, interquartile range, and outliers are displayed for each time point. Abbreviations: FDR, false discovery rate; ns, non−significant; * p < 0.05; ** p < 0.01; *** p < 0.001.

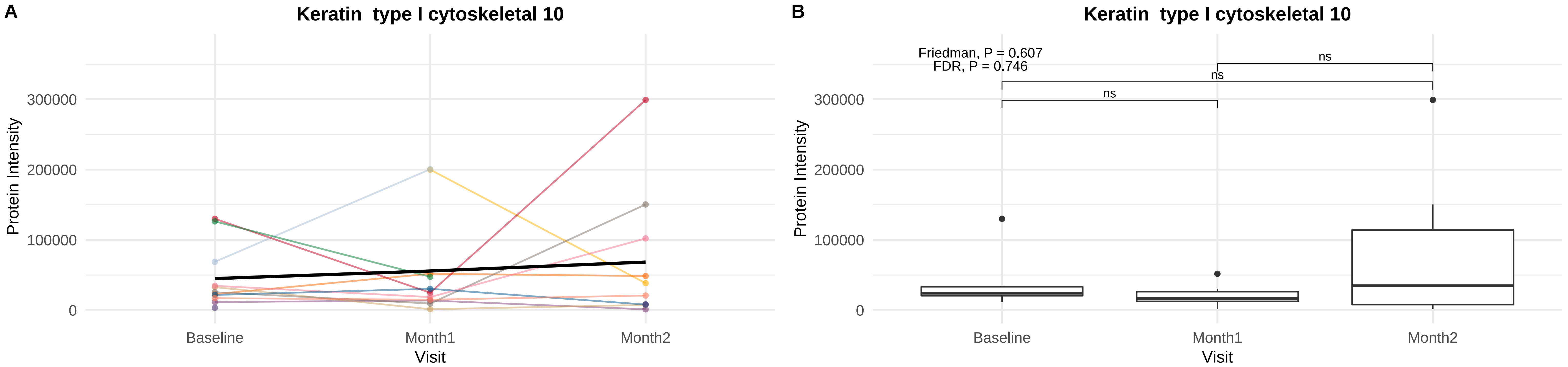

Supplementary Figure S 179

A) Line plot illustrating individual patient trajectories of Keratin type I cytoskeletal 10 intensity over time. The bold black line indicates the mean intensity over time. B) Box plots depicting the distribution of Keratin type I cytoskeletal 10 intensities at baseline, month 1, and month 2. Only AMD patients with measurements at all visits are included. The median, interquartile range, and outliers are displayed for each time point. Abbreviations: FDR, false discovery rate; ns, non−significant; * p < 0.05; ** p < 0.01; *** p < 0.001.

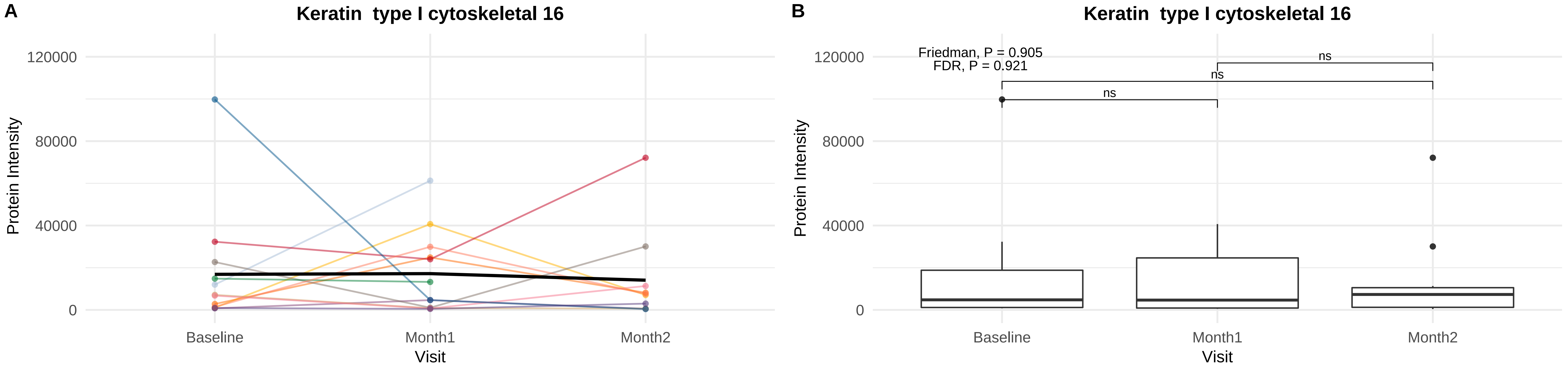

Supplementary Figure S 180

A) Line plot illustrating individual patient trajectories of Keratin type I cytoskeletal 16 intensity over time. The bold black line indicates the mean intensity over time. B) Box plots depicting the distribution of Keratin type I cytoskeletal 16 intensities at baseline, month 1, and month 2. Only AMD patients with measurements at all visits are included. The median, interquartile range, and outliers are displayed for each time point. Abbreviations: FDR, false discovery rate; ns, non−significant; * p < 0.05; ** p < 0.01; *** p < 0.001.

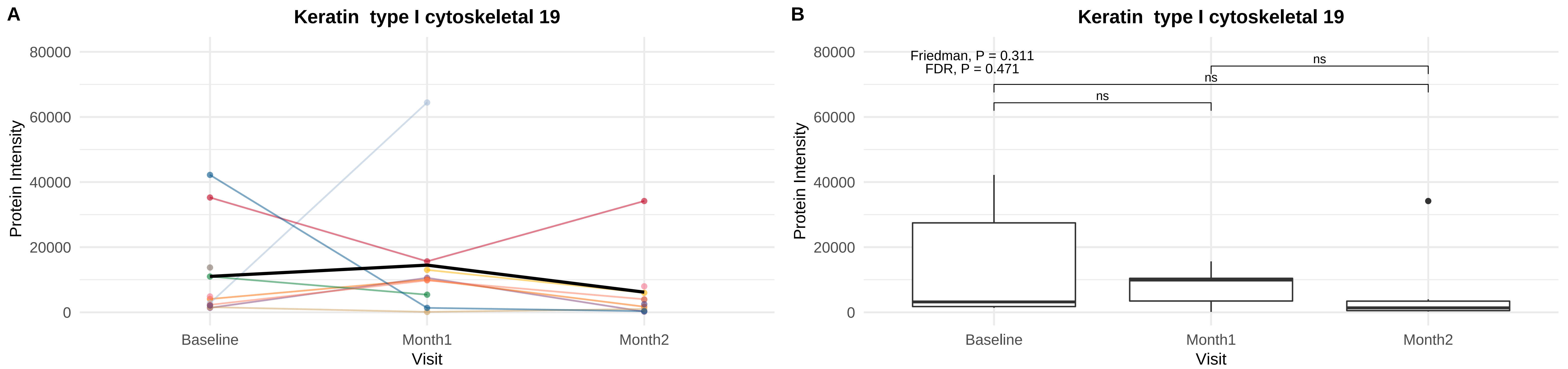

Supplementary Figure S 181

A) Line plot illustrating individual patient trajectories of Keratin type I cytoskeletal 19 intensity over time. The bold black line indicates the mean intensity over time. B) Box plots depicting the distribution of Keratin type I cytoskeletal 19 intensities at baseline, month 1, and month 2. Only AMD patients with measurements at all visits are included. The median, interquartile range, and outliers are displayed for each time point. Abbreviations: FDR, false discovery rate; ns, non−significant; * p < 0.05; ** p < 0.01; *** p < 0.001.

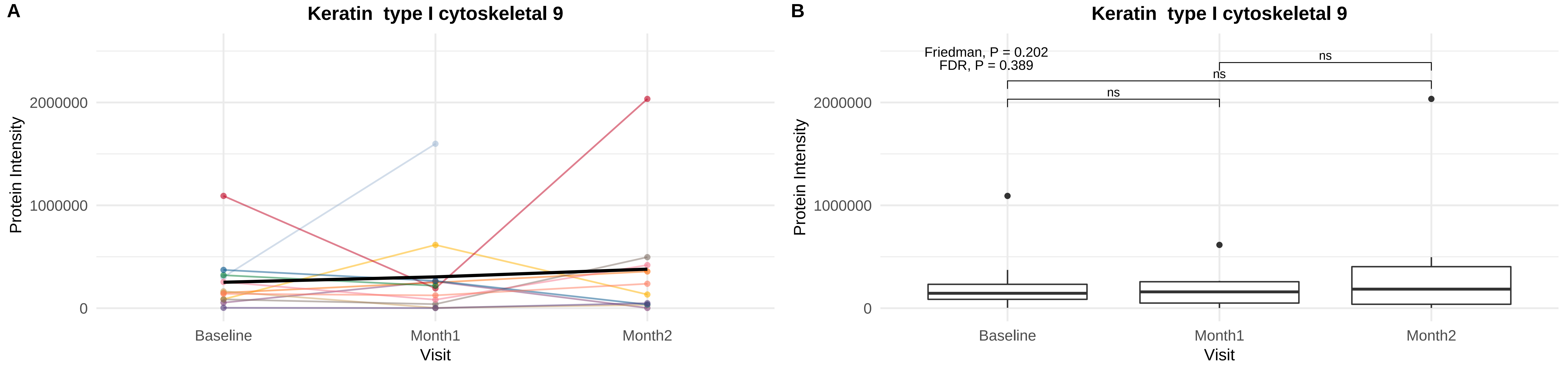

Supplementary Figure S 182

A) Line plot illustrating individual patient trajectories of Keratin type I cytoskeletal 9 intensity over time. The bold black line indicates the mean intensity over time. B) Box plots depicting the distribution of Keratin type I cytoskeletal 9 intensities at baseline, month 1, and month 2. Only AMD patients with measurements at all visits are included. The median, interquartile range, and outliers are displayed for each time point. Abbreviations: FDR, false discovery rate; ns, non−significant; * p < 0.05; ** p < 0.01; *** p < 0.001.

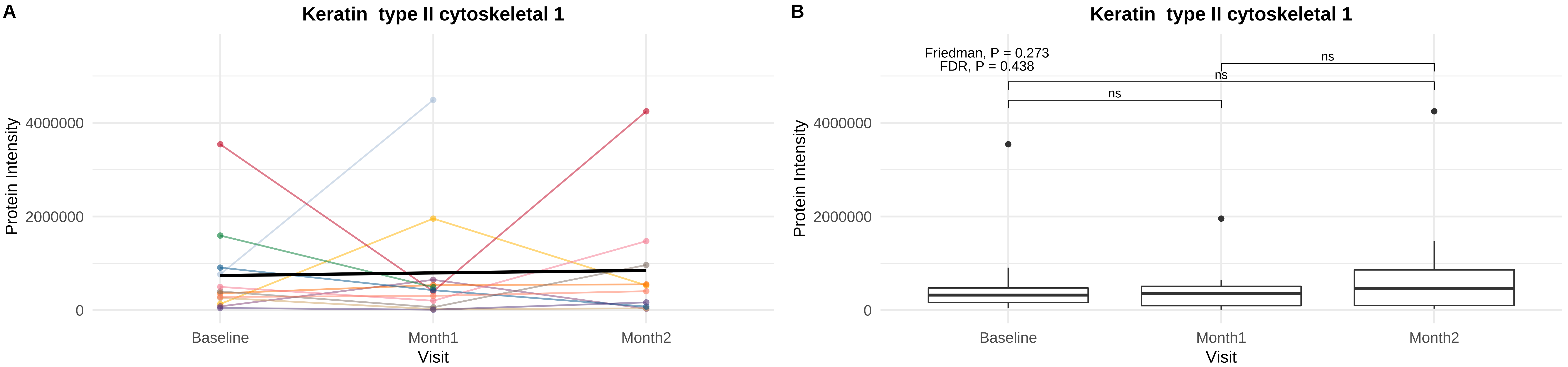

Supplementary Figure S 183

A) Line plot illustrating individual patient trajectories of Keratin type II cytoskeletal 1 intensity over time. The bold black line indicates the mean intensity over time. B) Box plots depicting the distribution of Keratin type II cytoskeletal 1 intensities at baseline, month 1, and month 2. Only AMD patients with measurements at all visits are included. The median, interquartile range, and outliers are displayed for each time point. Abbreviations: FDR, false discovery rate; ns, non−significant; * p < 0.05; ** p < 0.01; *** p < 0.001.

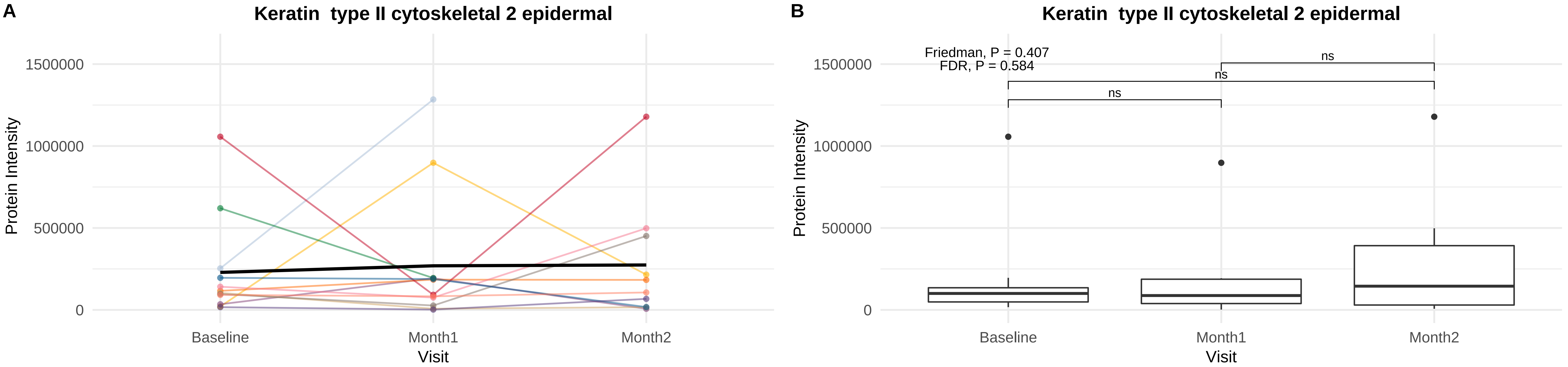

Supplementary Figure S 184

A) Line plot illustrating individual patient trajectories of Keratin type II cytoskeletal 2 epidermal intensity over time. The bold black line indicates the mean intensity over time. B) Box plots depicting the distribution of Keratin type II cytoskeletal 2 epidermal intensities at baseline, month 1, and month 2. Only AMD patients with measurements at all visits are included. The median, interquartile range, and outliers are displayed for each time point. Abbreviations: FDR, false discovery rate; ns, non−significant; * p < 0.05; ** p < 0.01; *** p < 0.001.

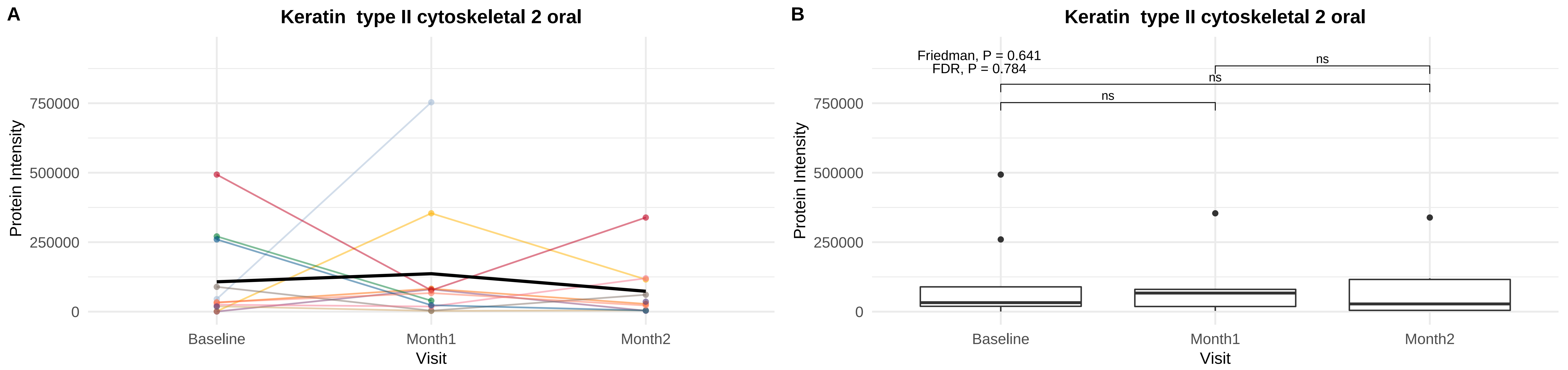

Supplementary Figure S 185

A) Line plot illustrating individual patient trajectories of Keratin type II cytoskeletal 2 oral intensity over time. The bold black line indicates the mean intensity over time. B) Box plots depicting the distribution of Keratin type II cytoskeletal 2 oral intensities at baseline, month 1, and month 2. Only AMD patients with measurements at all visits are included. The median, interquartile range, and outliers are displayed for each time point. Abbreviations: FDR, false discovery rate; ns, non−significant; * p < 0.05; ** p < 0.01; *** p < 0.001.

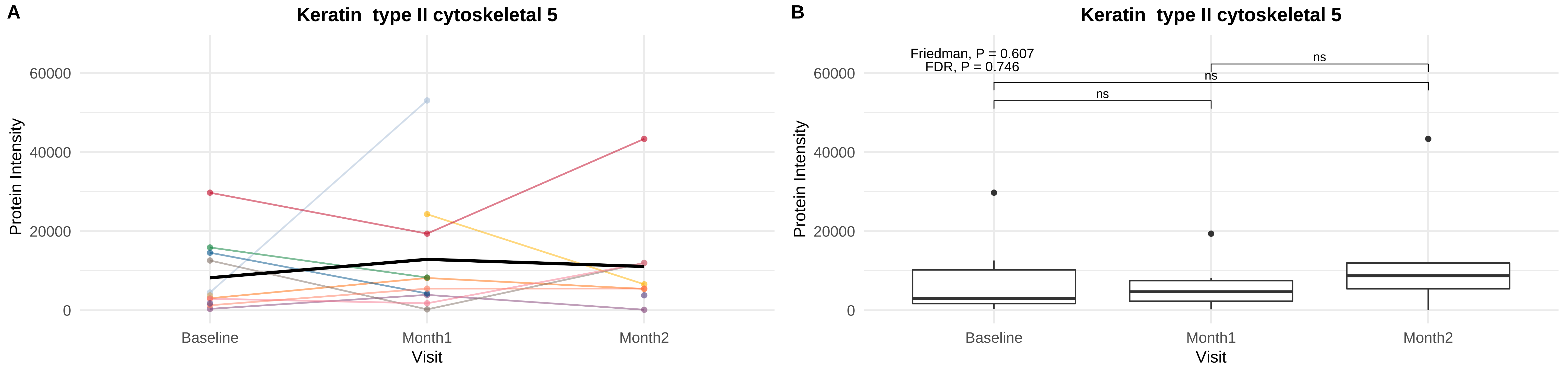

Supplementary Figure S 186

A) Line plot illustrating individual patient trajectories of Keratin type II cytoskeletal 5 intensity over time. The bold black line indicates the mean intensity over time. B) Box plots depicting the distribution of Keratin type II cytoskeletal 5 intensities at baseline, month 1, and month 2. Only AMD patients with measurements at all visits are included. The median, interquartile range, and outliers are displayed for each time point. Abbreviations: FDR, false discovery rate; ns, non−significant; * p < 0.05; ** p < 0.01; *** p < 0.001.

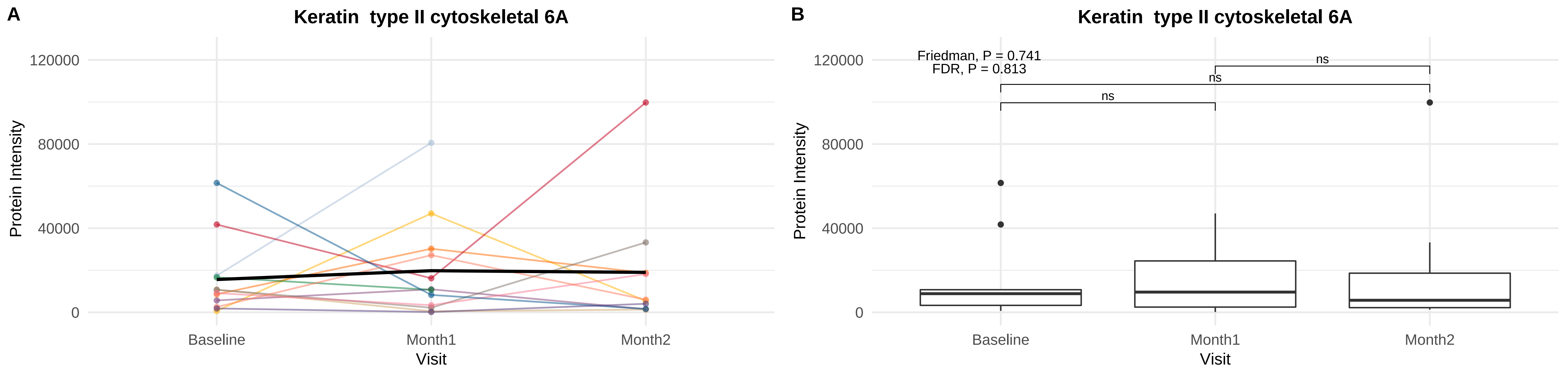

Supplementary Figure S 187

A) Line plot illustrating individual patient trajectories of Keratin type II cytoskeletal 6A intensity over time. The bold black line indicates the mean intensity over time. B) Box plots depicting the distribution of Keratin type II cytoskeletal 6A intensities at baseline, month 1, and month 2. Only AMD patients with measurements at all visits are included. The median, interquartile range, and outliers are displayed for each time point. Abbreviations: FDR, false discovery rate; ns, non−significant; * p < 0.05; ** p < 0.01; *** p < 0.001.

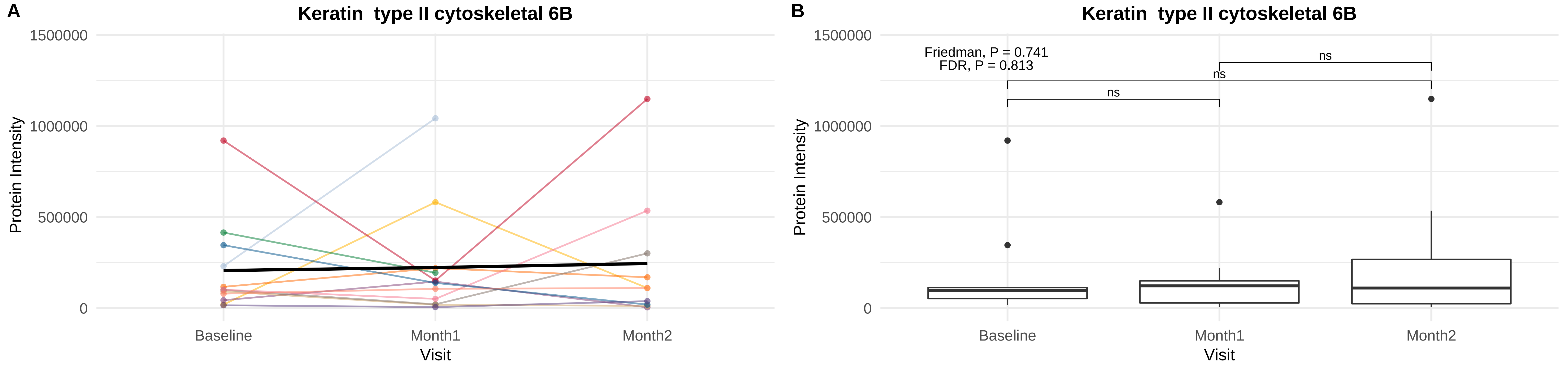

Supplementary Figure S 188

A) Line plot illustrating individual patient trajectories of Keratin type II cytoskeletal 6B intensity over time. The bold black line indicates the mean intensity over time. B) Box plots depicting the distribution of Keratin type II cytoskeletal 6B intensities at baseline, month 1, and month 2. Only AMD patients with measurements at all visits are included. The median, interquartile range, and outliers are displayed for each time point. Abbreviations: FDR, false discovery rate; ns, non−significant; * p < 0.05; ** p < 0.01; *** p < 0.001.

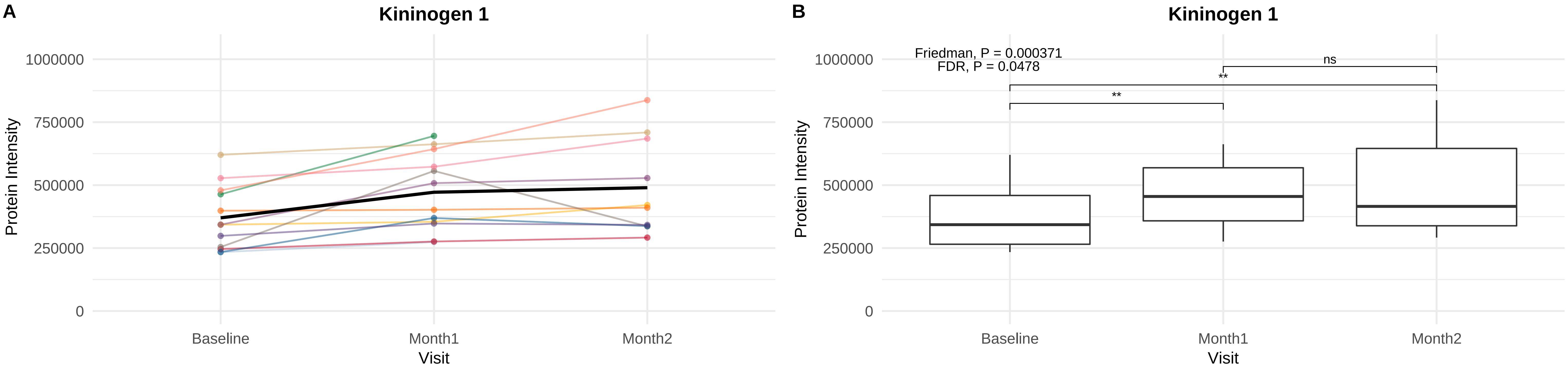

Supplementary Figure S 189

A) Line plot illustrating individual patient trajectories of Kininogen 1 intensity over time. The bold black line indicates the mean intensity over time. B) Box plots depicting the distribution of Kininogen 1 intensities at baseline, month 1, and month 2. Only AMD patients with measurements at all visits are included. The median, interquartile range, and outliers are displayed for each time point. Abbreviations: FDR, false discovery rate; ns, non−significant; * p < 0.05; ** p < 0.01; *** p < 0.001.

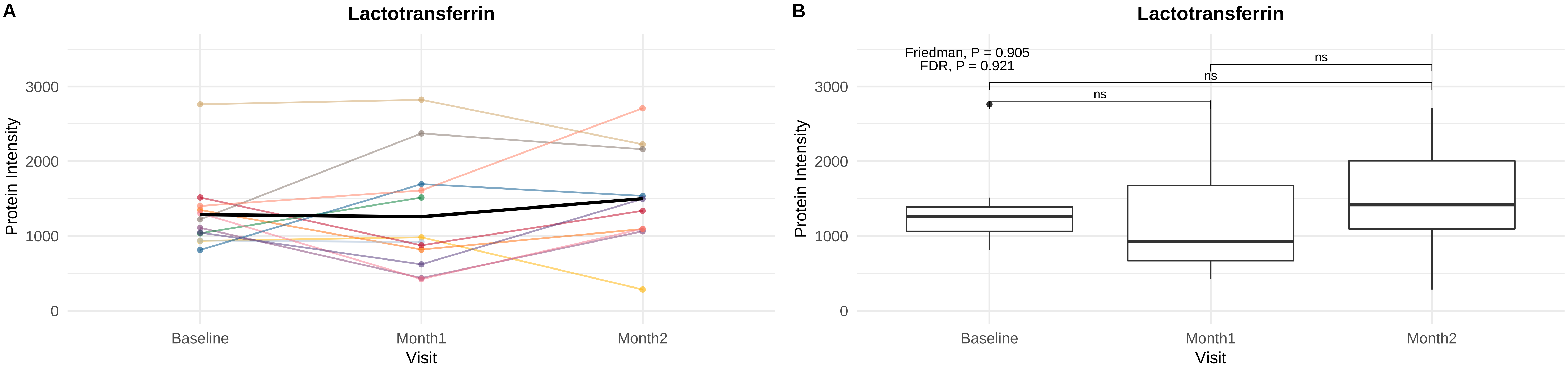

Supplementary Figure S 190

A) Line plot illustrating individual patient trajectories of Lactotransferrin intensity over time. The bold black line indicates the mean intensity over time. B) Box plots depicting the distribution of Lactotransferrin intensities at baseline, month 1, and month 2. Only AMD patients with measurements at all visits are included. The median, interquartile range, and outliers are displayed for each time point. Abbreviations: FDR, false discovery rate; ns, non−significant; * p < 0.05; ** p < 0.01; *** p < 0.001.

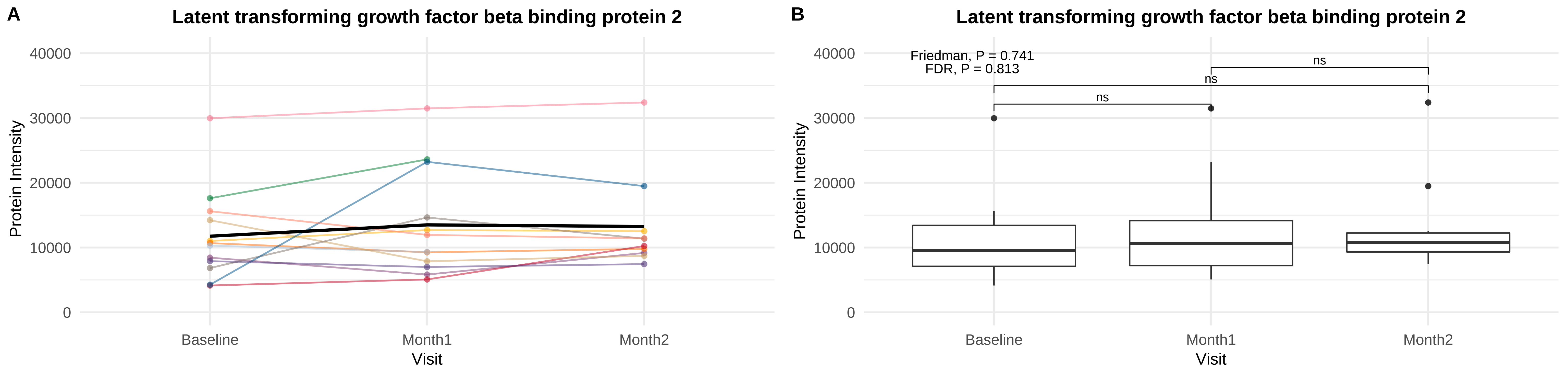

Supplementary Figure S 191

A) Line plot illustrating individual patient trajectories of Latent transforming growth factor beta binding protein 2 intensity over time. The bold black line indicates the mean intensity over time. B) Box plots depicting the distribution of Latent transforming growth factor beta binding protein 2 intensities at baseline, month 1, and month 2. Only AMD patients with measurements at all visits are included. The median, interquartile range, and outliers are displayed for each time point. Abbreviations: FDR, false discovery rate; ns, non−significant; * p < 0.05; ** p < 0.01; *** p < 0.001.

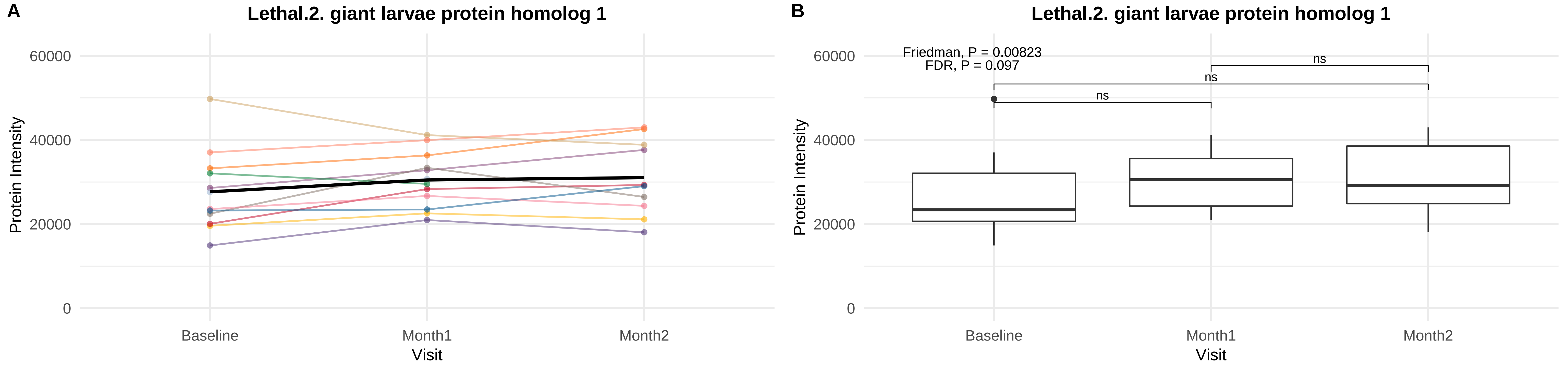

Supplementary Figure S 192

A) Line plot illustrating individual patient trajectories of Lethal.2. giant larvae protein homolog 1 intensity over time. The bold black line indicates the mean intensity over time. B) Box plots depicting the distribution of Lethal.2. giant larvae protein homolog 1 intensities at baseline, month 1, and month 2. Only AMD patients with measurements at all visits are included. The median, interquartile range, and outliers are displayed for each time point. Abbreviations: FDR, false discovery rate; ns, non−significant; * p < 0.05; ** p < 0.01; *** p < 0.001.

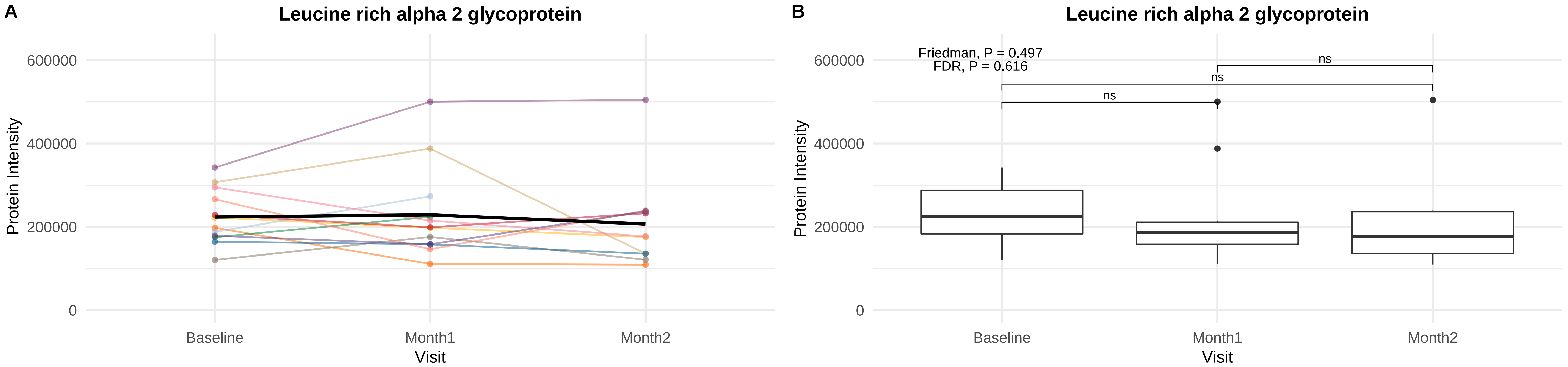

Supplementary Figure S 193

A) Line plot illustrating individual patient trajectories of Leucine rich alpha 2 glycoprotein intensity over time. The bold black line indicates the mean intensity over time. B) Box plots depicting the distribution of Leucine rich alpha 2 glycoprotein intensities at baseline, month 1, and month 2. Only AMD patients with measurements at all visits are included. The median, interquartile range, and outliers are displayed for each time point. Abbreviations: FDR, false discovery rate; ns, non−significant; * $p < 0.05$; ** $p < 0.01$; *** $p < 0.001$.

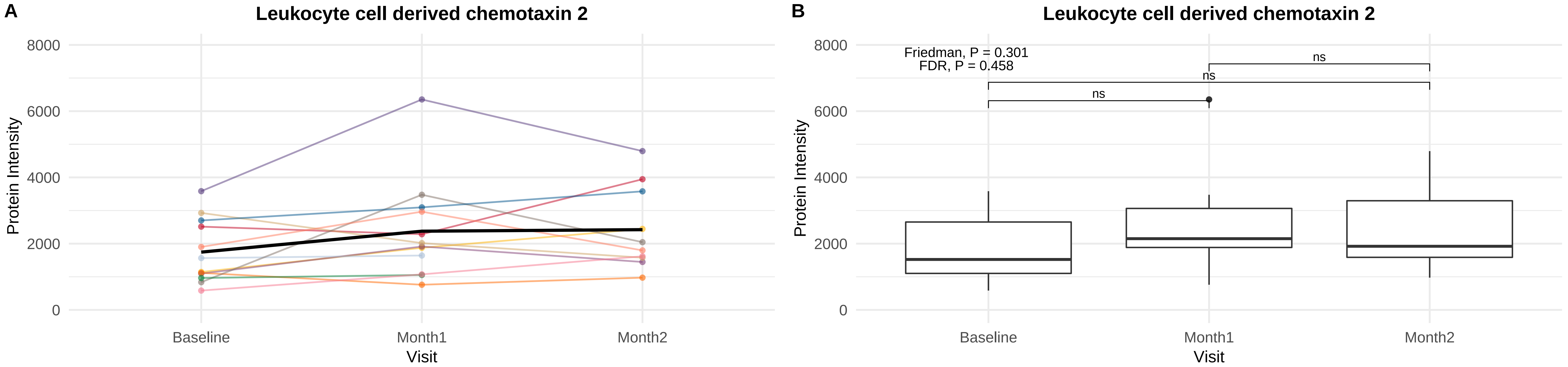

Supplementary Figure S 194

A) Line plot illustrating individual patient trajectories of Leukocyte cell derived chemotaxin 2 intensity over time. The bold black line indicates the mean intensity over time. B) Box plots depicting the distribution of Leukocyte cell derived chemotaxin 2 intensities at baseline, month 1, and month 2. Only AMD patients with measurements at all visits are included. The median, interquartile range, and outliers are displayed for each time point. Abbreviations: FDR, false discovery rate; ns, non−significant; * $p < 0.05$; ** $p < 0.01$; *** $p < 0.001$.

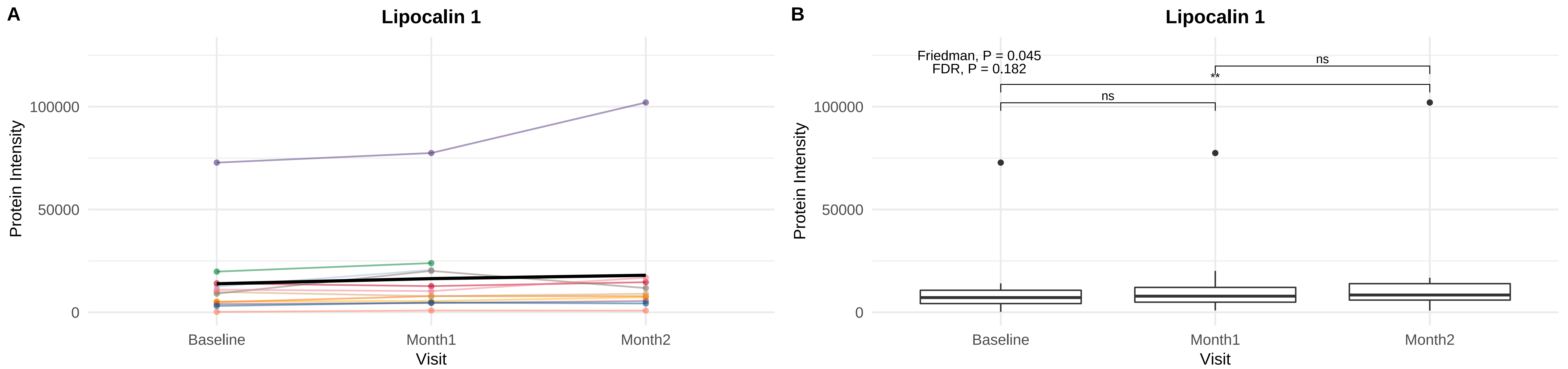

Supplementary Figure S 195

A) Line plot illustrating individual patient trajectories of Lipocalin 1 intensity over time. The bold black line indicates the mean intensity over time. B) Box plots depicting the distribution of Lipocalin 1 intensities at baseline, month 1, and month 2. Only AMD patients with measurements at all visits are included. The median, interquartile range, and outliers are displayed for each time point. Abbreviations: FDR, false discovery rate; ns, non−significant; * p < 0.05; ** p < 0.01; *** p < 0.001.

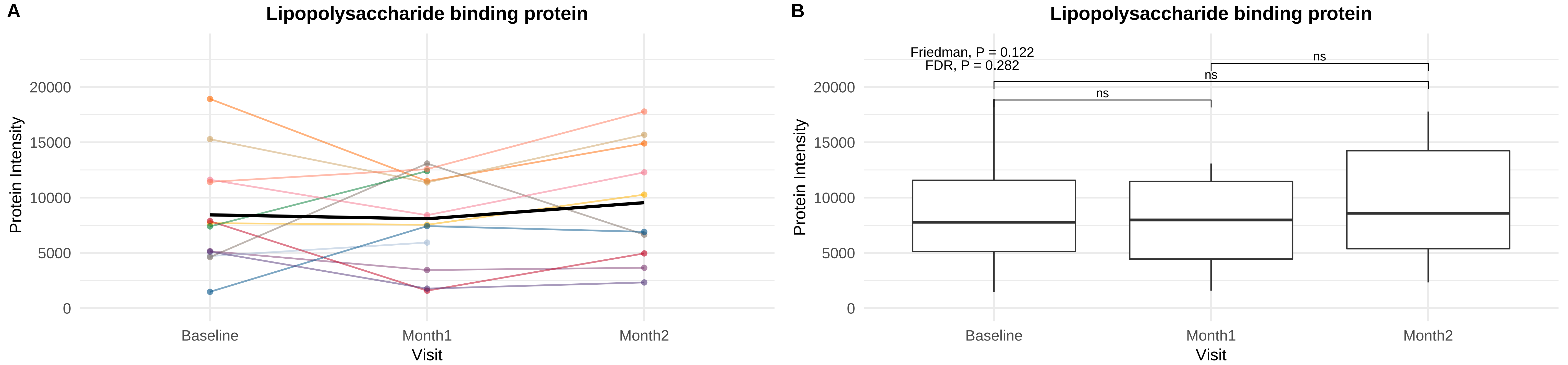

Supplementary Figure S 196

A) Line plot illustrating individual patient trajectories of Lipopolysaccharide binding protein intensity over time. The bold black line indicates the mean intensity over time. B) Box plots depicting the distribution of Lipopolysaccharide binding protein intensities at baseline, month 1, and month 2. Only AMD patients with measurements at all visits are included. The median, interquartile range, and outliers are displayed for each time point. Abbreviations: FDR, false discovery rate; ns, non−significant; * p < 0.05; ** p < 0.01; *** p < 0.001.

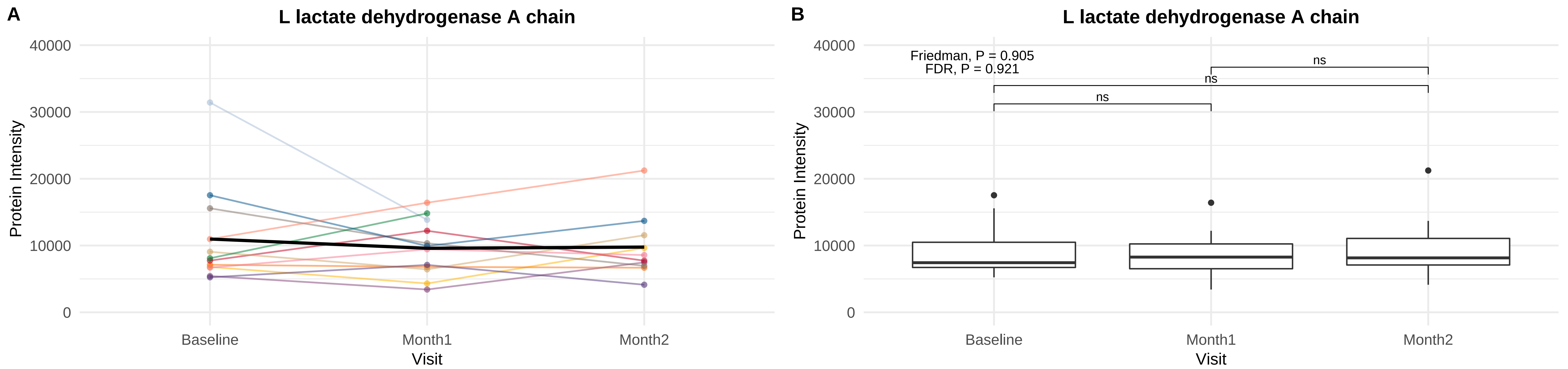

Supplementary Figure S 197

A) Line plot illustrating individual patient trajectories of L lactate dehydrogenase A chain intensity over time. The bold black line indicates the mean intensity over time. B) Box plots depicting the distribution of L lactate dehydrogenase A chain intensities at baseline, month 1, and month 2. Only AMD patients with measurements at all visits are included. The median, interquartile range, and outliers are displayed for each time point. Abbreviations: FDR, false discovery rate; ns, non−significant; * p < 0.05; ** p < 0.01; *** p < 0.001.

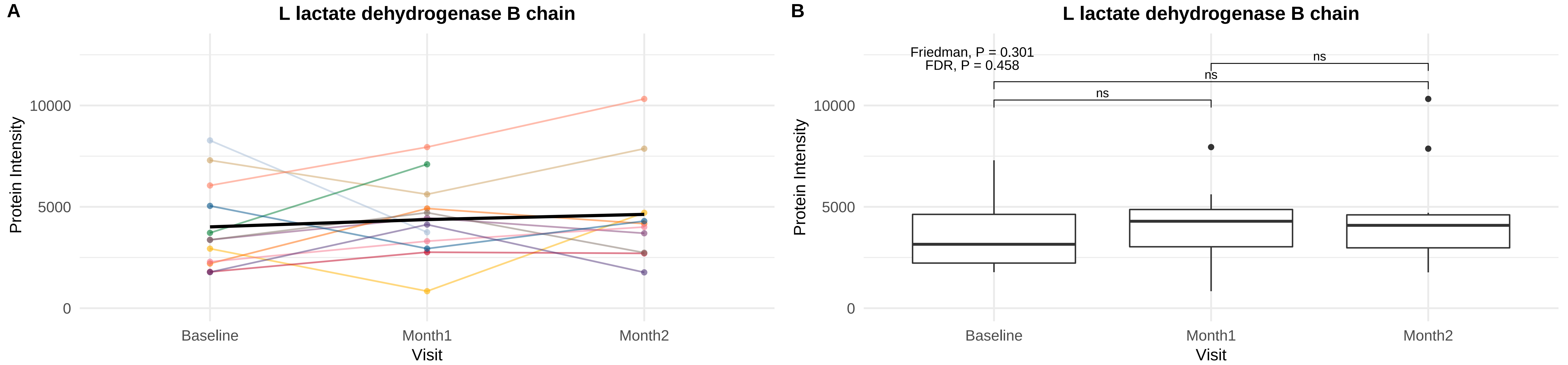

Supplementary Figure S 198

A) Line plot illustrating individual patient trajectories of L lactate dehydrogenase B chain intensity over time. The bold black line indicates the mean intensity over time. B) Box plots depicting the distribution of L lactate dehydrogenase B chain intensities at baseline, month 1, and month 2. Only AMD patients with measurements at all visits are included. The median, interquartile range, and outliers are displayed for each time point. Abbreviations: FDR, false discovery rate; ns, non−significant; * p < 0.05; ** p < 0.01; *** p < 0.001.

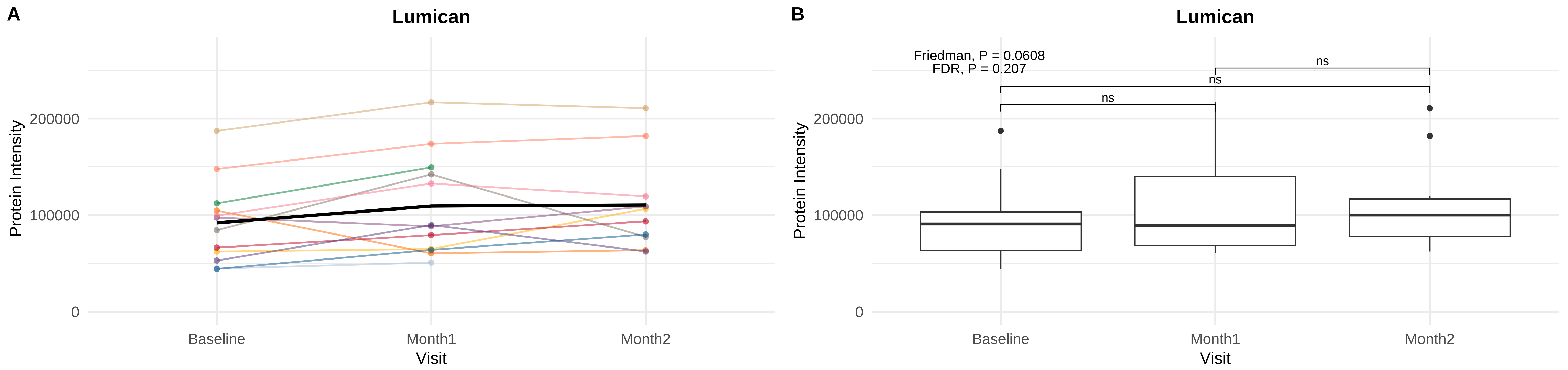

Supplementary Figure S 199

A) Line plot illustrating individual patient trajectories of Lumican intensity over time. The bold black line indicates the mean intensity over time. B) Box plots depicting the distribution of Lumican intensities at baseline, month 1, and month 2. Only AMD patients with measurements at all visits are included. The median, interquartile range, and outliers are displayed for each time point. Abbreviations: FDR, false discovery rate; ns, non−significant; * p < 0.05; ** p < 0.01; *** p < 0.001.

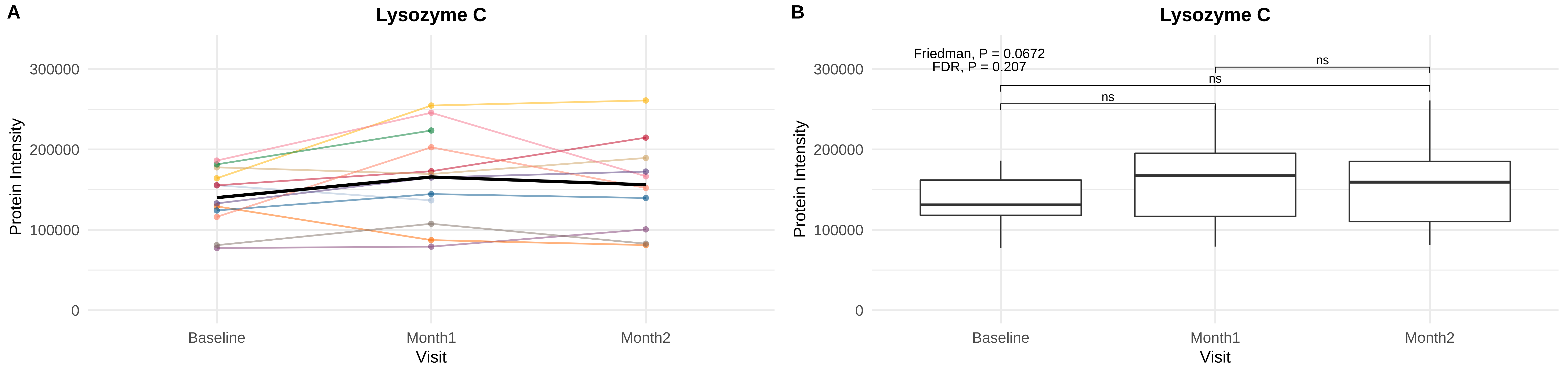

Supplementary Figure S 200

A) Line plot illustrating individual patient trajectories of Lysozyme C intensity over time. The bold black line indicates the mean intensity over time. B) Box plots depicting the distribution of Lysozyme C intensities at baseline, month 1, and month 2. Only AMD patients with measurements at all visits are included. The median, interquartile range, and outliers are displayed for each time point. Abbreviations: FDR, false discovery rate; ns, non−significant; * p < 0.05; ** p < 0.01; *** p < 0.001.

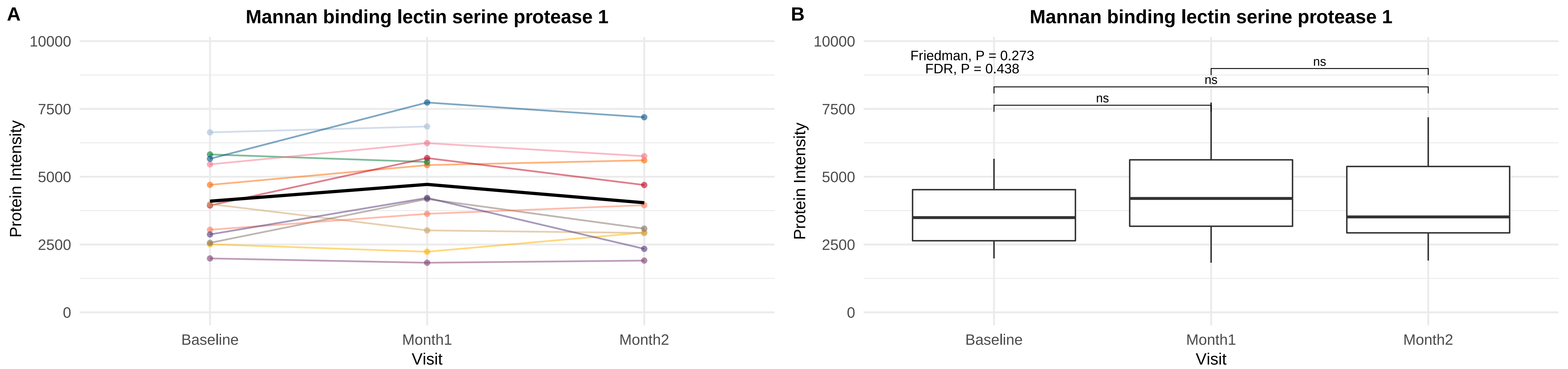

Supplementary Figure S 201

A) Line plot illustrating individual patient trajectories of Mannan binding lectin serine protease 1 intensity over time. The bold black line indicates the mean intensity over time. B) Box plots depicting the distribution of Mannan binding lectin serine protease 1 intensities at baseline, month 1, and month 2. Only AMD patients with measurements at all visits are included. The median, interquartile range, and outliers are displayed for each time point. Abbreviations: FDR, false discovery rate; ns, non−significant; * p < 0.05; ** p < 0.01; *** p < 0.001.

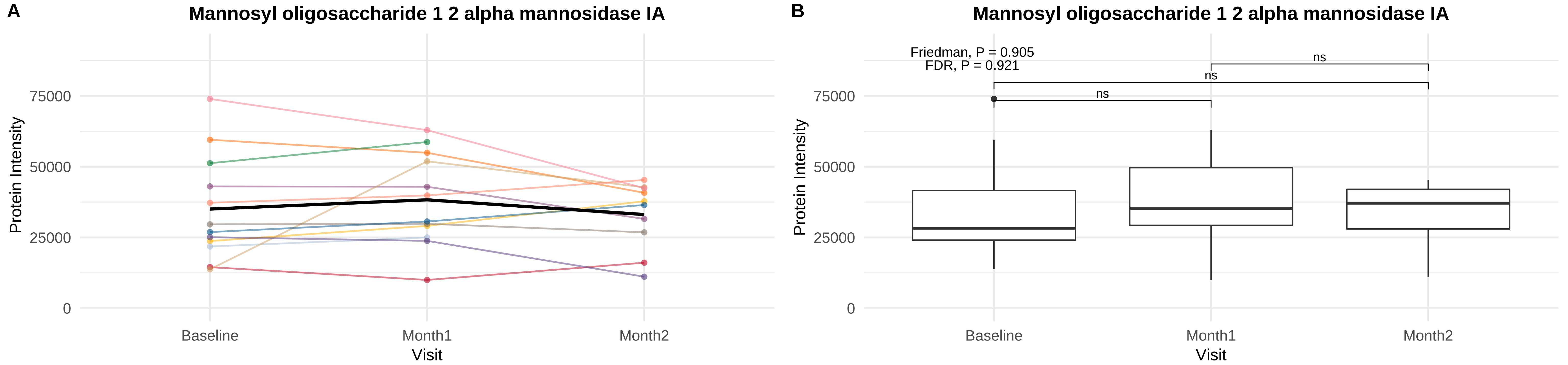

Supplementary Figure S 202

A) Line plot illustrating individual patient trajectories of Mannosyl oligosaccharide 1 2 alpha mannosidase IA intensity over time. The bold black line indicates the mean intensity over time. B) Box plots depicting the distribution of Mannosyl oligosaccharide 1 2 alpha mannosidase IA intensities at baseline, month 1, and month 2. Only AMD patients with measurements at all visits are included. The median, interquartile range, and outliers are displayed for each time point. Abbreviations: FDR, false discovery rate; ns, non−significant; * p < 0.05; ** p < 0.01; *** p < 0.001

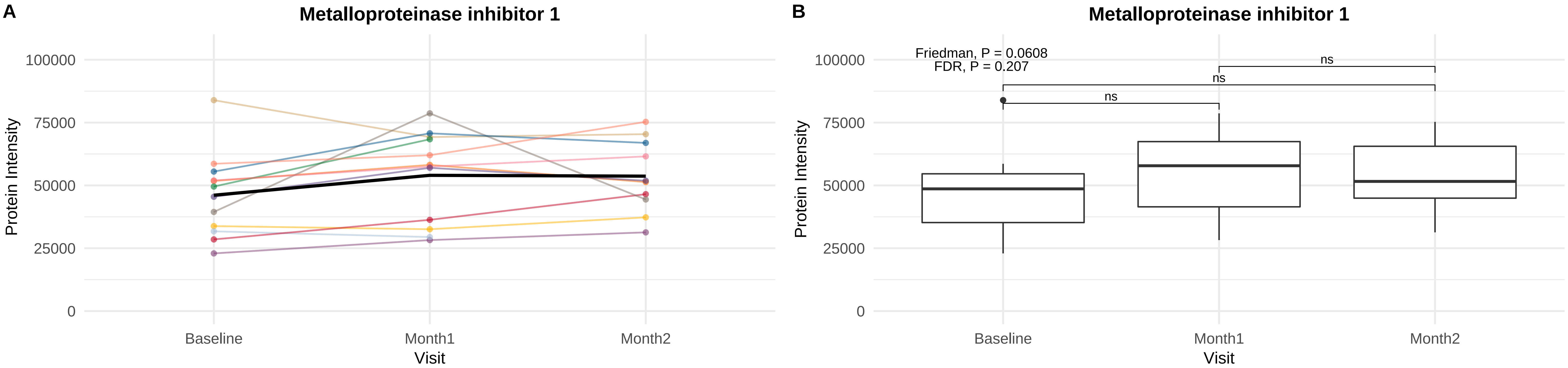

Supplementary Figure S 203

A) Line plot illustrating individual patient trajectories of Metalloproteinase inhibitor 1 intensity over time. The bold black line indicates the mean intensity over time. B) Box plots depicting the distribution of Metalloproteinase inhibitor 1 intensities at baseline, month 1, and month 2. Only AMD patients with measurements at all visits are included. The median, interquartile range, and outliers are displayed for each time point. Abbreviations: FDR, false discovery rate; ns, non−significant; * p < 0.05; ** p < 0.01; *** p < 0.001.

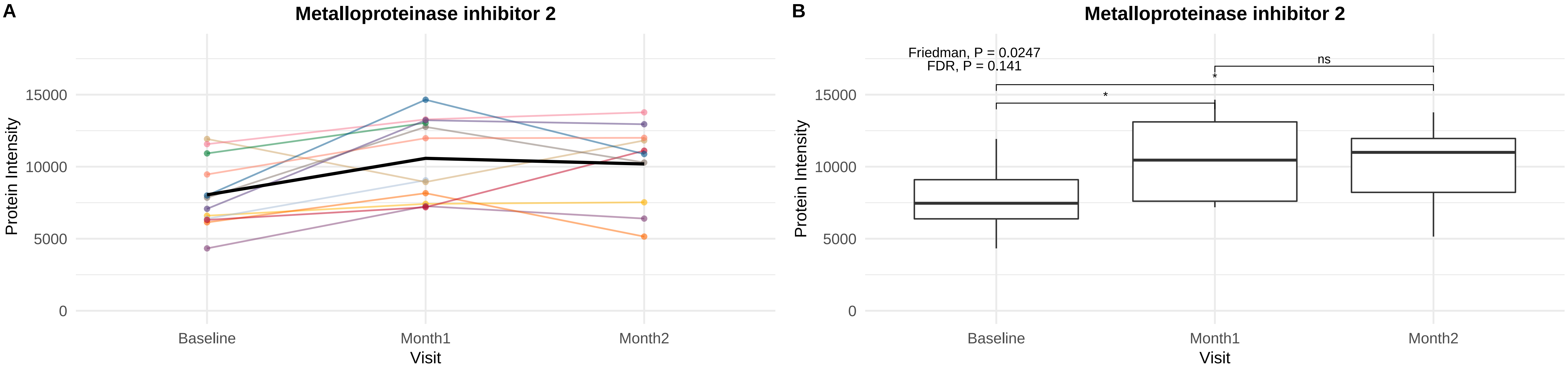

Supplementary Figure S 204

A) Line plot illustrating individual patient trajectories of Metalloproteinase inhibitor 2 intensity over time. The bold black line indicates the mean intensity over time. B) Box plots depicting the distribution of Metalloproteinase inhibitor 2 intensities at baseline, month 1, and month 2. Only AMD patients with measurements at all visits are included. The median, interquartile range, and outliers are displayed for each time point. Abbreviations: FDR, false discovery rate; ns, non−significant; * p < 0.05; ** p < 0.01; *** p < 0.001.

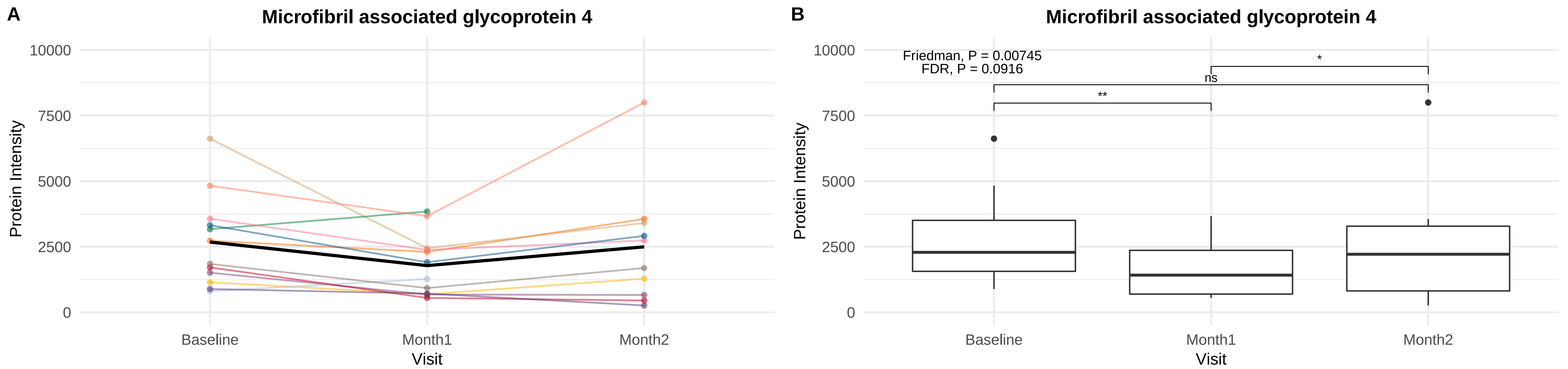

Supplementary Figure S 205

A) Line plot illustrating individual patient trajectories of Microfibril associated glycoprotein 4 intensity over time. The bold black line indicates the mean intensity over time. B) Box plots depicting the distribution of Microfibril associated glycoprotein 4 intensities at baseline, month 1, and month 2. Only AMD patients with measurements at all visits are included. The median, interquartile range, and outliers are displayed for each time point. Abbreviations: FDR, false discovery rate; ns, non−significant; * p < 0.05; ** p < 0.01; *** p < 0.001.

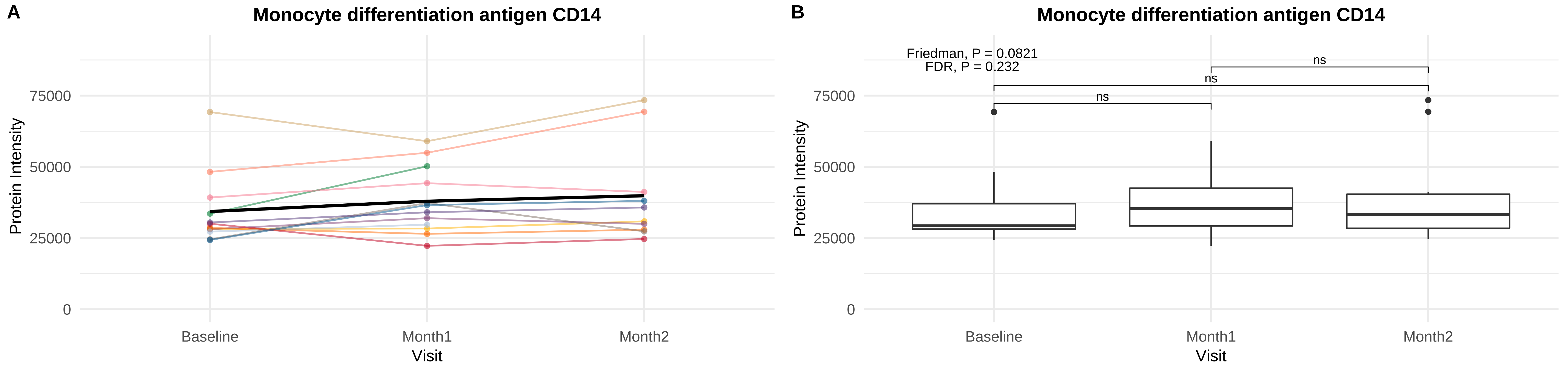

Supplementary Figure S 206

A) Line plot illustrating individual patient trajectories of Monocyte differentiation antigen CD14 intensity over time. The bold black line indicates the mean intensity over time. B) Box plots depicting the distribution of Monocyte differentiation antigen CD14 intensities at baseline, month 1, and month 2. Only AMD patients with measurements at all visits are included. The median, interquartile range, and outliers are displayed for each time point. Abbreviations: FDR, false discovery rate; ns, non−significant; * p < 0.05; ** p < 0.01; *** p < 0.001.

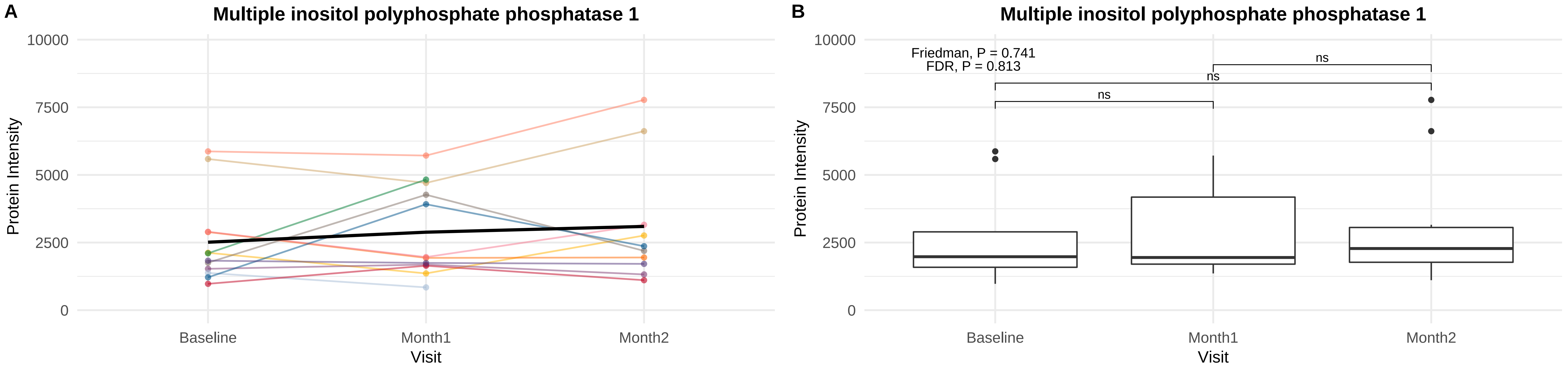

Supplementary Figure S 207

A) Line plot illustrating individual patient trajectories of Multiple inositol polyphosphate phosphatase 1 intensity over time. The bold black line indicates the mean intensity over time. B) Box plots depicting the distribution of Multiple inositol polyphosphate phosphatase 1 intensities at baseline, month 1, and month 2. Only AMD patients with measurements at all visits are included. The median, interquartile range, and outliers are displayed for each time point. Abbreviations: FDR, false discovery rate; ns, non−significant; * p < 0.05; ** p < 0.01; *** p < 0.001.

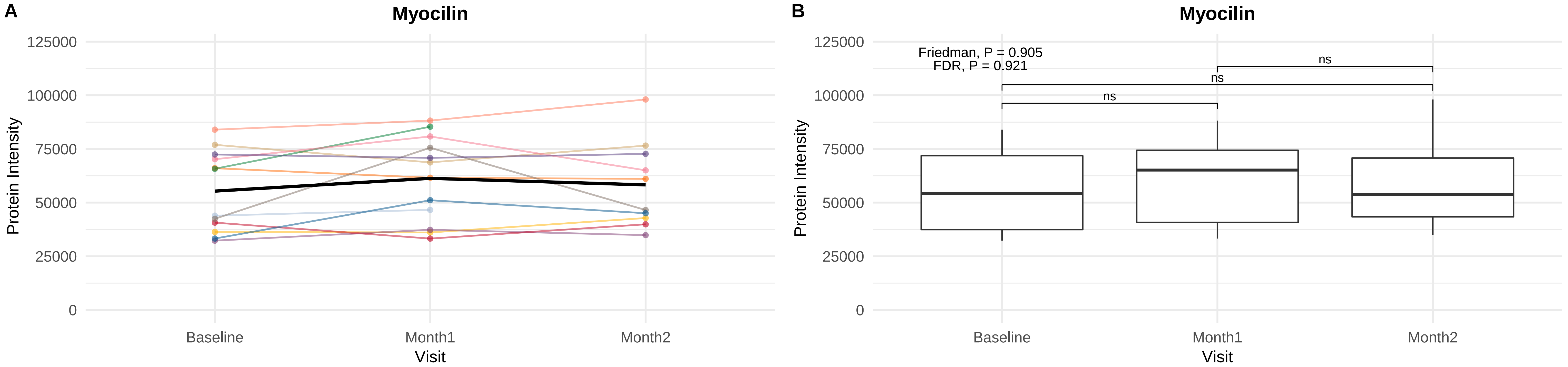

Supplementary Figure S 208

A) Line plot illustrating individual patient trajectories of Myocilin intensity over time. The bold black line indicates the mean intensity over time. B) Box plots depicting the distribution of Myocilin intensities at baseline, month 1, and month 2. Only AMD patients with measurements at all visits are included. The median, interquartile range, and outliers are displayed for each time point. Abbreviations: FDR, false discovery rate; ns, non−significant; * p < 0.05; ** p < 0.01; *** p < 0.001.

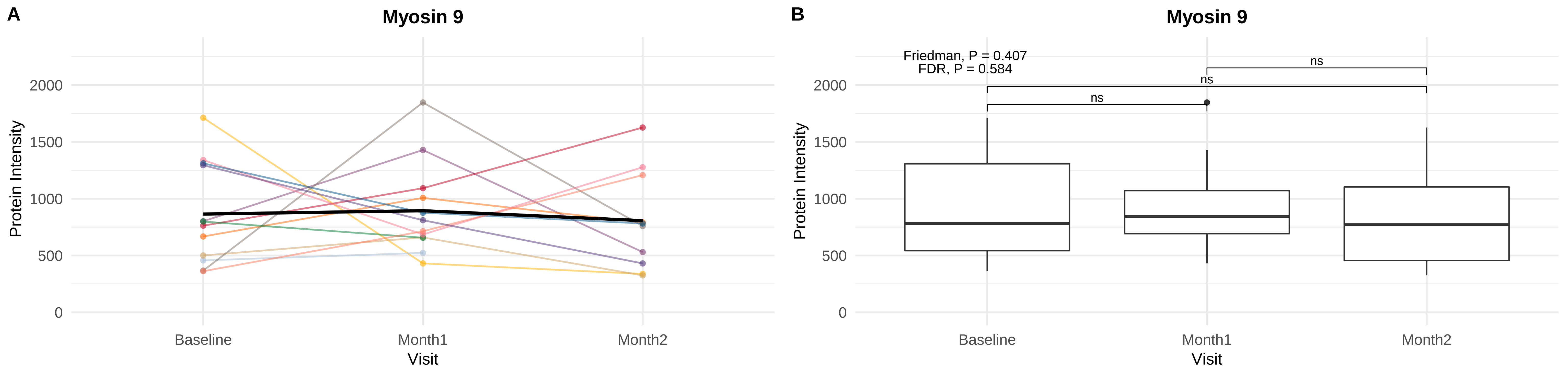

Supplementary Figure S 209

A) Line plot illustrating individual patient trajectories of Myosin 9 intensity over time. The bold black line indicates the mean intensity over time. B) Box plots depicting the distribution of Myosin 9 intensities at baseline, month 1, and month 2. Only AMD patients with measurements at all visits are included. The median, interquartile range, and outliers are displayed for each time point. Abbreviations: FDR, false discovery rate; ns, non−significant; * p < 0.05; ** p < 0.01; *** p < 0.001.

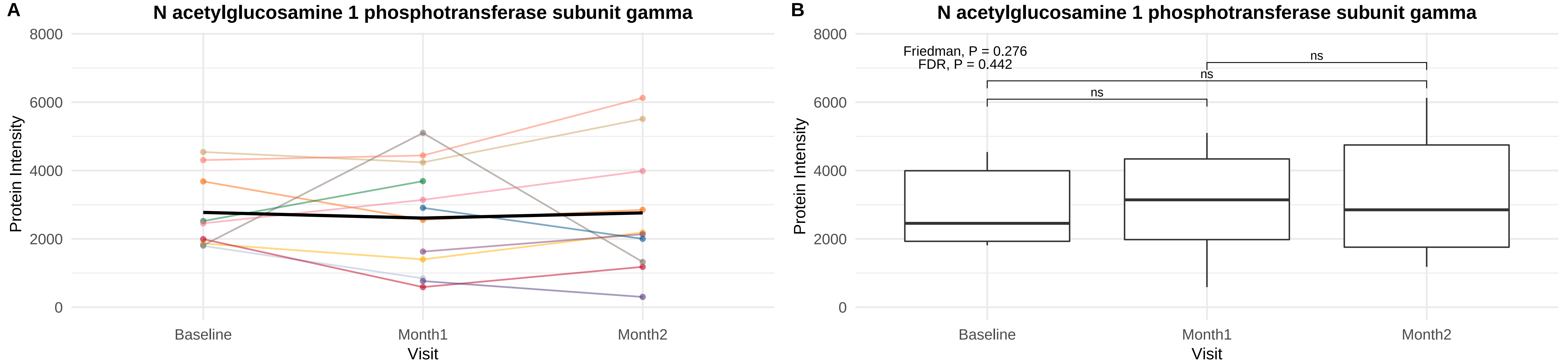

Supplementary Figure S 210

A) Line plot illustrating individual patient trajectories of N acetylglucosamine 1 phosphotransferase subunit gamma intensity over time. The bold black line indicates the mean intensity over time. B) Box plots depicting the distribution of N acetylglucosamine 1 phosphotransferase subunit ga intensities at baseline, month 1, and month 2. Only AMD patients with measurements at all visits are included. The median, interquartile range, and outliers are displayed for each time point. Abbreviations: FDR, false discovery rate; ns, non−significant; * p < 0.05; ** p < 0.01; *** p < 0.001

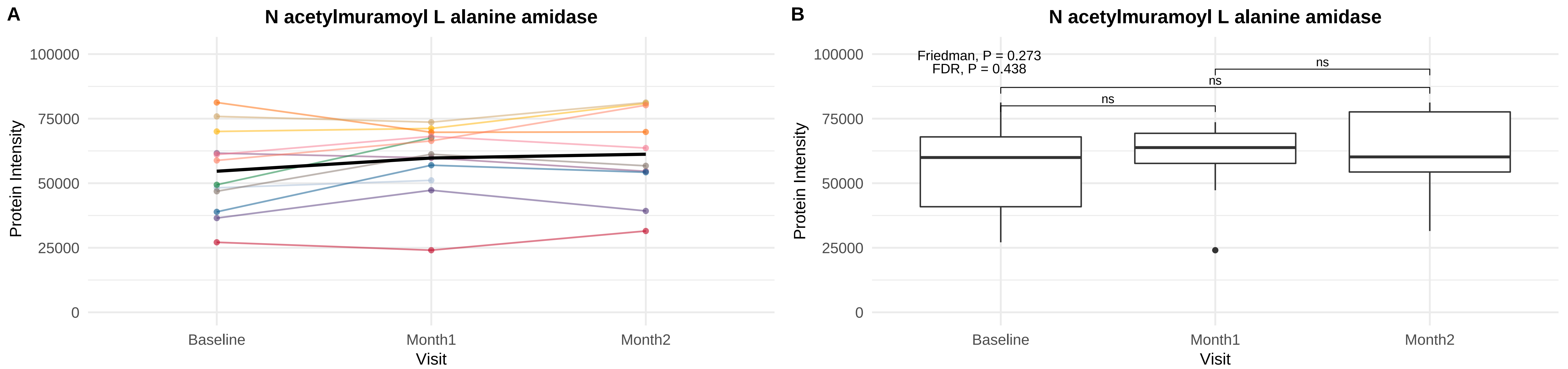

Supplementary Figure S 211

A) Line plot illustrating individual patient trajectories of N acetylmuramoyl L alanine amidase intensity over time. The bold black line indicates the mean intensity over time. B) Box plots depicting the distribution of N acetylmuramoyl L alanine amidase intensities at baseline, month 1, and month 2. Only AMD patients with measurements at all visits are included. The median, interquartile range, and outliers are displayed for each time point. Abbreviations: FDR, false discovery rate; ns, non−significant; * p < 0.05; ** p < 0.01; *** p < 0.001.

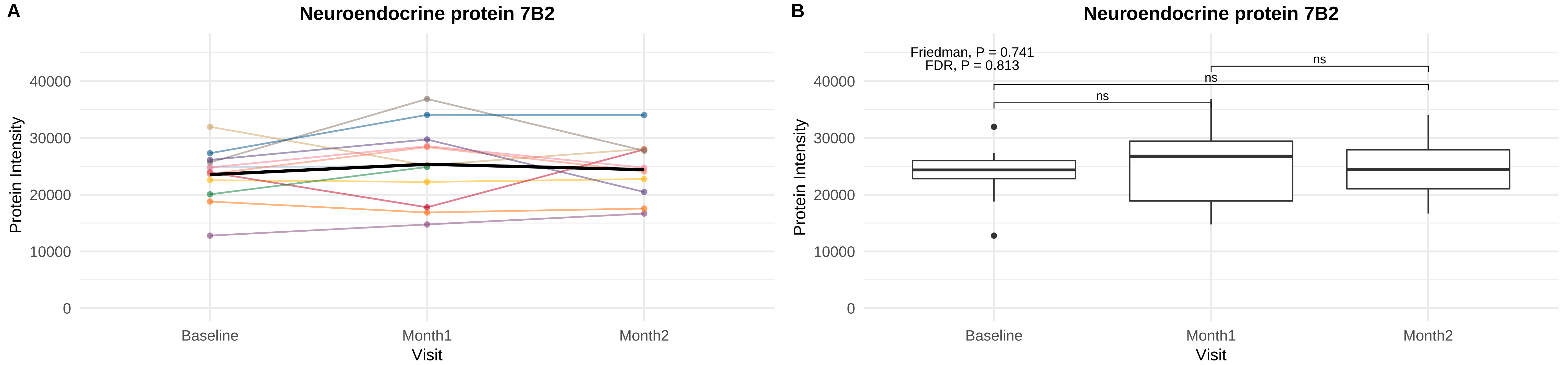

Supplementary Figure S 212

A) Line plot illustrating individual patient trajectories of Neuroendocrine protein 7B2 intensity over time. The bold black line indicates the mean intensity over time. B) Box plots depicting the distribution of Neuroendocrine protein 7B2 intensities at baseline, month 1, and month 2. Only AMD patients with measurements at all visits are included. The median, interquartile range, and outliers are displayed for each time point. Abbreviations: FDR, false discovery rate; ns, non−significant; * p < 0.05; ** p < 0.01; *** p < 0.001.

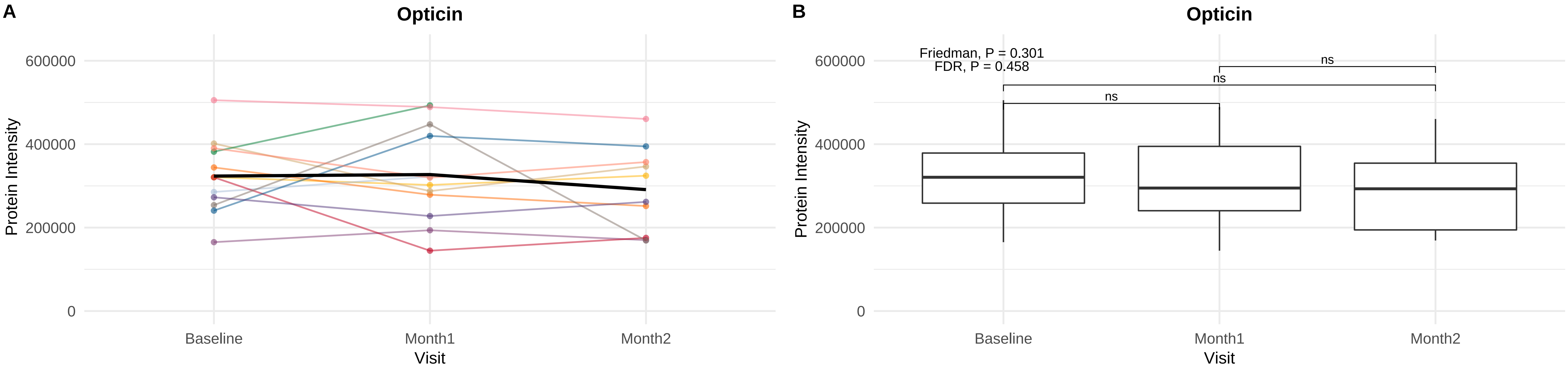

Supplementary Figure S 213

A) Line plot illustrating individual patient trajectories of Opticin intensity over time. The bold black line indicates the mean intensity over time. B) Box plots depicting the distribution of Opticin intensities at baseline, month 1, and month 2. Only AMD patients with measurements at all visits are included. The median, interquartile range, and outliers are displayed for each time point. Abbreviations: FDR, false discovery rate; ns, non−significant; * p < 0.05; ** p < 0.01; *** p < 0.001.

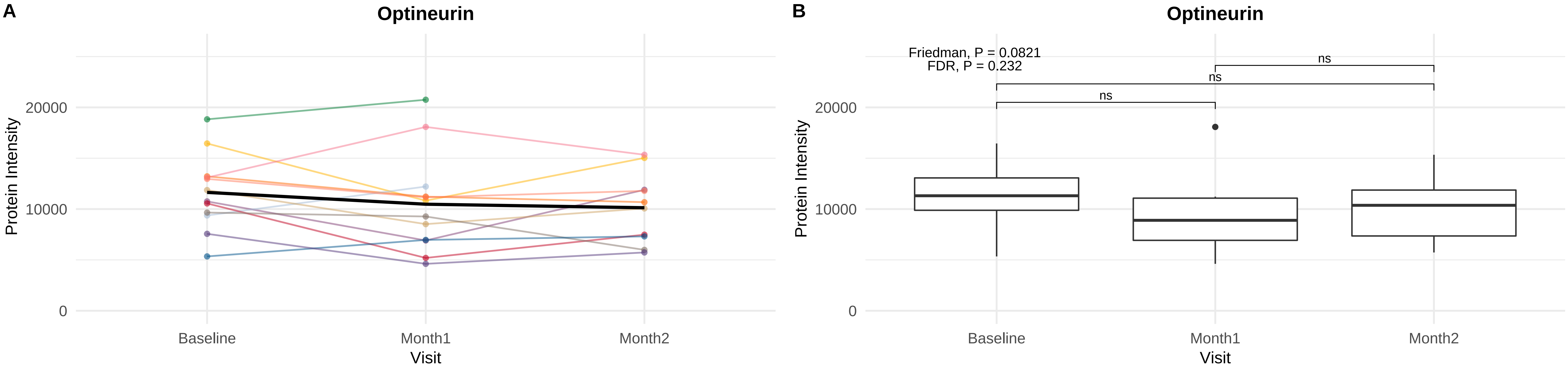

Supplementary Figure S 214

A) Line plot illustrating individual patient trajectories of Optineurin intensity over time. The bold black line indicates the mean intensity over time. B) Box plots depicting the distribution of Optineurin intensities at baseline, month 1, and month 2. Only AMD patients with measurements at all visits are included. The median, interquartile range, and outliers are displayed for each time point. Abbreviations: FDR, false discovery rate; ns, non−significant; * p < 0.05; ** p < 0.01; *** p < 0.001.

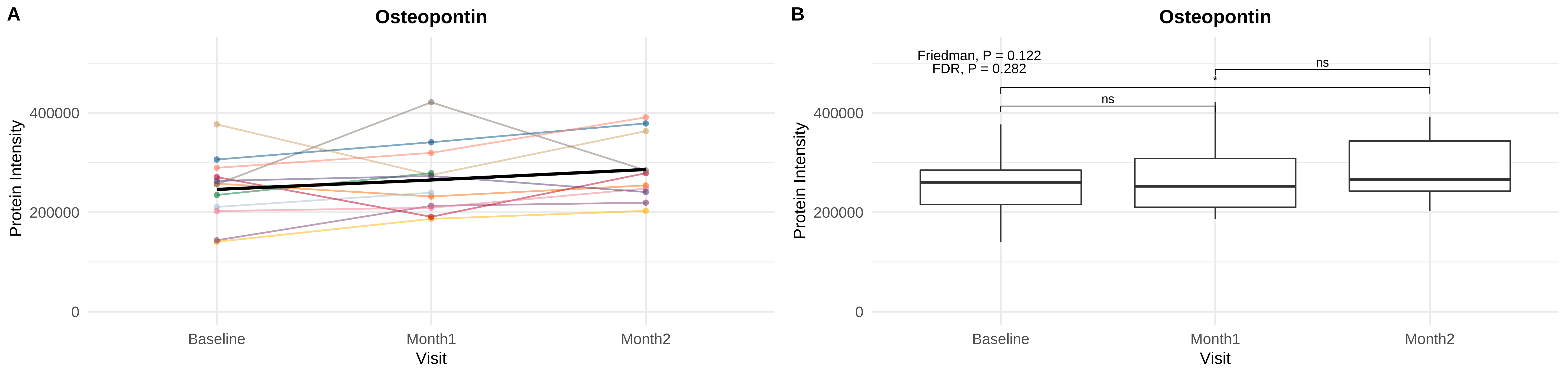

Supplementary Figure S 215

A) Line plot illustrating individual patient trajectories of Osteopontin intensity over time. The bold black line indicates the mean intensity over time. B) Box plots depicting the distribution of Osteopontin intensities at baseline, month 1, and month 2. Only AMD patients with measurements at all visits are included. The median, interquartile range, and outliers are displayed for each time point. Abbreviations: FDR, false discovery rate; ns, non−significant; * p < 0.05; ** p < 0.01; *** p < 0.001.

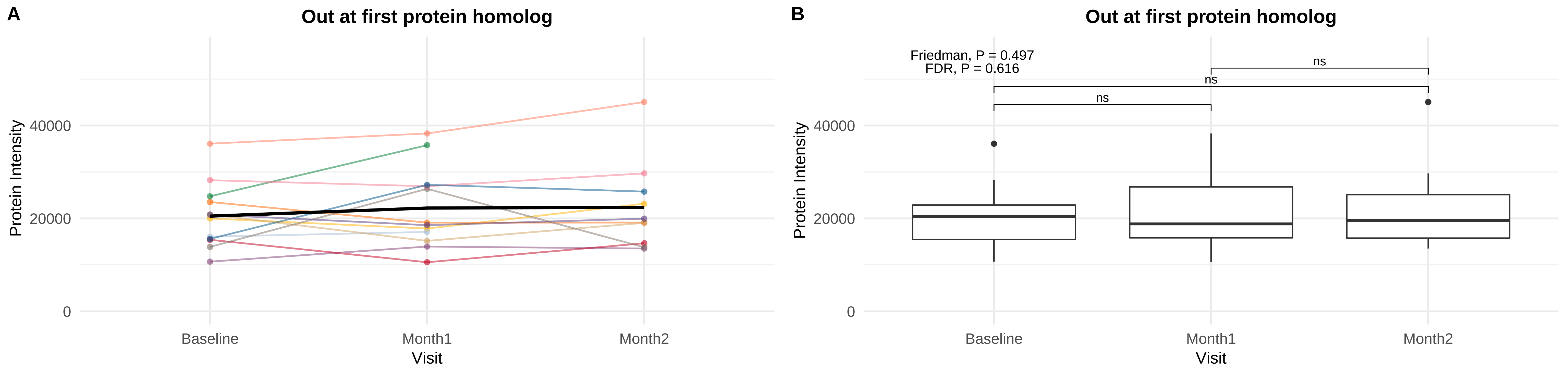

Supplementary Figure S 216

A) Line plot illustrating individual patient trajectories of Out at first protein homolog intensity over time. The bold black line indicates the mean intensity over time. B) Box plots depicting the distribution of Out at first protein homolog intensities at baseline, month 1, and month 2. Only AMD patients with measurements at all visits are included. The median, interquartile range, and outliers are displayed for each time point. Abbreviations: FDR, false discovery rate; ns, non−significant; * p < 0.05; ** p < 0.01; *** p < 0.001.

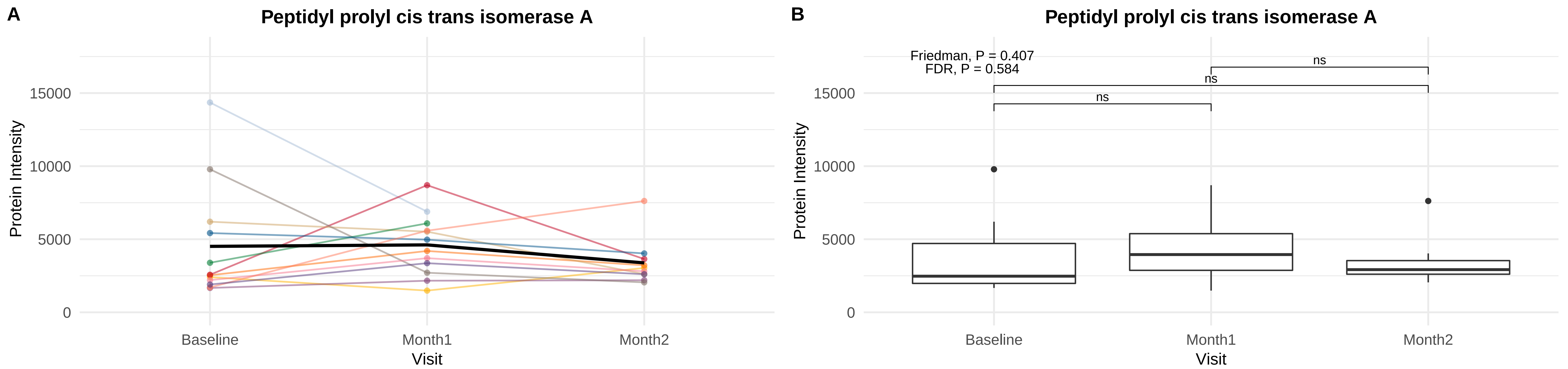

Supplementary Figure S 217

A) Line plot illustrating individual patient trajectories of Peptidyl prolyl cis trans isomerase A intensity over time. The bold black line indicates the mean intensity over time. B) Box plots depicting the distribution of Peptidyl prolyl cis trans isomerase A intensities at baseline, month 1, and month 2. Only AMD patients with measurements at all visits are included. The median, interquartile range, and outliers are displayed for each time point. Abbreviations: FDR, false discovery rate; ns, non−significant; * p < 0.05; ** p < 0.01; *** p < 0.001.

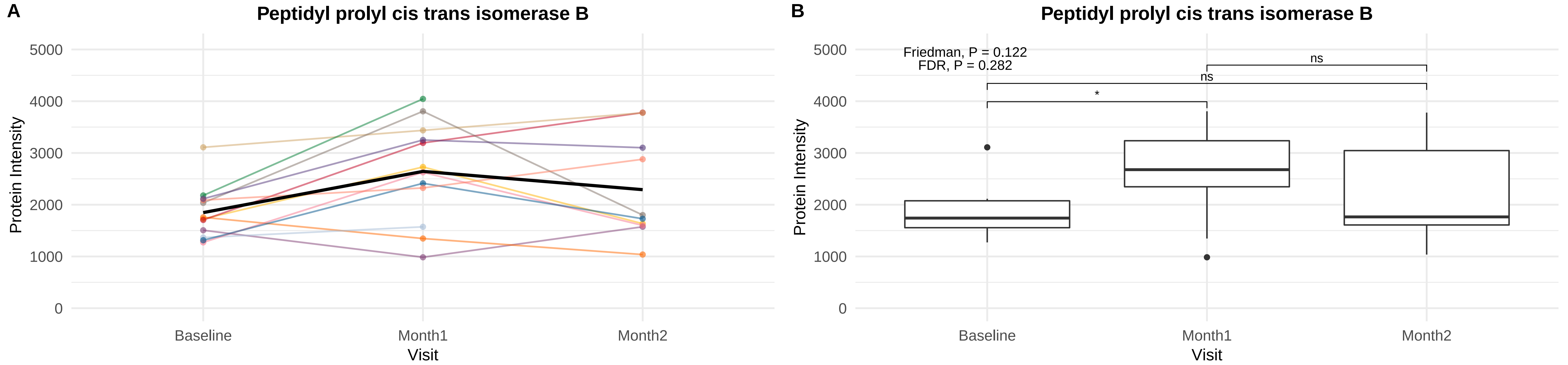

Supplementary Figure S 218

A) Line plot illustrating individual patient trajectories of Peptidyl prolyl cis trans isomerase B intensity over time. The bold black line indicates the mean intensity over time. B) Box plots depicting the distribution of Peptidyl prolyl cis trans isomerase B intensities at baseline, month 1, and month 2. Only AMD patients with measurements at all visits are included. The median, interquartile range, and outliers are displayed for each time point. Abbreviations: FDR, false discovery rate; ns, non−significant; * p < 0.05; ** p < 0.01; *** p < 0.001.

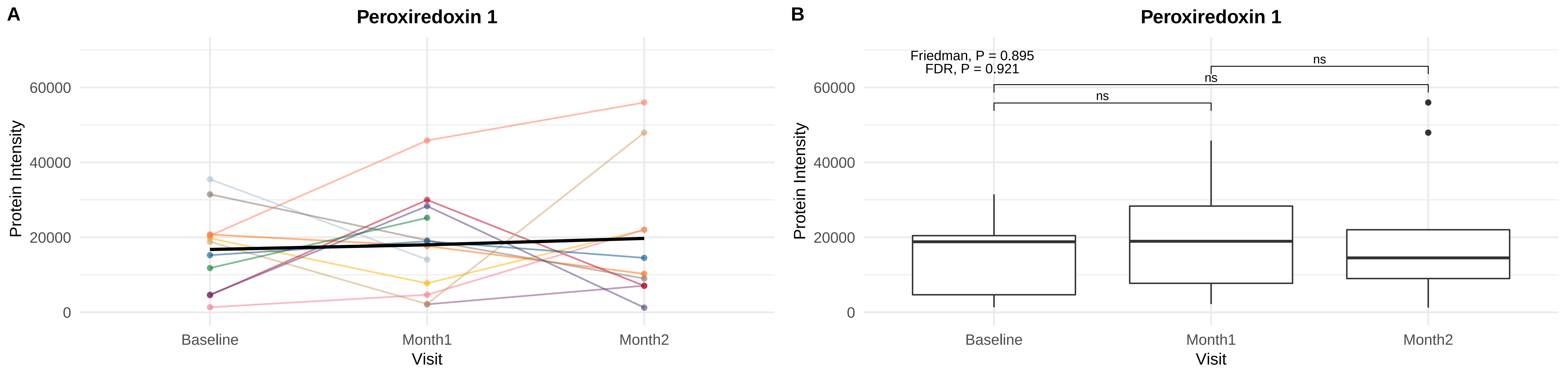

Supplementary Figure S 219

A) Line plot illustrating individual patient trajectories of Peroxiredoxin 1 intensity over time. The bold black line indicates the mean intensity over time. B) Box plots depicting the distribution of Peroxiredoxin 1 intensities at baseline, month 1, and month 2. Only AMD patients with measurements at all visits are included. The median, interquartile range, and outliers are displayed for each time point. Abbreviations: FDR, false discovery rate; ns, non−significant; * p < 0.05; ** p < 0.01; *** p < 0.001.

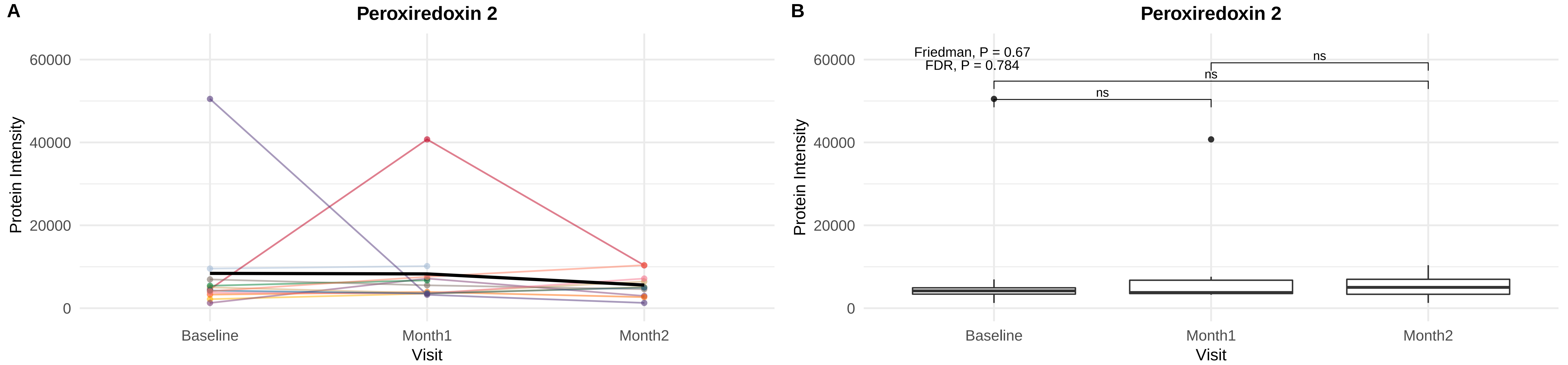

Supplementary Figure S 220

A) Line plot illustrating individual patient trajectories of Peroxiredoxin 2 intensity over time. The bold black line indicates the mean intensity over time. B) Box plots depicting the distribution of Peroxiredoxin 2 intensities at baseline, month 1, and month 2. Only AMD patients with measurements at all visits are included. The median, interquartile range, and outliers are displayed for each time point. Abbreviations: FDR, false discovery rate; ns, non−significant; * p < 0.05; ** p < 0.01; *** p < 0.001.

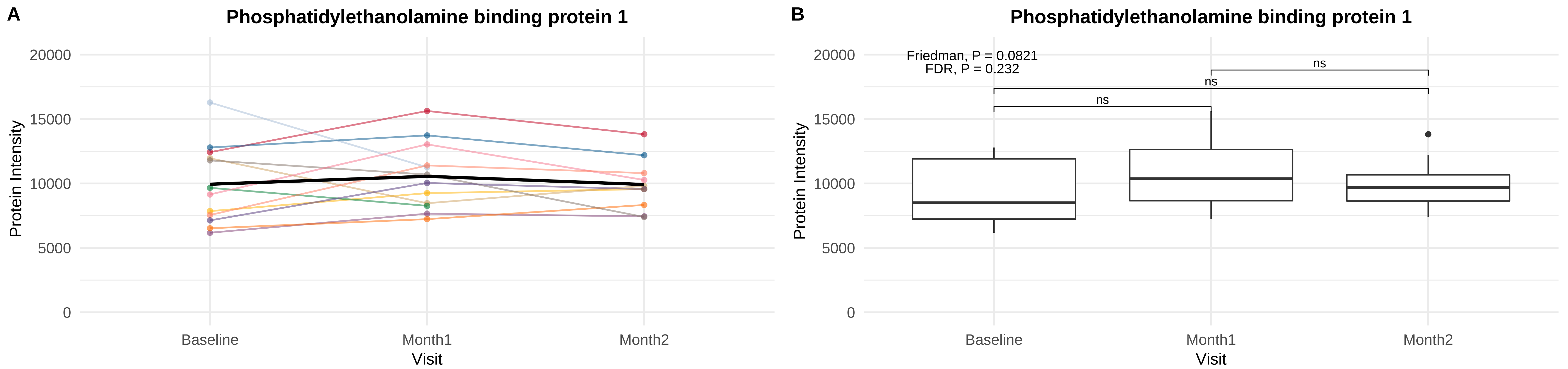

Supplementary Figure S 221

A) Line plot illustrating individual patient trajectories of Phosphatidylethanolamine binding protein 1 intensity over time. The bold black line indicates the mean intensity over time. B) Box plots depicting the distribution of Phosphatidylethanolamine binding protein 1 intensities at baseline, month 1, and month 2. Only AMD patients with measurements at all visits are included. The median, interquartile range, and outliers are displayed for each time point. Abbreviations: FDR, false discovery rate; ns, non−significant; * p < 0.05; ** p < 0.01; *** p < 0.001.

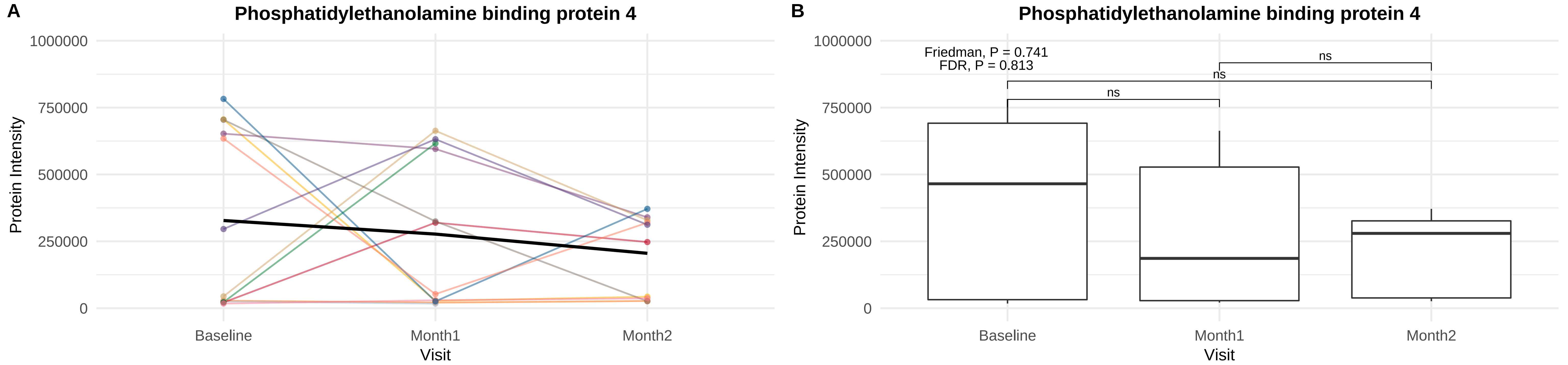

Supplementary Figure S 222

A) Line plot illustrating individual patient trajectories of Phosphatidylethanolamine binding protein 4 intensity over time. The bold black line indicates the mean intensity over time. B) Box plots depicting the distribution of Phosphatidylethanolamine binding protein 4 intensities at baseline, month 1, and month 2. Only AMD patients with measurements at all visits are included. The median, interquartile range, and outliers are displayed for each time point. Abbreviations: FDR, false discovery rate; ns, non−significant; * p < 0.05; ** p < 0.01; *** p < 0.001.

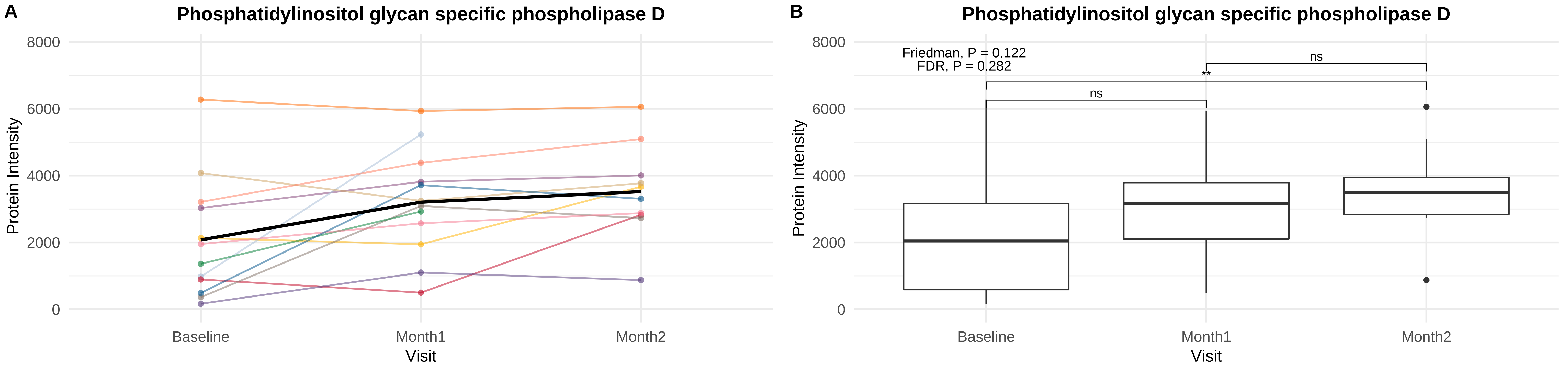

Supplementary Figure S 223

A) Line plot illustrating individual patient trajectories of Phosphatidylinositol glycan specific phospholipase D intensity over time. The bold black line indicates the mean intensity over time. B) Box plots depicting the distribution of Phosphatidylinositol glycan specific phospholipase D intensities at baseline, month 1, and month 2. Only AMD patients with measurements at all visits are included. The median, interquartile range, and outliers are displayed for each time point. Abbreviations: FDR, false discovery rate; ns, non−significant; * p < 0.05; ** p < 0.01; *** p < 0.001.

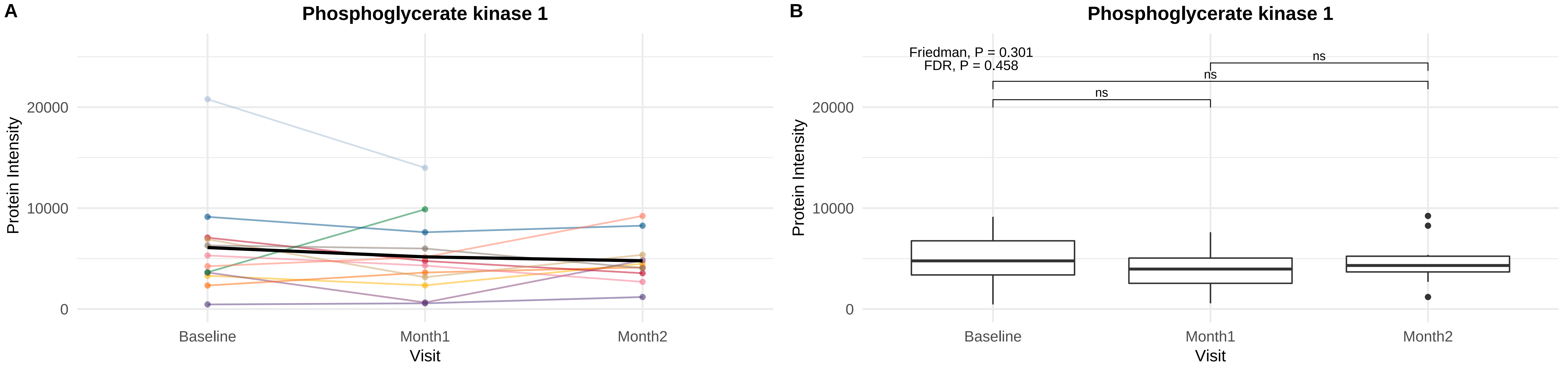

Supplementary Figure S 224

A) Line plot illustrating individual patient trajectories of Phosphoglycerate kinase 1 intensity over time. The bold black line indicates the mean intensity over time. B) Box plots depicting the distribution of Phosphoglycerate kinase 1 intensities at baseline, month 1, and month 2. Only AMD patients with measurements at all visits are included. The median, interquartile range, and outliers are displayed for each time point. Abbreviations: FDR, false discovery rate; ns, non−significant; * p < 0.05; ** p < 0.01; *** p < 0.001.

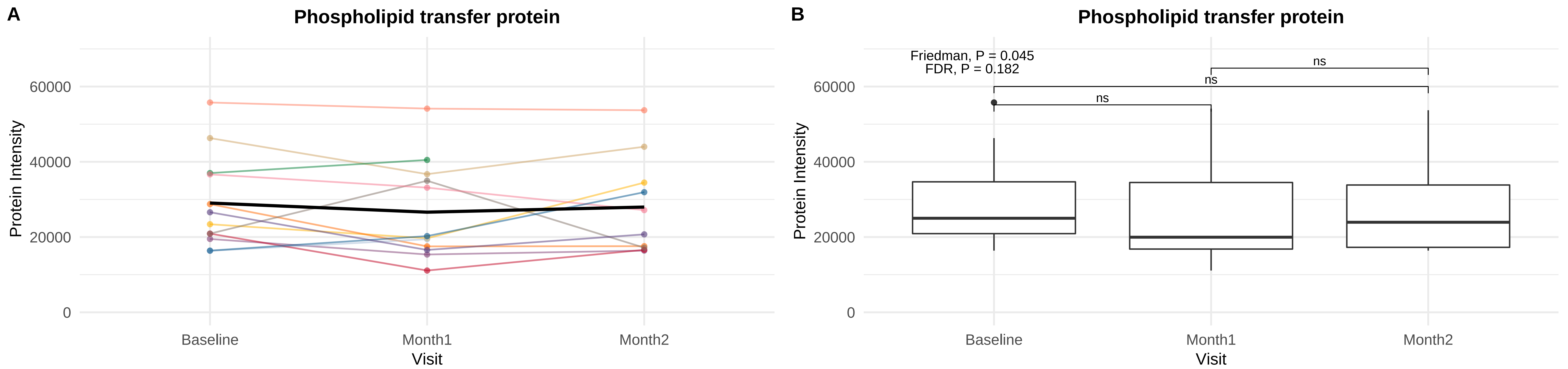

Supplementary Figure S 225

A) Line plot illustrating individual patient trajectories of Phospholipid transfer protein intensity over time. The bold black line indicates the mean intensity over time. B) Box plots depicting the distribution of Phospholipid transfer protein intensities at baseline, month 1, and month 2. Only AMD patients with measurements at all visits are included. The median, interquartile range, and outliers are displayed for each time point. Abbreviations: FDR, false discovery rate; ns, non−significant; * $p < 0.05$; ** $p < 0.01$; *** $p < 0.001$.

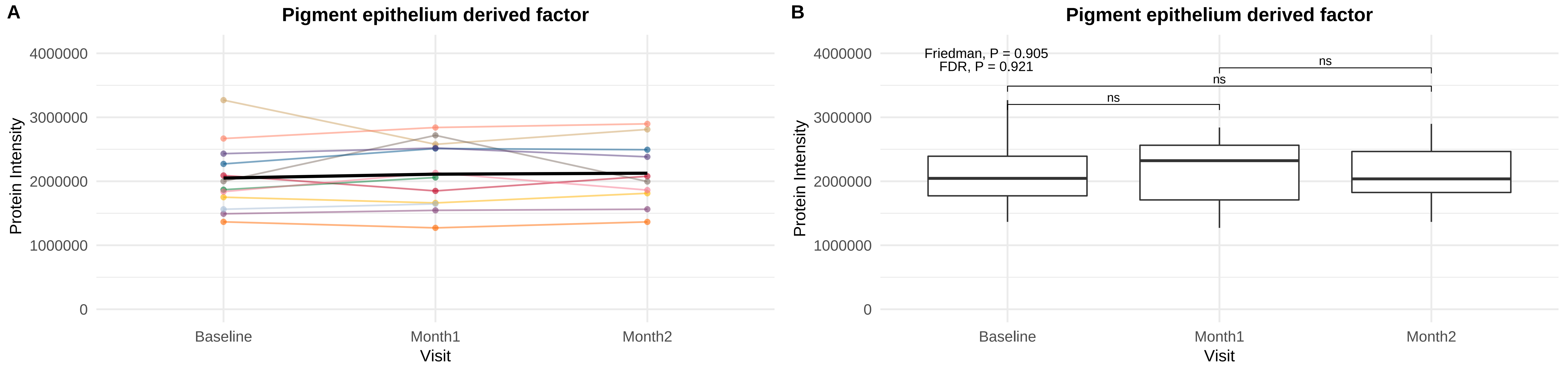

Supplementary Figure S 226

A) Line plot illustrating individual patient trajectories of Pigment epithelium derived factor intensity over time. The bold black line indicates the mean intensity over time. B) Box plots depicting the distribution of Pigment epithelium derived factor intensities at baseline, month 1, and month 2. Only AMD patients with measurements at all visits are included. The median, interquartile range, and outliers are displayed for each time point. Abbreviations: FDR, false discovery rate; ns, non−significant; * $p < 0.05$; ** $p < 0.01$; *** $p < 0.001$.

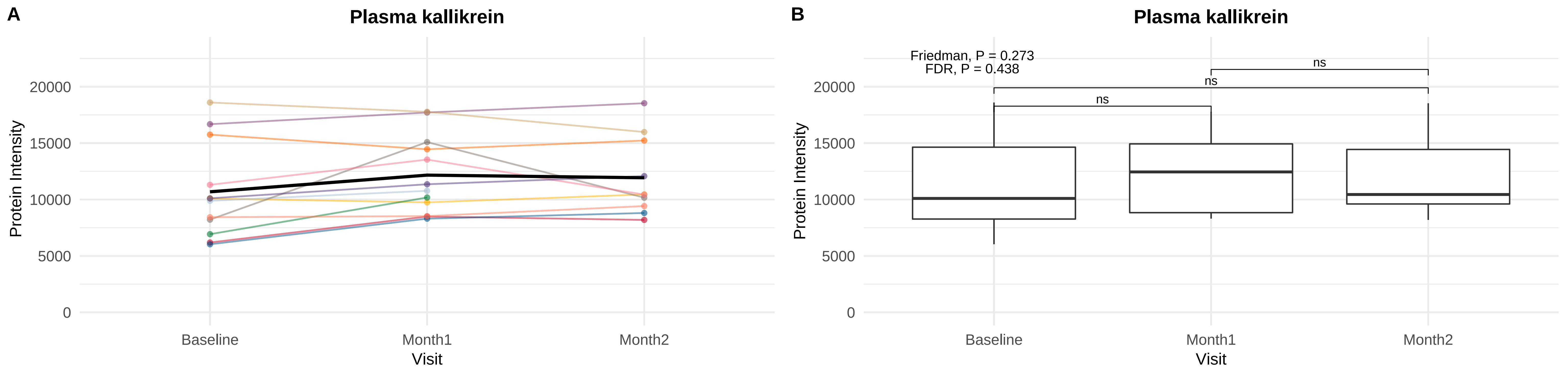

Supplementary Figure S 227

A) Line plot illustrating individual patient trajectories of Plasma kallikrein intensity over time. The bold black line indicates the mean intensity over time. B) Box plots depicting the distribution of Plasma kallikrein intensities at baseline, month 1, and month 2. Only AMD patients with measurements at all visits are included. The median, interquartile range, and outliers are displayed for each time point. Abbreviations: FDR, false discovery rate; ns, non−significant; * p < 0.05; ** p < 0.01; *** p < 0.001.

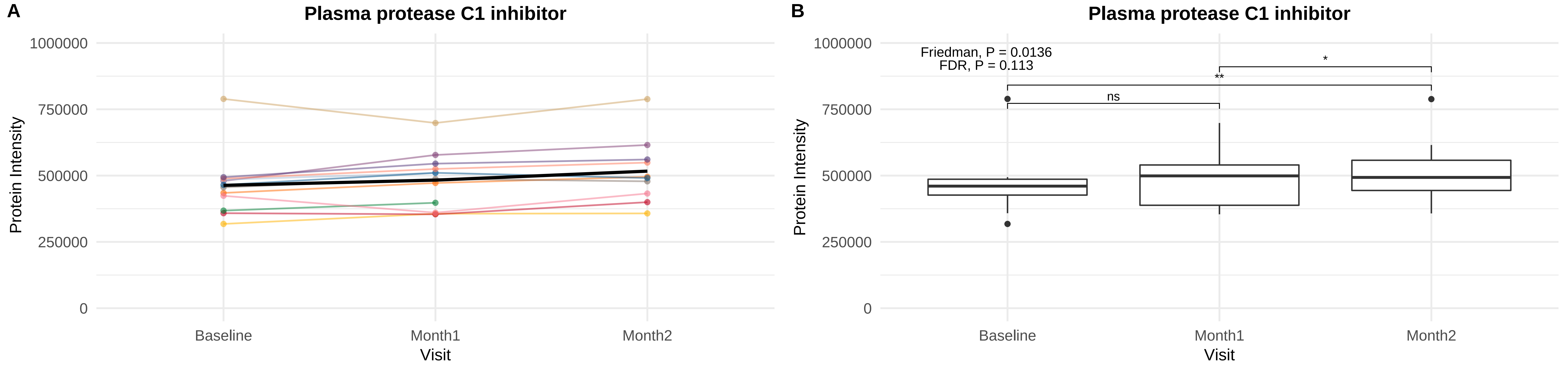

Supplementary Figure S 228

A) Line plot illustrating individual patient trajectories of Plasma protease C1 inhibitor intensity over time. The bold black line indicates the mean intensity over time. B) Box plots depicting the distribution of Plasma protease C1 inhibitor intensities at baseline, month 1, and month 2. Only AMD patients with measurements at all visits are included. The median, interquartile range, and outliers are displayed for each time point. Abbreviations: FDR, false discovery rate; ns, non−significant; * p < 0.05; ** p < 0.01; *** p < 0.001.

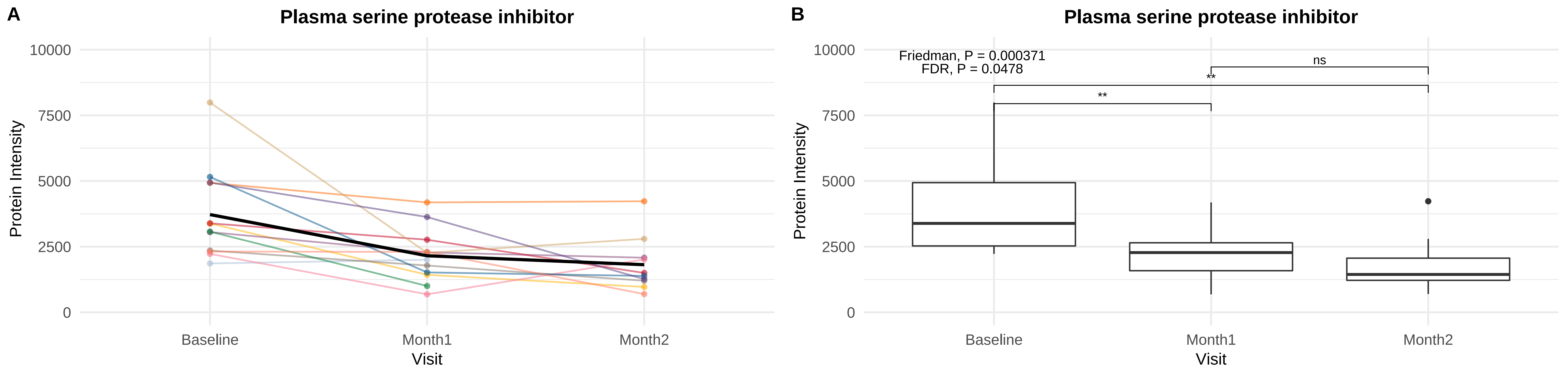

Supplementary Figure S 229

A) Line plot illustrating individual patient trajectories of Plasma serine protease inhibitor intensity over time. The bold black line indicates the mean intensity over time. B) Box plots depicting the distribution of Plasma serine protease inhibitor intensities at baseline, month 1, and month 2. Only AMD patients with measurements at all visits are included. The median, interquartile range, and outliers are displayed for each time point. Abbreviations: FDR, false discovery rate; ns, non−significant; * p < 0.05; ** p < 0.01; *** p < 0.001.

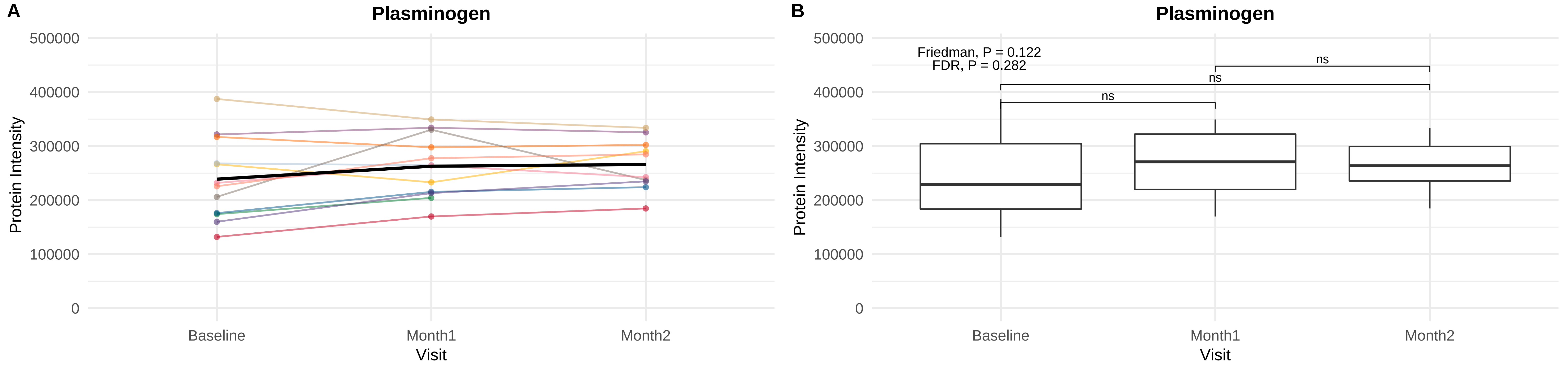

Supplementary Figure S 230

A) Line plot illustrating individual patient trajectories of Plasminogen intensity over time. The bold black line indicates the mean intensity over time. B) Box plots depicting the distribution of Plasminogen intensities at baseline, month 1, and month 2. Only AMD patients with measurements a all visits are included. The median, interquartile range, and outliers are displayed for each time point. Abbreviations: FDR, false discovery rate; ns, non−significant; * p < 0.05; ** p < 0.01; *** p < 0.001.

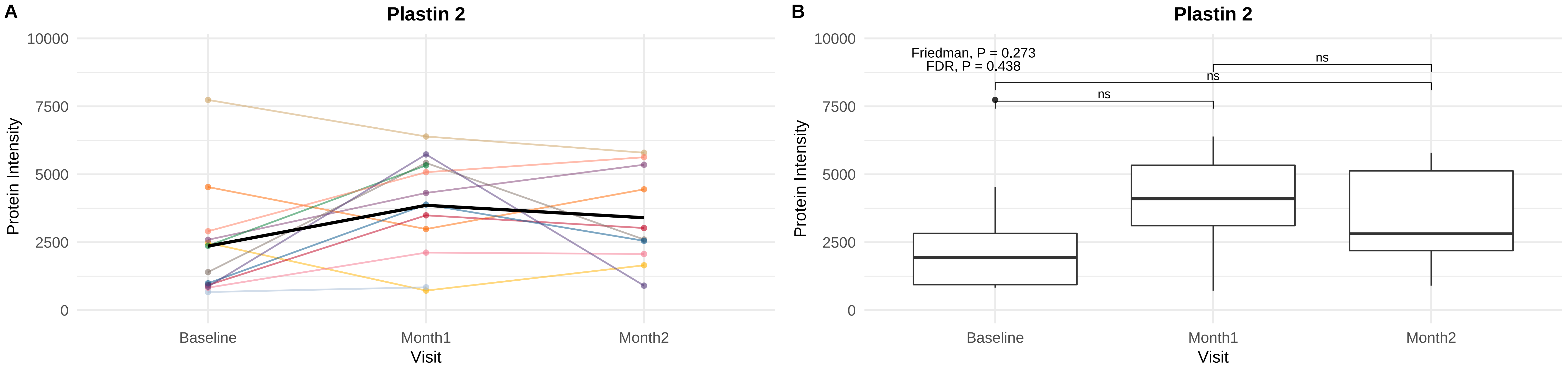

Supplementary Figure S 231

A) Line plot illustrating individual patient trajectories of Plastin 2 intensity over time. The bold black line indicates the mean intensity over time. B) Box plots depicting the distribution of Plastin 2 intensities at baseline, month 1, and month 2. Only AMD patients with measurements at all visits are included. The median, interquartile range, and outliers are displayed for each time point. Abbreviations: FDR, false discovery rate; ns, non−significant; * p < 0.05; ** p < 0.01; *** p < 0.001.

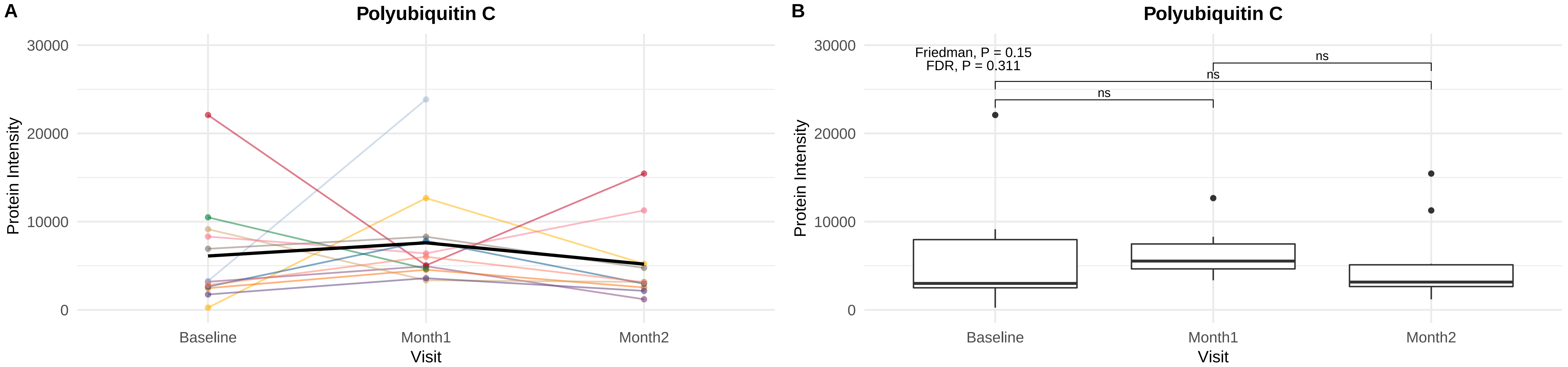

Supplementary Figure S 232

A) Line plot illustrating individual patient trajectories of Polyubiquitin C intensity over time. The bold black line indicates the mean intensity over time. B) Box plots depicting the distribution of Polyubiquitin C intensities at baseline, month 1, and month 2. Only AMD patients with measurements at all visits are included. The median, interquartile range, and outliers are displayed for each time point. Abbreviations: FDR, false discovery rate; ns, non−significant; * p < 0.05; ** p < 0.01; *** p < 0.001.

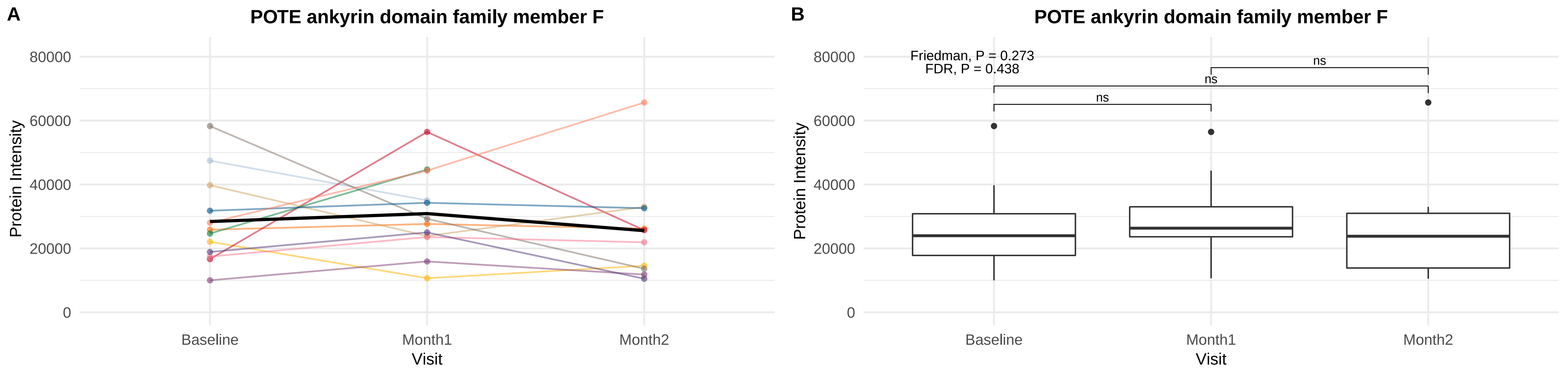

Supplementary Figure S 233

A) Line plot illustrating individual patient trajectories of POTE ankyrin domain family member F intensity over time. The bold black line indicates the mean intensity over time. B) Box plots depicting the distribution of POTE ankyrin domain family member F intensities at baseline, month 1, a[illegible] month 2. Only AMD patients with measurements at all visits are included. The median, interquartile range, and outliers are displayed for each time point. Abbreviations: FDR, false discovery rate; ns, non−significant; * p < 0.05; ** p < 0.01; *** p < 0.001.

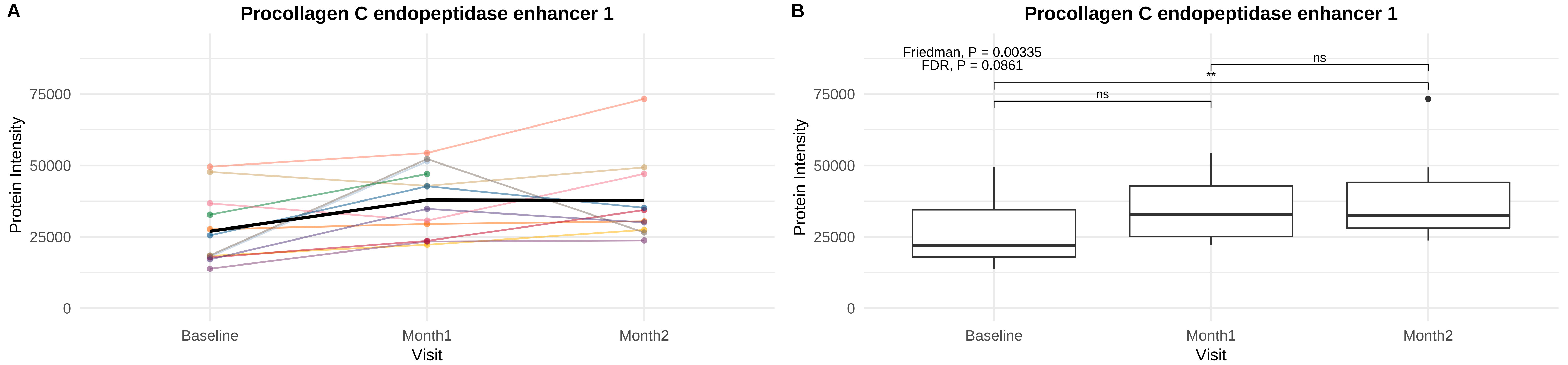

Supplementary Figure S 234

A) Line plot illustrating individual patient trajectories of Procollagen C endopeptidase enhancer 1 intensity over time. The bold black line indicates the mean intensity over time. B) Box plots depicting the distribution of Procollagen C endopeptidase enhancer 1 intensities at baseline, month 1, and month 2. Only AMD patients with measurements at all visits are included. The median, interquartile range, and outliers are displayed for each time point. Abbreviations: FDR, false discovery rate; ns, non−significant; * p < 0.05; ** p < 0.01; *** p < 0.001.

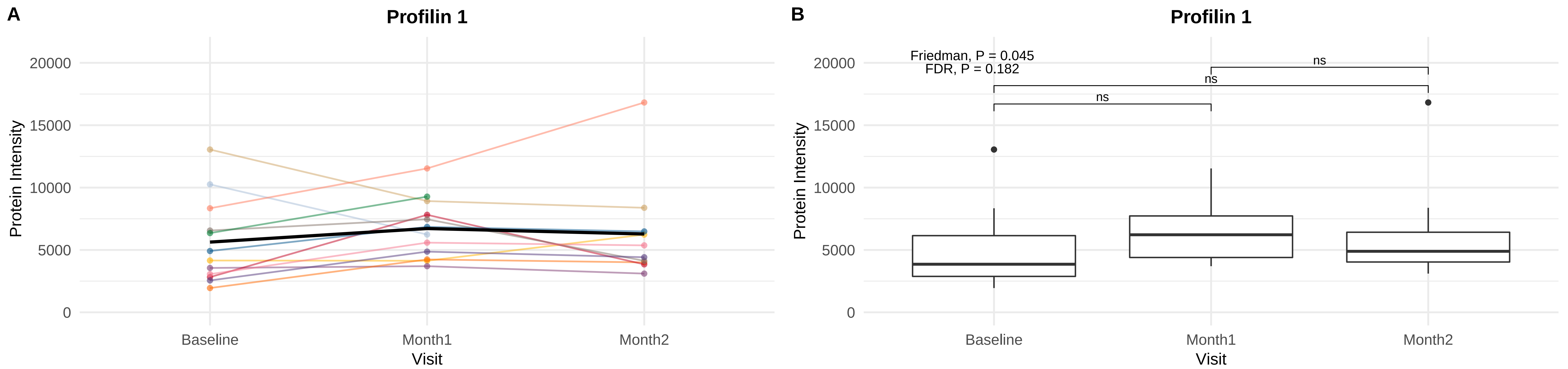

Supplementary Figure S 235

A) Line plot illustrating individual patient trajectories of Profilin 1 intensity over time. The bold black line indicates the mean intensity over time. B) Box plots depicting the distribution of Profilin 1 intensities at baseline, month 1, and month 2. Only AMD patients with measurements at all visits are included. The median, interquartile range, and outliers are displayed for each time point. Abbreviations: FDR, false discovery rate; ns, non−significant; * p < 0.05; ** p < 0.01; *** p < 0.001.

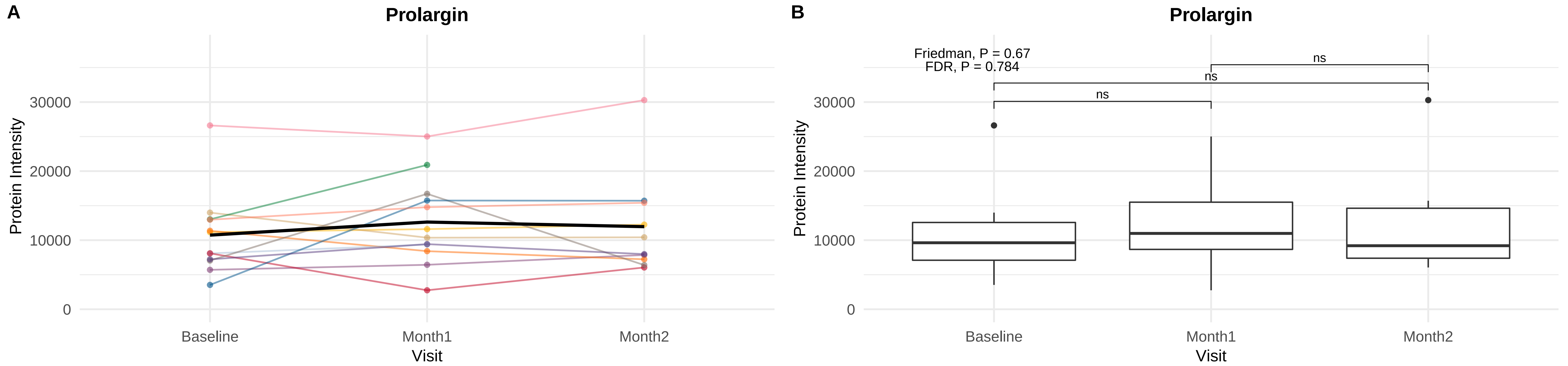

Supplementary Figure S 236

A) Line plot illustrating individual patient trajectories of Prolargin intensity over time. The bold black line indicates the mean intensity over time. B) Box plots depicting the distribution of Prolargin intensities at baseline, month 1, and month 2. Only AMD patients with measurements at all visits are included. The median, interquartile range, and outliers are displayed for each time point. Abbreviations: FDR, false discovery rate; ns, non−significant; * p < 0.05; ** p < 0.01; *** p < 0.001.

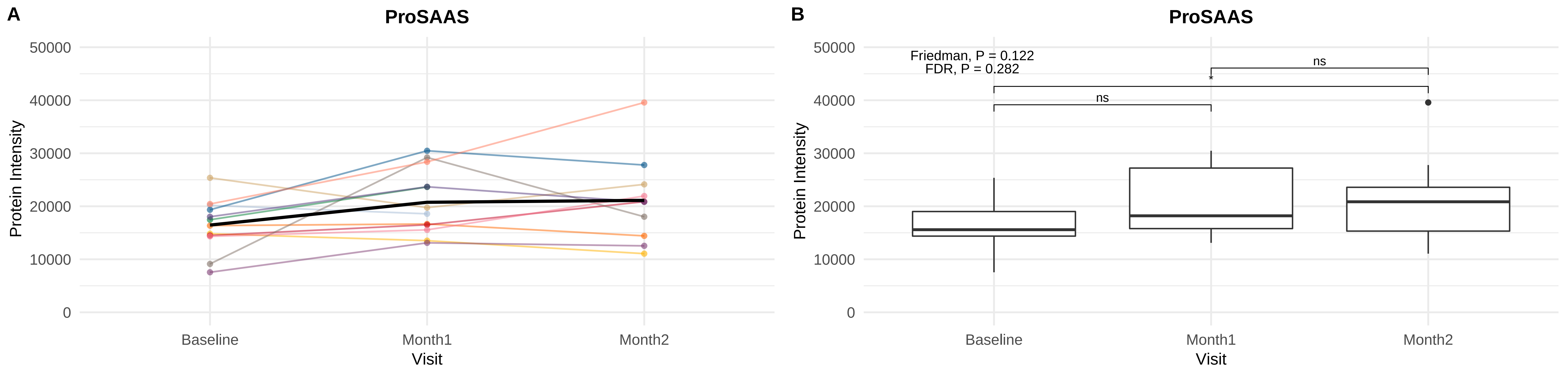

Supplementary Figure S 237

A) Line plot illustrating individual patient trajectories of ProSAAS intensity over time. The bold black line indicates the mean intensity over time. B) Box plots depicting the distribution of ProSAAS intensities at baseline, month 1, and month 2. Only AMD patients with measurements at all visits are included. The median, interquartile range, and outliers are displayed for each time point. Abbreviations: FDR, false discovery rate; ns, non−significant; * p < 0.05; ** p < 0.01; *** p < 0.001.

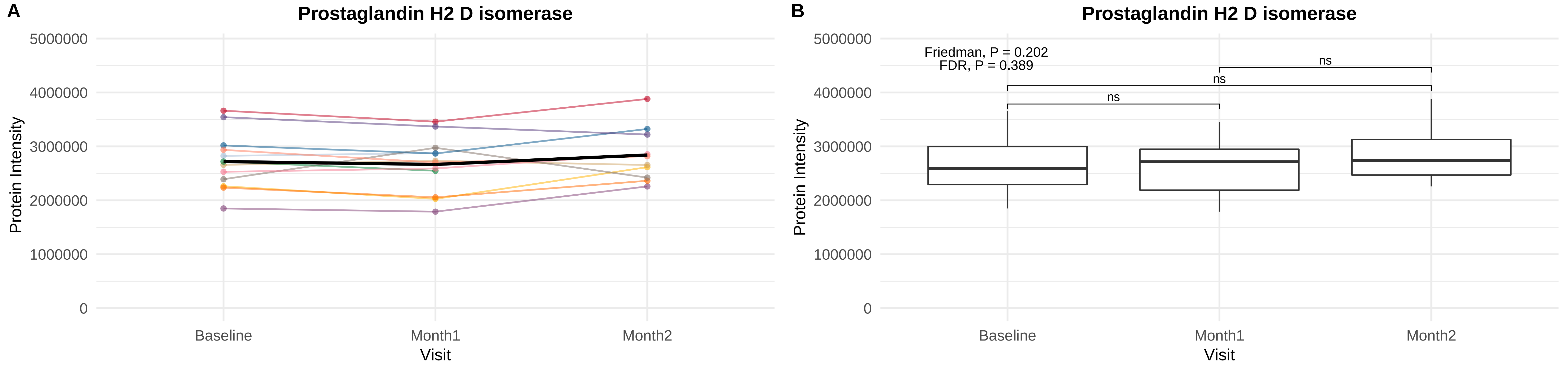

Supplementary Figure S 238

A) Line plot illustrating individual patient trajectories of Prostaglandin H2 D isomerase intensity over time. The bold black line indicates the mean intensity over time. B) Box plots depicting the distribution of Prostaglandin H2 D isomerase intensities at baseline, month 1, and month 2. Only AMD patients with measurements at all visits are included. The median, interquartile range, and outliers are displayed for each time point. Abbreviations: FDR, false discovery rate; ns, non−significant; * p < 0.05; ** p < 0.01; *** p < 0.001.

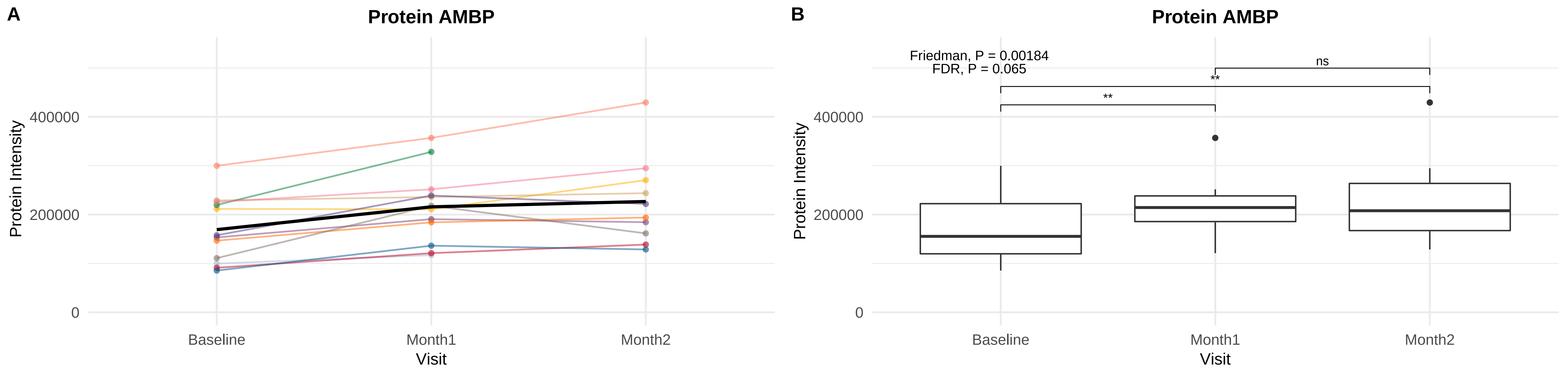

Supplementary Figure S 239

A) Line plot illustrating individual patient trajectories of Protein AMBP intensity over time. The bold black line indicates the mean intensity over time. B) Box plots depicting the distribution of Protein AMBP intensities at baseline, month 1, and month 2. Only AMD patients with measurements at all visits are included. The median, interquartile range, and outliers are displayed for each time point. Abbreviations: FDR, false discovery rate; ns, non−significant; * p < 0.05; ** p < 0.01; *** p < 0.001.

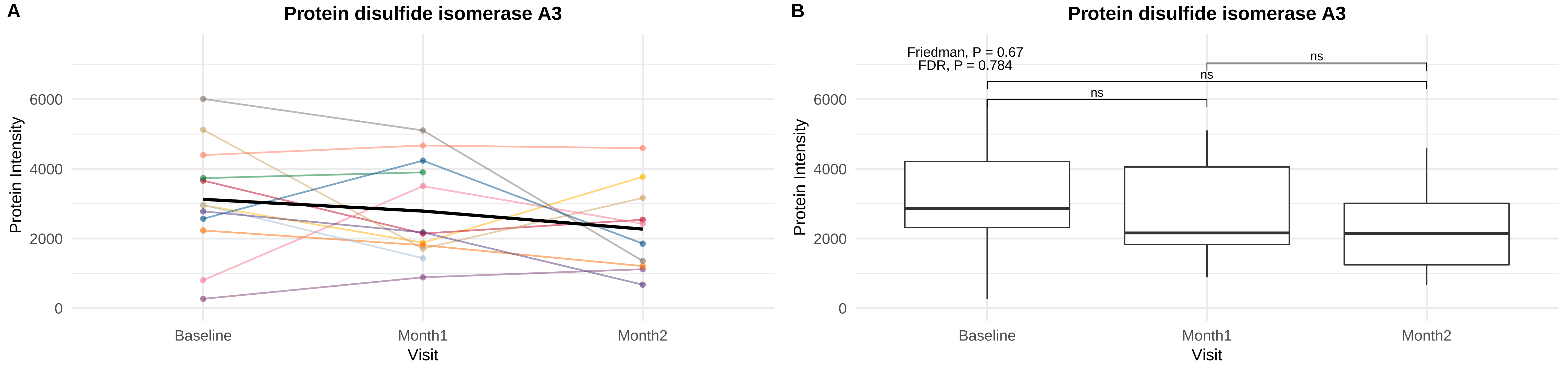

Supplementary Figure S 240

A) Line plot illustrating individual patient trajectories of Protein disulfide isomerase A3 intensity over time. The bold black line indicates the mean intensity over time. B) Box plots depicting the distribution of Protein disulfide isomerase A3 intensities at baseline, month 1, and month 2. Only AMD patients with measurements at all visits are included. The median, interquartile range, and outliers are displayed for each time point. Abbreviations: FDR, false discovery rate; ns, non−significant; * p < 0.05; ** p < 0.01; *** p < 0.001.

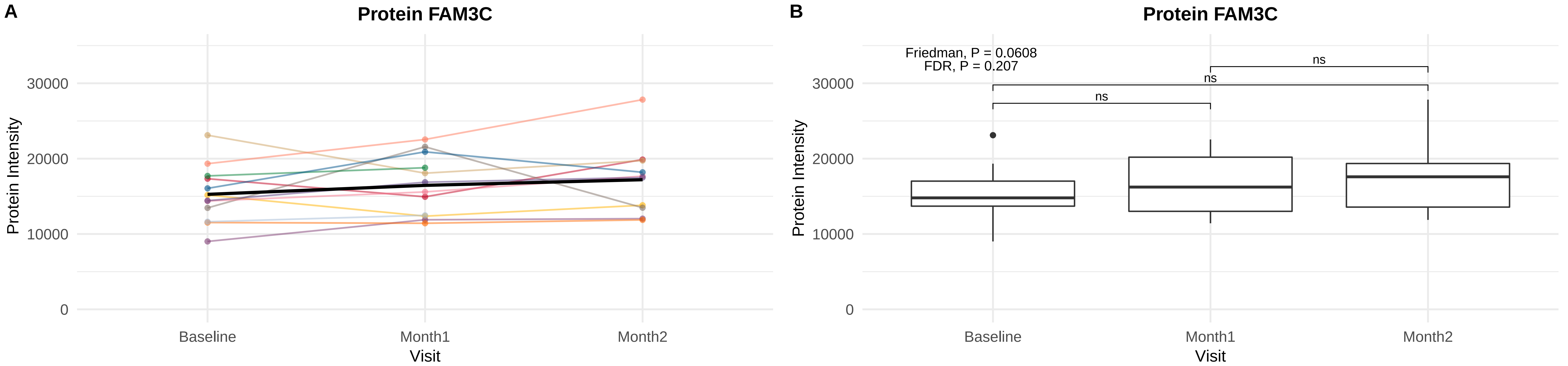

Supplementary Figure S 241

A) Line plot illustrating individual patient trajectories of Protein FAM3C intensity over time. The bold black line indicates the mean intensity over time. B) Box plots depicting the distribution of Protein FAM3C intensities at baseline, month 1, and month 2. Only AMD patients with measureme at all visits are included. The median, interquartile range, and outliers are displayed for each time point. Abbreviations: FDR, false discovery rate; ns, non−significant; * p < 0.05; ** p < 0.01; *** p < 0.001.

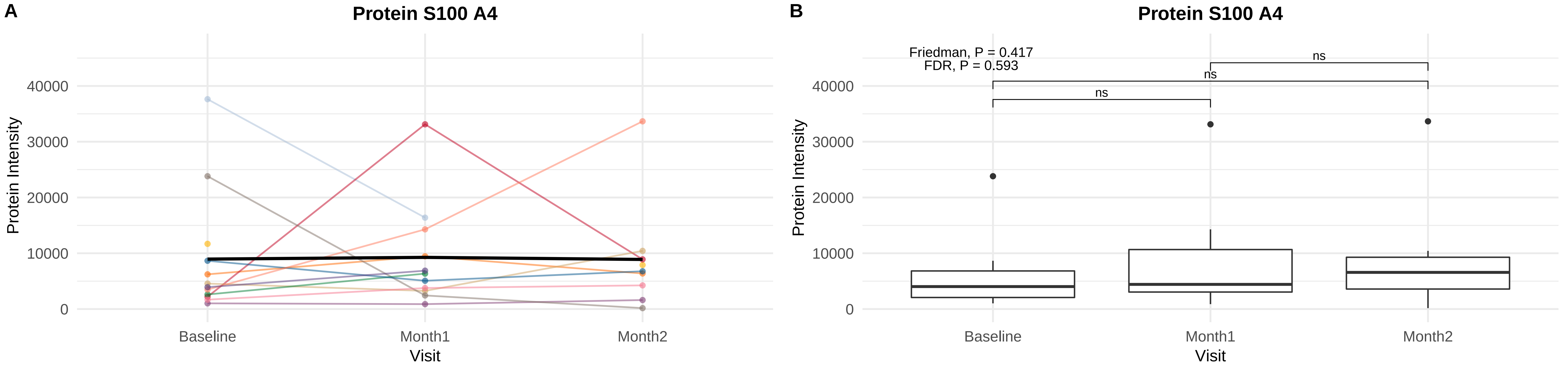

Supplementary Figure S 242

A) Line plot illustrating individual patient trajectories of Protein S100 A4 intensity over time. The bold black line indicates the mean intensity over time. B) Box plots depicting the distribution of Protein S100 A4 intensities at baseline, month 1, and month 2. Only AMD patients with measurements at all visits are included. The median, interquartile range, and outliers are displayed for each time point. Abbreviations: FDR, false discovery rate; ns, non−significant; * p < 0.05; ** p < 0.01; *** p < 0.001.

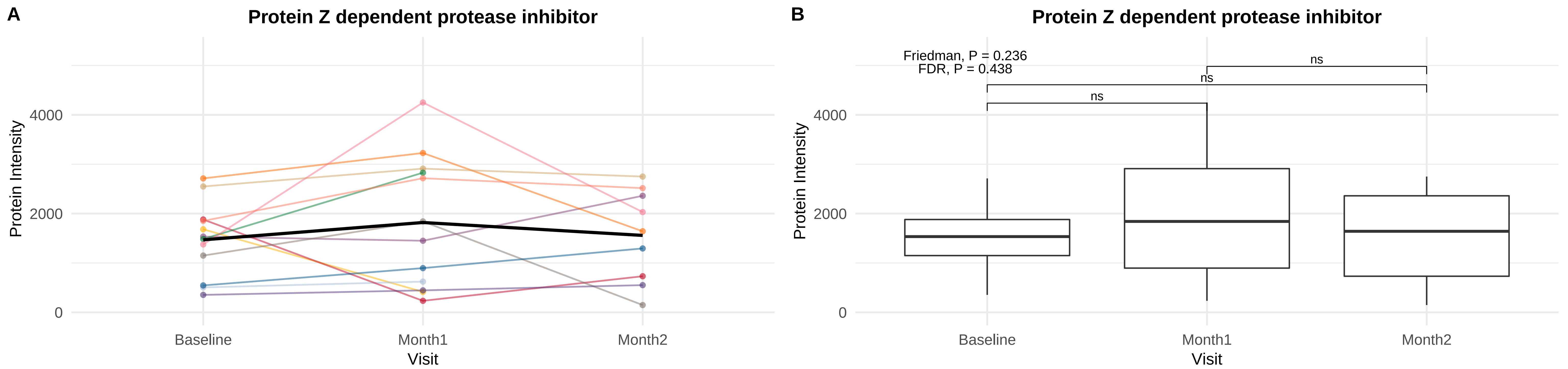

Supplementary Figure S 243

A) Line plot illustrating individual patient trajectories of Protein Z dependent protease inhibitor intensity over time. The bold black line indicates the mean intensity over time. B) Box plots depicting the distribution of Protein Z dependent protease inhibitor intensities at baseline, month 1, and month 2. Only AMD patients with measurements at all visits are included. The median, interquartile range, and outliers are displayed for each time point. Abbreviations: FDR, false discovery rate; ns, non−significant; * p < 0.05; ** p < 0.01; *** p < 0.001.

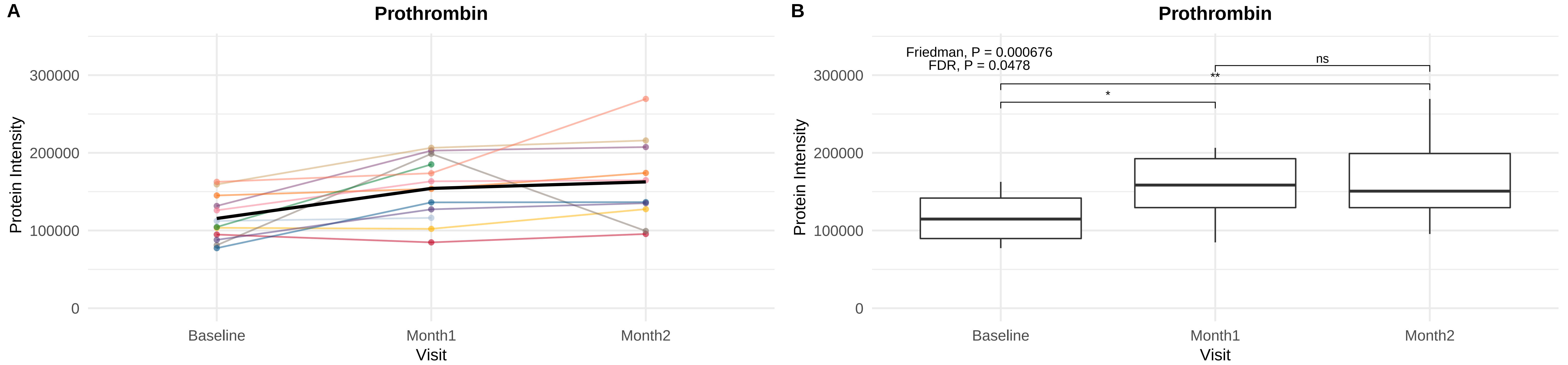

Supplementary Figure S 244

A) Line plot illustrating individual patient trajectories of Prothrombin intensity over time. The bold black line indicates the mean intensity over time. B) Box plots depicting the distribution of Prothrombin intensities at baseline, month 1, and month 2. Only AMD patients with measurements at all visits are included. The median, interquartile range, and outliers are displayed for each time point. Abbreviations: FDR, false discovery rate; ns, non−significant; * p < 0.05; ** p < 0.01; *** p < 0.001.

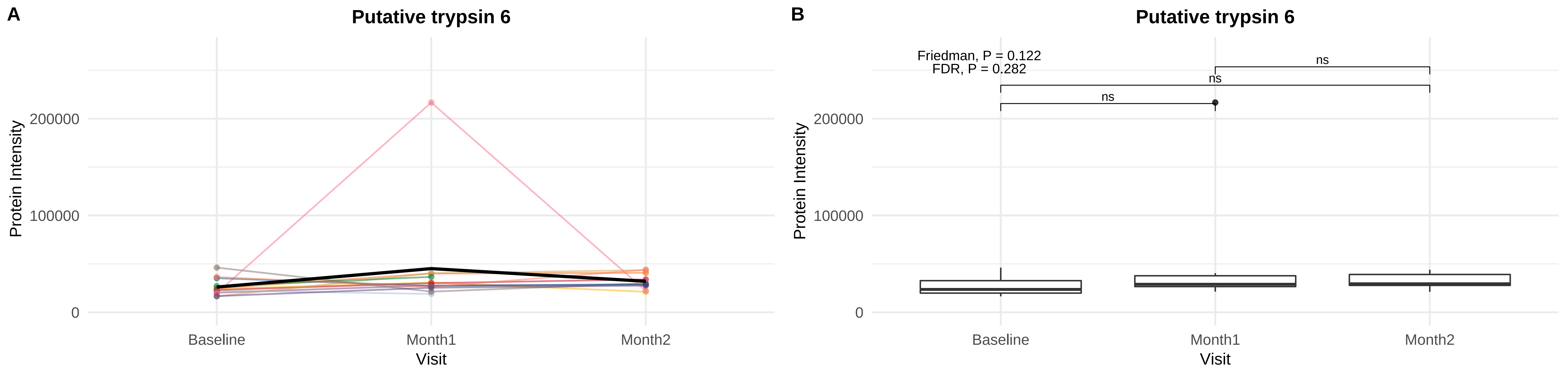

Supplementary Figure S 245

A) Line plot illustrating individual patient trajectories of Putative trypsin 6 intensity over time. The bold black line indicates the mean intensity over time. B) Box plots depicting the distribution of Putative trypsin 6 intensities at baseline, month 1, and month 2. Only AMD patients with measurements at all visits are included. The median, interquartile range, and outliers are displayed for each time point. Abbreviations: FDR, false discovery rate; ns, non−significant; * p < 0.05; ** p < 0.01; *** p < 0.001.

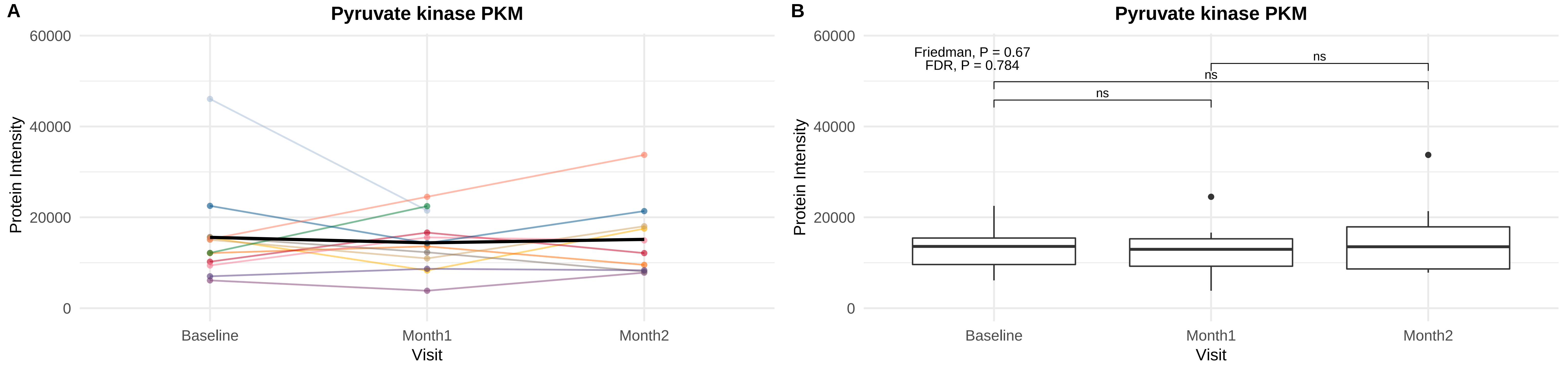

Supplementary Figure S 246

A) Line plot illustrating individual patient trajectories of Pyruvate kinase PKM intensity over time. The bold black line indicates the mean intensity over time. B) Box plots depicting the distribution of Pyruvate kinase PKM intensities at baseline, month 1, and month 2. Only AMD patients with measurements at all visits are included. The median, interquartile range, and outliers are displayed for each time point. Abbreviations: FDR, false discovery rate; ns, non−significant; * p < 0.05; ** p < 0.01; *** p < 0.001.

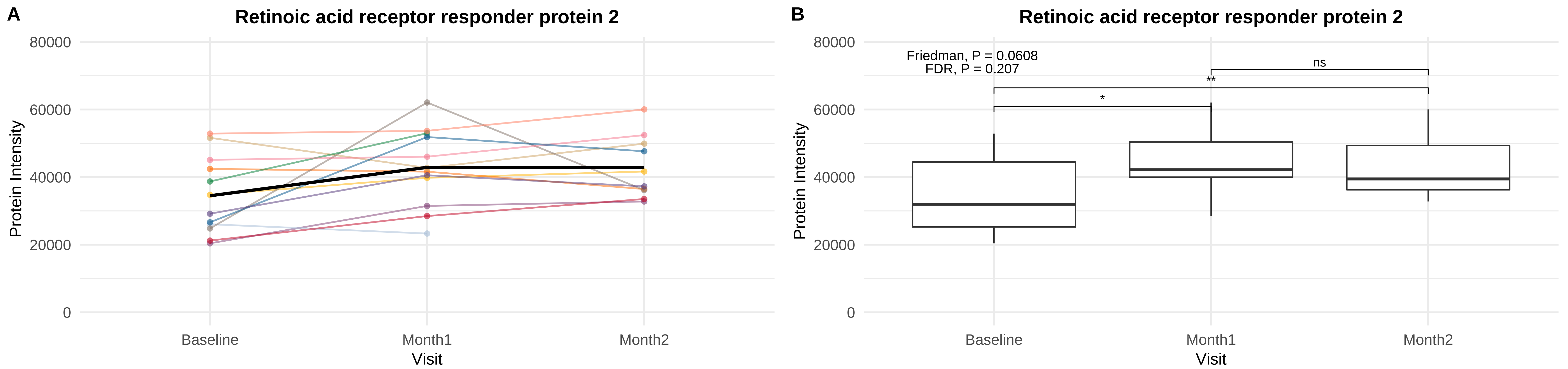

Supplementary Figure S 247

A) Line plot illustrating individual patient trajectories of Retinoic acid receptor responder protein 2 intensity over time. The bold black line indicates the mean intensity over time. B) Box plots depicting the distribution of Retinoic acid receptor responder protein 2 intensities at baseline, month 1, and month 2. Only AMD patients with measurements at all visits are included. The median, interquartile range, and outliers are displayed for each time point. Abbreviations: FDR, false discovery rate; ns, non−significant; * p < 0.05; ** p < 0.01; *** p < 0.001.

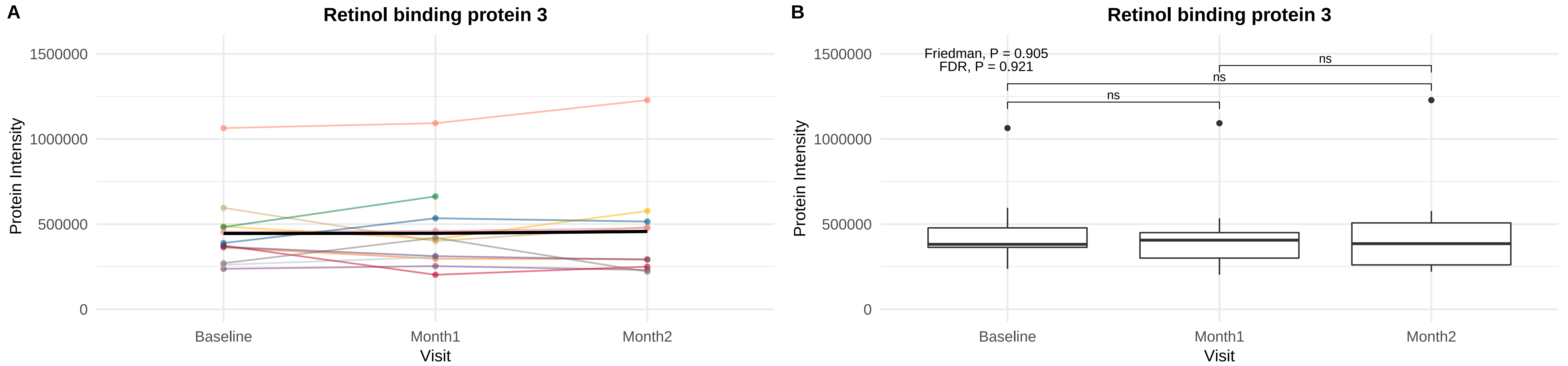

Supplementary Figure S 248

A) Line plot illustrating individual patient trajectories of Retinol binding protein 3 intensity over time. The bold black line indicates the mean intensity over time. B) Box plots depicting the distribution of Retinol binding protein 3 intensities at baseline, month 1, and month 2. Only AMD patients with measurements at all visits are included. The median, interquartile range, and outliers are displayed for each time point. Abbreviations: FDR, false discovery rate; ns, non−significant; * p < 0.05; ** p < 0.01; *** p < 0.001.

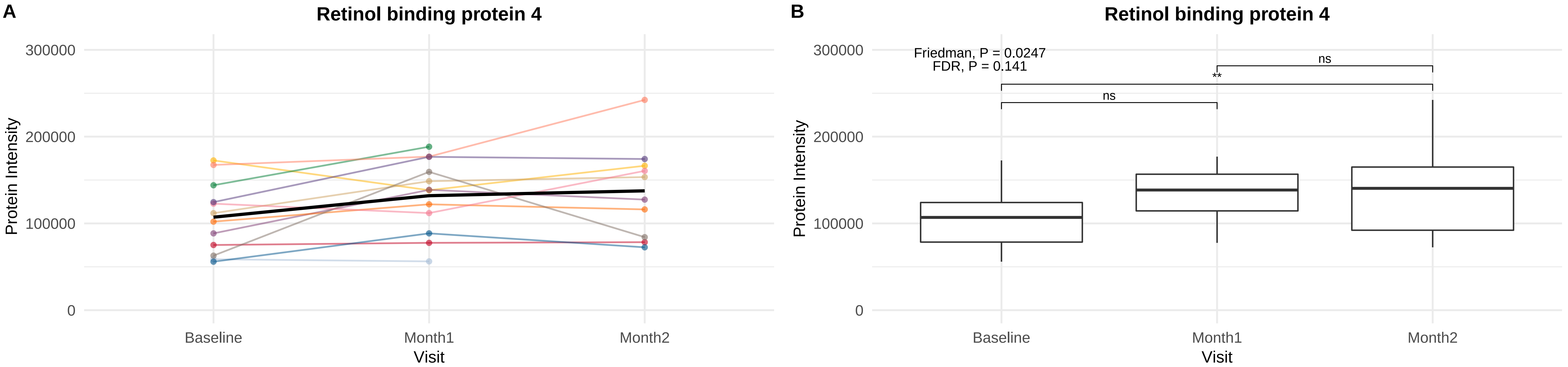

Supplementary Figure S 249

A) Line plot illustrating individual patient trajectories of Retinol binding protein 4 intensity over time. The bold black line indicates the mean intensity over time. B) Box plots depicting the distribution of Retinol binding protein 4 intensities at baseline, month 1, and month 2. Only AMD patients with measurements at all visits are included. The median, interquartile range, and outliers are displayed for each time point. Abbreviations: FDR, false discovery rate; ns, non−significant; * p < 0.05; ** p < 0.01; *** p < 0.001.

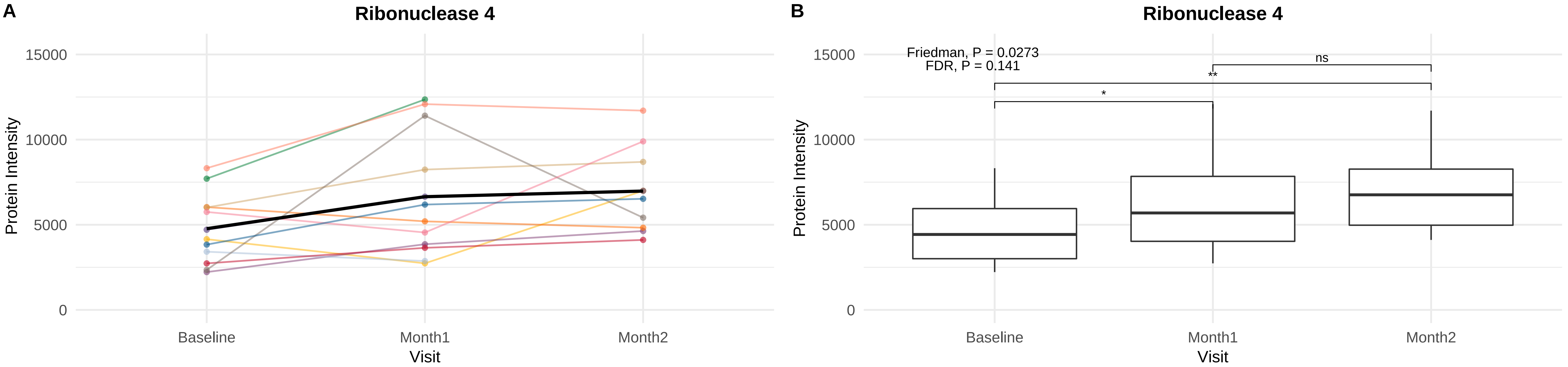

Supplementary Figure S 250

A) Line plot illustrating individual patient trajectories of Ribonuclease 4 intensity over time. The bold black line indicates the mean intensity over time. B) Box plots depicting the distribution of Ribonuclease 4 intensities at baseline, month 1, and month 2. Only AMD patients with measurements at all visits are included. The median, interquartile range, and outliers are displayed for each time point. Abbreviations: FDR, false discovery rate; ns, non−significant; * p < 0.05; ** p < 0.01; *** p < 0.001.

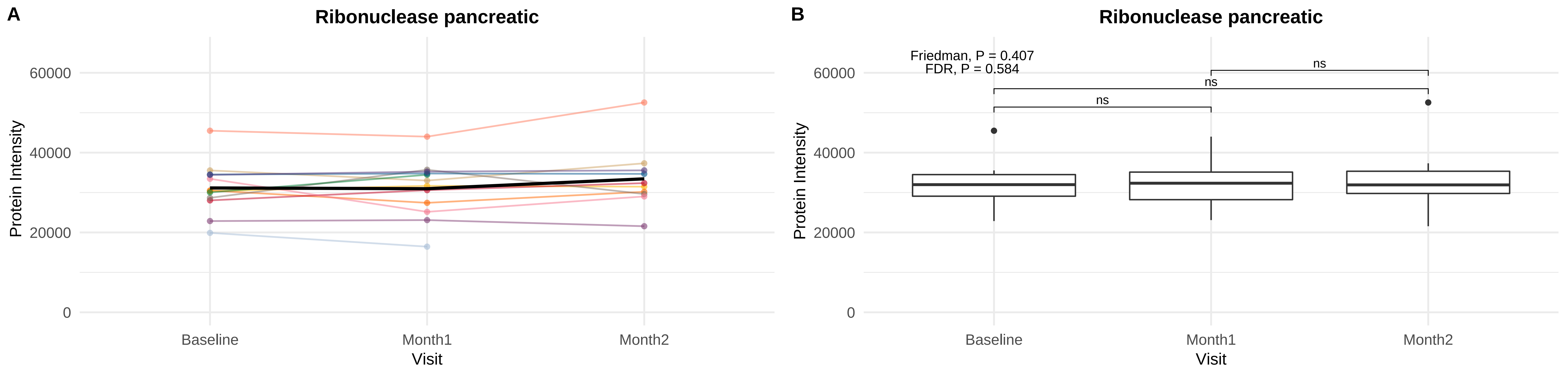

Supplementary Figure S 251

A) Line plot illustrating individual patient trajectories of Ribonuclease pancreatic intensity over time. The bold black line indicates the mean intensity over time. B) Box plots depicting the distribution of Ribonuclease pancreatic intensities at baseline, month 1, and month 2. Only AMD patients with measurements at all visits are included. The median, interquartile range, and outliers are displayed for each time point. Abbreviations: FDR, false discovery rate; ns, non−significant; * p < 0.05; ** p < 0.01; *** p < 0.001.

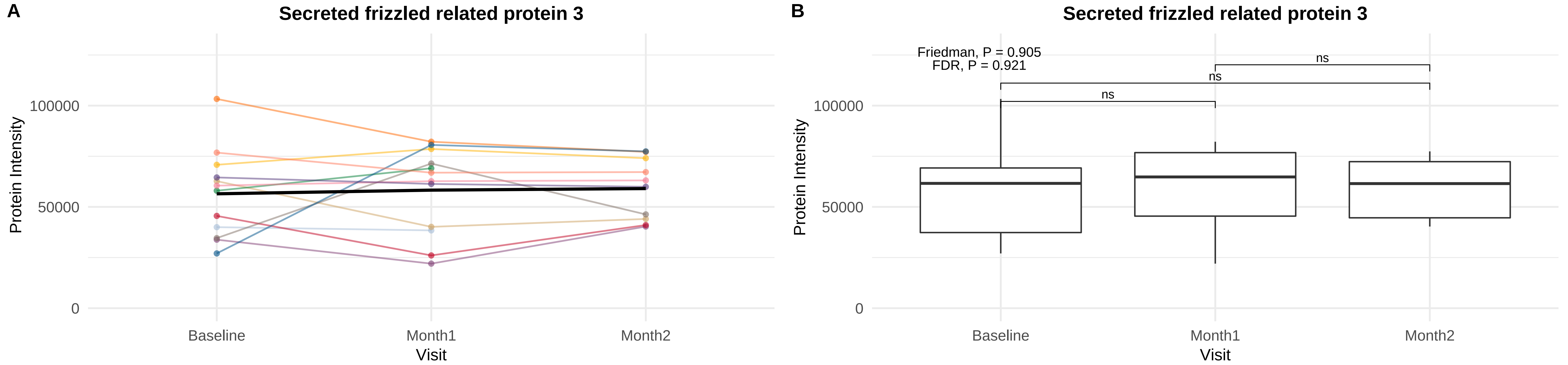

Supplementary Figure S 252

A) Line plot illustrating individual patient trajectories of Secreted frizzled related protein 3 intensity over time. The bold black line indicates the mean intensity over time. B) Box plots depicting the distribution of Secreted frizzled related protein 3 intensities at baseline, month 1, and month 2. Only AMD patients with measurements at all visits are included. The median, interquartile range, and outliers are displayed for each time point. Abbreviations: FDR, false discovery rate; ns, non−significant; * p < 0.05; ** p < 0.01; *** p < 0.001.

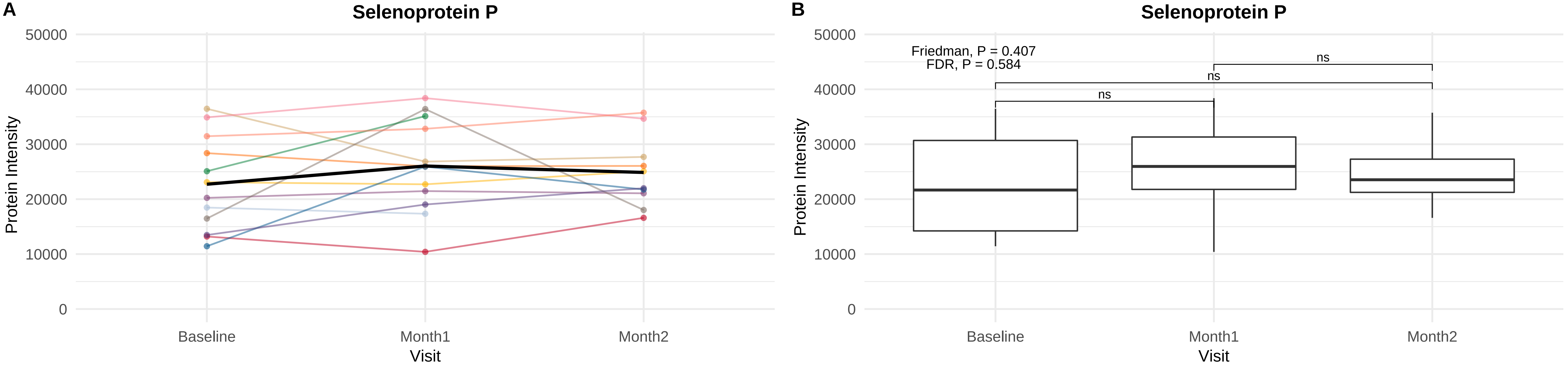

Supplementary Figure S 253

A) Line plot illustrating individual patient trajectories of Selenoprotein P intensity over time. The bold black line indicates the mean intensity over time. B) Box plots depicting the distribution of Selenoprotein P intensities at baseline, month 1, and month 2. Only AMD patients with measurements at all visits are included. The median, interquartile range, and outliers are displayed for each time point. Abbreviations: FDR, false discovery rate; ns, non−significant; * p < 0.05; ** p < 0.01; *** p < 0.001.

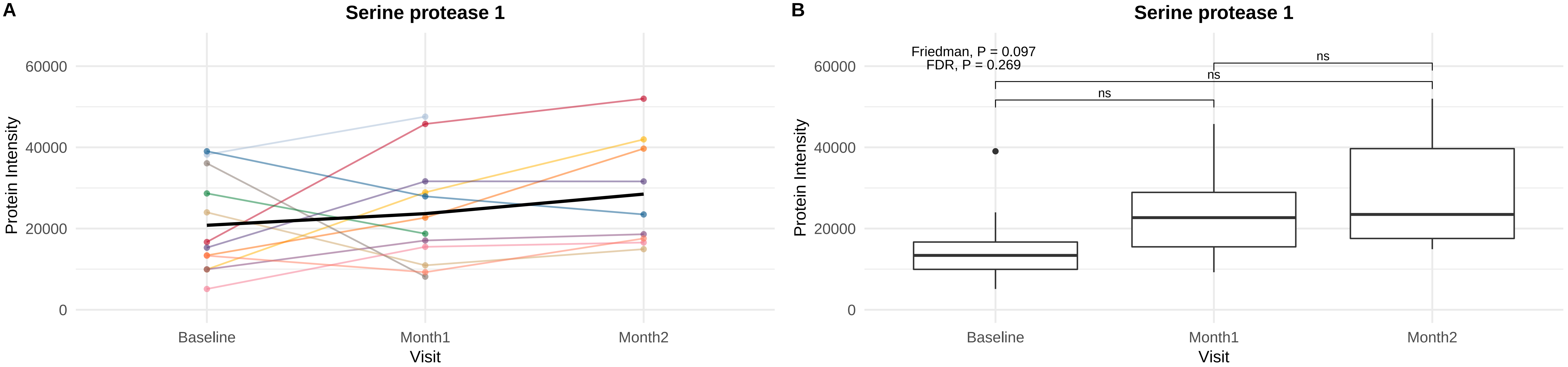

Supplementary Figure S 254

A) Line plot illustrating individual patient trajectories of Serine protease 1 intensity over time. The bold black line indicates the mean intensity over time. B) Box plots depicting the distribution of Serine protease 1 intensities at baseline, month 1, and month 2. Only AMD patients with measurements at all visits are included. The median, interquartile range, and outliers are displayed for each time point. Abbreviations: FDR, false discovery rate; ns, non−significant; * p < 0.05; ** p < 0.01; *** p < 0.001.

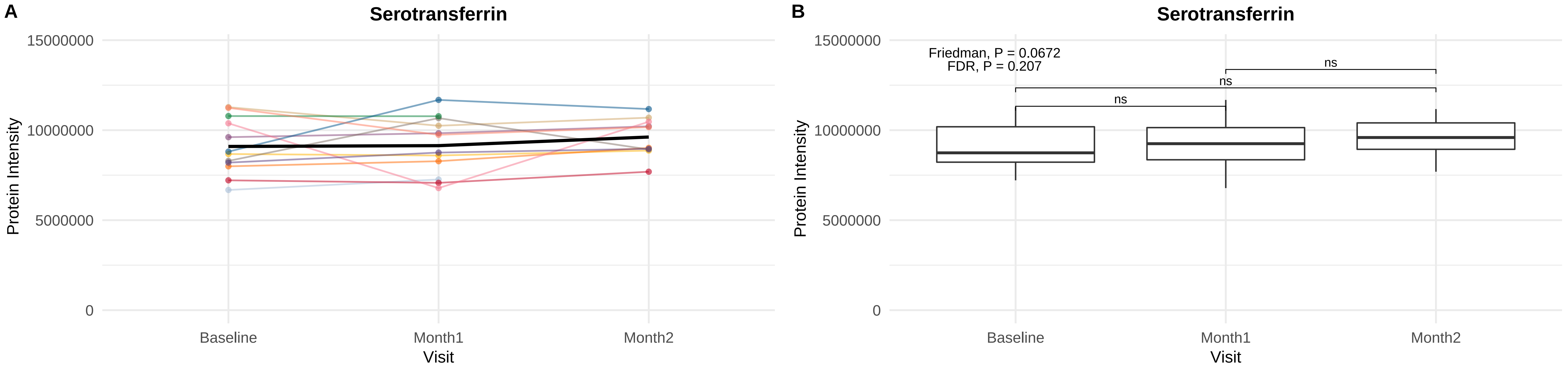

Supplementary Figure S 255

A) Line plot illustrating individual patient trajectories of Serotransferrin intensity over time. The bold black line indicates the mean intensity over time. B) Box plots depicting the distribution of Serotransferrin intensities at baseline, month 1, and month 2. Only AMD patients with measurements at all visits are included. The median, interquartile range, and outliers are displayed for each time point. Abbreviations: FDR, false discovery rate; ns, non−significant; * p < 0.05; ** p < 0.01; *** p < 0.001.

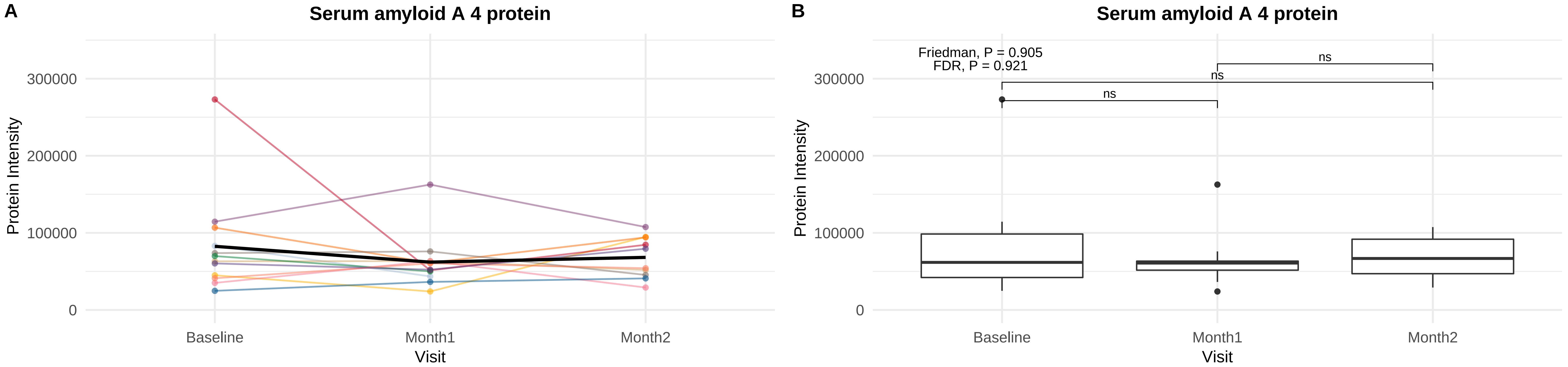

Supplementary Figure S 256

A) Line plot illustrating individual patient trajectories of Serum amyloid A 4 protein intensity over time. The bold black line indicates the mean intensity over time. B) Box plots depicting the distribution of Serum amyloid A 4 protein intensities at baseline, month 1, and month 2. Only AMD patients with measurements at all visits are included. The median, interquartile range, and outliers are displayed for each time point. Abbreviations: FDR, false discovery rate; ns, non−significant; * p < 0.05; ** p < 0.01; *** p < 0.001.

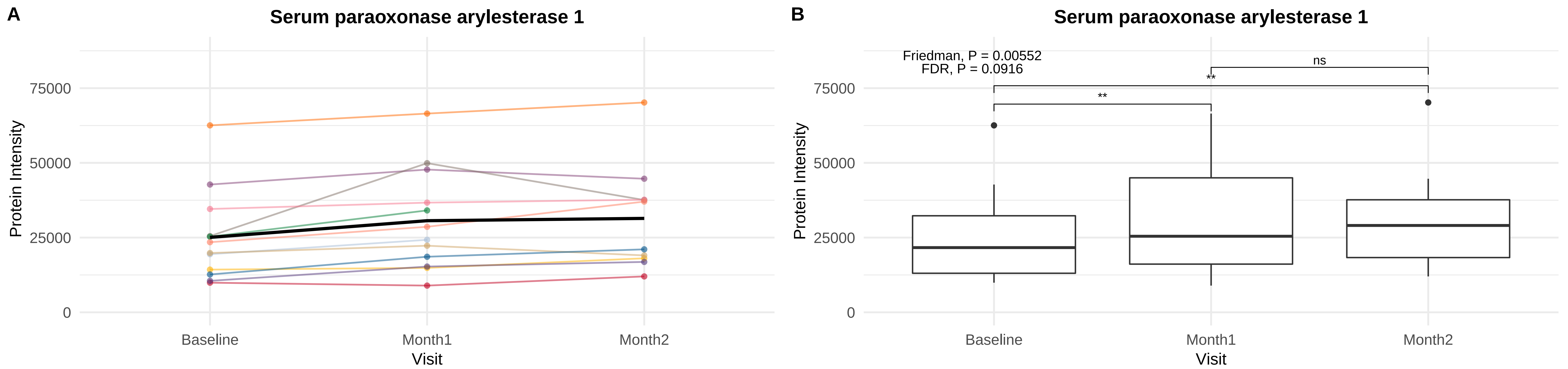

Supplementary Figure S 257

A) Line plot illustrating individual patient trajectories of Serum paraoxonase arylesterase 1 intensity over time. The bold black line indicates the mean intensity over time. B) Box plots depicting the distribution of Serum paraoxonase arylesterase 1 intensities at baseline, month 1, and month 2. Only AMD patients with measurements at all visits are included. The median, interquartile range, and outliers are displayed for each time point. Abbreviations: FDR, false discovery rate; ns, non−significant; * p < 0.05; ** p < 0.01; *** p < 0.001.

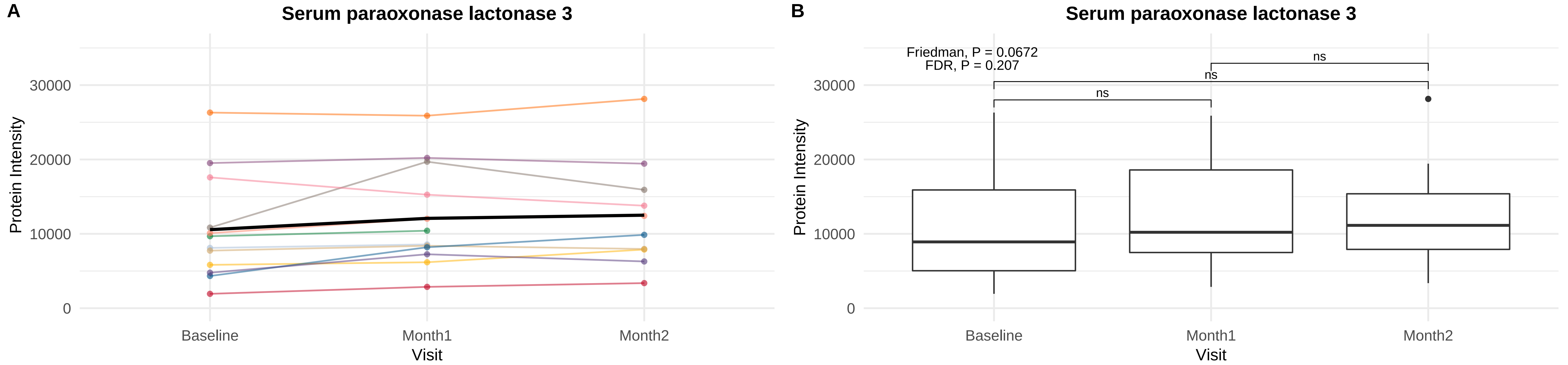

Supplementary Figure S 258

A) Line plot illustrating individual patient trajectories of Serum paraoxonase lactonase 3 intensity over time. The bold black line indicates the mean intensity over time. B) Box plots depicting the distribution of Serum paraoxonase lactonase 3 intensities at baseline, month 1, and month 2. Only AMD patients with measurements at all visits are included. The median, interquartile range, and outliers are displayed for each time point. Abbreviations: FDR, false discovery rate; ns, non−significant; * p < 0.05; ** p < 0.01; *** p < 0.001.

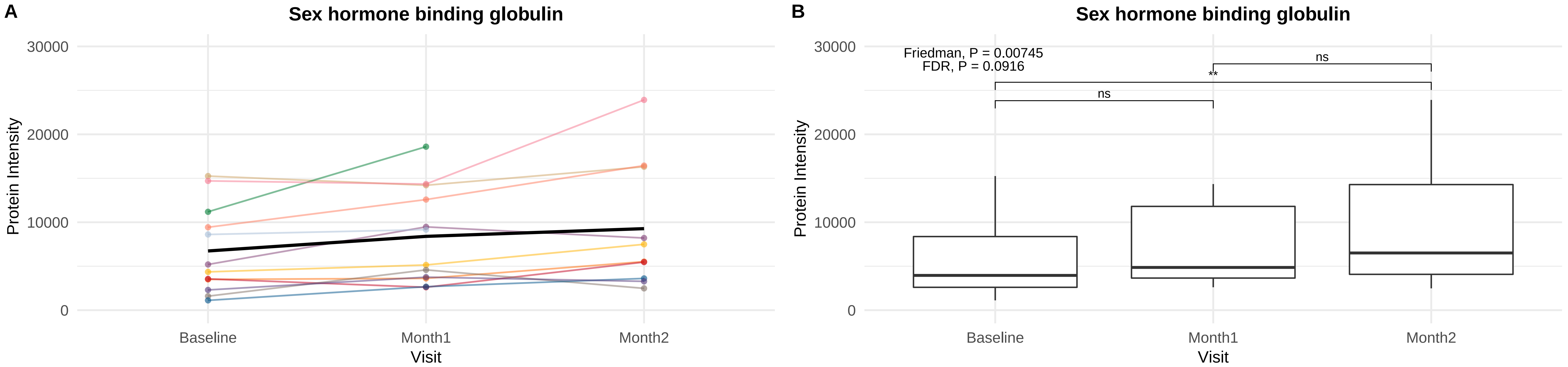

Supplementary Figure S 259

A) Line plot illustrating individual patient trajectories of Sex hormone binding globulin intensity over time. The bold black line indicates the mean intensity over time. B) Box plots depicting the distribution of Sex hormone binding globulin intensities at baseline, month 1, and month 2. Only AMD patients with measurements at all visits are included. The median, interquartile range, and outliers are displayed for each time point. Abbreviations: FDR, false discovery rate; ns, non−significant; * p < 0.05; ** p < 0.01; *** p < 0.001.

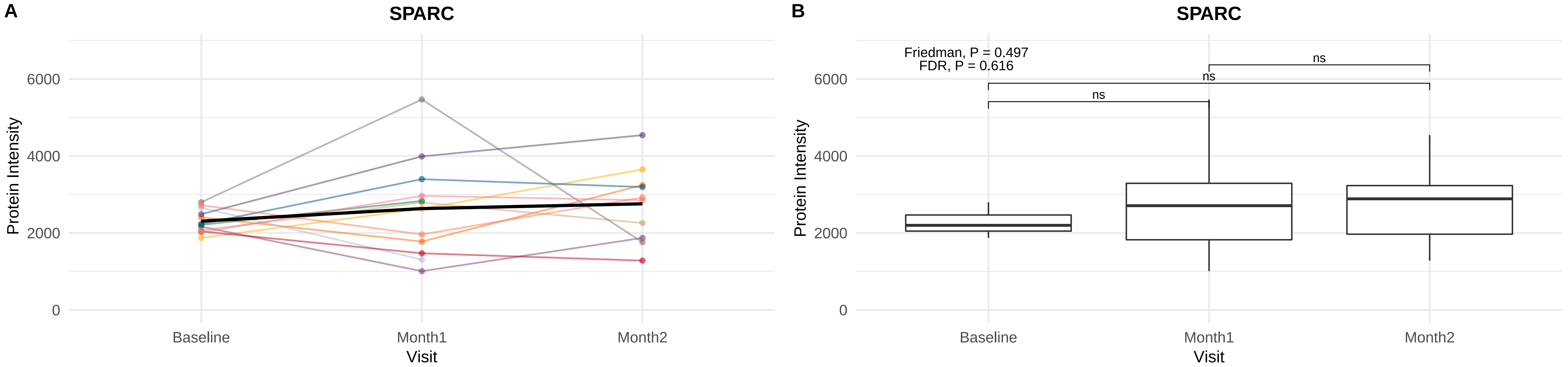

Supplementary Figure S 260

A) Line plot illustrating individual patient trajectories of SPARC intensity over time. The bold black line indicates the mean intensity over time. B) Box plots depicting the distribution of SPARC intensities at baseline, month 1, and month 2. Only AMD patients with measurements at all visits are included. The median, interquartile range, and outliers are displayed for each time point. Abbreviations: FDR, false discovery rate; ns, non−significant; * p < 0.05; ** p < 0.01; *** p < 0.001.

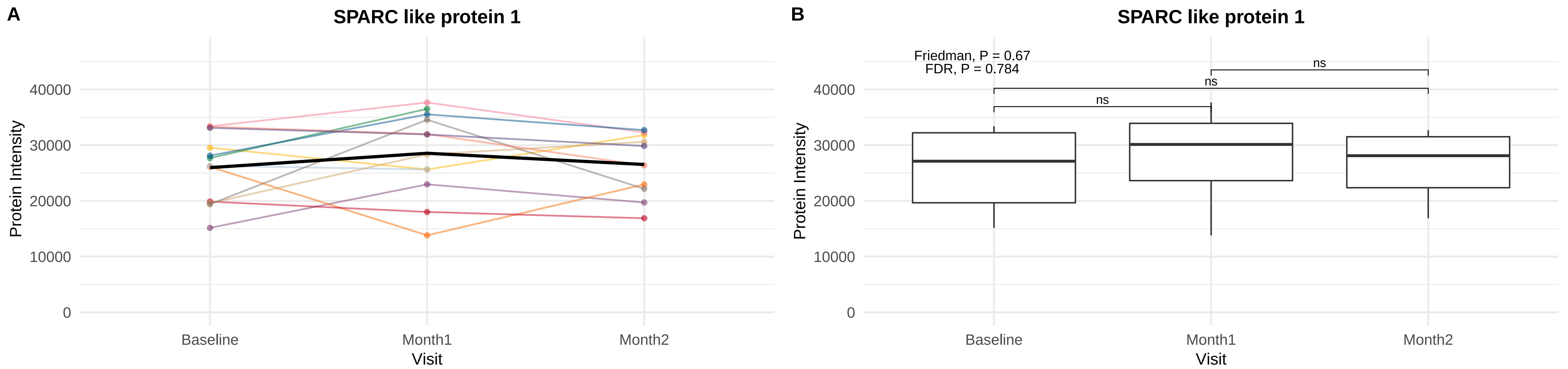

Supplementary Figure S 261

A) Line plot illustrating individual patient trajectories of SPARC like protein 1 intensity over time. The bold black line indicates the mean intensity over time. B) Box plots depicting the distribution of SPARC like protein 1 intensities at baseline, month 1, and month 2. Only AMD patients with measurements at all visits are included. The median, interquartile range, and outliers are displayed for each time point. Abbreviations: FDR, false discovery rate; ns, non−significant; * p < 0.05; ** p < 0.01; *** p < 0.001.

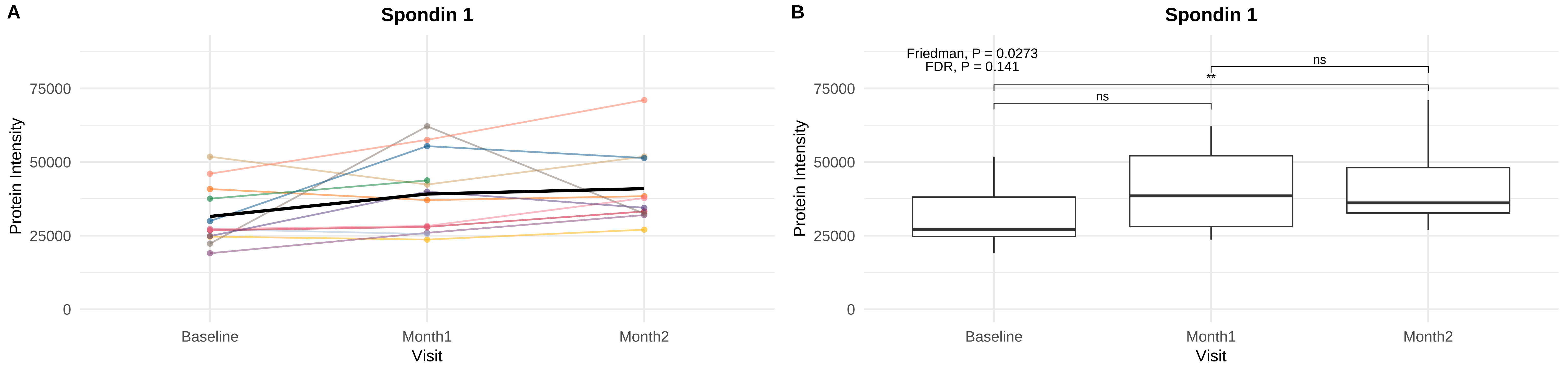

Supplementary Figure S 262

A) Line plot illustrating individual patient trajectories of Spondin 1 intensity over time. The bold black line indicates the mean intensity over time. B) Box plots depicting the distribution of Spondin 1 intensities at baseline, month 1, and month 2. Only AMD patients with measurements at all visits are included. The median, interquartile range, and outliers are displayed for each time point. Abbreviations: FDR, false discovery rate; ns, non−significant; * p < 0.05; ** p < 0.01; *** p < 0.001.

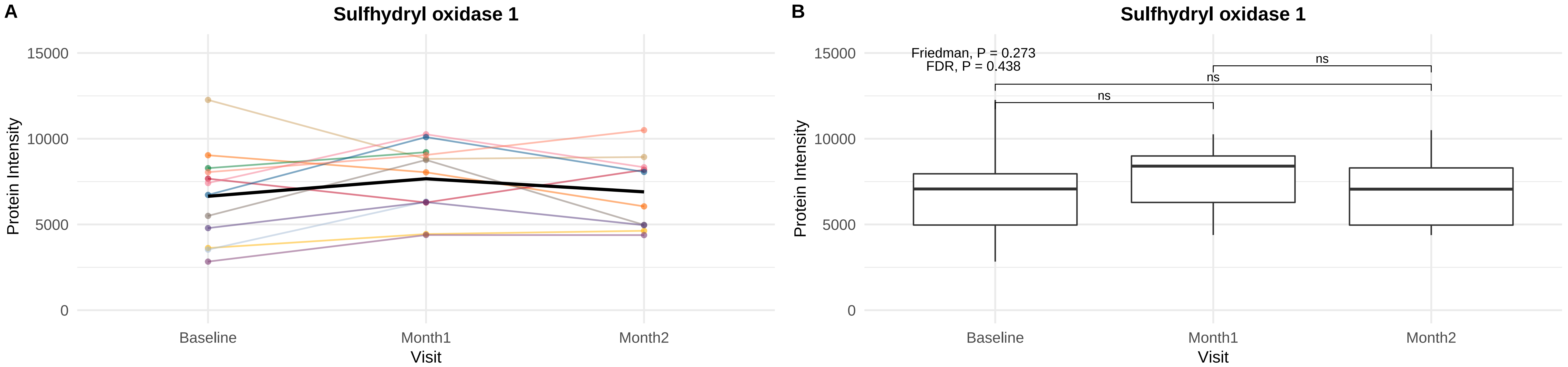

Supplementary Figure S 263

A) Line plot illustrating individual patient trajectories of Sulfhydryl oxidase 1 intensity over time. The bold black line indicates the mean intensity over time. B) Box plots depicting the distribution of Sulfhydryl oxidase 1 intensities at baseline, month 1, and month 2. Only AMD patients with measurements at all visits are included. The median, interquartile range, and outliers are displayed for each time point. Abbreviations: FDR, false discovery rate; ns, non−significant; * p < 0.05; ** p < 0.01; *** p < 0.001.

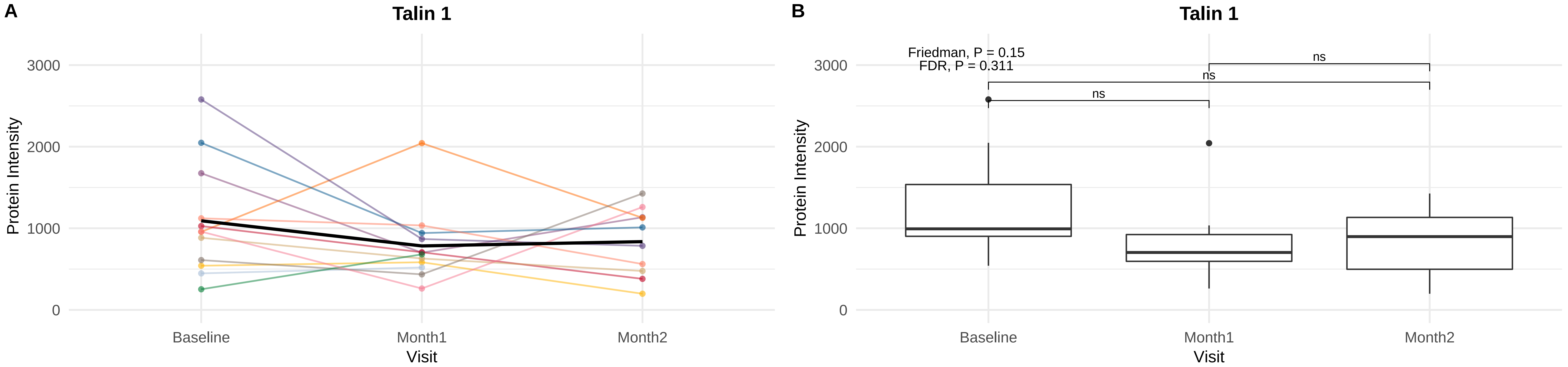

Supplementary Figure S 264

A) Line plot illustrating individual patient trajectories of Talin 1 intensity over time. The bold black line indicates the mean intensity over time. B) Box plots depicting the distribution of Talin 1 intensities at baseline, month 1, and month 2. Only AMD patients with measurements at all visits are included. The median, interquartile range, and outliers are displayed for each time point. Abbreviations: FDR, false discovery rate; ns, non−significant; * p < 0.05; ** p < 0.01; *** p < 0.001.

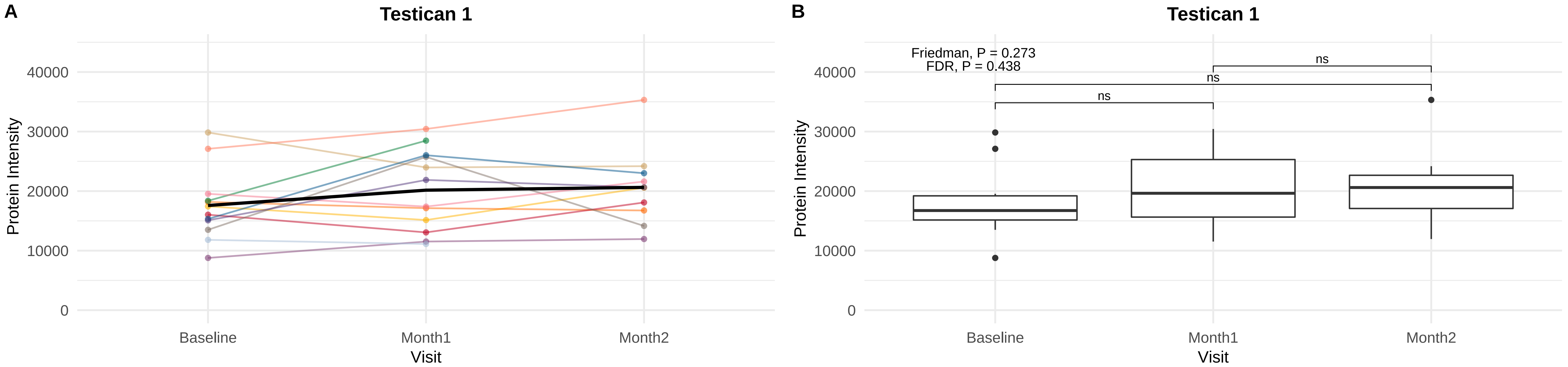

Supplementary Figure S 265

A) Line plot illustrating individual patient trajectories of Testican 1 intensity over time. The bold black line indicates the mean intensity over time. B) Box plots depicting the distribution of Testican 1 intensities at baseline, month 1, and month 2. Only AMD patients with measurements at all visits are included. The median, interquartile range, and outliers are displayed for each time point. Abbreviations: FDR, false discovery rate; ns, non−significant; * p < 0.05; ** p < 0.01; *** p < 0.001.

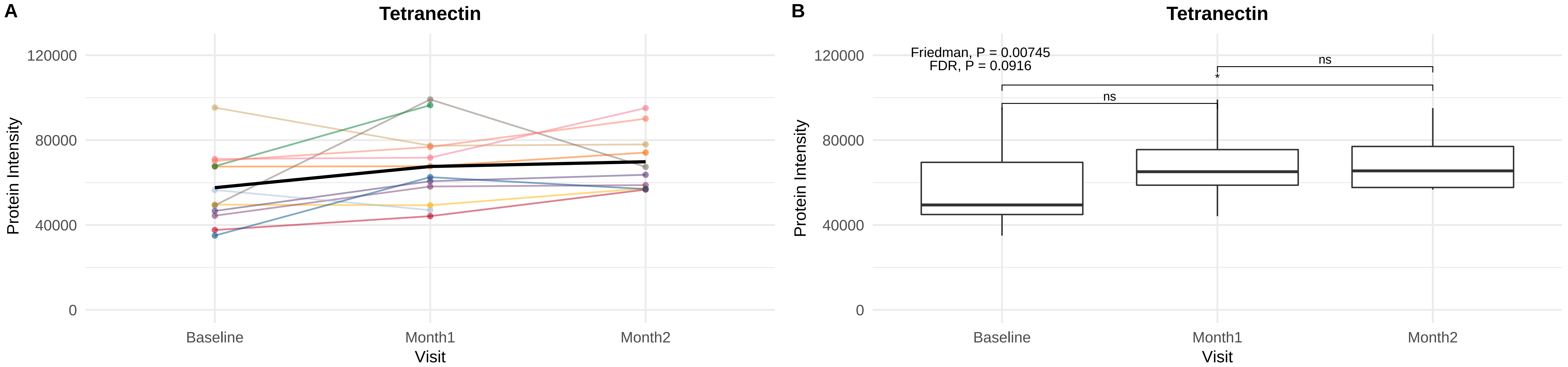

Supplementary Figure S 266

A) Line plot illustrating individual patient trajectories of Tetranectin intensity over time. The bold black line indicates the mean intensity over time. B) Box plots depicting the distribution of Tetranectin intensities at baseline, month 1, and month 2. Only AMD patients with measurements at all visits are included. The median, interquartile range, and outliers are displayed for each time point. Abbreviations: FDR, false discovery rate; ns, non−significant; * p < 0.05; ** p < 0.01; *** p < 0.001.

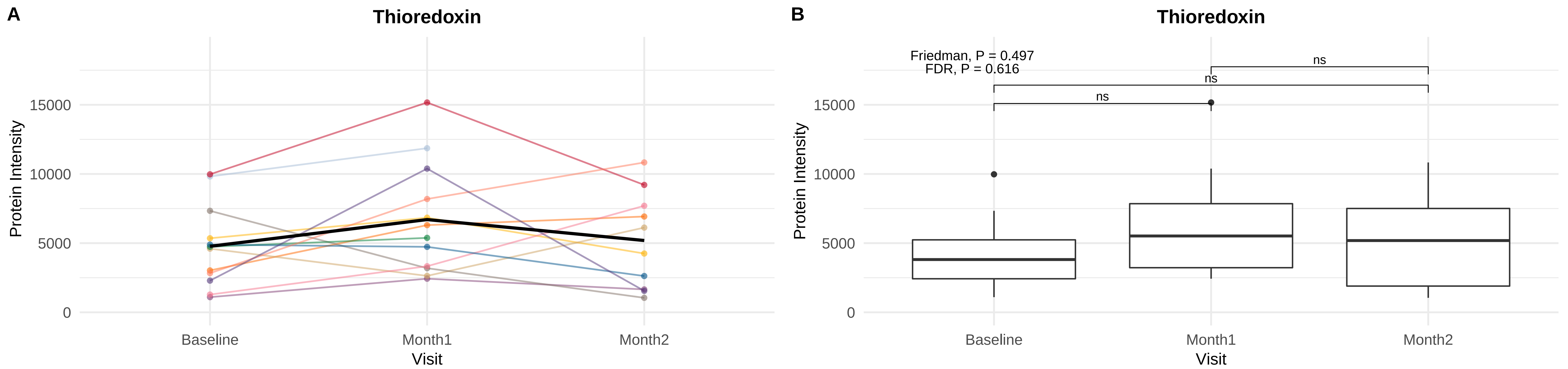

Supplementary Figure S 267

A) Line plot illustrating individual patient trajectories of Thioredoxin intensity over time. The bold black line indicates the mean intensity over time. B) Box plots depicting the distribution of Thioredoxin intensities at baseline, month 1, and month 2. Only AMD patients with measurements at all visits are included. The median, interquartile range, and outliers are displayed for each time point. Abbreviations: FDR, false discovery rate; ns, non−significant; * p < 0.05; ** p < 0.01; *** p < 0.001.

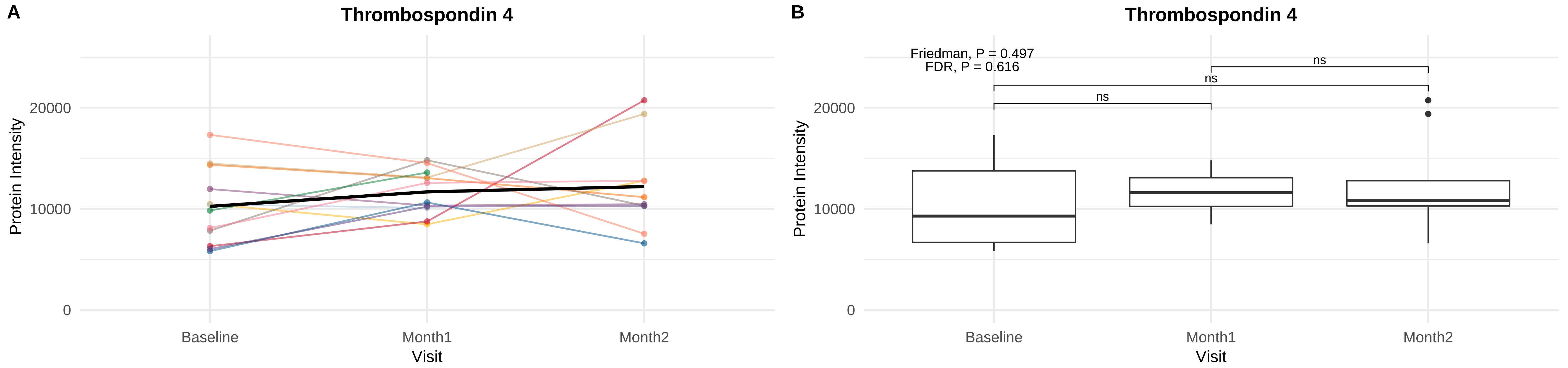

Supplementary Figure S 268

A) Line plot illustrating individual patient trajectories of Thrombospondin 4 intensity over time. The bold black line indicates the mean intensity over time. B) Box plots depicting the distribution of Thrombospondin 4 intensities at baseline, month 1, and month 2. Only AMD patients with measurements at all visits are included. The median, interquartile range, and outliers are displayed for each time point. Abbreviations: FDR, false discovery rate; ns, non−significant; * p < 0.05; ** p < 0.01; *** p < 0.001.

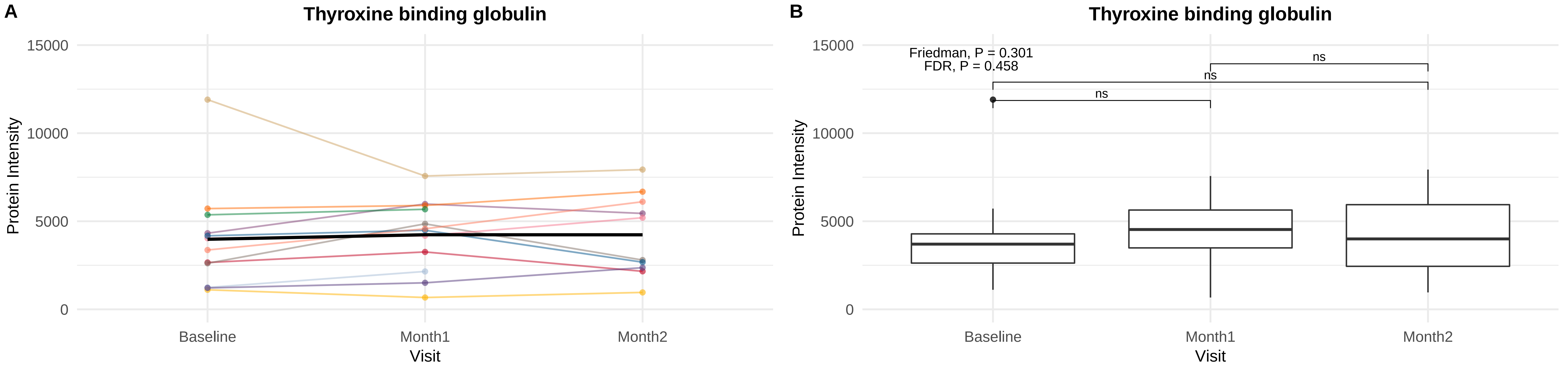

Supplementary Figure S 269

A) Line plot illustrating individual patient trajectories of Thyroxine binding globulin intensity over time. The bold black line indicates the mean intensity over time. B) Box plots depicting the distribution of Thyroxine binding globulin intensities at baseline, month 1, and month 2. Only AMD patients with measurements at all visits are included. The median, interquartile range, and outliers are displayed for each time point. Abbreviations: FDR, false discovery rate; ns, non−significant; * p < 0.05; ** p < 0.01; *** p < 0.001.

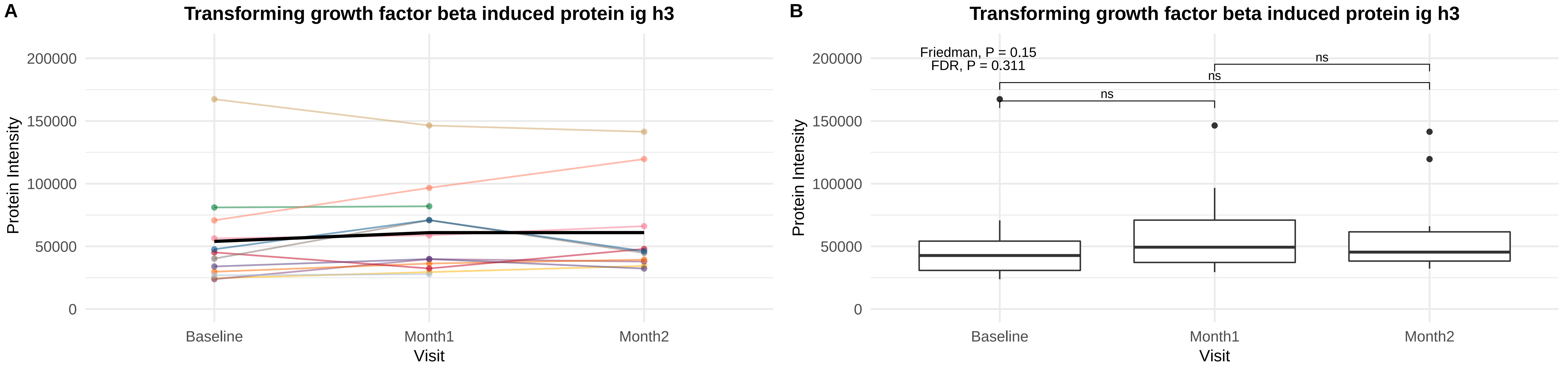

Supplementary Figure S 270

A) Line plot illustrating individual patient trajectories of Transforming growth factor beta induced protein ig h3 intensity over time. The bold black line indicates the mean intensity over time. B) Box plots depicting the distribution of Transforming growth factor beta induced protein ig h3 intensities at baseline, month 1, and month 2. Only AMD patients with measurements at all visits are included. The median, interquartile range, and outliers are displayed for each time point. Abbreviations: FDR, false discovery rate; ns, non−significant; * p < 0.05; ** p < 0.01; *** p < 0.001

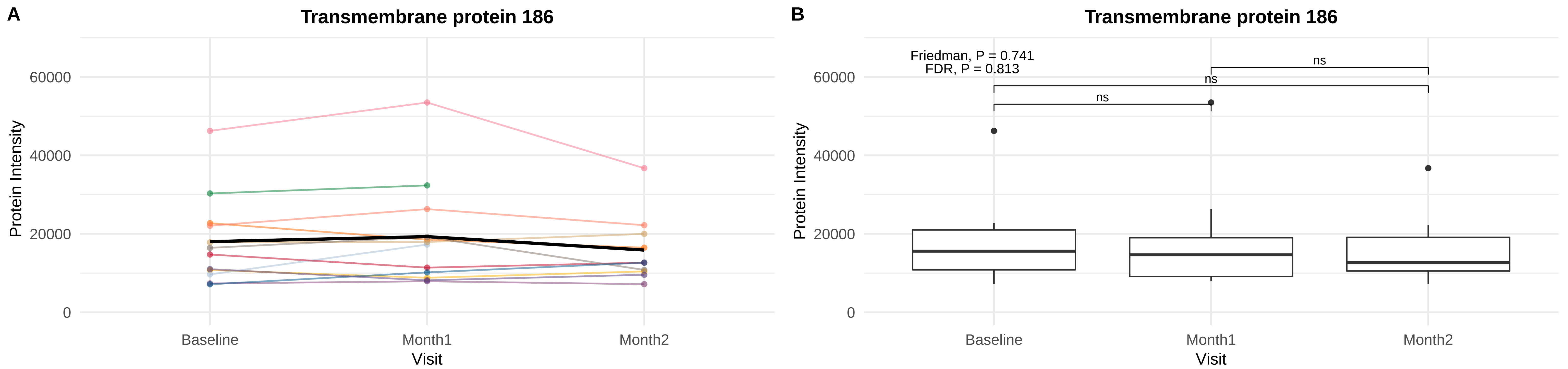

Supplementary Figure S 271

A) Line plot illustrating individual patient trajectories of Transmembrane protein 186 intensity over time. The bold black line indicates the mean intensity over time. B) Box plots depicting the distribution of Transmembrane protein 186 intensities at baseline, month 1, and month 2. Only AMD patients with measurements at all visits are included. The median, interquartile range, and outliers are displayed for each time point. Abbreviations: FDR, false discovery rate; ns, non−significant; * p < 0.05; ** p < 0.01; *** p < 0.001.

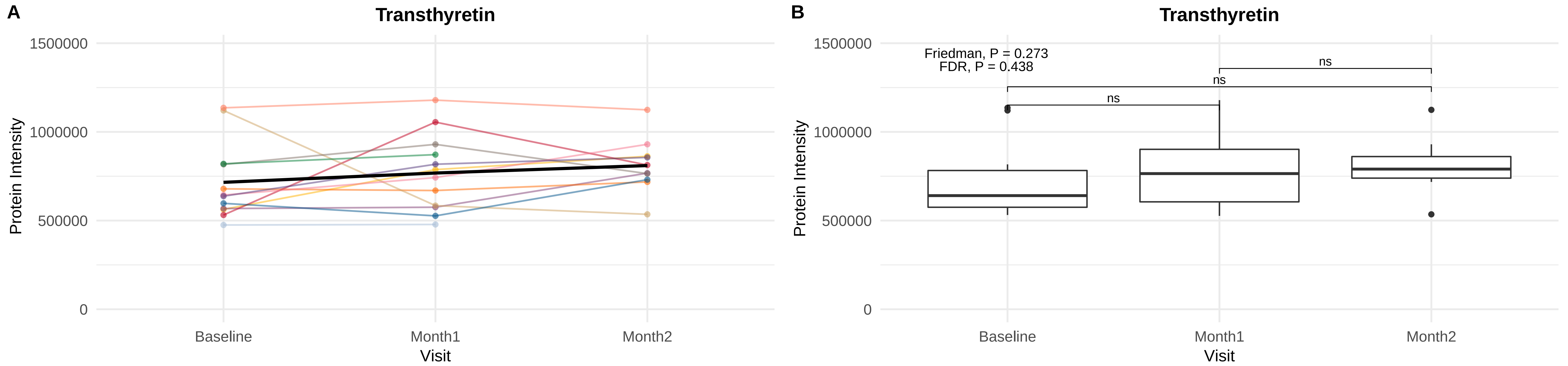

Supplementary Figure S 272

A) Line plot illustrating individual patient trajectories of Transthyretin intensity over time. The bold black line indicates the mean intensity over time. B) Box plots depicting the distribution of Transthyretin intensities at baseline, month 1, and month 2. Only AMD patients with measurements at all visits are included. The median, interquartile range, and outliers are displayed for each time point. Abbreviations: FDR, false discovery rate; ns, non−significant; * p < 0.05; ** p < 0.01; *** p < 0.001.

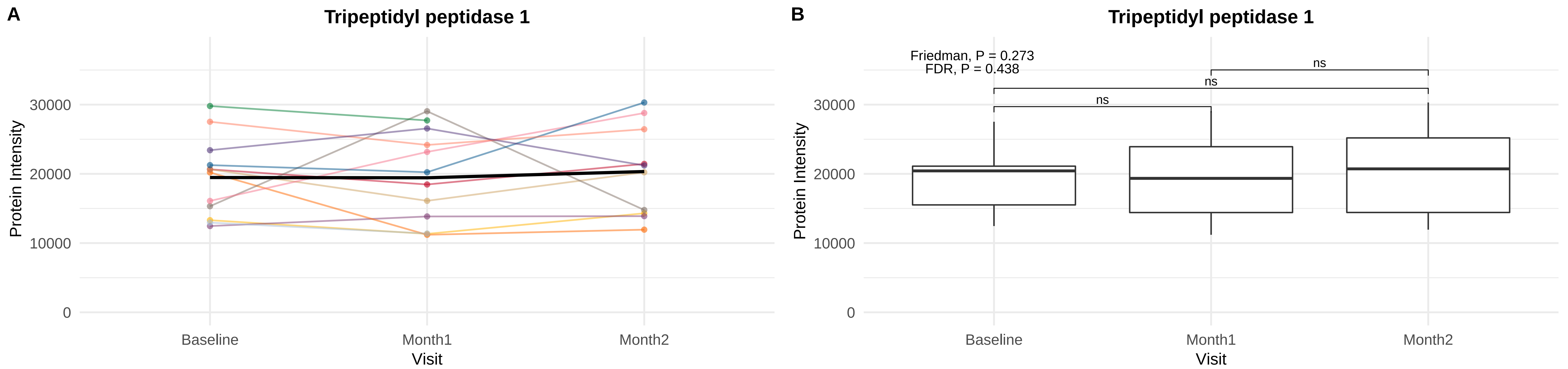

Supplementary Figure S 273

A) Line plot illustrating individual patient trajectories of Tripeptidyl peptidase 1 intensity over time. The bold black line indicates the mean intensity over time. B) Box plots depicting the distribution of Tripeptidyl peptidase 1 intensities at baseline, month 1, and month 2. Only AMD patients with measurements at all visits are included. The median, interquartile range, and outliers are displayed for each time point. Abbreviations: FDR, false discovery rate; ns, non−significant; * p < 0.05; ** p < 0.01; *** p < 0.001.

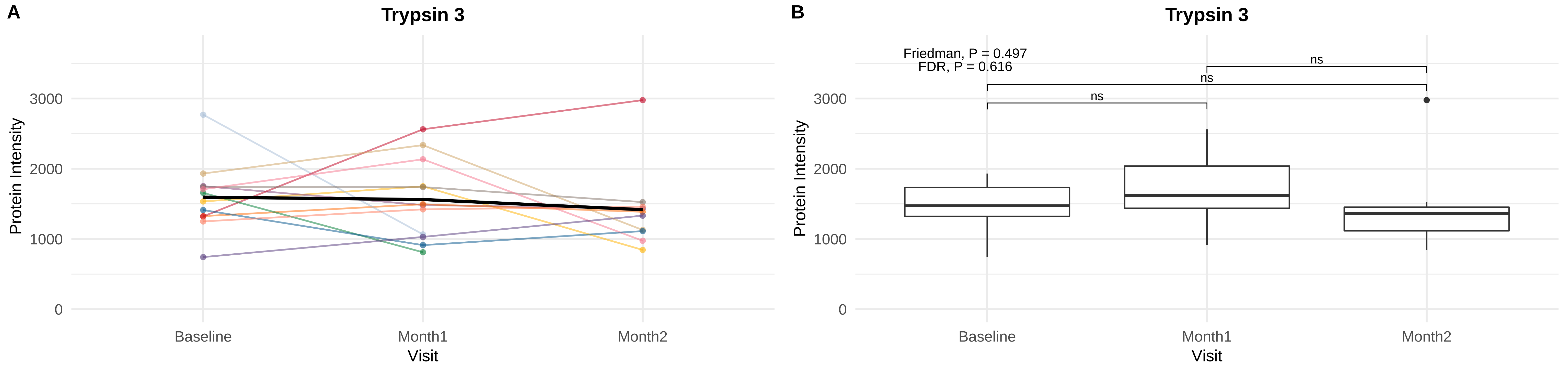

Supplementary Figure S 274

A) Line plot illustrating individual patient trajectories of Trypsin 3 intensity over time. The bold black line indicates the mean intensity over time. B) Box plots depicting the distribution of Trypsin 3 intensities at baseline, month 1, and month 2. Only AMD patients with measurements at all visits are included. The median, interquartile range, and outliers are displayed for each time point. Abbreviations: FDR, false discovery rate; ns, non−significant; * p < 0.05; ** p < 0.01; *** p < 0.001.

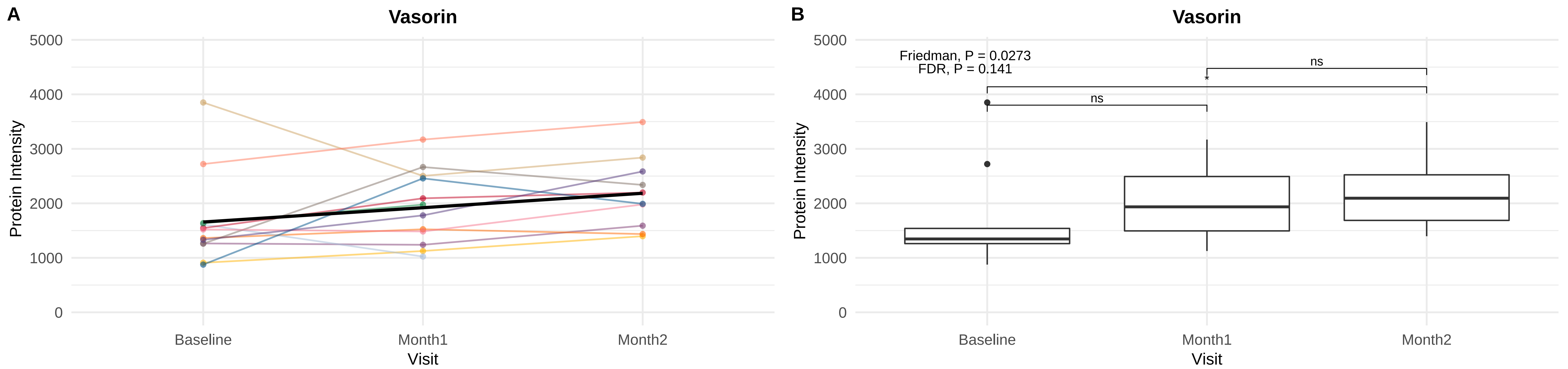

Supplementary Figure S 275

A) Line plot illustrating individual patient trajectories of Vasorin intensity over time. The bold black line indicates the mean intensity over time. B) Box plots depicting the distribution of Vasorin intensities at baseline, month 1, and month 2. Only AMD patients with measurements at all visits are included. The median, interquartile range, and outliers are displayed for each time point. Abbreviations: FDR, false discovery rate; ns, non−significant; * p < 0.05; ** p < 0.01; *** p < 0.001.

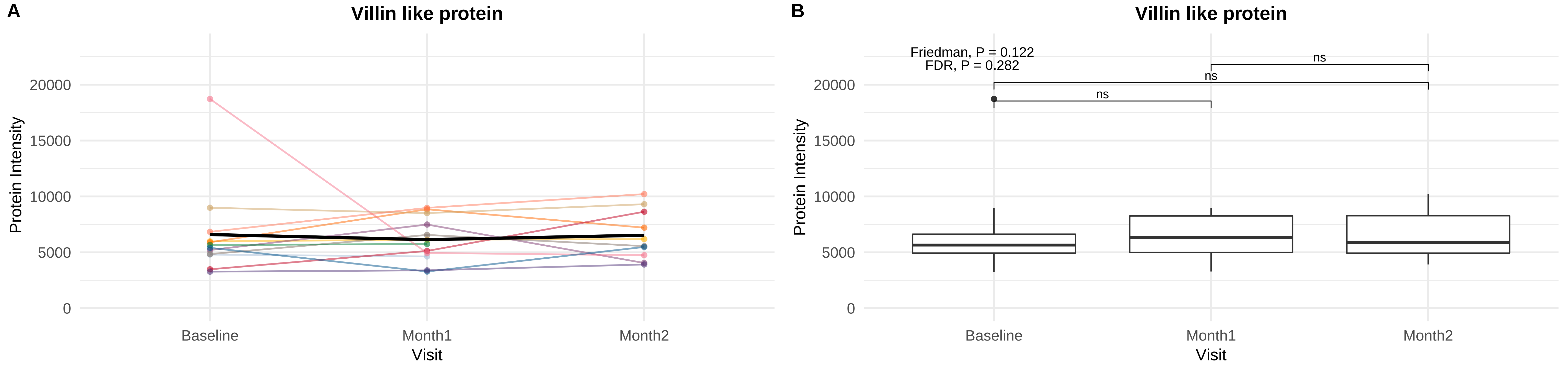

Supplementary Figure S 276

A) Line plot illustrating individual patient trajectories of Villin like protein intensity over time. The bold black line indicates the mean intensity over time. B) Box plots depicting the distribution of Villin like protein intensities at baseline, month 1, and month 2. Only AMD patients with measurements at all visits are included. The median, interquartile range, and outliers are displayed for each time point. Abbreviations: FDR, false discovery rate; ns, non−significant; * p < 0.05; ** p < 0.01; *** p < 0.001.

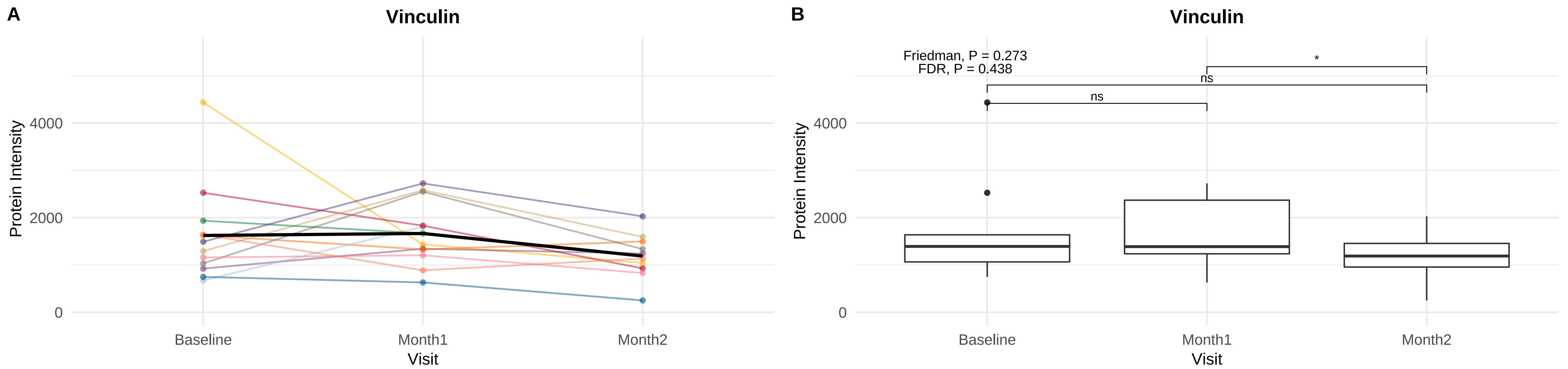

Supplementary Figure S 277

A) Line plot illustrating individual patient trajectories of Vinculin intensity over time. The bold black line indicates the mean intensity over time. B) Box plots depicting the distribution of Vinculin intensities at baseline, month 1, and month 2. Only AMD patients with measurements at all visits are included. The median, interquartile range, and outliers are displayed for each time point. Abbreviations: FDR, false discovery rate; ns, non−significant; * p < 0.05; ** p < 0.01; *** p < 0.001.

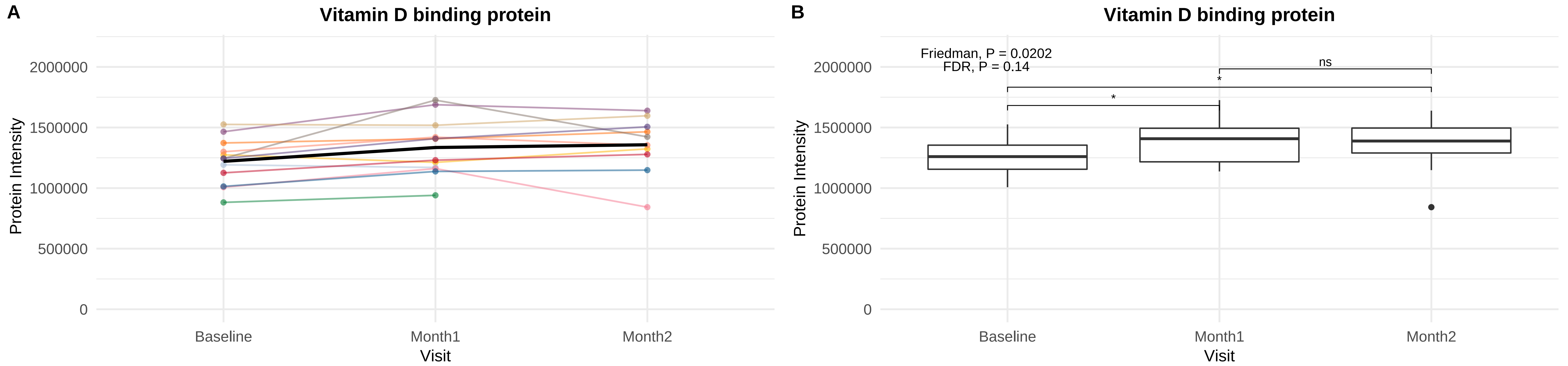

Supplementary Figure S 278

A) Line plot illustrating individual patient trajectories of Vitamin D binding protein intensity over time. The bold black line indicates the mean intensity over time. B) Box plots depicting the distribution of Vitamin D binding protein intensities at baseline, month 1, and month 2. Only AMD patients with measurements at all visits are included. The median, interquartile range, and outliers are displayed for each time point. Abbreviations: FDR, false discovery rate; ns, non−significant; * p < 0.05; ** p < 0.01; *** p < 0.001.

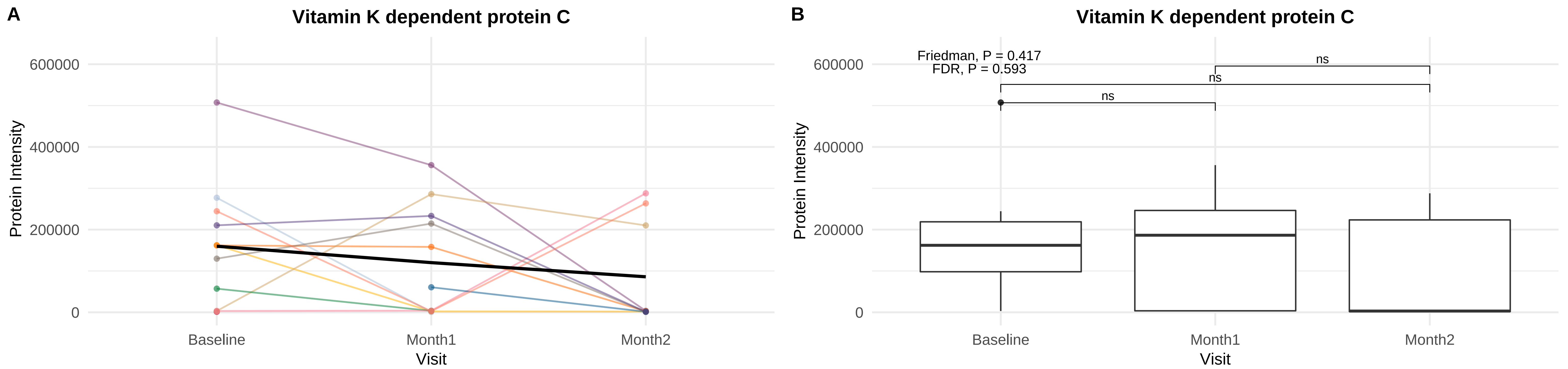

Supplementary Figure S 279

A) Line plot illustrating individual patient trajectories of Vitamin K dependent protein C intensity over time. The bold black line indicates the mean intensity over time. B) Box plots depicting the distribution of Vitamin K dependent protein C intensities at baseline, month 1, and month 2. Only AMD patients with measurements at all visits are included. The median, interquartile range, and outliers are displayed for each time point. Abbreviations: FDR, false discovery rate; ns, non−significant; * p < 0.05; ** p < 0.01; *** p < 0.001.

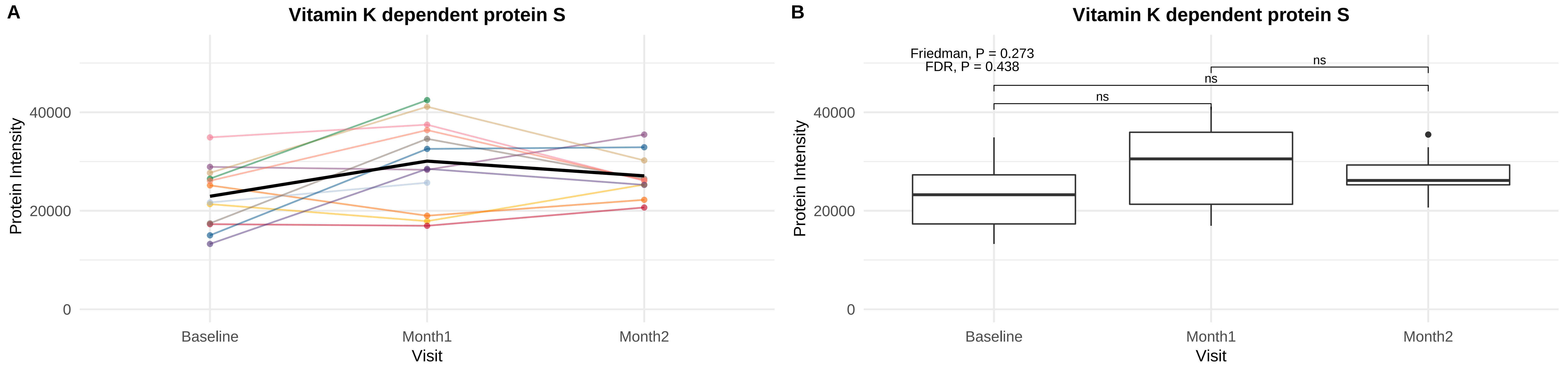

Supplementary Figure S 280

A) Line plot illustrating individual patient trajectories of Vitamin K dependent protein S intensity over time. The bold black line indicates the mean intensity over time. B) Box plots depicting the distribution of Vitamin K dependent protein S intensities at baseline, month 1, and month 2. Only AMD patients with measurements at all visits are included. The median, interquartile range, and outliers are displayed for each time point. Abbreviations: FDR, false discovery rate; ns, non−significant; * p < 0.05; ** p < 0.01; *** p < 0.001.

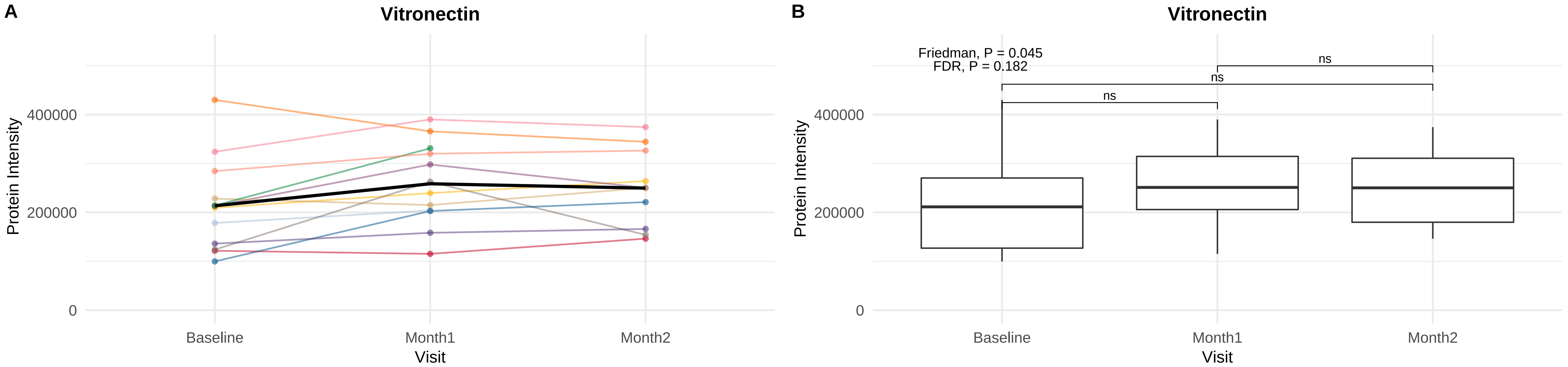

Supplementary Figure S 281

A) Line plot illustrating individual patient trajectories of Vitronectin intensity over time. The bold black line indicates the mean intensity over time. B) Box plots depicting the distribution of Vitronectin intensities at baseline, month 1, and month 2. Only AMD patients with measurements at all visits are included. The median, interquartile range, and outliers are displayed for each time point. Abbreviations: FDR, false discovery rate; ns, non−significant; * p < 0.05; ** p < 0.01; *** p < 0.001.

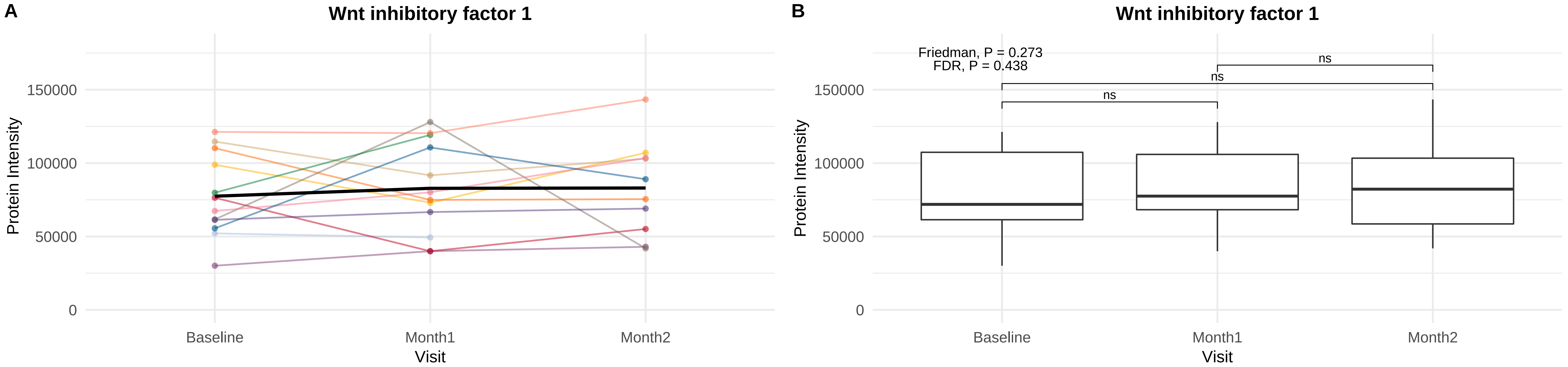

Supplementary Figure S 282

A) Line plot illustrating individual patient trajectories of Wnt inhibitory factor 1 intensity over time. The bold black line indicates the mean intensity over time. B) Box plots depicting the distribution of Wnt inhibitory factor 1 intensities at baseline, month 1, and month 2. Only AMD patients with measurements at all visits are included. The median, interquartile range, and outliers are displayed for each time point. Abbreviations: FDR, false discovery rate; ns, non−significant; * p < 0.05; ** p < 0.01; *** p < 0.001.

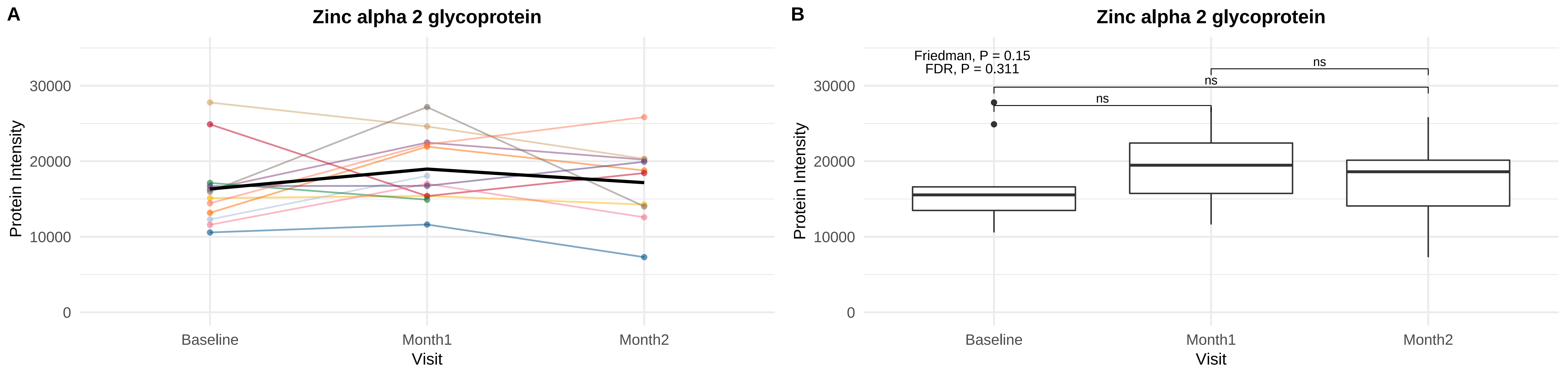

Supplementary Figure S 283

A) Line plot illustrating individual patient trajectories of Zinc alpha 2 glycoprotein intensity over time. The bold black line indicates the mean intensity over time. B) Box plots depicting the distribution of Zinc alpha 2 glycoprotein intensities at baseline, month 1, and month 2. Only AMD patients with measurements at all visits are included. The median, interquartile range, and outliers are displayed for each time point. Abbreviations: FDR, false discovery rate; ns, non−significant; * p < 0.05; ** p < 0.01; *** p < 0.001.

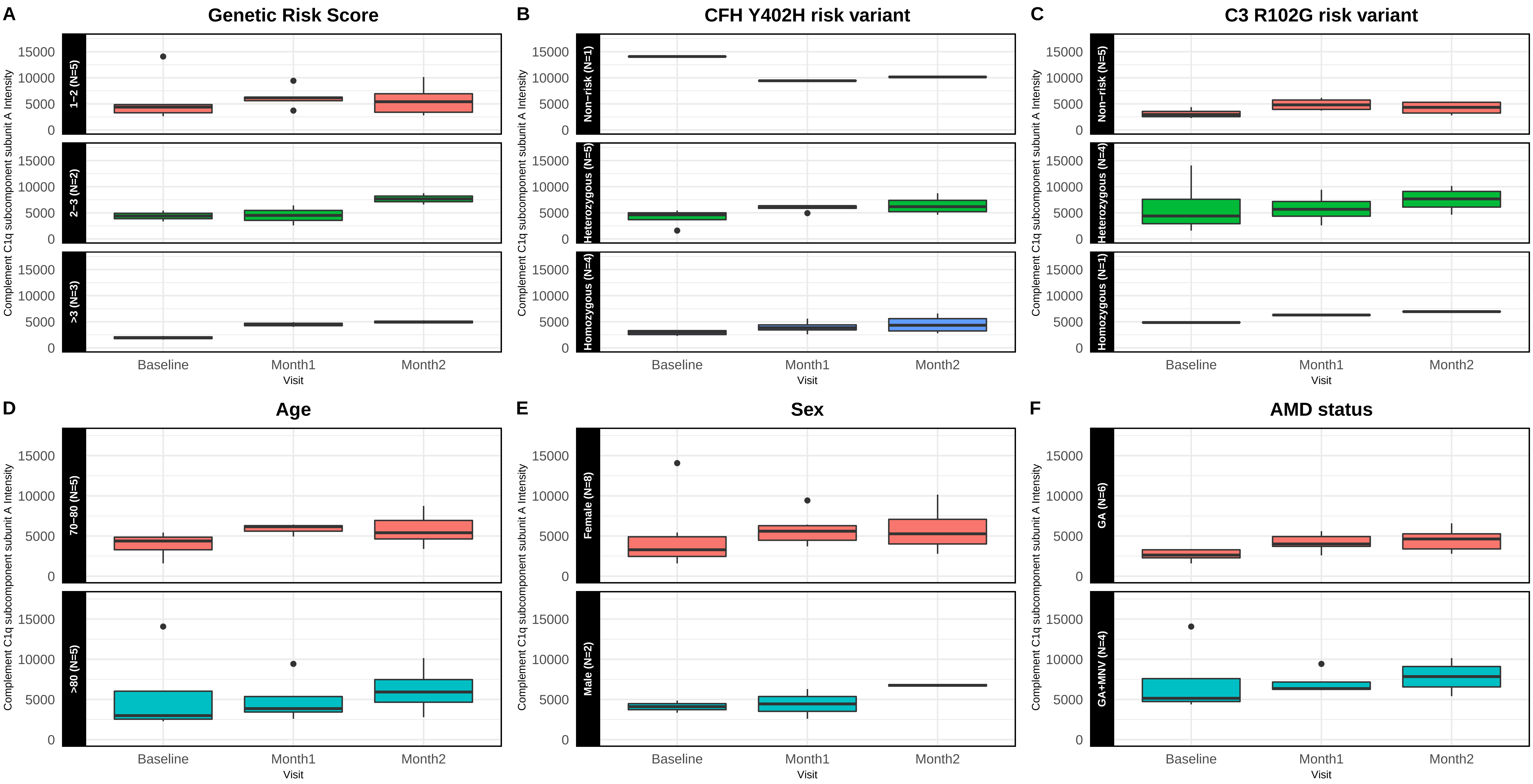

Supplementary Figure S284

Box plots depicting the distribution of Complement C1q subcomponent subunit A intensity at baseline, month 1, and month 2. Only AMD patients with measurements at all visits are included. The median, interquartile range, and outliers are displayed for each time point. Stratified on A) GRS. B) CFH Y402H risk variant. C) C3 R102G risk variant. D) Age. E) Sex. F) AMD status.

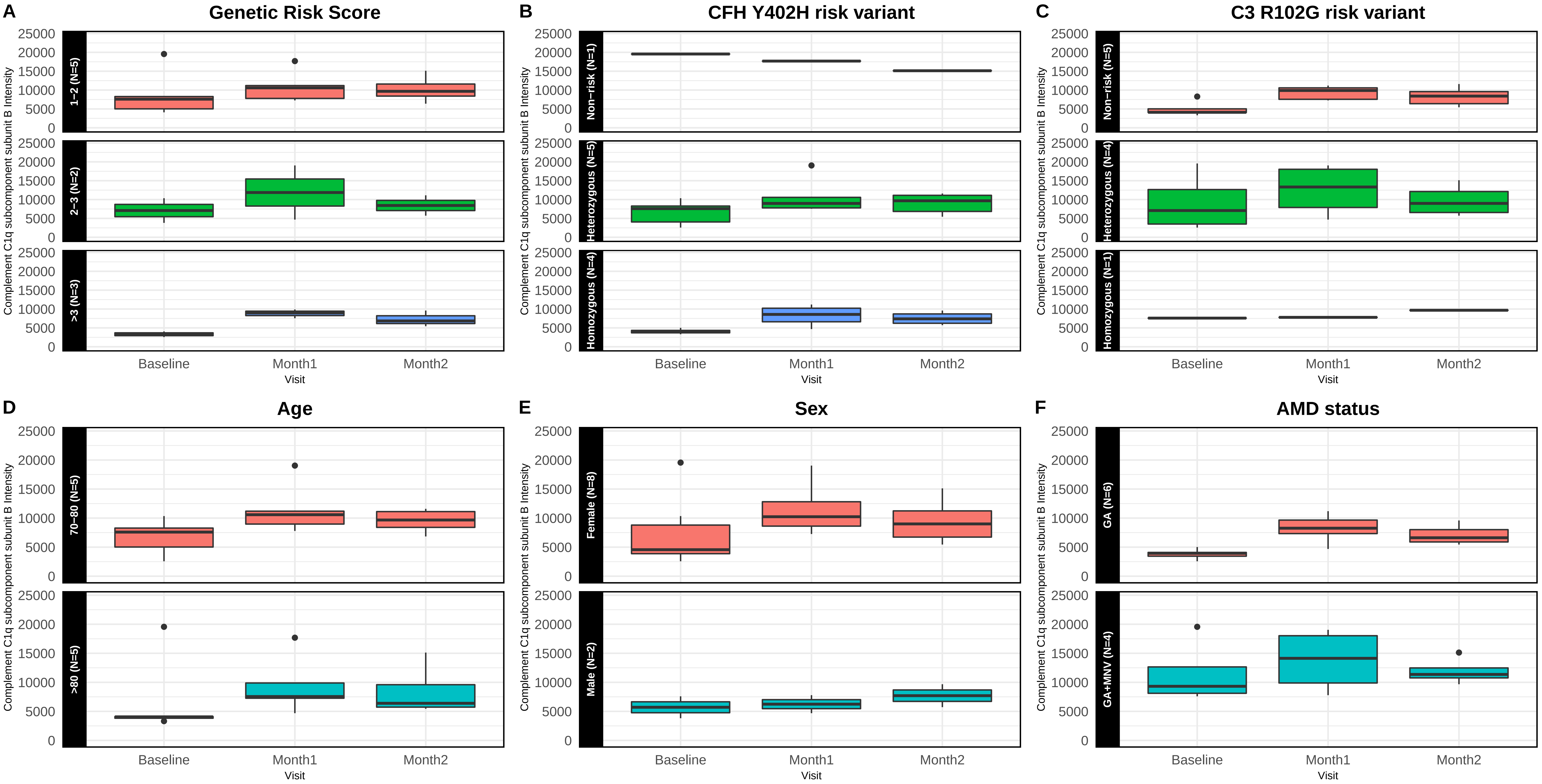

Supplementary Figure S285

Box plots depicting the distribution of Complement C1q subcomponent subunit B intensity at baseline, month 1, and month 2. Only AMD patients with measurements at all visits are included. The median, interquartile range, and outliers are displayed for each time point. Stratified on A) GRS. B) CFH Y402H risk variant. C) C3 R102G risk variant. D) Age. E) Sex. F) AMD status.

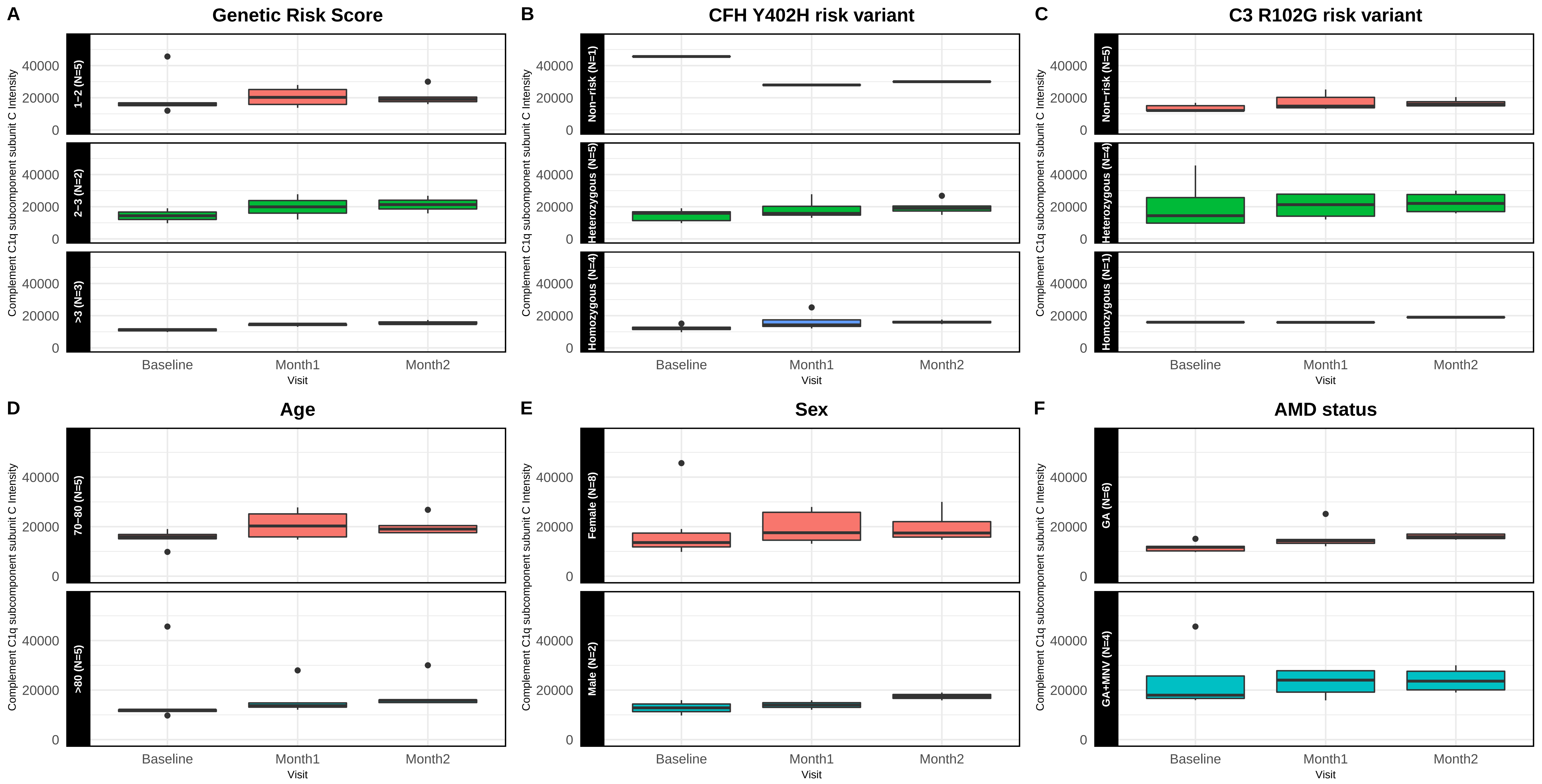

Supplementary Figure S286

Box plots depicting the distribution of Complement C1q subcomponent subunit C intensity at baseline, month 1, and month 2. Only AMD patients with measurements at all visits are included. The median, interquartile range, and outliers are displayed for each time point. Stratified on A) GRS. B) CFH Y402H risk variant. C) C3 R102G risk variant. D) Age. E) Sex. F) AMD status.

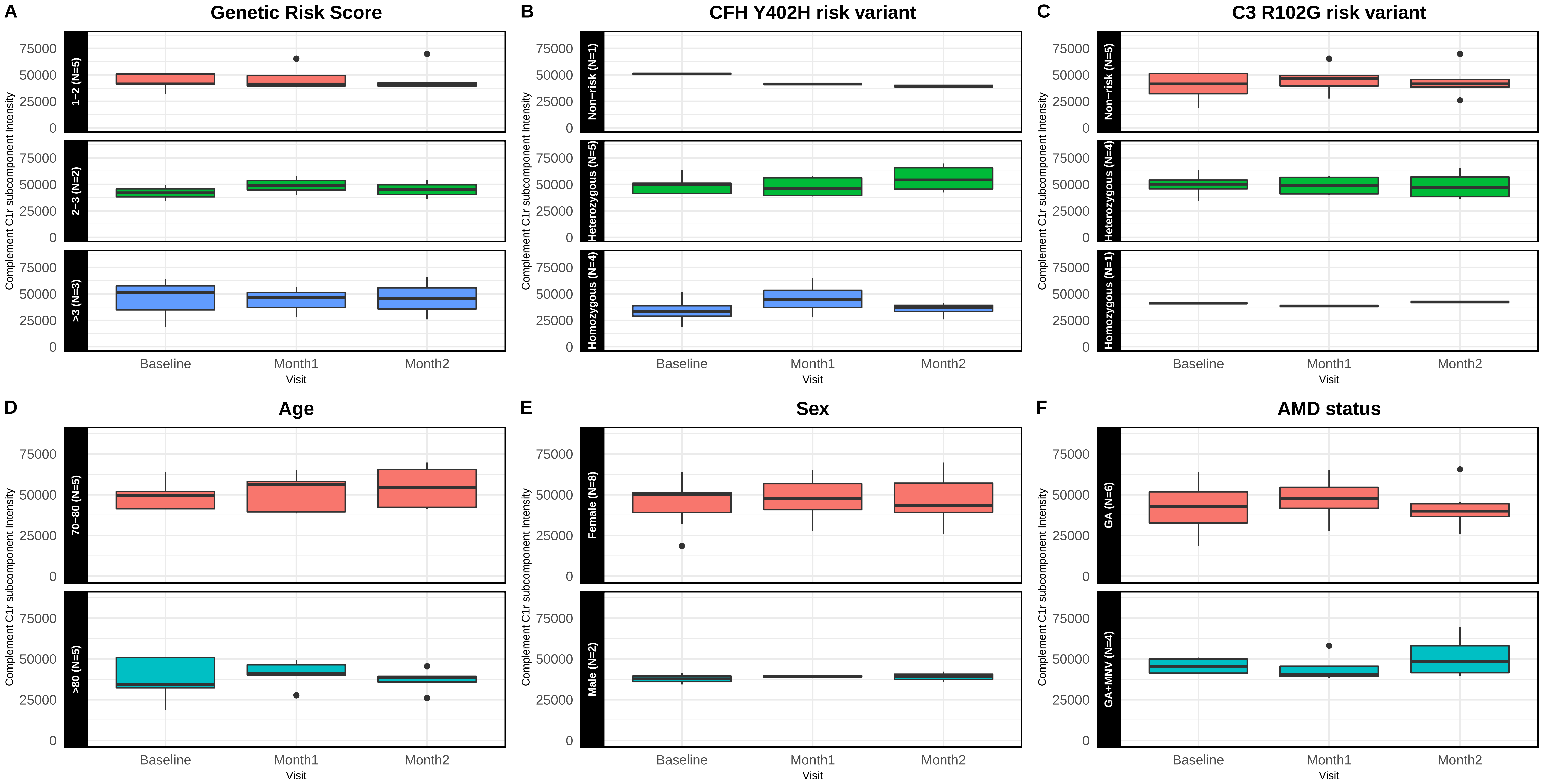

Supplementary Figure S287

Box plots depicting the distribution of Complement C1r subcomponent intensity at baseline, month 1, and month 2. Only AMD patients with measurements at all visits are included. The median, interquartile range, and outliers are displayed for each time point. Stratified on A) GRS. B) CFH Y402H risk variant. C) C3 R102G risk variant. D) Age. E) Sex. F) AMD status.

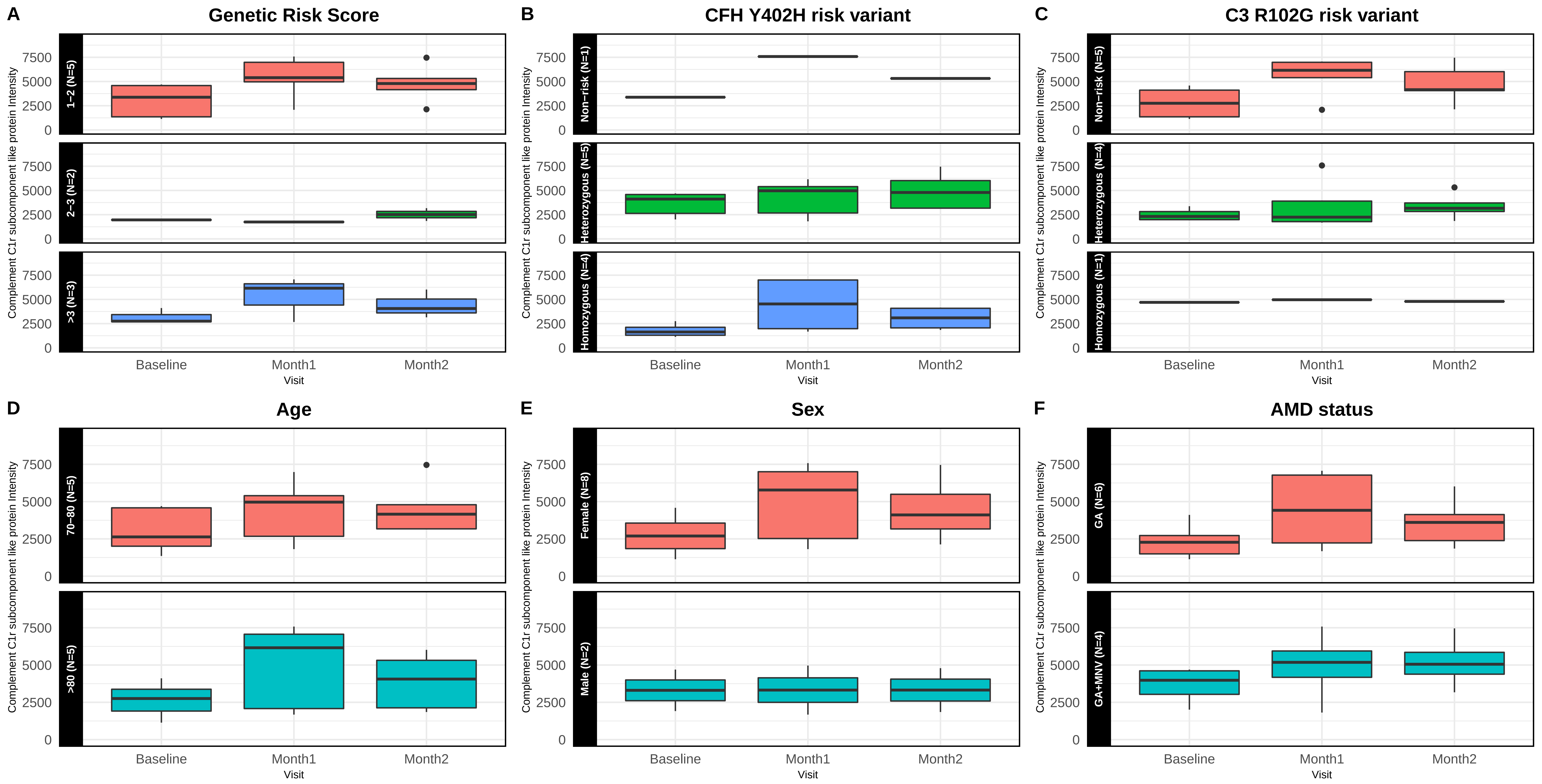

Supplementary Figure S288

Box plots depicting the distribution of Complement C1r subcomponent like protein intensity at baseline, month 1, and month 2. Only AMD patients with measurements at all visits are included. The median, interquartile range, and outliers are displayed for each time point. Stratified on A) GRS. B) CFH Y402H risk variant. C) C3 R102G risk variant. D) Age. E) Sex. F) AMD status.

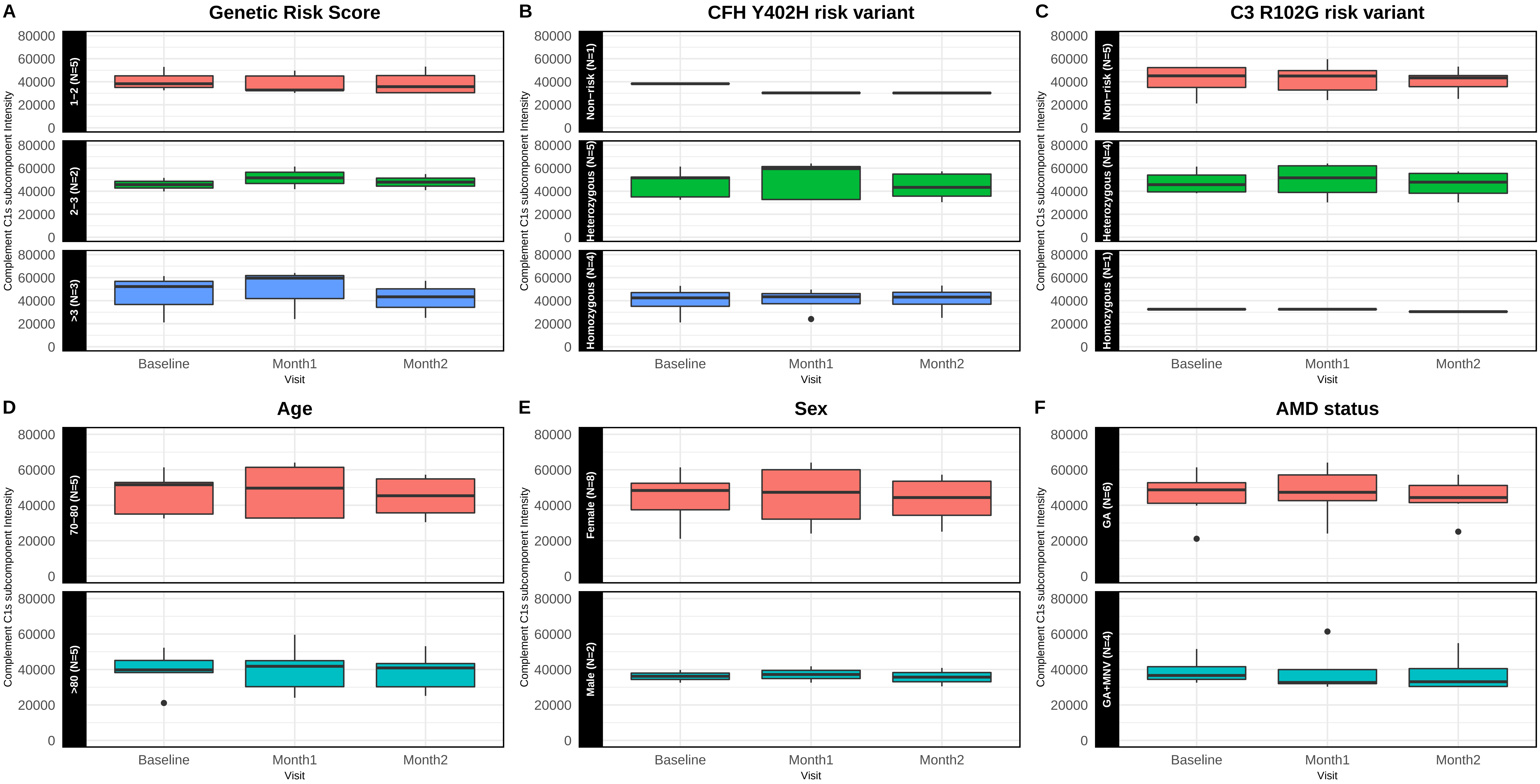

Supplementary Figure S289

Box plots depicting the distribution of Complement C1s subcomponent intensity at baseline, month 1, and month 2. Only AMD patients with measurements at all visits are included. The median, interquartile range, and outliers are displayed for each time point. Stratified on A) GRS. B) CFH Y402H risk variant. C) C3 R102G risk variant. D) Age. E) Sex. F) AMD status.

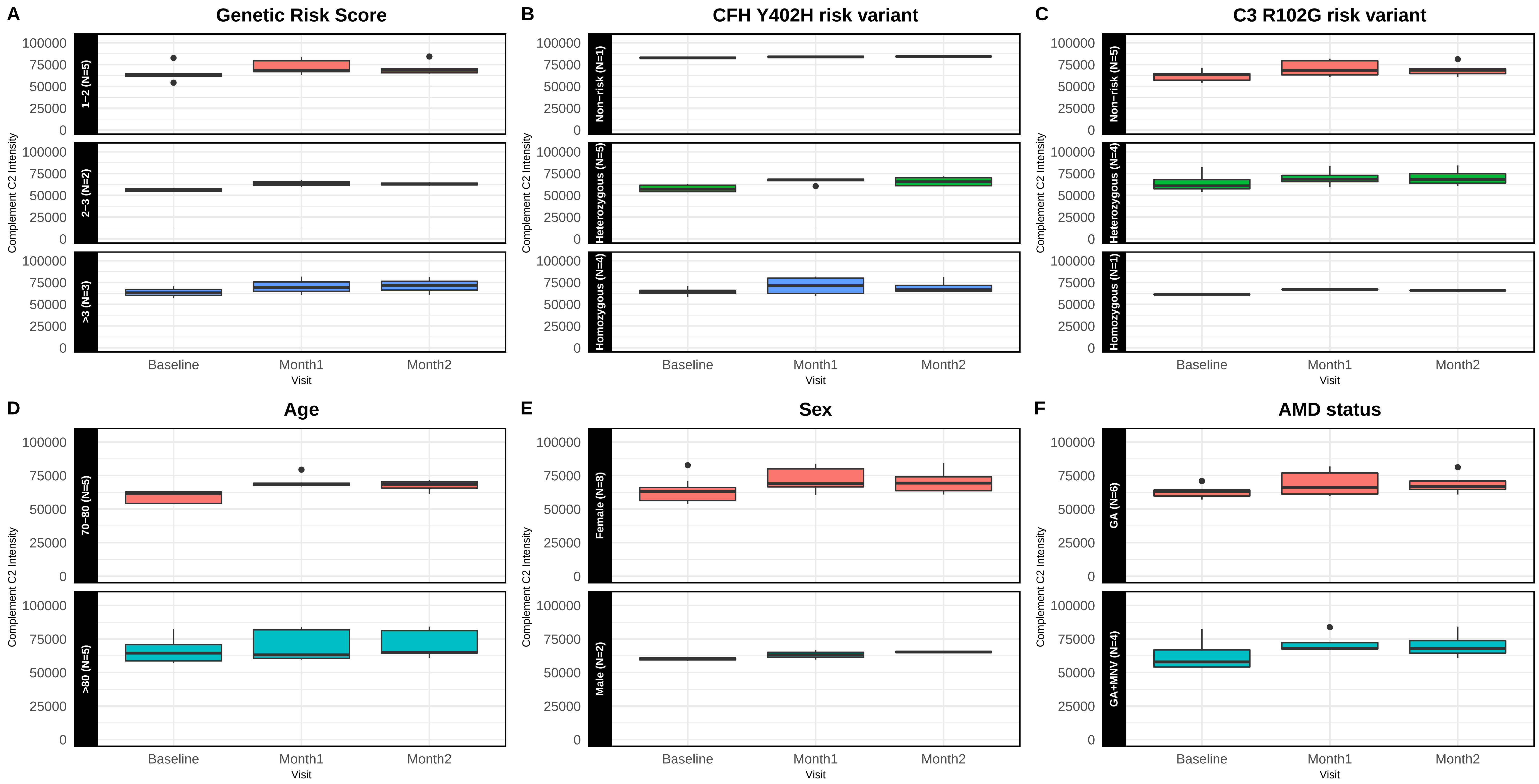

Supplementary Figure S290

Box plots depicting the distribution of Complement C2 intensity at baseline, month 1, and month 2. Only AMD patients with measurements at all visits are included. The median, interquartile range, and outliers are displayed for each time point. Stratified on A) GRS. B) CFH Y402H risk variant. C) C3 R102G risk variant. D) Age. E) Sex. F) AMD status.

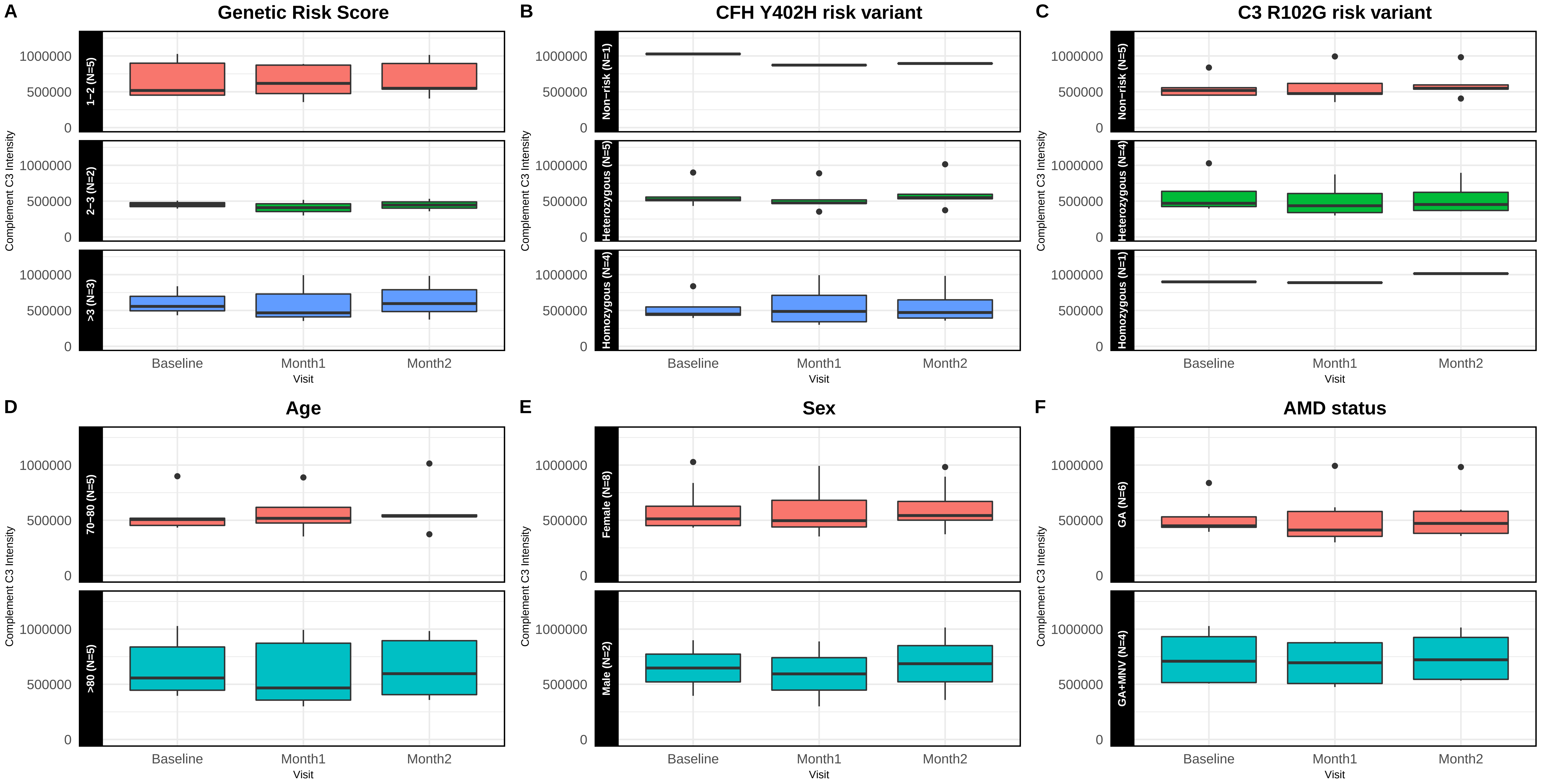

Supplementary Figure S291

Box plots depicting the distribution of Complement C3 intensity at baseline, month 1, and month 2. Only AMD patients with measurements at all visits are included. The median, interquartile range, and outliers are displayed for each time point. Stratified on A) GRS. B) CFH Y402H risk variant. C) C3 R102G risk variant. D) Age. E) Sex. F) AMD status.

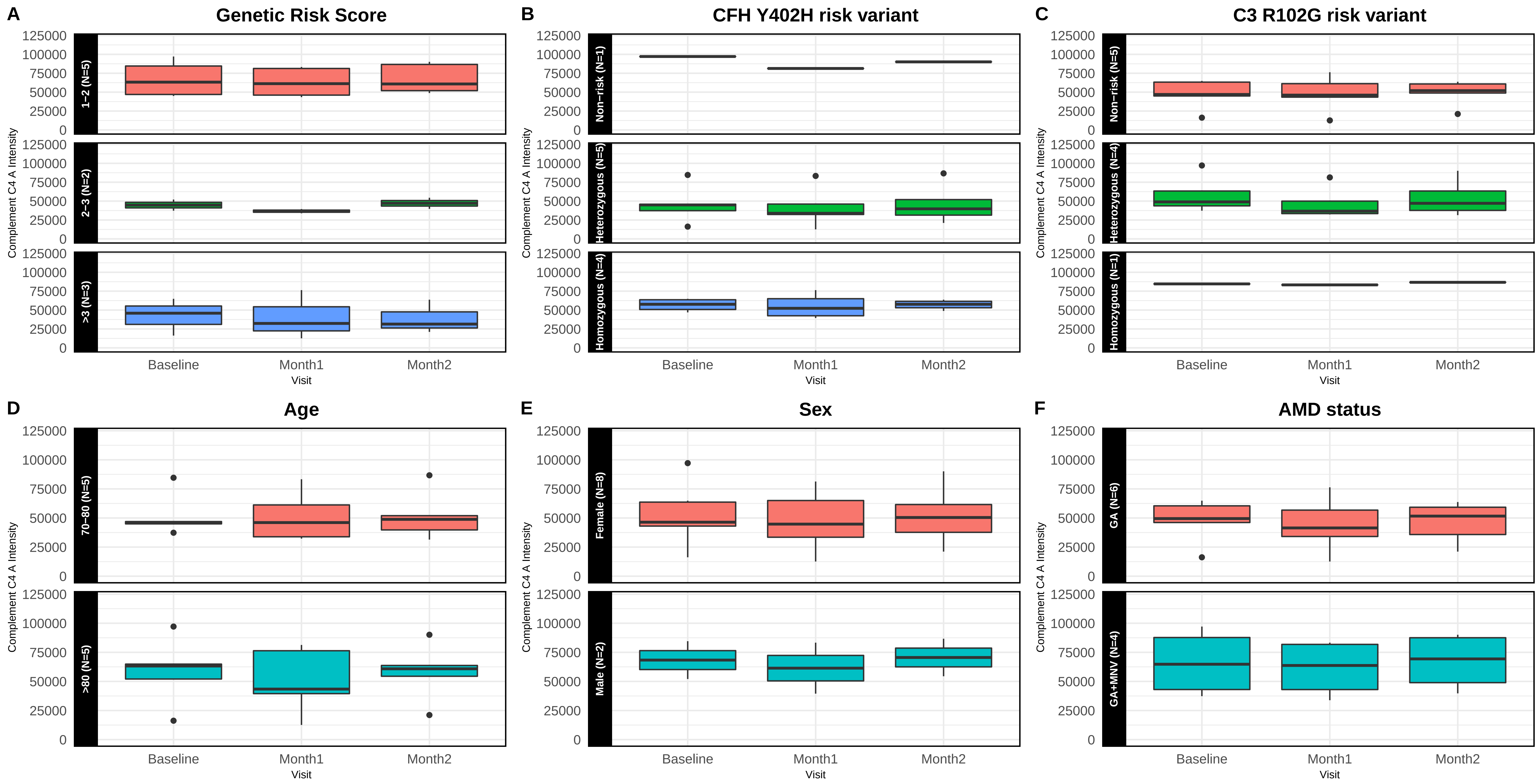

Supplementary Figure S292

Box plots depicting the distribution of Complement C4 A intensity at baseline, month 1, and month 2. Only AMD patients with measurements at all visits are included. The median, interquartile range, and outliers are displayed for each time point. Stratified on A) GRS. B) CFH Y402H risk variant. C) C3 R102G risk variant. D) Age. E) Sex. F) AMD status.

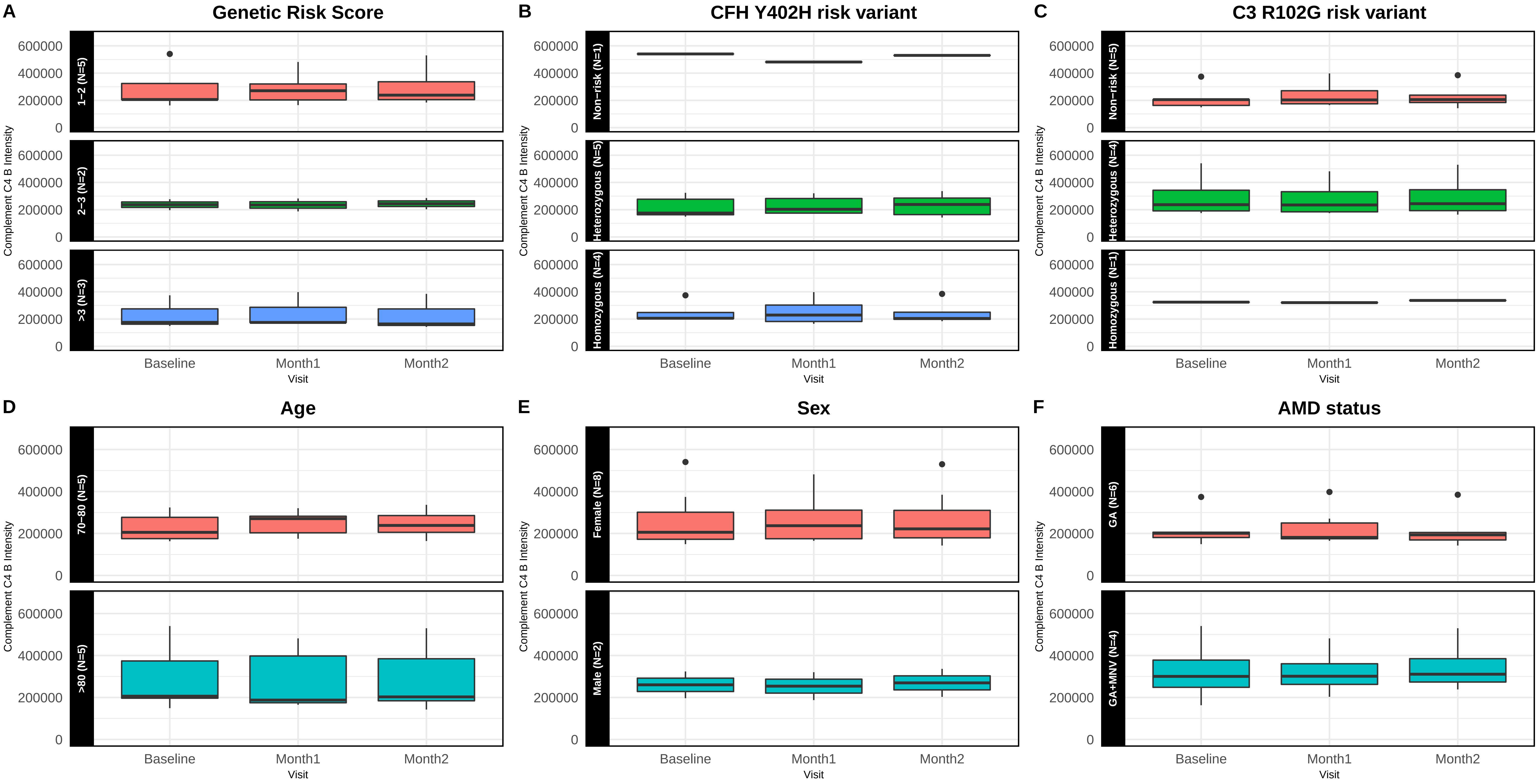

Supplementary Figure S293

Box plots depicting the distribution of Complement C4 B intensity at baseline, month 1, and month 2. Only AMD patients with measurements at all visits are included. The median, interquartile range, and outliers are displayed for each time point. Stratified on A) GRS. B) CFH Y402H risk variant. C) C3 R102G risk variant. D) Age. E) Sex. F) AMD status.

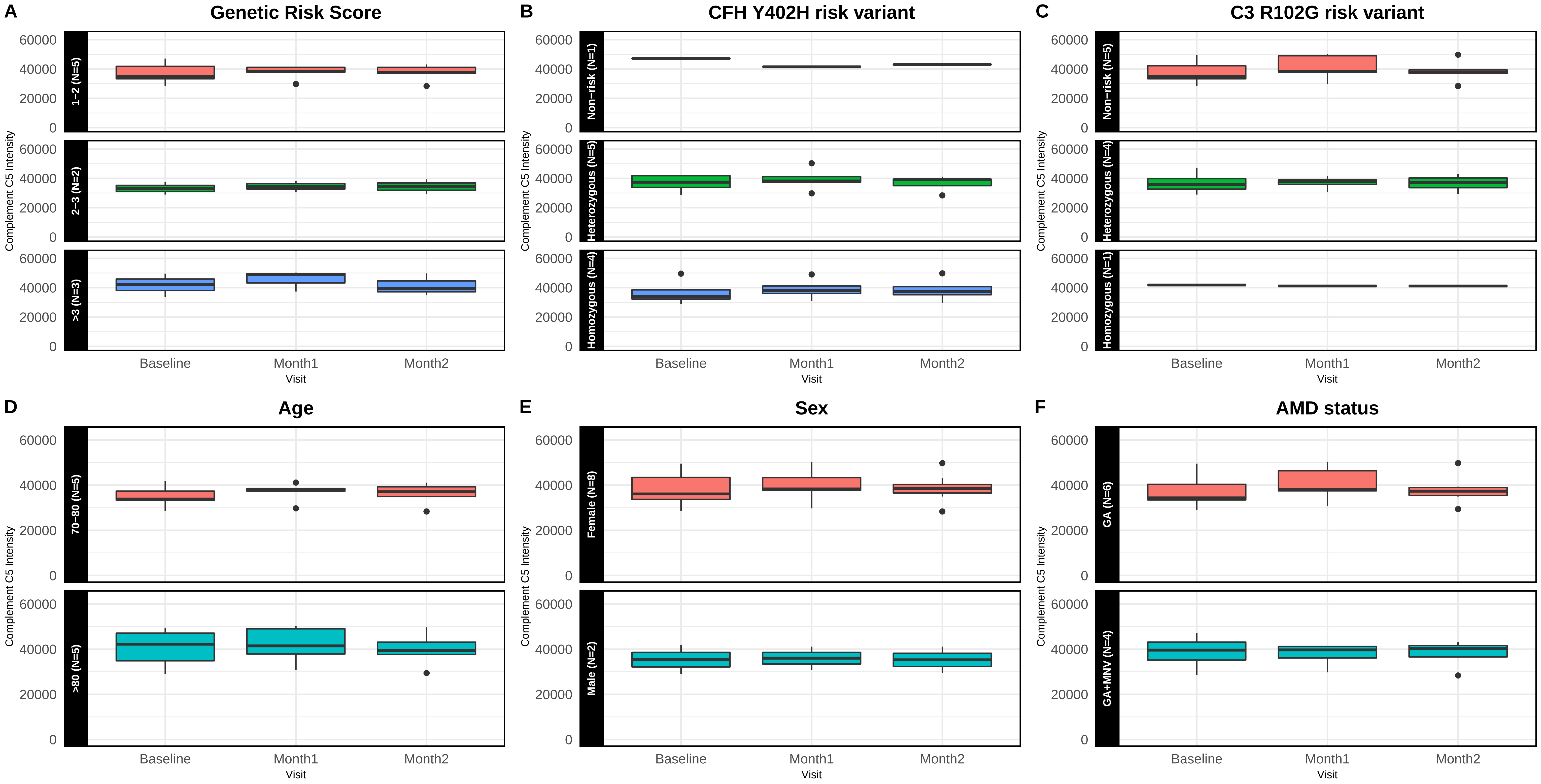

Supplementary Figure S294

Box plots depicting the distribution of Complement C5 intensity at baseline, month 1, and month 2. Only AMD patients with measurements at all visits are included. The median, interquartile range, and outliers are displayed for each time point. Stratified on A) GRS. B) CFH Y402H risk variant. C) C3 R102G risk variant. D) Age. E) Sex. F) AMD status.

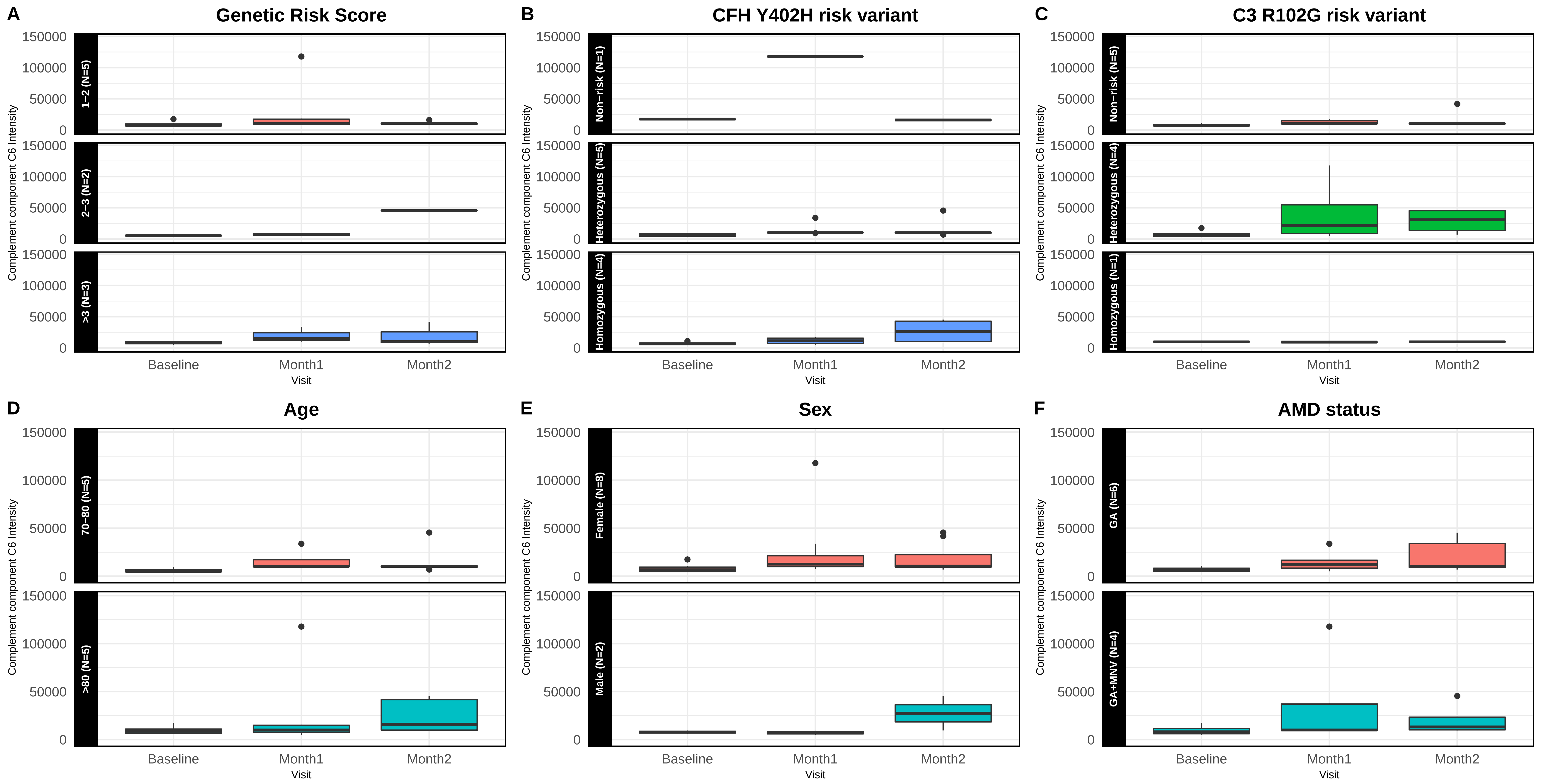

Supplementary Figure S295

Box plots depicting the distribution of Complement component C6 intensity at baseline, month 1, and month 2. Only AMD patients with measurements at all visits are included. The median, interquartile range, and outliers are displayed for each time point. Stratified on A) GRS. B) CFH Y402H risk variant. C) C3 R102G risk variant. D) Age. E) Sex. F) AMD status.

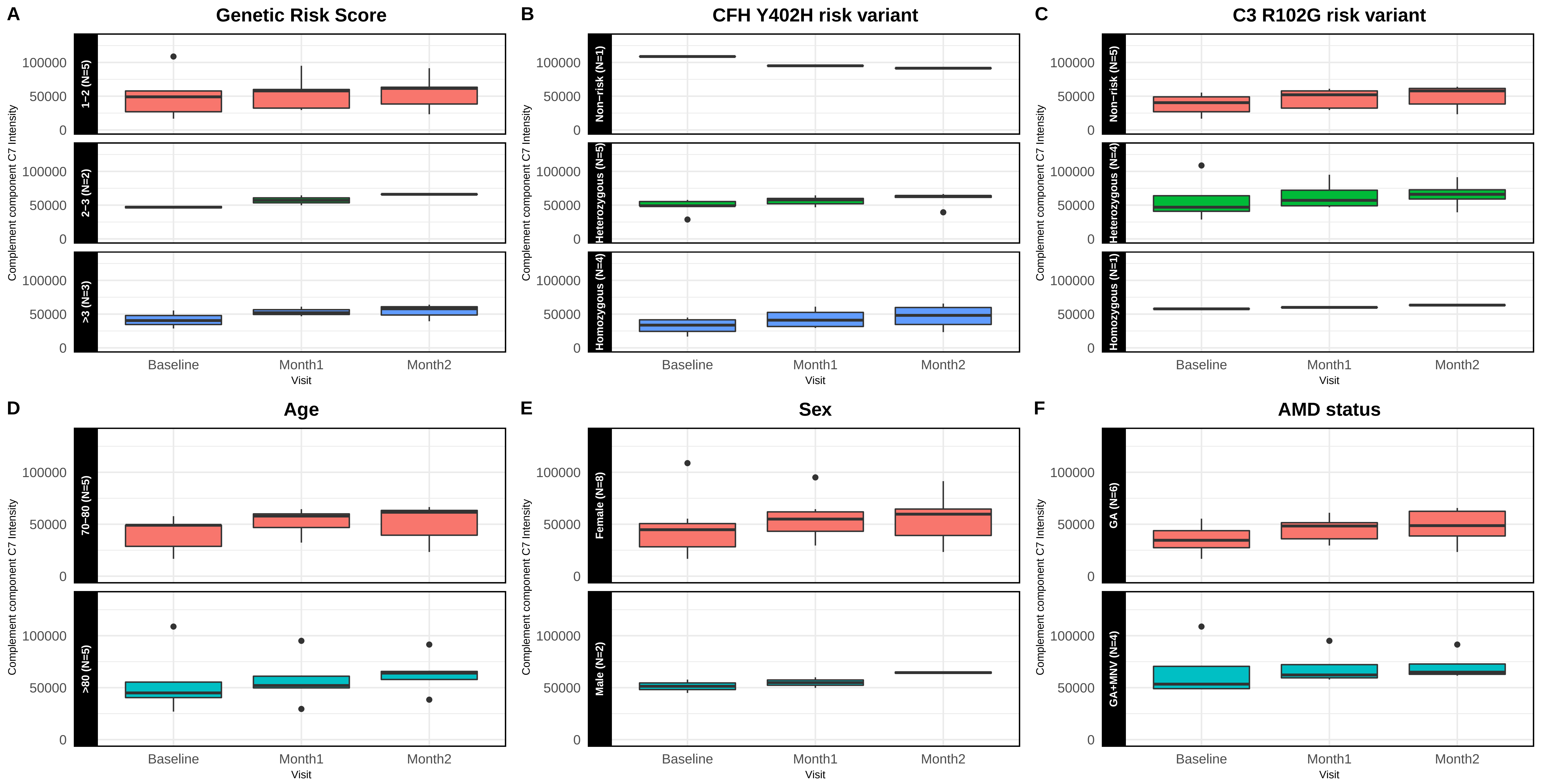

Supplementary Figure S296

Box plots depicting the distribution of Complement component C7 intensity at baseline, month 1, and month 2. Only AMD patients with measurements at all visits are included. The median, interquartile range, and outliers are displayed for each time point. Stratified on A) GRS. B) CFH Y402H risk variant. C) C3 R102G risk variant. D) Age. E) Sex. F) AMD status.

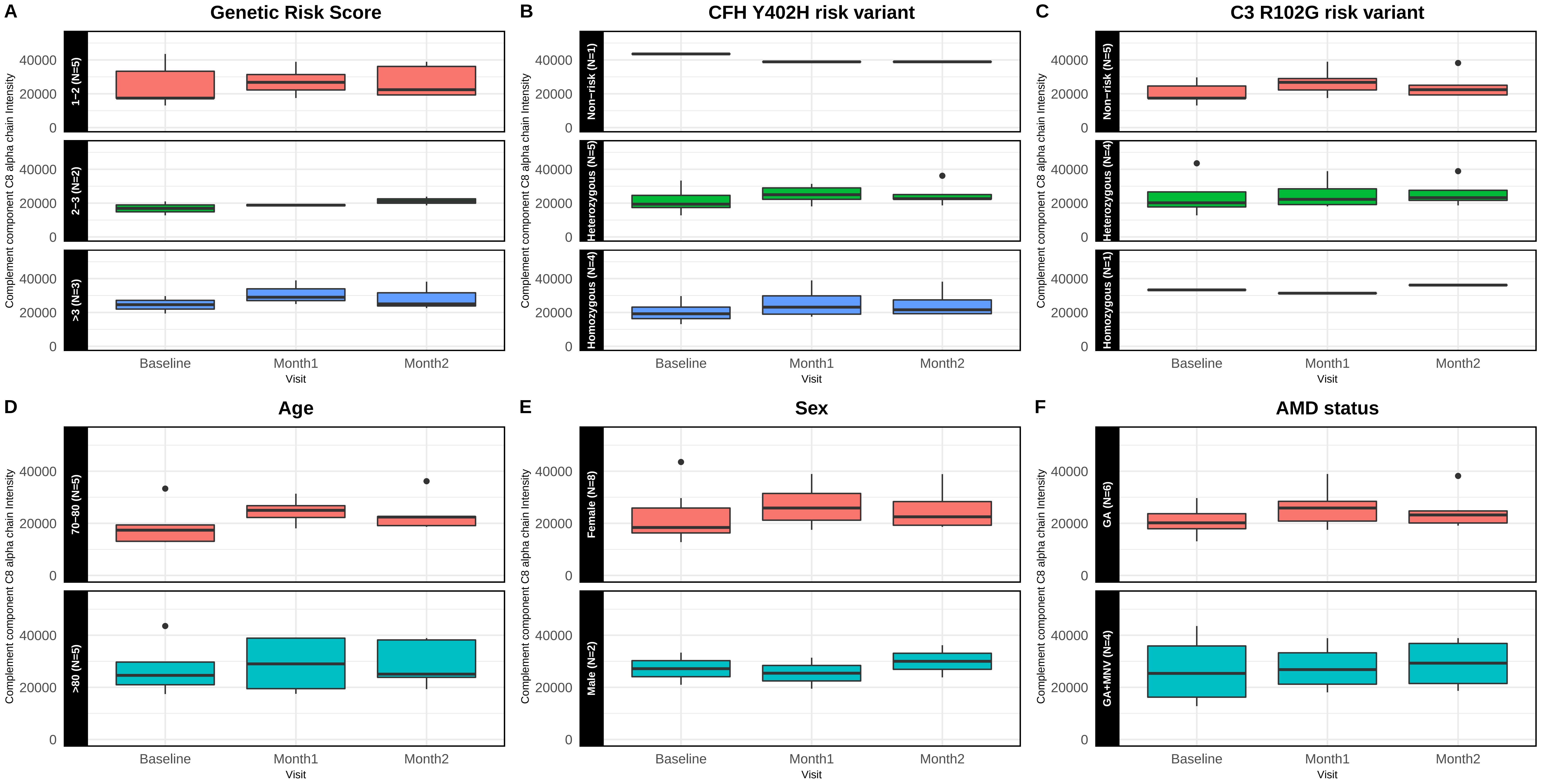

Supplementary Figure S297

Box plots depicting the distribution of Complement component C8 alpha chain intensity at baseline, month 1, and month 2. Only AMD patients with measurements at all visits are included. The median, interquartile range, and outliers are displayed for each time point. Stratified on A) GRS. B) CFH Y402H risk variant. C) C3 R102G risk variant. D) Age. E) Sex. F) AMD status.

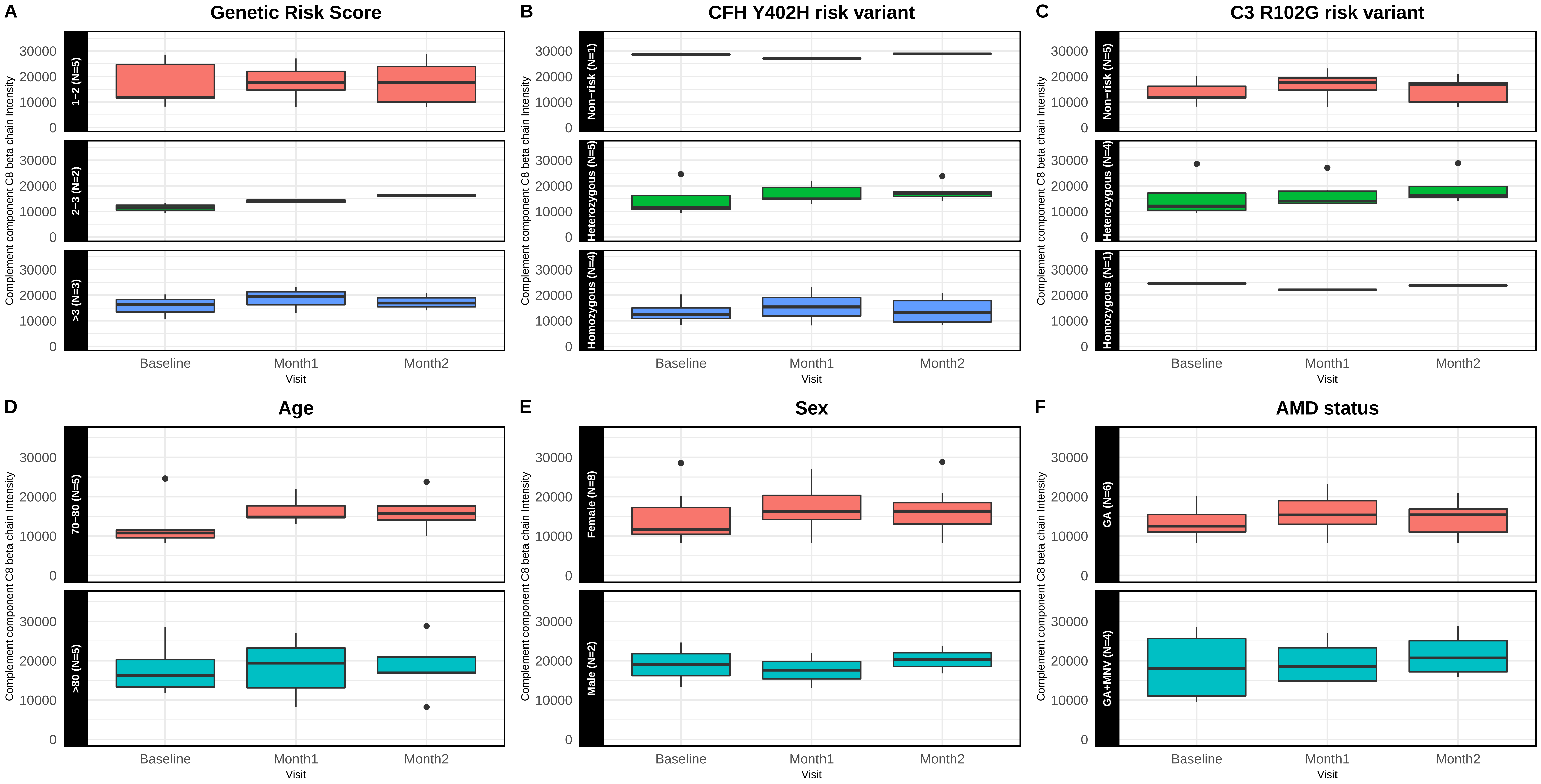

Supplementary Figure S298

Box plots depicting the distribution of Complement component C8 beta chain intensity at baseline, month 1, and month 2. Only AMD patients with measurements at all visits are included. The median, interquartile range, and outliers are displayed for each time point. Stratified on A) GRS. B) CFH Y402H risk variant. C) C3 R102G risk variant. D) Age. E) Sex. F) AMD status.

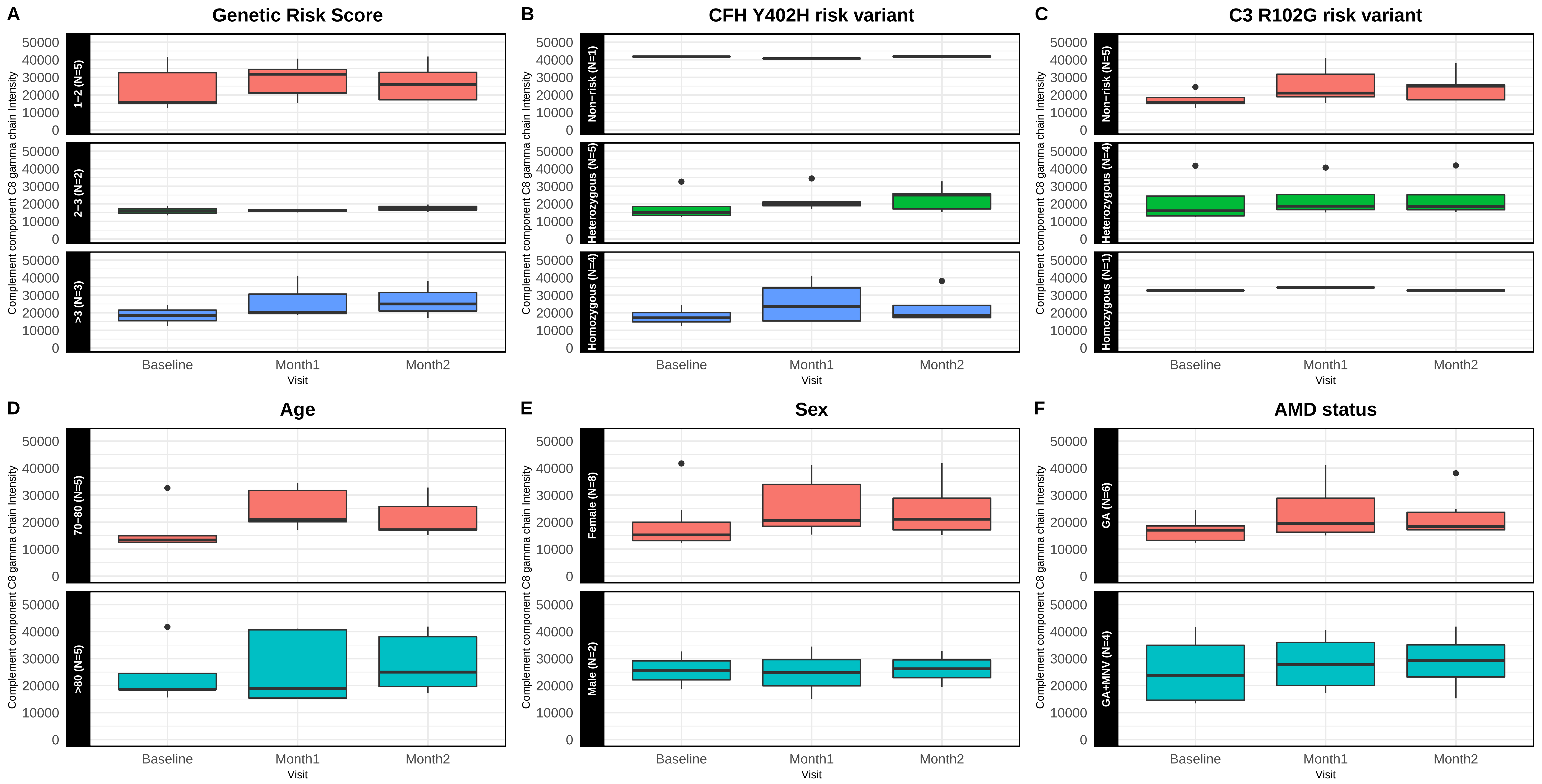

Supplementary Figure S299

Box plots depicting the distribution of Complement component C8 gamma chain intensity at baseline, month 1, and month 2. Only AMD patients with measurements at all visits are included. The median, interquartile range, and outliers are displayed for each time point. Stratified on A) GRS. B) CFH Y402H risk variant. C) C3 R102G risk variant. D) Age. E) Sex. F) AMD status.

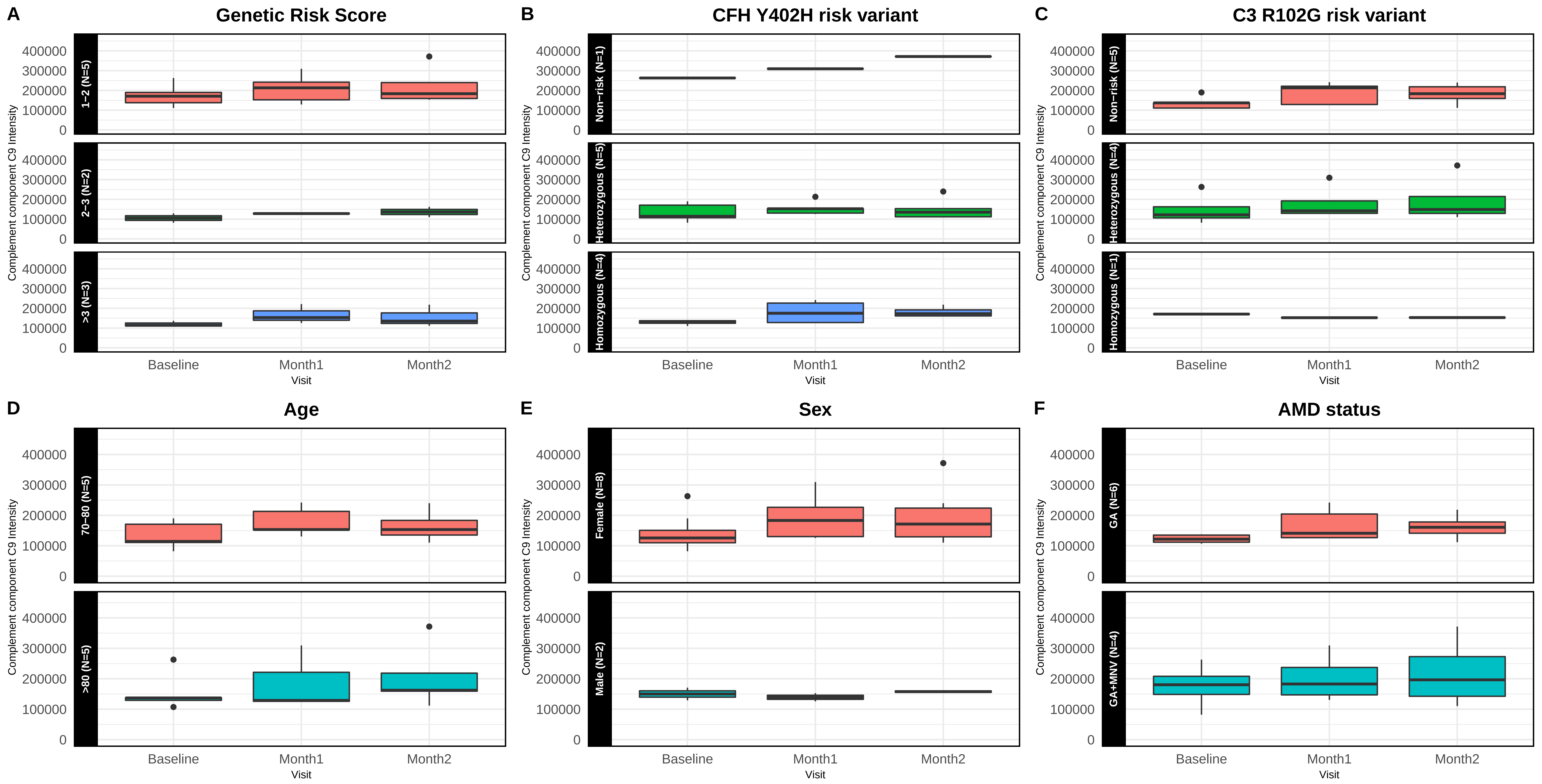

Supplementary Figure S300

Box plots depicting the distribution of Complement component C9 intensity at baseline, month 1, and month 2. Only AMD patients with measurements at all visits are included. The median, interquartile range, and outliers are displayed for each time point. Stratified on A) GRS. B) CFH Y402H risk variant. C) C3 R102G risk variant. D) Age. E) Sex. F) AMD status.

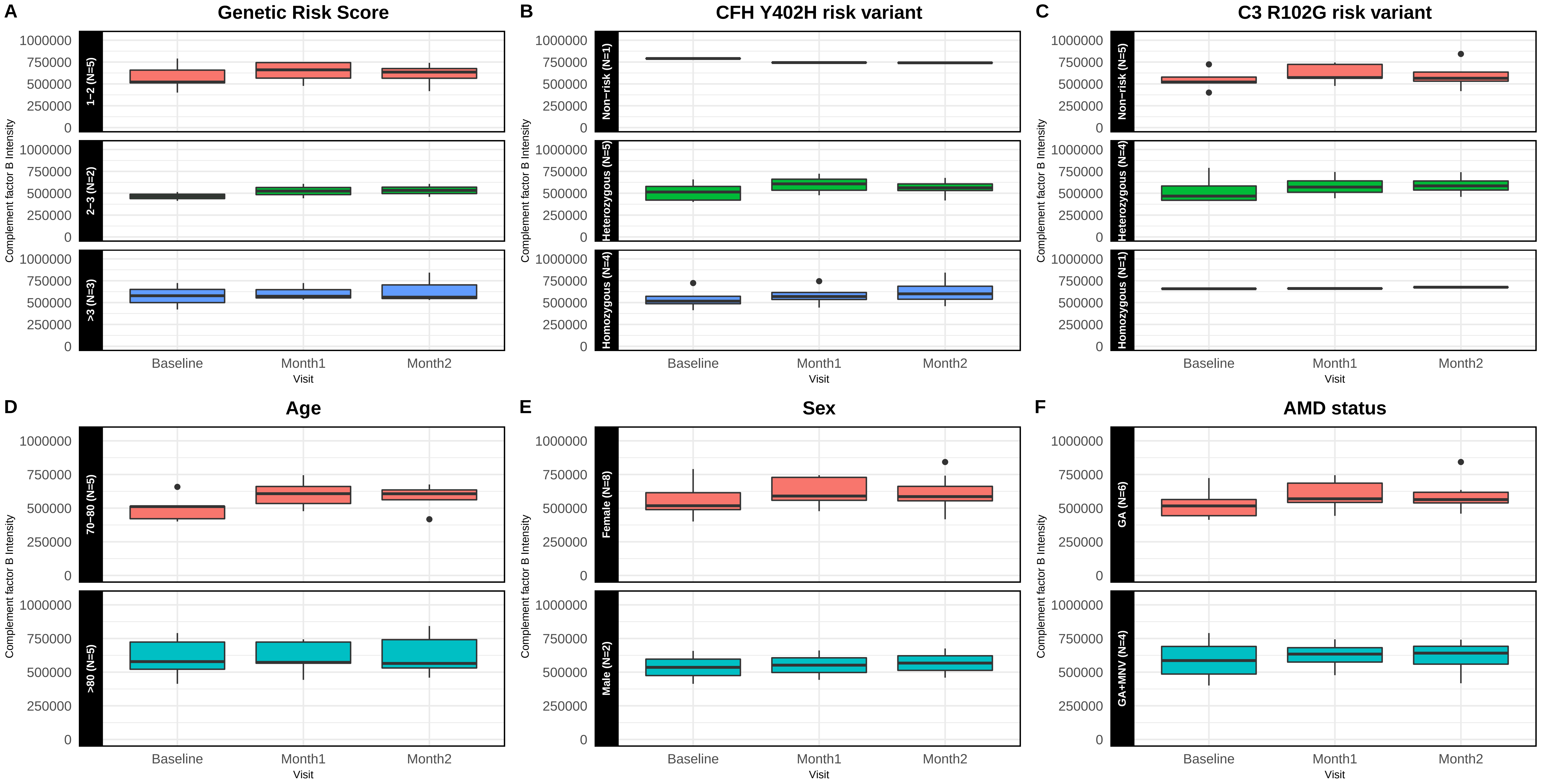

Supplementary Figure S301

Box plots depicting the distribution of Complement factor B intensity at baseline, month 1, and month 2. Only AMD patients with measurements at all visits are included. The median, interquartile range, and outliers are displayed for each time point. Stratified on A) GRS. B) CFH Y402H risk variant. C) C3 R102G risk variant. D) Age. E) Sex. F) AMD status.

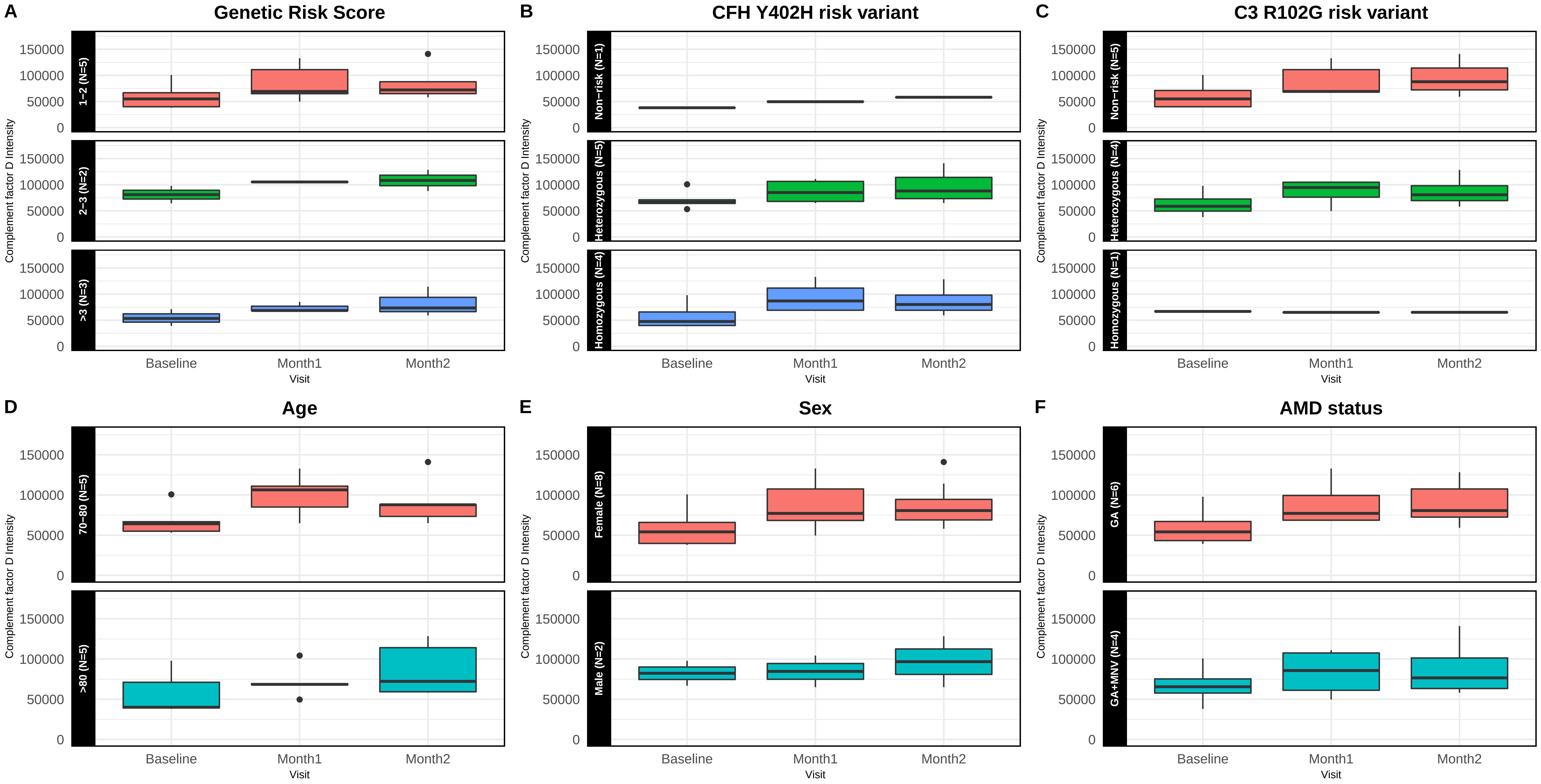

Supplementary Figure S302

Box plots depicting the distribution of Complement factor D intensity at baseline, month 1, and month 2. Only AMD patients with measurements at all visits are included. The median, interquartile range, and outliers are displayed for each time point. Stratified on A) GRS. B) CFH Y402H risk variant. C) C3 R102G risk variant. D) Age. E) Sex. F) AMD status.

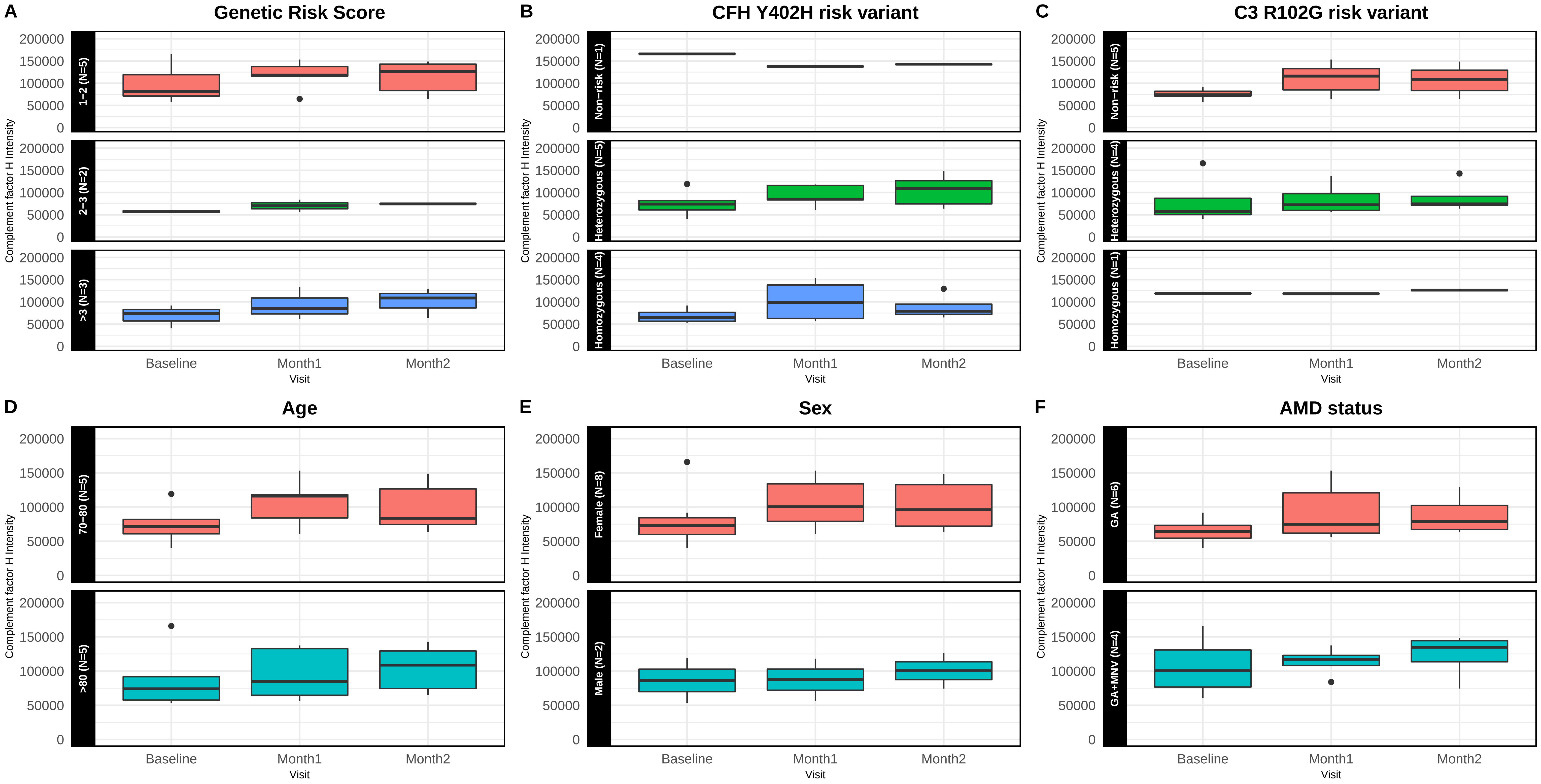

Supplementary Figure S303

Box plots depicting the distribution of Complement factor H intensity at baseline, month 1, and month 2. Only AMD patients with measurements at all visits are included. The median, interquartile range, and outliers are displayed for each time point. Stratified on A) GRS. B) CFH Y402H risk variant. C) C3 R102G risk variant. D) Age. E) Sex. F) AMD status.

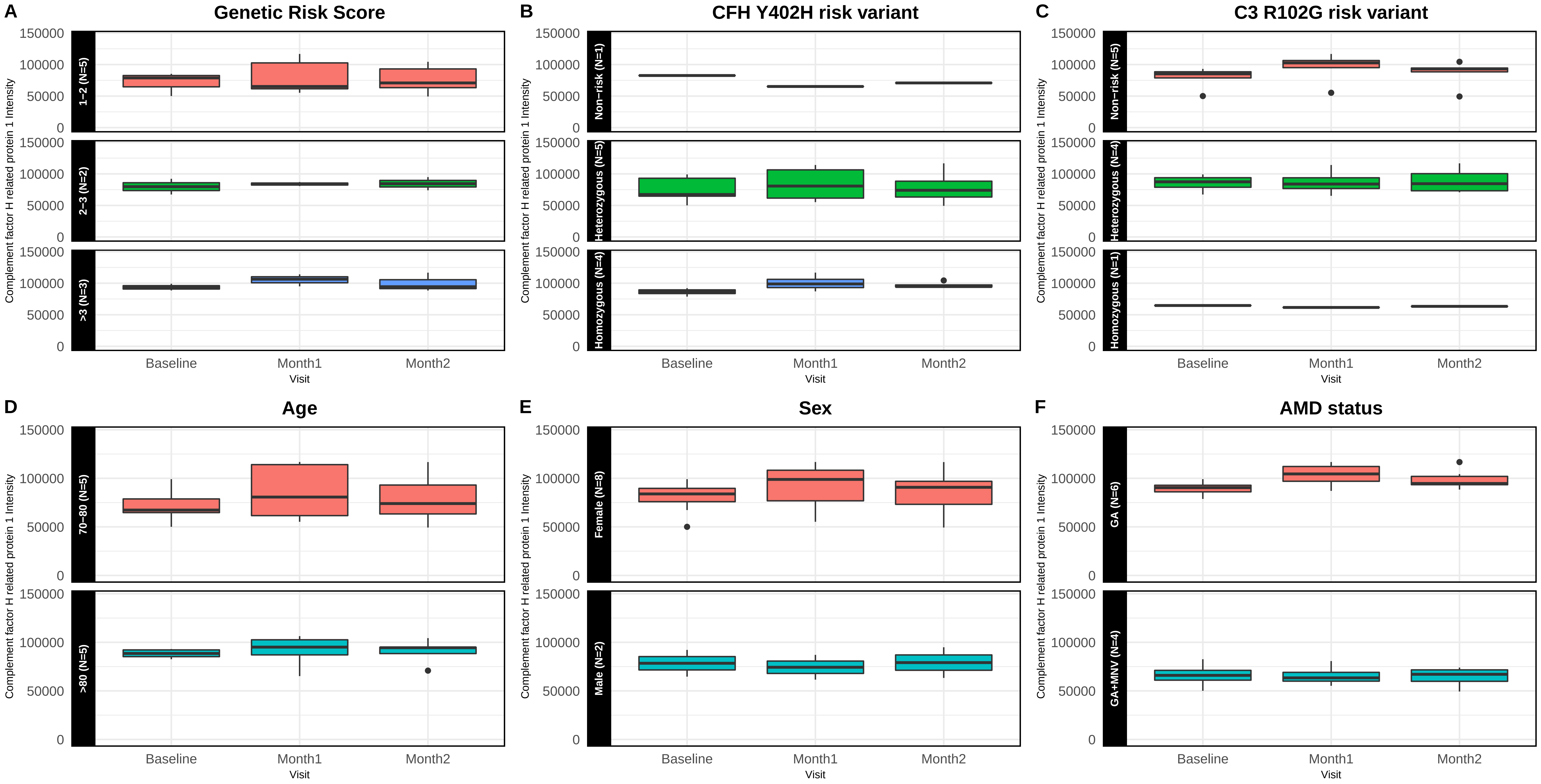

Supplementary Figure S304

Box plots depicting the distribution of Complement factor H related protein 1 intensity at baseline, month 1, and month 2. Only AMD patients with measurements at all visits are included. The median, interquartile range, and outliers are displayed for each time point. Stratified on A) GRS. B) CFH Y402H risk variant. C) C3 R102G risk variant. D) Age. E) Sex. F) AMD status.

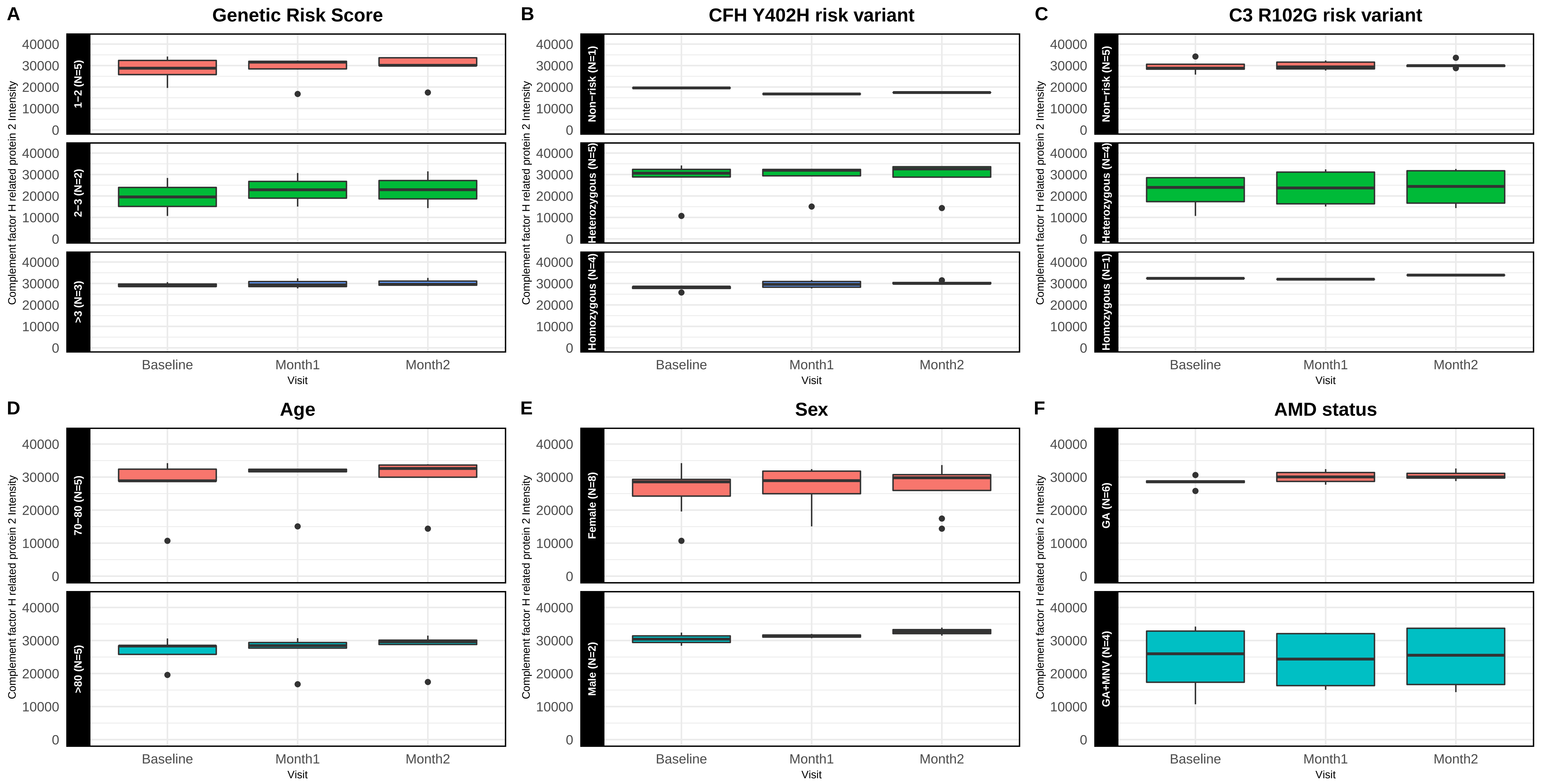

Supplementary Figure S305

Box plots depicting the distribution of Complement factor H related protein 2 intensity at baseline, month 1, and month 2. Only AMD patients with measurements at all visits are included. The median, interquartile range, and outliers are displayed for each time point. Stratified on A) GRS. B) CFH Y402H risk variant. C) C3 R102G risk variant. D) Age. E) Sex. F) AMD status.

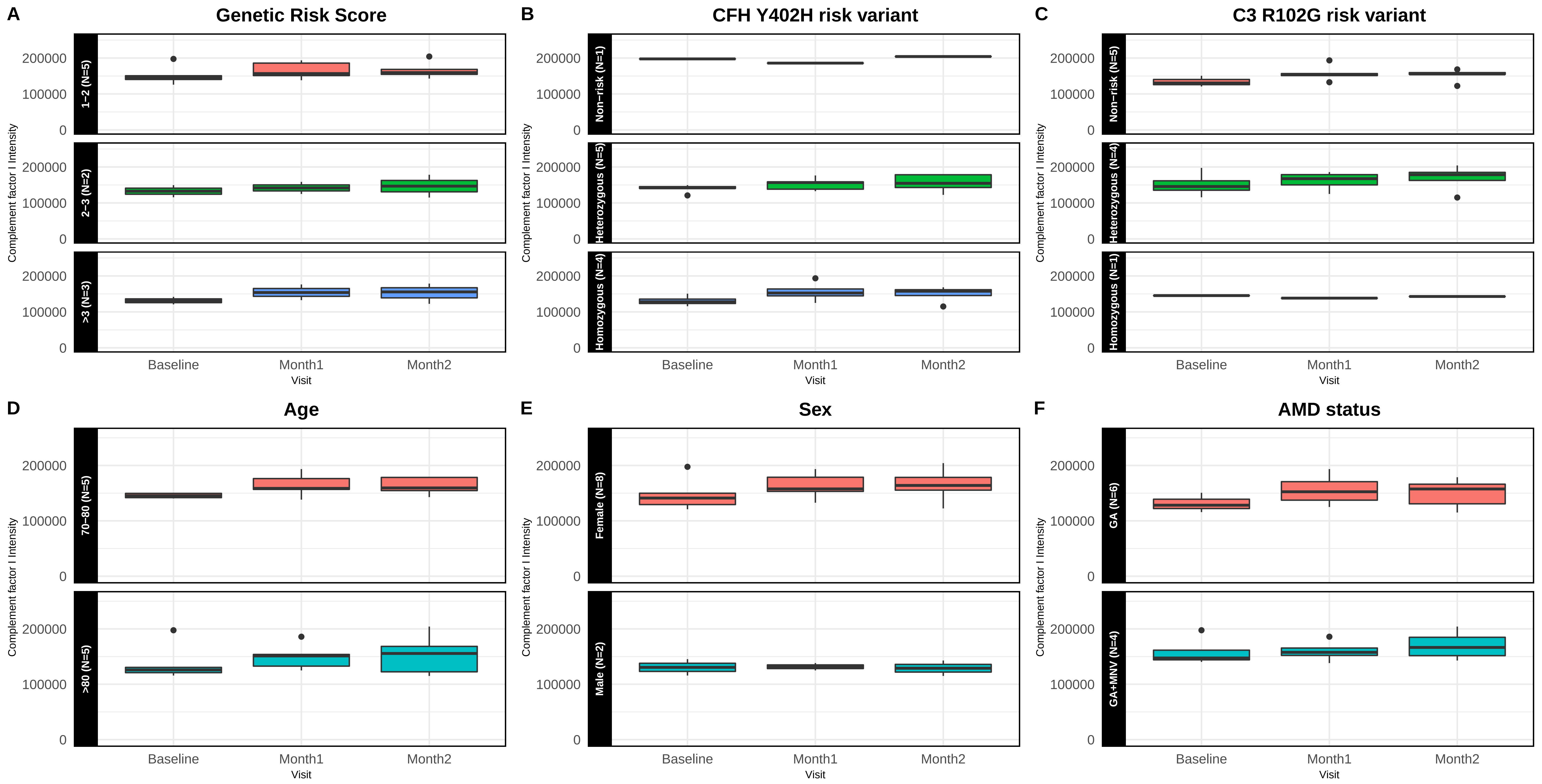

Supplementary Figure S306

Box plots depicting the distribution of Complement factor I intensity at baseline, month 1, and month 2. Only AMD patients with measurements at all visits are included. The median, interquartile range, and outliers are displayed for each time point. Stratified on A) GRS. B) CFH Y402H risk variant. C) C3 R102G risk variant. D) Age. E) Sex. F) AMD status.

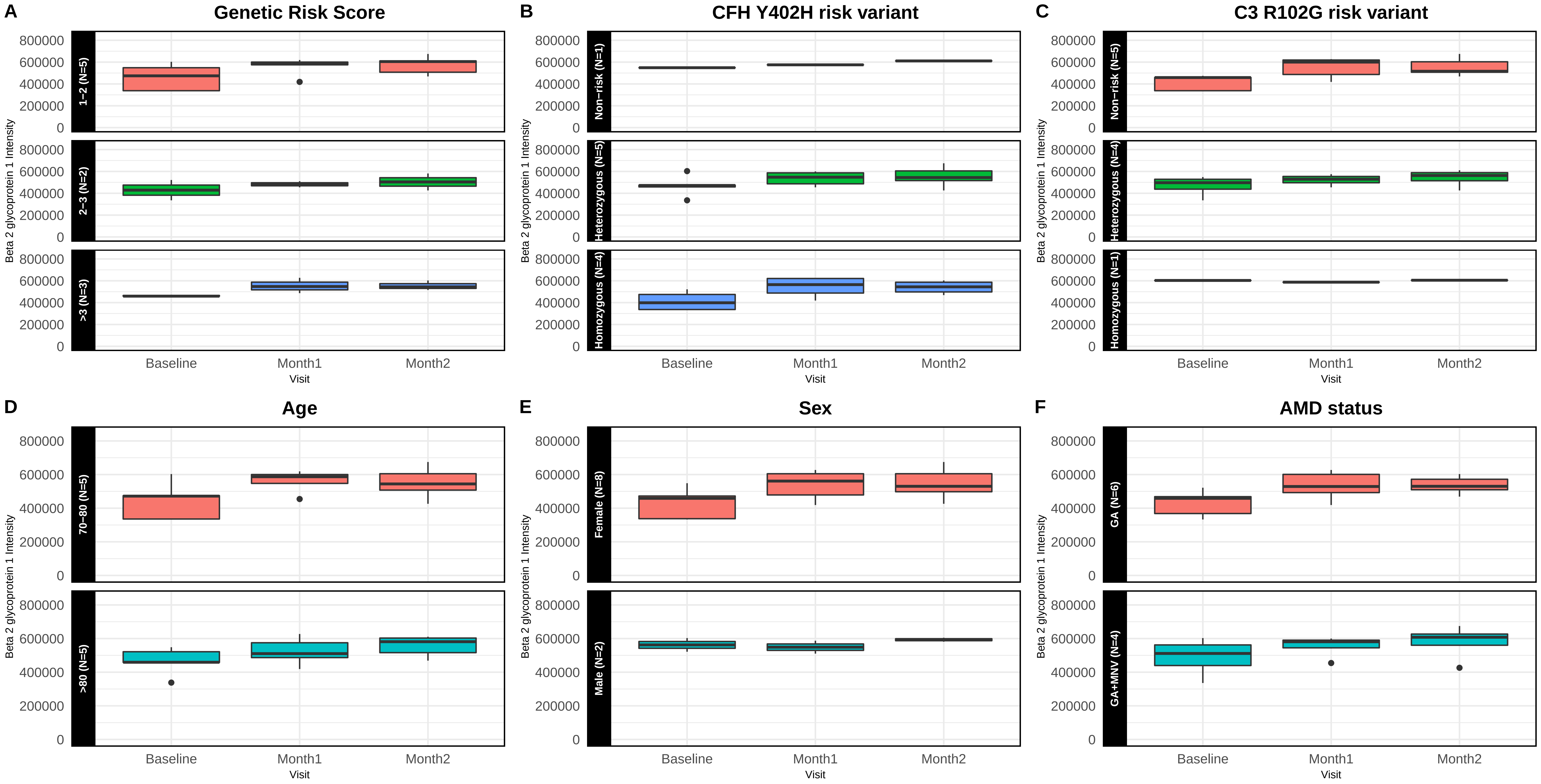

Supplementary Figure S307

Box plots depicting the distribution of Beta 2 glycoprotein 1 intensity at baseline, month 1, and month 2. Only AMD patients with measurements at all visits are included. The median, interquartile range, and outliers are displayed for each time point. Stratified on A) GRS. B) CFH Y402H risk variant. C) C3 R102G risk variant. D) Age. E) Sex. F) AMD status.

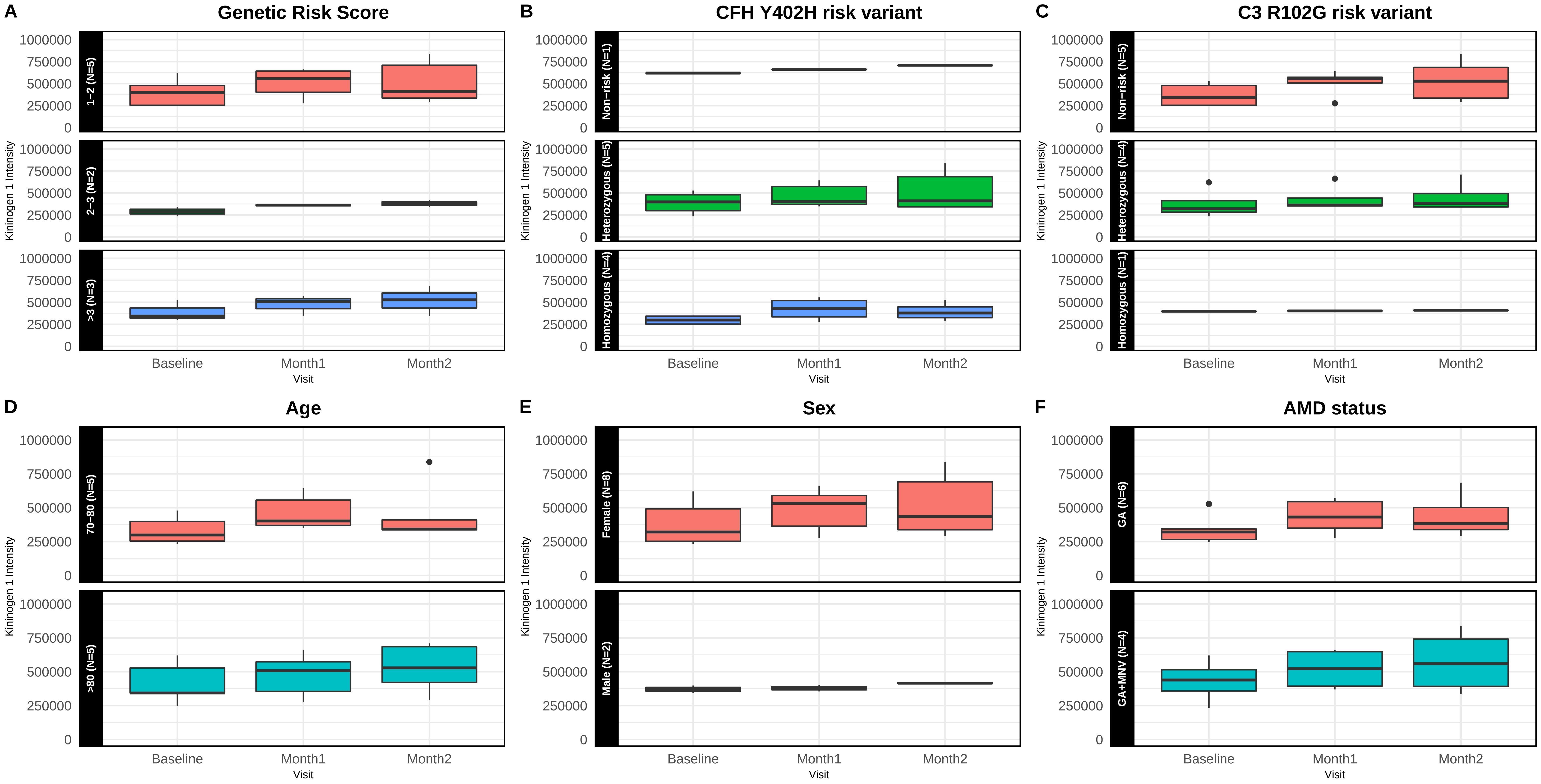

Supplementary Figure S308

Box plots depicting the distribution of Kininogen 1 intensity at baseline, month 1, and month 2. Only AMD patients with measurements at all visits are included. The median, interquartile range, and outliers are displayed for each time point. Stratified on A) GRS. B) CFH Y402H risk variant. C) C3 R102G risk variant. D) Age. E) Sex. F) AMD status.

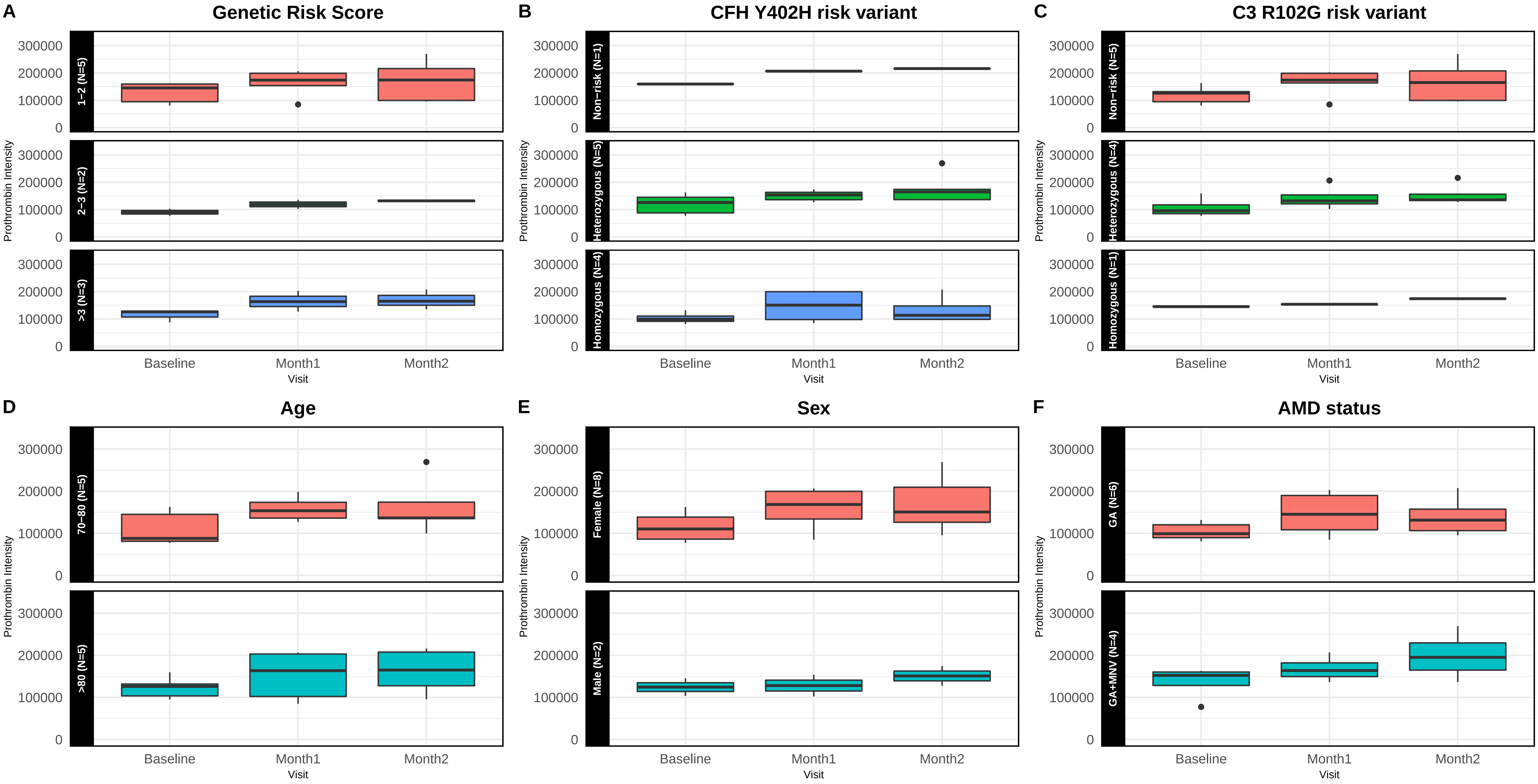

Supplementary Figure S309

Box plots depicting the distribution of Prothrombin intensity at baseline, month 1, and month 2. Only AMD patients with measurements at all visits are included. The median, interquartile range, and outliers are displayed for each time point. Stratified on A) GRS. B) CFH Y402H risk variant. C) C3 R102G risk variant. D) Age. E) Sex. F) AMD status.

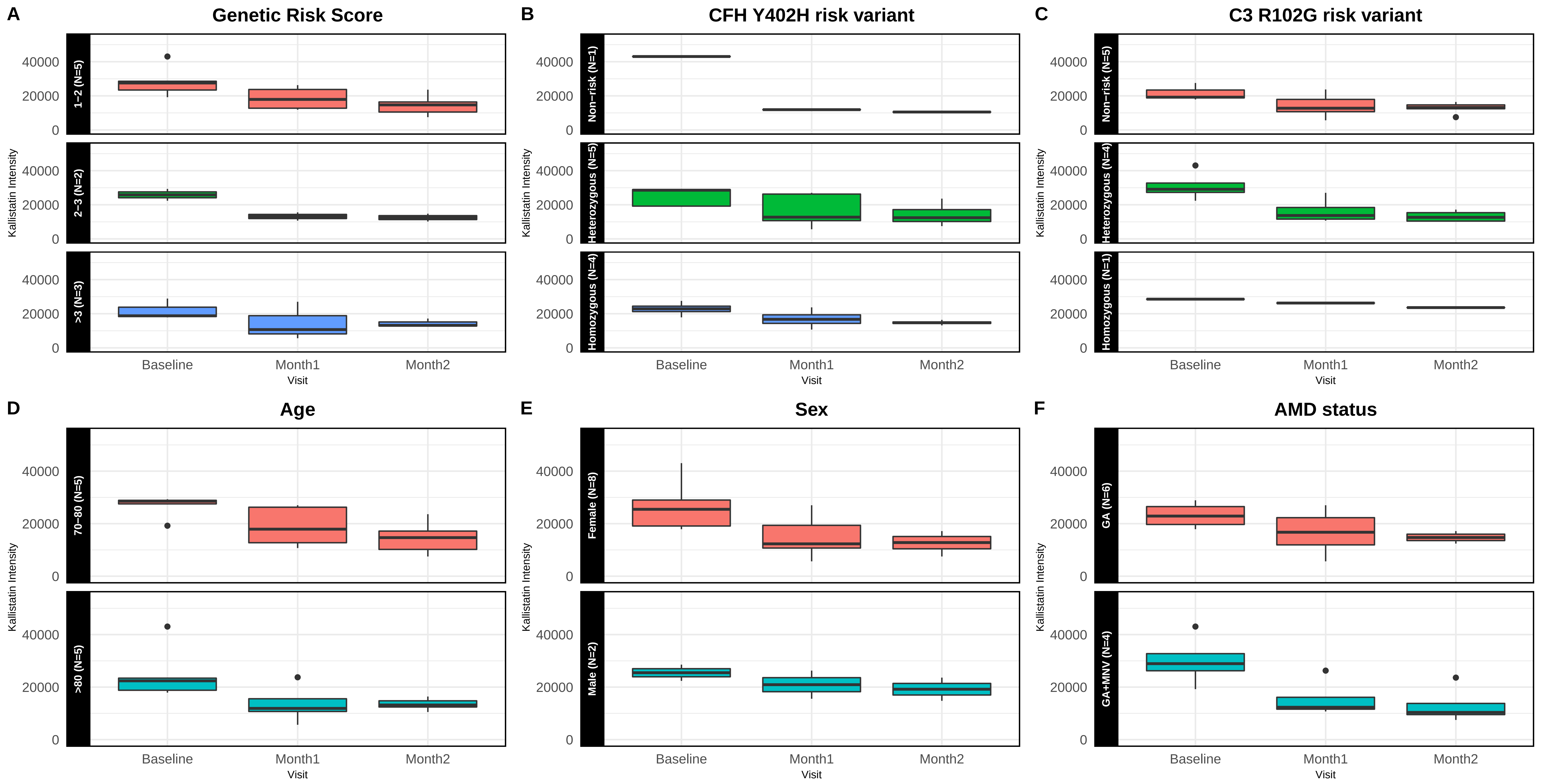

Supplementary Figure S310

Box plots depicting the distribution of Kallistatin intensity at baseline, month 1, and month 2. Only AMD patients with measurements at all visits are included. The median, interquartile range, and outliers are displayed for each time point. Stratified on A) GRS. B) CFH Y402H risk variant. C) C3 R102G risk variant. D) Age. E) Sex. F) AMD status.

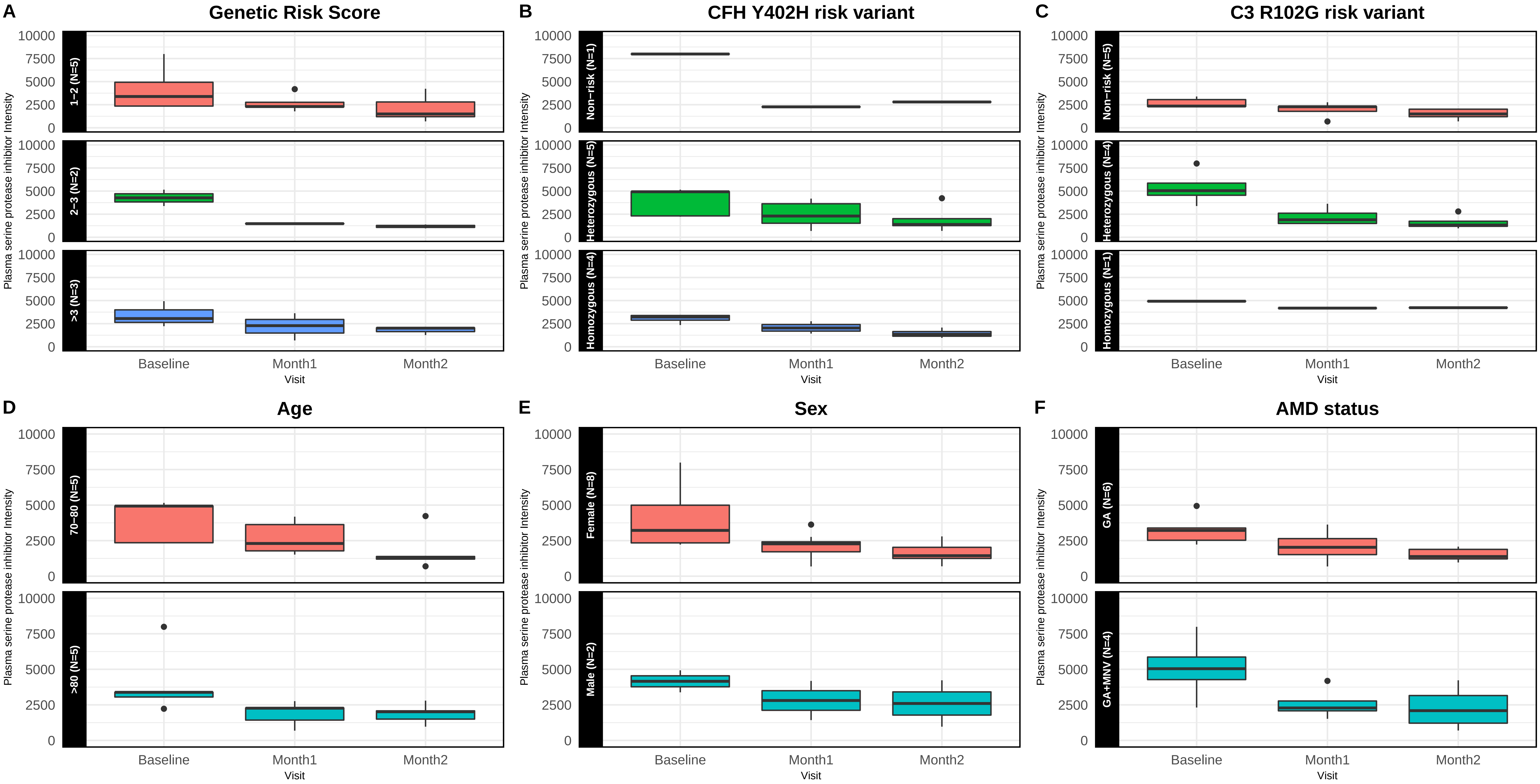

Supplementary Figure S311

Box plots depicting the distribution of Plasma serine protease inhibitor intensity at baseline, month 1, and month 2. Only AMD patients with measurements at all visits are included. The median, interquartile range, and outliers are displayed for each time point. Stratified on A) GRS. B) CFH Y402H risk variant. C) C3 R102G risk variant. D) Age. E) Sex. F) AMD status.

Description	GeneRatio	BgRatio	pvalue	p.adjust	qvalue	geneID
Complement cascade	14/53	58/11091	4.349969e−21	5.524460e−19	4.304179e−19	CPB2/C1QA/C1QB/C1QC/C2/C6/C7/C8A/C8G/CFD/CFH/SERPING1/F2/VTN
Regulation of Complement cascade	13/53	47/11091	1.723026e−20	1.094121e−18	8.524444e−19	CPB2/C1QA/C1QB/C1QC/C2/C6/C7/C8A/C8G/CFH/SERPING1/F2/VTN
Platelet degranulation	14/53	129/11091	6.614070e−16	2.799956e−14	2.181483e−14	ALB/ORM1/ORM2/A1BG/AHSG/APOH/CFD/HRG/IGF2/SERPINA4/KNG1/SERPING1/PFN1/CLEC3B
Response to elevated platelet cytosolic Ca2+	14/53	134/11091	1.139603e−15	3.618241e−14	2.819019e−14	ALB/ORM1/ORM2/A1BG/AHSG/APOH/CFD/HRG/IGF2/SERPINA4/KNG1/SERPING1/PFN1/CLEC3B
Platelet activation, signaling and aggregation	15/53	263/11091	7.907314e−13	2.008458e−11	1.564816e−11	ALB/ORM1/ORM2/A1BG/AHSG/APOH/CFD/HRG/IGF2/SERPINA4/KNG1/SERPING1/PFN1/F2/CLEC3B
Intrinsic Pathway of Fibrin Clot Formation	7/53	23/11091	8.720532e−12	1.845846e−10	1.438123e−10	F9/F10/F12/KNG1/SERPING1/SERPINA5/F2
Formation of Fibrin Clot (Clotting Cascade)	7/53	39/11091	5.161659e−10	9.364725e−09	7.296180e−09	F9/F10/F12/KNG1/SERPING1/SERPINA5/F2
Regulation of Insulin−like Growth Factor (IGF) transport and uptake by Insulin−like Growth Factor Binding Proteins (IGFBPs)	9/53	125/11091	6.406271e−09	1.016995e−07	7.923545e−08	ALB/AHSG/CP/CST3/IGFBP7/IGF2/ITIH2/KNG1/F2
Defects of contact activation system (CAS) and kallikrein/kinin system (KKS)	5/53	16/11091	8.621251e−09	1.216554e−07	9.478335e−08	F9/F10/F12/SERPING1/F2
Diseases of hemostasis	5/53	19/11091	2.270308e−08	2.883291e−07	2.246410e−07	F9/F10/F12/SERPING1/F2
Initial triggering of complement	5/53	23/11091	6.475527e−08	7.476291e−07	5.824876e−07	C1QA/C1QB/C1QC/C2/CFD
Scavenging of heme from plasma	4/53	13/11091	3.218381e−07	3.406120e−06	2.653753e−06	ALB/HBB/HPX/AMBP
Post−translational protein phosphorylation	7/53	108/11091	7.277543e−07	7.109600e−06	5.539183e−06	ALB/AHSG/CP/CST3/IGFBP7/ITIH2/KNG1
Gamma−carboxylation of protein precursors	3/53	10/11091	1.207652e−05	1.022479e−04	7.966265e−05	F9/F10/F2
Removal of aminoterminal propeptides from gamma−carboxylated proteins	3/53	10/11091	1.207652e−05	1.022479e−04	7.966265e−05	F9/F10/F2
Gamma−carboxylation, transport, and amino−terminal cleavage of proteins	3/53	11/11091	1.654911e−05	1.313586e−04	1.023432e−04	F9/F10/F2
Creation of C4 and C2 activators	3/53	14/11091	3.613970e−05	2.699848e−04	2.103487e−04	C1QA/C1QB/C1QC
Binding and Uptake of Ligands by Scavenger Receptors	4/53	42/11091	4.547217e−05	3.208314e−04	2.499640e−04	ALB/HBB/HPX/AMBP
Common Pathway of Fibrin Clot Formation	3/53	22/11091	1.488196e−04	9.947417e−04	7.750163e−04	F10/SERPINA5/F2
Neutrophil degranulation	9/53	480/11091	4.001329e−04	2.540844e−03	1.979605e−03	ORM1/ORM2/A1BG/AHSG/CFD/CST3/GSN/HBB/TIMP2
HDL remodeling	2/53	10/11091	9.838576e−04	5.679542e−03	4.425006e−03	ALB/PLTP
Prednisone ADME	2/53	10/11091	9.838576e−04	5.679542e−03	4.425006e−03	ALB/SERPINA6
Metabolism of Angiotensinogen to Angiotensins	2/53	17/11091	2.910422e−03	1.607059e−02	1.252081e−02	AGT/CPB2
Gamma carboxylation, hypusinylation, hydroxylation, and arylsulfatase activation	3/53	61/11091	3.049920e−03	1.613916e−02	1.257423e−02	F9/F10/F2

Supplementary Table S1

Significantly enriched Reactome pathways, based on proteins showing longitudinal changes after Pegcetacoplan treatment (Friedman $p < 0.05$). Network visualization of the top 10 significantly enriched Reactome pathways is depicted in Figure 6 of the main manuscript.
